# Supplementary material for: Iron-catalyzed radical Markovnikov hydrohalogenation and hydroazidation of alkenes
Source: Nat Commun. 2024 Aug 22;15:7230. doi: 10.1038/s41467-024-51706-x (PMC11341871; doi:10.1038/s41467-024-51706-x)
Supplement: Supplementary file 1 — Supplementary Information [file 41467_2024_51706_MOESM1_ESM.pdf]

Supplementary Information

Iron-Catalyzed Radical Markovnikov Hydrohalogenation and Hydroazidation of Alkenes

Jonas Elfert, Nils Lennart Frye, Isabel Rempel, Constantin Gabriel Daniliuc and Armido Studer*

Institute of Organic Chemistry, University of Münster, Corrensstraße 36, 48149 Münster, Germany

studer@uni-muenster.de

Table of Contents

1. Supplementary Methods........2

1.1 General Information........2

1.2 General Procedures........3

1.3 Mechanistic Experiments and Proposal........5

1.4 Failed Substrates........9

1.5 Synthesis and Characterization of Starting Materials........10

1.6 Synthesis and Characterization of Hydration Products........13

1.7 X-Ray Data........32

2. Supplementary Tables........36

2.1 Optimization Studies........36

3. Supplementary Figures........43

3.1 NMR Spectra........43

4. Supplementary References........103

1. Supplementary Methods

1.1 General Information

General procedures and reactions with compounds sensitive to air or moisture were conducted in oven-dried glassware under an atmosphere of argon using Schlenk-technique. Anhydrous THF and Et_2O were freshly distilled from potassium under argon. Anhydrous dichloromethane was dried over P_4O_{10} and also freshly distilled prior to use. Other anhydrous solvents were purchased from *Thermo Scientific* in extra dry quality. All other solvents and reagents were used as received (unless otherwise noted) from *Sigma Aldrich, Fisher Thermo Scientific, BLDPharm, Fluorochem, TCI, ABCR, Strem, Biosynth and Indagoo.*

NMR: ^{1}H and ^{13}C spectra were recorded on a *Bruker* Avance II 300 or a *Bruker* AV 400 at 300 K. Chemical shifts (δ (ppm)) are reported relative to TMS (δ(^{1}H) 0.0 ppm, δ(^{13}C) 0.0 ppm). The solvents residual proton resonance and the respective carbon resonance ($CHCl_3$, δ(^{1}H) 7.26 ppm, δ(^{13}C) 77.16 ppm) were used for calibration.

Thin layer chromatography was done using *Merck* silica gel 60 F 254 plates; detection with UV light or by visualization with a $KMnO_4$ or in *p*-anisaldehyde stain.

Flash chromatography (FC) was carried out with silica gel 40-63 µm sourced from *Merck* or *VWR* at approximately 0.2 bar.

IR spectra were recorded as powder or film on a *Jasco* FT/IR-4600 spectrometer.

Melting points were determined on a *Bruker M-560* apparatus and are uncorrected.

HRMS ESI were recorded on a *Thermo Fisher Scientific* Orbitrap Velos Pro and *Thermo Fisher Scientific* LTQ Orbitrap XL spectrometer.

GC-FID GC-FID was conducted on an *Agilent GC 8860* or *Agilent GC 7890A*, Flame Ionization Detection (FID), carrier gas: H_2 Column: *Agilent HP-5*, Phenyl-Methyl Siloxan (30m x 320µm, 0,25µm Film). The method used starts with the injection temperature T_0 followed by heating the column to temperature T_1 (ramp) and this temperature is held for an additional time t_1 (T_0 = 50 °C, T_1 = 300 °C, ramp = 30 °C/min, t_1 = 15 min), constant flow 1,5 mL/min, split mode 15:1. (Standard dodecane peak comes at 3.04 min).

GC-MS chromatograms were recorded on an *Agilent Technologies 7820A* GC-system equipped with an *Agilent 5977B MSD(EI)* detector and a *HP-5MS* column with helium as carrier gas; the major signals are quoted as ratio of m/z in Daltons; the method used starts with the injection temperature T_0, after holding this temperature for 3 min, the column is heated to temperature T_1 (ramp) and this temperature is held for an additional time t (T_0 = 50 °C, T_1 = 300 °C, ramp = 10 °C/min or 30 °C/min, t = 15 min).

HRMS EI were recorded on a Thermo Fisher exactive GC.

1.2 General Procedures

Hydrobromination of terminal and 1,2-disubstituted alkenes (GP-Br-1)

$Fe(acac)_3$ (17.7 mg, 50.0 µmol, 10 mol%) was placed in an oven dried Schlenk tube under argon equipped with a magnetic stir bar and dissolved in dry methanol (4 mL). The alkene (0.50 mmol, 1.0 eq.) and methyl 2-bromo-2-methylpropanoate (78 µL, 0.60 mmol, 1.2 eq.) were added, followed by dropwise addition of phenylsilane (62 µL, 0.50 mmol, 1.0 eq.). The reaction was stirred for 48 h at room temperature. Afterwards, the solvent was evaporated and the residue was purified using flash chromatography to obtain the pure product.

Hydrobromination of 1,1-disubstituted and trisubstituted alkenes (GP-Br-2)

$Fe(dpm)_3$ (12.2 mg, 20.0 µmol, 10 mol%) was placed in an oven dried Schlenk tube under argon equipped with a magnetic stir bar and dissolved in dry *i*PrOH (1 mL) and dry THF (1 mL). The alkene (0.20 mmol, 1.0 eq.) and methyl 2-bromo-2-methylpropanoate (28 µL, 0.21 mmol, 1.1 eq.) were added, followed by dropwise addition of isopropoxy(phenyl)silane (39 µL, 0.22 mmol, 1.0 eq.). The reaction was stirred for 48 h at room temperature. Afterwards, the solvent was evaporated and the residue was purified using flash chromatography to obtain the pure product.

Hydroiodination of terminal and 1,2-disubstituted alkenes (GP-I)

If ethyl 2-iodo-propanoate has been stored for a longer time, it develops a purple color. The iodine impurity can be removed by dissolving in DCM, washing with aqueous sodium sulfite solution, drying over $MgSO_4$ and evaporating the solvent.

$Fe(acac)_3$ (17.7 mg, 50.0 µmol, 10 mol%) was placed in an oven dried Schlenk tube under argon equipped with a magnetic stir bar and dissolved in dry methanol (4 mL). The alkene (0.50 mmol, 1.0 eq.) and ethyl 2-iodo-propanoate (68 µL, 0.50 mmol, 1.0 eq.) were added, followed by dropwise addition of phenylsilane (62 µL, 0.50 mmol, 1.0 eq.). The reaction was stirred for 4 days at room temperature (alternatively 30 h at 40 °C). Afterwards, the solvent was evaporated and the residue was purified using flash chromatography to obtain the pure product.

Hydrochlorination of terminal and 1,2-disubstituted alkenes (GP-Cl-1)

$Fe(acac)_3$ (17.7 mg, 50.0 µmol, 10 mol%) was placed in an oven dried Schlenk tube under argon equipped with a magnetic stir bar and dissolved in dry *i*PrOH (2 mL) and dry THF (2 mL). The alkene (0.50 mmol, 1.0 eq.) and *p*TsCl (143 mg, 0.750 mmol, 1.5 eq.) were added, followed by dropwise addition of phenylsilane (246 µL, 2.00 mmol, 4.0 eq.). The reaction was stirred for 48 h at 40 °C. Afterwards, the solvent was evaporated and the residue was purified using flash chromatography to obtain the pure product.

Hydrochlorination of 1,1-disubstituted and trisubstituted alkenes (GP-Cl-2)

$Fe(dpm)_3$ (12.2 mg, 20.0 µmol, 10 mol%) was placed in an oven dried Schlenk tube under argon equipped with a magnetic stir bar and dissolved in dry *i*PrOH (1 mL) and dry THF (1 mL). The

alkene (0.20 mmol, 1.0 eq.) and *p*TsCl (57.2 mg, 0.300 mmol, 1.5 eq.) were added, followed by dropwise addition of isopropoxy(phenyl)silane (64 µL, 0.40 mmol, 2.0 eq.). The reaction was stirred for 24 h at room temperature. Afterwards, the solvent was evaporated and the residue was purified using flash chromatography to obtain the pure product.

Hydroazidation of alkenes (GP-N3)

$Fe(dpm)_3$ (12.1 mg, 20.0 µmol, 10 mol%) is placed in an oven dried Schlenk tube under argon equipped with a magnetic stir bar and dissolved in dry *i*PrOH (1 mL) and dry THF (1 mL). The alkene (0.20 mmol, 1.0 eq.) and pTsN_3 (59.2 mg, 0.300 mmol, 1.5 eq.) are added, followed by dropwise addition of isopropoxy(phenyl)silane (64 µL, 0.40 mmol, 2.0 eq.). The reaction is stirred for 24 h at room temperature. Afterwards, the solvent is evaporated and the residue purified using flash chromatography to obtain the pure product.

Procedure for single crystals from azides for X-ray analysis

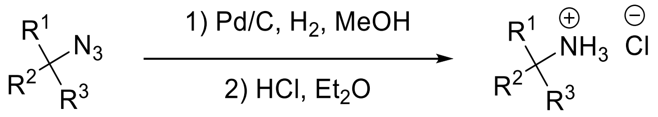

To determine the absolute configuration of low molecular weight azides, reduction to the amine followed by precipitation as hydrogen chloride salt was performed:

A sample of the azide (~0.1 mmol) is dissolved in a MeOH (~3 mL). Pd/C (10wt% in respect to the azide, 5wt% Pd/C was used) is added and the flask put under H_2 atmosphere with a balloon. After ~4 h (TLC check), the mixture is filtrated through celite and the solvent removed. The residue is redissolved in EtOAc and the amine extracted with 2M HCl. The combined aqueous phases are made strongly basic with 50% NaOH and the amine is reextracted with DCM. After drying over $MgSO_4$ and removal of the solvent, the residue is dissolved in Et_2O (1 mL) and 2M HCl in Et_2O (~0.5 mL) is added. This may or may not already lead to a precipitate. The mixture is left to evaporate in the fumehood overnight to get rid of the ether and additional HCl. The oily or solid residue is recrystallized from MeCN to yield suitable single crystals of the ammonium chloride salt for X-ray analysis.

1.3 Mechanistic Experiments and Proposal

Trapping of DLP-generated radicals with 2a

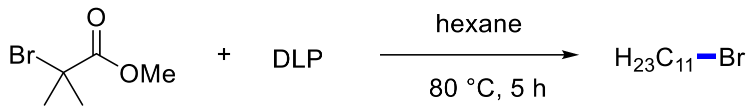

2a 0.5 eq. 20%

2a (78 µL, 0.60 mmol, 1.0 eq.) and dilauroylperoxide (120 mg, 0.300 mmol, 1.0 eq.) were dissolved in *n*-hexane (4 mL) and stirred at 80 °C for 5 h. The yield was determined by GC analysis using *n*-dodecane as internal standard.

Radical clock experiment

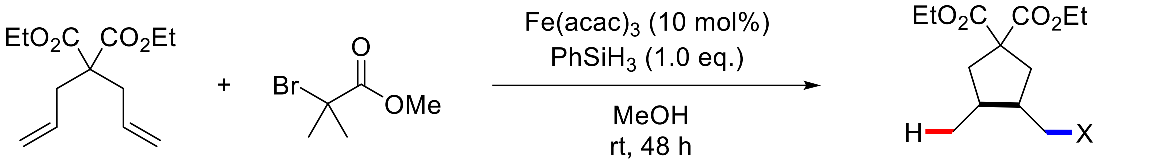

2a (1.2 eq.) **11,** 49% (*cis*:*trans* = 9.1:1)

The reaction was carried out according to **GP-Br-1** with diethyl 2,2-diallylmalonate (121 µL, 0.500 mmol, 1.0 eq.) as the starting material. Flash chromatography (pentane/Et_2O 95:5→92:8) gave an inseparable mixture of the diastereoisomers of **11** (78 mg, 0.24 mmol, 49%) as clear liquid. Diastereomeric ratio was determined by GC analysis. The non-cyclized product was not observed.

NMR data for major isomer: **^{1}H NMR** (400 MHz, $CDCl_3$) δ 4.17 (q, *J* = 7.1 Hz, 2H, OCH_2), 4.17 (q, *J* = 7.1 Hz, 2H, OCH_2), 3.40 – 3.27 (m, 2H), 2.51 – 2.41 (m, 3H), 2.31 (ddd, *J* = 12.9, 7.8, 6.3 Hz, 1H), 2.17 – 2.08 (m, 1H), 2.03 (dd, *J* = 13.9, 5.8 Hz, 1H), 1.23 (t, *J* = 7.1 Hz, 3H, OCH_2CH_3), 1.23 (t, *J* = 7.1 Hz, 3H, , OCH_2CH_3), 0.90 (d, *J* = 7.1 Hz, 3H, CH_3). **^{13}C NMR** (101 MHz, $CDCl_3$) δ 172.6, 172.6, 66.0, 61.6(6), 61.6(5), 59.0, 45.4, 41.3, 38.0, 36.0, 34.0, 14.5, 14.1. **FTIR** (neat): ν/cm^{-1} 2979*w*, 2965*w*, 1726*s*, 1465*w*, 1444*w*, 1384*w*, 1367*w*, 1249*s*, 1179*s*, 1147*m*, 1095*m*, 1043*m*, 858*m*, 643*m*. **HRMS** (ESI): calculated for $[C_{13}H_{21}O_4Br+Na^+]$: 343.05154; found: 343.05137.

Hydrofunctionalization of (−)-*α*-pinene

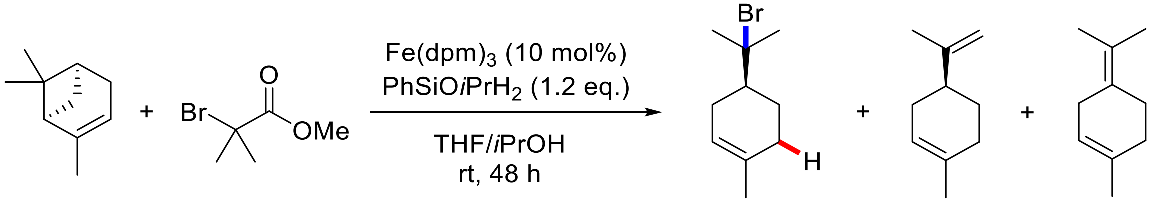

12 **2a** (1.1 eq.) **14**, ~30% **15a**, 39% **15b**, 9%

The reaction was carried out according to **GP-Br-2** with (−)-*α*-pinene (32 µL, 0.20 mmol, 1.0 eq.) as the starting material. Yields were determined by GC analysis. Conversion of 80% was observed by GC. Limonene (**15a**) and terpinolene (**15b**) result from radical ring opening of pinene. The

brominated species could not be isolated, decomposing to alkene during chromatography. Thus, the GC yield of **14** could only be estimated. Slightly larger amounts of the brominated species and lower amounts of limonene were observed when 3 eq. of **2a** were used. Hydrobrominated (–)-*α*-pinene was not observed. Evidently, the ring-opening is faster than the trapping with **2a**.

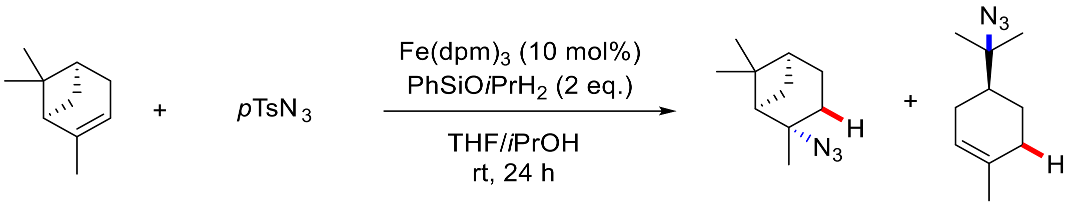

12 **6b** (1.5 eq.) **13a**, 29% **13b**, 29%

Attempting hydroazidation with (−)-*α*-pinene led to 64% conversion of starting material and around 29% isolated yield each of both expected azide of pinene **13a** as well as the azide of limonene **13b**, resulting from radical ring-opening. This in in line with the expected behaviour of *p*TsN_3 being a more efficient radical trap. Neither limonene nor terpinolene were observed by GC analysis. Pure spectra could not be obtained due to the products co-evaporating with the solvent and yields had to be estimated by calculating out the solvent.

13a: ^{1}**H NMR** (300 MHz, $CDCl_3$) δ 2.29 – 2.17 (m, 1H), 2.05 – 1.56 (m, 7H), 1.42 (s, 3H), 1.26 (s, 3H), 0.99 (s, 3H). ^{13}**C NMR** (76 MHz, $CDCl_3$) δ 67.8, 51.7, 40.4, 38.5, 29.0, 28.9, 28.0, 27.5, 25.3, 23.6.

The NMR data are in accordance with literature.[1]

13b: ^{1}**H NMR** (300 MHz, $CDCl_3$) δ 5.40 – 5.33 (m, 1H), 2.12 – 1.73 (m, 6H), 1.65 (s, 3H), 1.61 – 1.48 (m, 1H), 1.26 (s, 3H), 1.23 (s, 3H). ^{13}**C NMR** (76 MHz, $CDCl_3$) δ 134.1, 120.2, 64.3, 43.4, 30.9, 26.8, 24.2, 23.9, 23.3, 23.0.

The NMR data are in accordance with literature.[2]

Competitive hydrobromination and -iodination

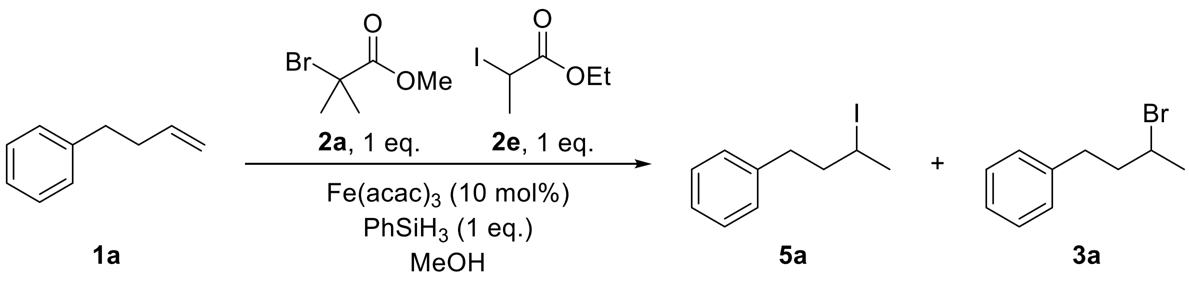

1a **5a** **3a**

The reaction was carried out according to **GP-I** on 0.1 mmol scale using but-3-en-1-ylbenzene (12 µL, 0.10 mmol, 1.0 eq.) and additionally adding methyl 2-bromo-2-methylpropanoate (13 µL, 0.10 mmol, 1 eq.) as second radical trap. The conversion and yield were followed by GC analysis. After 24 h, 53% conversion and 39% yield of **5a** were calculated. No formation of **3a** was observed. After 168 h, 62% conversion and 41% yield of **5a** were calculated. **3a** was not formed and no further conversion was observed. This is in line with the expectation of **2a** being a more efficient radical trap. Further, it supports the assumption of catalyst decomposition being the cause for the

longer reaction time for the hydroiodination. The reaction did not go to completion because of the higher amount of radical trap in relation to starting material for this experiment, leading to eventual decomposition of the catalyst.

Mechanistic proposal

Based on our findings and mechanistic investigations of a related reaction by Holland et al.,[3] we propose the mechanism depicted in Scheme S1. The iron hydride can react by HAT with an alkene **1** to form the metal alkyl complex **A**, which is in equilibrium with the radical **B**. The radical abstracts the halogen from our radical trap **2a** or **2b**, leading to the desired product **3** or **5**. The stabilized radical **C** can react by concerted proton-electron transfer with the metal-alcohol complex to reoxidize the catalyst. The iron(III) species can then react with the silane to reform the iron hydride. An alternative pathway would be the intermediate formation of ester enolate complex **D**, which is protonated to form the same iron(III) species.

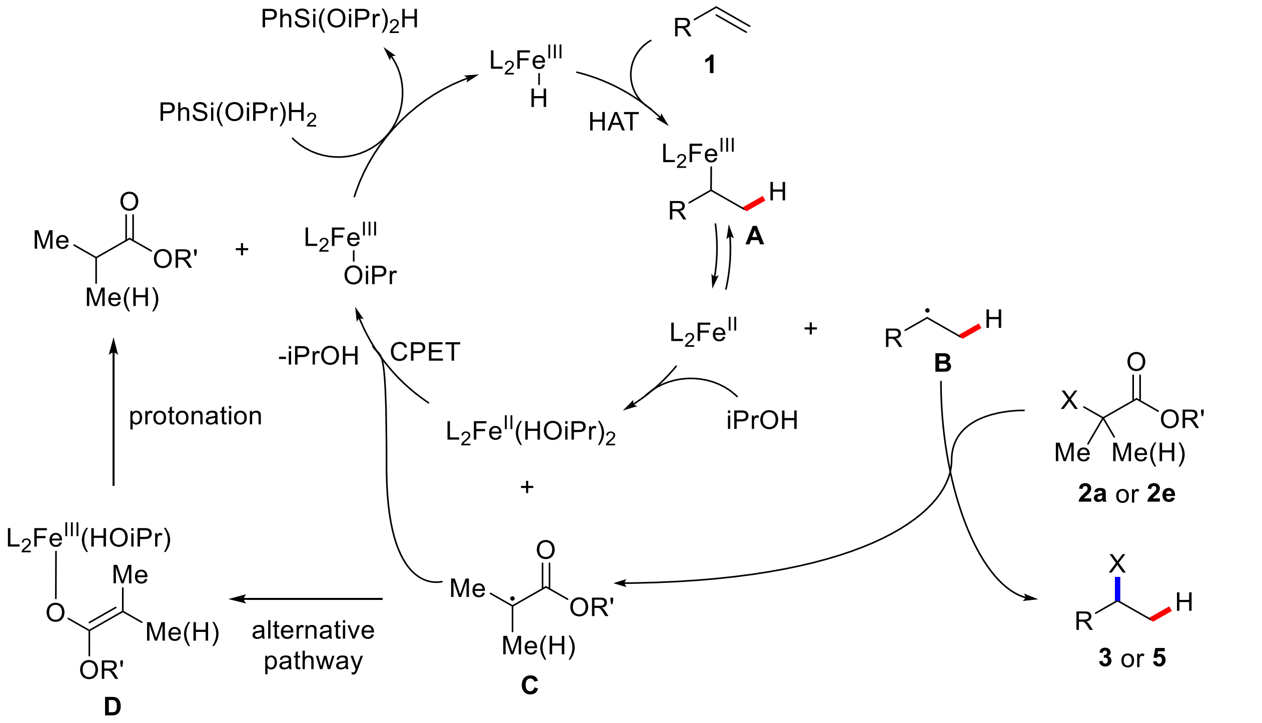

Supplementary Figure 1: Proposed mechanism for the hydrofunctionalization using ester-based radical traps. L = acac or dpm, X = Br or I, R' = Me or Et. HAT = hydrogen atom transfer. CPET = concerted proton-electron transfer.

For the hydroazidation and hydrochlorination we propose the mechanism depicted in Scheme S2. The catalyst performs the same steps, but is instead reoxidized by sulfonyl radical **C**. The mechanism of the reoxidation is not entirely clear. For a related hydroalkynylation, Shi and Zhao proposed the direct oxidation leading to the sulfinic acid (as depicted).[4] Carreira et al. proposed the decomposition of the sulfonyl radical to a sulfinyl radical for their cobalt-catalyzed hydrochlorination (not depicted), which could also play a role in the mechanism of our variant.[5]

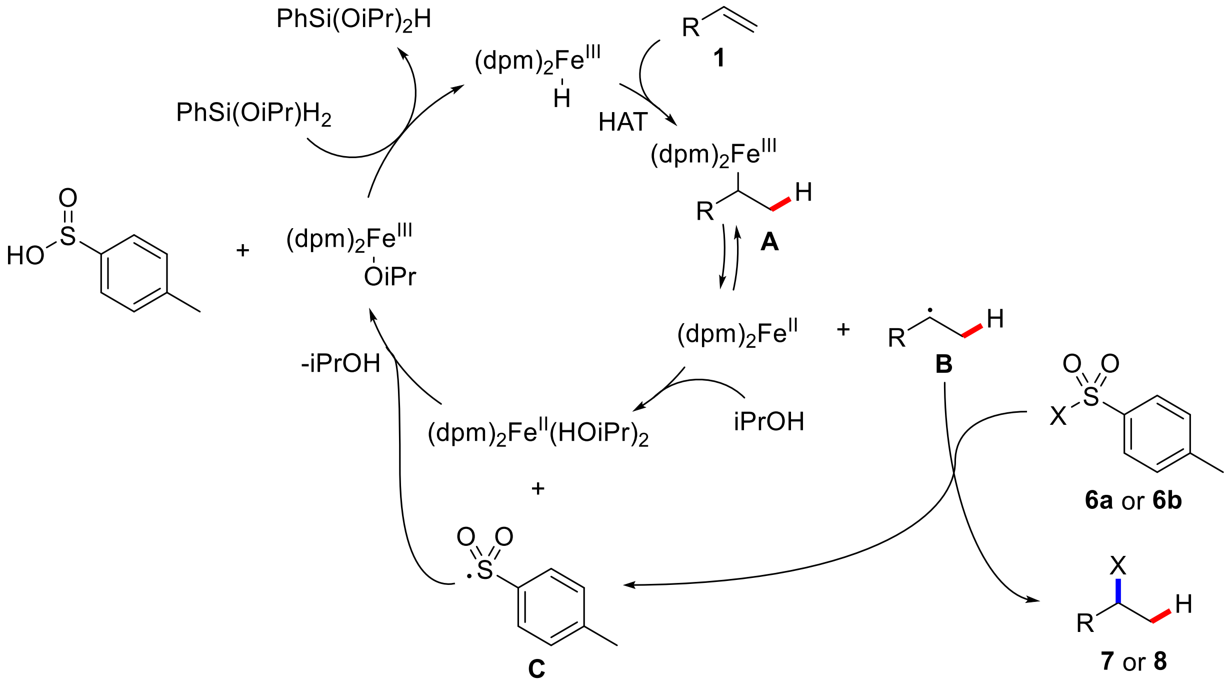

Supplementary Figure 2: Proposed mechanism for the hydrofunctionalization with tosyl-based radical traps. X = N_3 or Cl. dpm = dipivaloylmethane. HAT = hydrogen atom transfer.

The electron-withdrawing group appears to be key for catalytic turnover. In radical hydrofunctionalizations without addition of an oxidant, the radical trap is either based on EWG-substituted alkyls[6–9] or sulfonamides[4,10,11] to serve as oxidant for the iron(II)-species upon trapping. Some procedures are based on different oxidation mechanisms, although the reoxidizing radical is usually also of electrophilic nature for these reactions.[12–20]

1.4 Failed Substrates

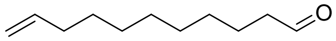

16

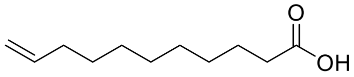

17

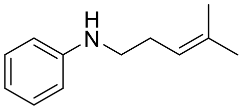

18

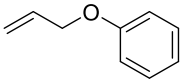

19

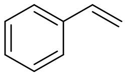

20

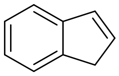

21

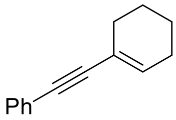

22

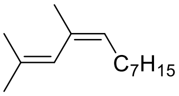

23

Supplementary Figure 3: Substrates that failed to yield hydrofunctionalization products.

Some functional groups were unfortunately not supported by the hydrofunctionalizations. Aldehyde **16** showed no product formation by GC analysis. For carboxylic acid **17** only traces of hydroazidation product were detected. Amine **18** displayed low conversion and only traces of hydrobromination and -azidation product as well. Allylic alcohols and ethers, like compound **19** showed high conversion by GC, but only traces of the desired product were observed. Styrene (**20**) and indene (**21**) showed no conversion. Presence of enynes (**22**) and 1,3-dienes (**23**) lead to no product formation, but instead to rapid formation of H_2 and high consumption of the silane component.

1.5 Synthesis and Characterization of Starting Materials

3-Methylbut-3-en-1-yl benzoate (1b)

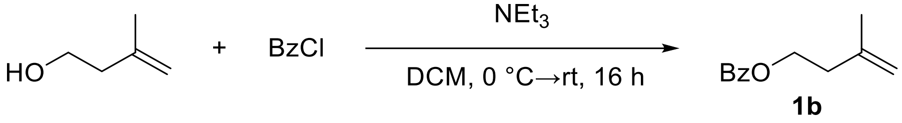

Isoprenol (2.5 mL, 22 mmol, 1.0 eq.) was dissolved in DCM (40 mL) and cooled to 0°C. BzCl (2.2 mL, 22 mmol, 1.0 eq.) was added, followed by dropwise addition of NEt_3 (6.1 mL, 44 mmol, 1.0 eq.). The ice bath was removed and the reaction stirred overnight. The reaction mixture was washed twice with 1M HCl and then with aq. sat. $NaHCO_3$. The organic phase was dried over $MgSO_4$ and the solvent evaporated. The residue was purified by flash chromatography (pentane/DCM 7:3). The product was received as a clear liquid (3.49 g, 18.3 mmol, 92%).

^{1}H NMR (300 MHz, $CDCl_3$) δ 8.06 – 8.01 (m, 2H, Ar$_H$), 7.59 – 7.51 (m, 1H, Ar$_H$), 7.48 – 7.39 (m, 2H, Ar$_H$), 4.89 – 4.81 (m, 1H, Alkenyl-C*H*), 4.83 – 4.80 (m, 1H, Alkenyl-C*H*), 4.44 (t, J = 6.8 Hz, 2H, OCH_2), 2.49 (t, J = 6.8 Hz, 2H, OCH$_2$CH_2), 1.82 (t, J = 1.2 Hz, 3H, CH_3).

The ^{1}H NMR data are in accordance with literature.[21]

***N*-(But-3-en-1-yl)-4-methylbenzenesulfonamide (1e)**

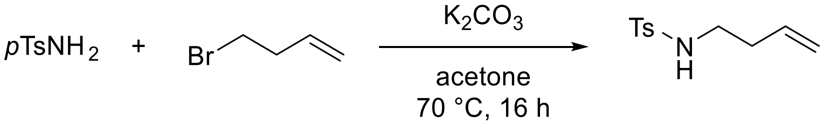

p-Toluenesulfonamide (1.9 g, 11 mmol, 1.1 eq.) and 4-bromobut-1-ene (1.0 mL, 10 mmol, 1.0 eq.) were dissolved in acetone (20 mL). K_2CO_3 (4.1 g, 30 mmol, 3.0 eq.) was added and the reaction stirred at 70 °C overnight. After cooling down, the mixture was filtrated through celite and the solvent evaporated. The residue was purified by flash chromatography (pentane/EtOAc 9:1→8:2) and the product was received as a yellow oil (1.2 g, 5.1 mmol, 51%).

^{1}H NMR (300 MHz, $CDCl_3$) δ 7.74 (d, *J* = 8.4 Hz, 2H, Ar$_H$), 7.31 (d, *J* = 7.9 Hz, 2H, Ar$_H$), 5.62 (ddt, *J* = 17.2, 10.4, 6.9 Hz, 1H, Alkenyl-C*H*CH$_2$), 5.12 – 5.06 (m, 1H, Alkenyl-CH*H*), 5.06 – 4.99 (m, 1H, Alkenyl-C*H*H), 4.39 (s br, 1H, N*H*), 3.07 – 2.95 (m, 2H, NCH_2), 2.43 (s, 3H, CH_3), 2.27 – 2.14 (m, 2H, NCH$_2$CH_2).

The ^{1}H NMR data are in accordance with literature.[22]

1-(4-(Pent-4-en-1-yloxy)phenyl)ethan-1-one (1h)

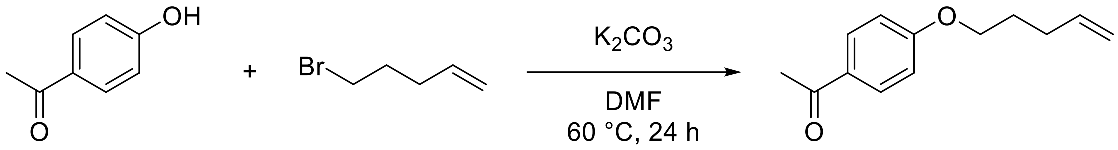

4-Hydroxyacetophenon (0.94 mL, 8.0 mmol, 1.0 eq.), 5-bromo-1-pentene (1.14 mL, 9.60 mmol, 1.2 eq.) and K_2CO_3 (1.33 g, 9.60 mmol, 1.2 eq.) were mixed in DMF (5 mL). The reaction mixture was stirred at 60 °C for 24 h and then water was added. The mixture was extracted with DCM and then the combined organic phases washed with 2M HCl two times. The organic phase was dried over $MgSO_4$ and the solvent removed. The residue was purified by flash chromatography (pentane/EtOAc 9:1). The product was obtained as clear liquid (1.54 g, 7.53 mmol, 94%).

^{1}H NMR (300 MHz, $CDCl_3$) δ 7.96 – 7.86 (m, 2H, Ar*H*), 6.95 – 6.85 (m, 2H, Ar*H*), 5.83 (ddt, *J* = 16.9, 10.2, 6.7 Hz, 1H, Alkenyl-C*H*), 5.11 – 4.91 (m, 2H, Alkenyl-CH_2), 4.01 (t, *J* = 6.4 Hz, 2H, OCH_2), 2.53 (s, 3H, CH_3), 2.29 – 2.13 (m, 2H, CHCH_2), 1.89 (tt, *J* = 7.2, 6.3 Hz, 2H, OCH$_2$CH_2).

The ^{1}H NMR data are in accordance with literature.[23]

Isopropoxy(phenyl)silane / RubenSilane (4)

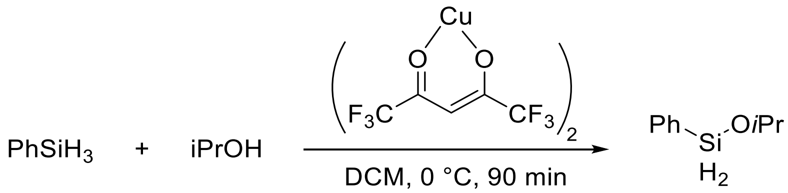

4

Isopropoxy(phenyl)silane was prepared according to a literature procedure.[24] In an oven dried Schlenk tube, Cu(hfac)$_2$•H_2O (297 mg, 0.600 mmol, 0.015 eq.) was dried at 80 °C in vacuum overnight (sublimes!). The flask was put under argon atmosphere and the blue Cu(hfac)$_2$ was dissolved in dry DCM (14 mL) and *i*PrOH (4.6 mL, 1.5 eq., 60 mmol) and the solution cooled to 0 °C. Phenylsilane (4.9 mL, 40 mmol, 1.0 eq.) was added at once and the reaction mixture stirred for 90 min. Afterwards, pentane was added (30 mL) and the mixture filtrated though celite. The solvent was removed and the residue was purified by micro distillation in vacuum (0.4 mbar, remaining $PhSiH_3$ evaporates at rt, product was distilled at 50 °C into a cooled receiving flask). The product was received as a clear liquid (4.52 g, 27.2 mmol, 68%) and stored in a Schlenk tube under argon in the fridge.

^{1}H NMR (300 MHz, $CDCl_3$) δ 7.70 – 7.62 (m, 2H, Ar*H*), 7.50 – 7.35 (m, 3H, Ar*H*), 5.03 (s, 2H, SiH_2), 4.09 (hept, J = 6.1 Hz, 1H, C*H*), 1.23 (d, J = 6.1 Hz, 6H, CH(CH_3)$_2$).

The ^{1}H NMR data are in accordance with literature.[24]

_p_-Tosylazide (6b)

$$p\text{TsCl} + \text{NaN}_3 \xrightarrow[\text{RT, 16 h}]{\text{Aceton/H}_2\text{O}} p\text{TsN}_3$$

*p*TsCl (3.81 g, 20.0 mmol, 1.0 eq.) was suspended in aceton (40 mL) and H_2O (40 mL) and cooled in an ice bath. NaN_3 (1.56 g, 24.0 mmol, 1.2 eq.) was added and the reaction mixture was stirred at room temperature overnight. The mixture was extracted 3x with EtOAc, the combined organic phases washed with brine, dried over $MgSO_4$ and the solvents evaporated. The resulting liquid is filtrated through a short column packed with silica using pentane/EtOAc 7:3 as eluent. After removal of solvent, the product is received as a clear liquid (3.73 g, 18.9 mmol, 95%).

^{1}H NMR (300 MHz, $CDCl_3$) δ 7.85 (d, *J* = 8.4 Hz, 2H, Ar_H), 7.41 (d, *J* = 8.1 Hz, 2H, Ar_H), 2.48 (s, 3H, CH_3).

The ^{1}H NMR data are in accordance with literature.[1]

1.6 Synthesis and Characterization of Hydration Products

(3-Bromobutyl)benzene (3a)

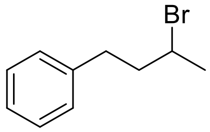

3a was prepared according to **GP-Br-1** using but-3-en-1-ylbenzene (75 µL, 0.50 mmol, 1.0 eq.) as the starting material. Flash chromatography (pentane) gave **3a** as a clear liquid (84.8 mg, 0.398 mmol, 80%).

^{1}H NMR (300 MHz, $CDCl_3$) δ 7.34 – 7.27 (m, 2H, Ar*H*), 7.25 – 7.17 (m, 3H, Ar*H*), 4.09 (dqd, *J* = 8.9, 6.7, 4.5 Hz, 1H, C*H*Br), 2.95 – 2.69 (m, 2H, Ph–C*H*$_2$), 2.23 – 1.98 (m, 2H, C*H*$_2$), 1.74 (d, *J* = 6.6 Hz, 3H, C*H*$_3$). **^{13}C NMR** (76 MHz, $CDCl_3$) δ 141.0, 128.7, 128.6, 126.2, 51.0, 42.8, 34.1, 26.7.

The NMR data are in accordance with literature.[25]

3-Bromo-3-methylbutyl benzoate (3b)

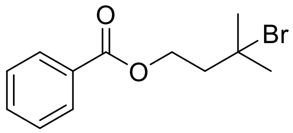

3b was prepared according to **GP-Br-2** using 3-methylbut-3-en-1-yl benzoate (38 µL, 0.20 mmol, 1.0 eq.) as the starting material. Flash chromatography (pentane/DCM 7:3) gave **3b** as a clear liquid (50.0 mg, 0.184 mmol, 92%).

^{1}H NMR (300 MHz, $CDCl_3$) δ 8.06 – 8.00 (m, 2H, Ar*H*), 7.61 – 7.53 (m, 1H, Ar*H*), 7.49 – 7.40 (m, 2H, Ar*H*), 4.59 (t, *J* = 6.8 Hz, 2H, OC*H*$_2$), 2.31 (t, *J* = 6.8 Hz, 2H, C*H*$_2$), 1.86 (s, 6H, 2xC*H*$_3$). **^{13}C NMR** (76 MHz, $CDCl_3$) δ 166.6, 133.2, 130.2, 129.7, 128.5, 64.5, 63.3, 45.6, 34.9.

The NMR data are in accordance with literature.[26]

1,10-Dibromoundecane (3c)

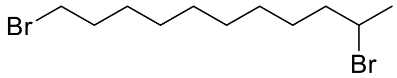

3c was prepared according to **GP-Br-1** using 11-bromoundec-1-ene (75 µL, 0.50 mmol, 1.0 eq.) as the starting material. Flash chromatography (pentane) gave **3c** as a clear liquid (133.5 mg, 0.4250 mmol, 85%).

^{1}H NMR (300 MHz, $CDCl_3$) δ 4.13 (dqd, *J* = 8.0, 6.7, 5.3 Hz, 1H, C*H*BrCH3), 3.40 (t, *J* = 6.8 Hz, 2H, C*H*$_2$Br), 1.92 – 1.72 (m, 4H, 2xC*H*$_2$), 1.70 (d, *J* = 6.7 Hz, 3H, C*H*$_3$), 1.51 – 1.35 (m, 4H, 2xC*H*$_2$), 1.29 (s, 8H, 4xC*H*$_2$). **^{13}C NMR** (76 MHz, $CDCl_3$) δ 52.1, 41.3, 34.2, 32.9, 29.5, 29.5, 29.1, 28.9, 28.3, 27.9, 26.6.

The NMR data are in accordance with literature.[27]

4-(2-Bromopropyl)-2-methoxyphenol (3d)

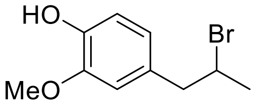

3d was prepared according to **GP-Br-1** using eugenol (77 µL, 0.50 mmol, 1.0 eq.) as the starting material. Flash chromatography (pentane/Et_2O 8:2) gave **3d** as a clear oil (88.7 mg, 0.363 mmol, 73%).

^{1}H NMR (300 MHz, $CDCl_3$) δ 6.89 – 6.82 (m, 1H, Ar*H*), 6.73 – 6.66 (m, 2H, Ar*H*), 5.57 (s br, 1H, O*H*), 4.35 – 4.18 (m, 1H, C*H*Br), 3.89 (s, 3H, OC*H*3), 3.16 (dd, *J* = 14.1, 6.9 Hz, 1H, C*H*H), 2.99 (dd, *J* = 14.1, 7.4 Hz, 1H, CH*H*), 1.69 (d, *J* = 6.6 Hz, 3H, C*H*3). **^{13}C NMR** (76 MHz, $CDCl_3$) δ 146.5, 144.6, 130.6, 122.1, 114.4, 111.8, 56.0, 51.2, 47.3, 25.7. **FTIR** (neat): *v*/cm^{-1} 3503*m*, 2967*w*, 2922*w*, 1607*w*, 1512*s*, 1450*m*, 1430*m*, 1375*m*, 1267*s*, 1234*s*, 1205*m*, 1173*m*, 1151*s*, 1123*s*, 1062*w*, 1032*s*, 999*m*, 935*w*, 898*w*, 873*w*, 850*w*, 817*m*, 794*s*, 740*w*, 650*w*, 632*w*, 557*m*. **HRMS** (ESI): calculated for $[(C_{10}H_{12}O_2Br)_2+Na^+]$: 510.99186; found: 510.99118.

***N*-(3-Bromobutyl)-4-methylbenzenesulfonamide (3e)**

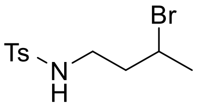

3e was prepared according to **GP-Br-1** using *N*-(but-3-en-1-yl)-4-methylbenzenesulfonamide (112.7 mg, 0.5000 mmol, 1.0 eq.) as the starting material. Flash chromatography (pentane/EtOAc 90:10→85:15) gave **3e** as a white solid (102.2 mg, 0.3337 mmol, 67%).

^{1}H NMR (300 MHz, $CDCl_3$) δ 7.75 (d, *J* = 8.3 Hz, 2H, Ar*H*), 7.31 (d, *J* = 8.4 Hz, 2H, Ar*H*), 4.94 (s br, 1H, N*H*), 4.13 (dqd, *J* = 13.4, 6.7, 4.1 Hz, 1H, C*H*Br), 3.20 – 2.97 (m, 2H, NC*H*2), 2.43 (s, 3H, Ar–C*H*3), 2.09 – 1.80 (m, 2H, NCH_2CH_2), 1.66 (d, *J* = 6.7 Hz, 3H, C*H*3). **^{13}C NMR** (76 MHz, $CDCl_3$) δ 143.7, 136.8, 129.9 (2xC_{Ar}), 127.2 (2xC_{Ar}), 48.0, 41.8, 40.7, 26.5, 21.7. **FTIR** (neat): *v*/cm^{-1} 3291*s*, 1458*w*, 1440*w*, 1413*w*, 1382*w*, 1323*s*, 1301*s*, 1246*m*, 1182*w*, 1149*s*, 1133*m*, 1082*s*, 1047*m*, 1018*m*, 1004*m*, 910*w*, 857*m*, 813*s*, 760*s*, 704*w*, 668*s*, 664*s*, 550*s*. **HRMS** (ESI): calculated for $[C_{11}H_{16}NO_2SBr+Na^+]$: 327.99773; found: 327.99740. **M.p.**: 65.5-66.4 °C.

((4-Bromopentyl)oxy)(tert-butyl)dimethylsilane (3f)

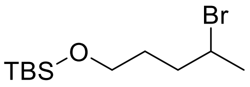

3f was prepared according to **GP-Br-1** using tert-butyldimethyl(pent-4-en-1-yloxy)silane (100 mg, 0.500 mmol, 1.0 eq.) as the starting material. Flash chromatography (pentane/DCM 95:5→90:10) gave **3f** as a clear liquid (87.0 mg, 0.309 mmol, 62%).

^{1}H NMR (300 MHz, $CDCl_3$) δ 4.23 – 4.10 (m, 1H, C*H*Br), 3.63 (t, *J* = 6.1 Hz, 2H, OC*H*2), 1.92 – 1.81 (m, 2H, OCH_2CH_2), 1.72 (d, *J* = 6.6 Hz, 3H, C*H*3), 1.83 – 1.52 (m, 2H $BrCHCH_2$), 0.89 (s, 9H, $(CH_3)_3$), 0.05 (s, 6H, $Si(CH_3)_2$). **^{13}C NMR** (76 MHz, $CDCl_3$) δ 62.5, 51.9, 37.8, 31.1, 26.7, 26.1 (3xCH_3), 18.5, -5.2 (2xCH_3). **^{29}Si NMR** (60 MHz, $CDCl_3$) δ 18.9. **FTIR** (neat): *v*/cm^{-1} 2953*m*, 2928*m*, 2886*w*, 2857*m*, 1472*m*, 1445*w*, 1378*w*, 1361*w*, 1253*s*, 1217*w*, 1051*s*, 1004*s*, 939*w*, 834*s*, 776*s*, 695*w*, 668*m*, 616*w*, 575*w*, 536*w*. **HRMS** (EI): calculated for $C_7H_{16}OSiBr$ (M-*t*Bu): 223.01483; found: 223.01400.

2-(7-Bromooctyl)oxirane (3g)

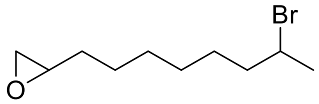

3g was prepared according to **GP-Br-1** using 2-(oct-7-en-1-yl)oxirane (92 µL, 0.50 mmol, 1.0 eq.) as the starting material. Flash chromatography (pentane/Et_2O 95:5→90:10) gave **3g** as a clear liquid (85.5 mg, 0.362 mmol, 72%, 1:1 mixture of diastereoisomers).

^{1}H NMR (400 MHz, $CDCl_3$) δ 4.12 (dqd, *J* = 8.3, 6.6, 5.1 Hz, 1H, C*H*Br), 2.89 (tdd, *J* = 5.1, 3.9, 2.7 Hz, 1H, OC*H*), 2.73 (dd, *J* = 5.1, 3.9 Hz, 1H, OC*H*H), 2.45 (dd, *J* = 5.0, 2.8 Hz, 1H, OCH*H*), 1.88 – 1.71 (m, 2H, OCH_2CH_2), 1.69 (d, *J* = 6.6 Hz, 3H, CH_3), 1.57 – 1.25 (m, 10H, 5xCH_2). **^{13}C NMR** (76 MHz, $CDCl_3$) δ 52.5, 52.0, 47.2, 41.2, 32.6, 29.4, 29.0, 27.8, 26.6, 26.0. **FTIR** (neat): *v*/cm^{-1} 2970*w*, 2926*s*, 2857*s*, 1457*m*, 1378*w*, 1260*m*, 1219*m*, 1130*m*, 914*m*, 833*s*, 724*m*, 668*s*, 618*m*, 531*s*. **HRMS** (ESI): calculated for [$C_{10}H_{19}OBr+Na^+$]: 259.04917; found: 259.04900.

1-(4-((4-Bromopentyl)oxy)phenyl)ethan-1-one (3h)

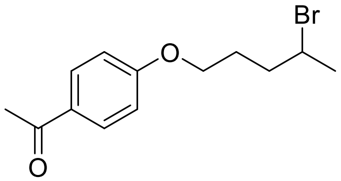

3h was prepared according to **GP-Br-1** using 1-(4-(pent-4-en-1-yloxy)phenyl)ethan-1-one (102.1 mg, 0.5000 mmol, 1.0 eq.) as the starting material. Flash chromatography (pentane/EtOAc 9:1) gave **3h** as a clear liquid (115.5 mg, 0.4050 mmol, 81%).

^{1}H NMR (300 MHz, $CDCl_3$) δ 7.97 – 7.85 (m, 2H, Ar_H), 6.95 – 6.85 (m, 2H, Ar_H), 4.27 – 4.11 (m, 1H, BrC*H*), 4.09 – 3.97 (m, 2H, OCH_2), 2.54 (s, 3H, C(=O)CH_2), 2.13 – 1.87 (m, 4H, 2xCH_2), 1.75 (d, *J* = 6.7 Hz, 3H, CH_3). **^{13}C NMR** (76 MHz, $CDCl_3$) δ 196.9, 162.9, 130.7 (2xCH), 130.4, 114.2 (2xCH), 67.4, 51.2, 37.6, 27.6, 26.7, 26.5. **FTIR** (neat): *v*/cm^{-1} 2955*w*, 1920*m*, 2871*w*, 2359*m*, 2341*m*, 1672*s*, 1598*s*, 1575*m*, 1559*w*, 1541*w*, 1508*m*, 1472*w*, 1419*w*, 1357*m*, 1306*w*, 1249*s*, 1169*s*, 1115*w*, 1019*m*, 982*w*, 956*m*, 831*s*, 758*w*, 589*s*, 536*w*, 502*w*. **HRMS** (ESI): calculated for [$C_{13}H_{17}O_2Br+Na^+$]: 307.03041; found: 307.03040.

Bromocyclododecane (3i)

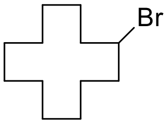

3i was prepared according to **GP-Br-1** using cyclododecene (96 µL, 0.50 mmol, 1.0 eq.) as the starting material. Flash chromatography (pentane) gave **3i** as a clear liquid (77.5 mg, 0.314 mmol, 63%).

^{1}H NMR (300 MHz, $CDCl_3$) δ 4.25 (tt, *J* = 7.3, 5.4 Hz, 1H, C*H*Br), 2.15 – 1.96 (m, 2H, 2xCBrCH*H*), 1.95 – 1.79 (m, 2H, 2xCBrC*H*H), 1.60 – 1.17 (m, 18H, Alkyl-*H*). **^{13}C NMR** (76 MHz, $CDCl_3$) δ 54.2, 34.8 (2xCH_2), 23.8(0) (2xCH_2), 23.7(8) (2xCH_2), 23.5 (3xCH_2), 22.9 (2xCH_2).

The NMR data are in accordance with literature.[28]

9-Bromotricosane (3j) / 10-Bromotricosan (3j’)

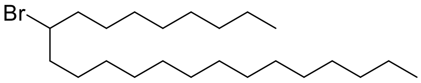

3j

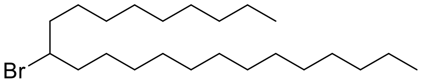

3j'

3j/**3j’** was prepared according to **GP-Br-1** using (*Z*)-tricos-9-ene (200 µL, 0.500 mmol, 1.0 eq.) as the starting material and using Methanol/THF (1:1) as solvent due to insolubility in Methanol. Flash chromatography (pentane) gave **3j** and **3j’** as inseparable 1:1 mixture as a clear liquid (135.3 mg, 0.3354 mmol, 67%).

^{1}H NMR (300 MHz, $CDCl_3$) δ 4.12 – 3.96 (m, 1H, C*H*Br), 1.92 – 1.68 (m, 4H, 2xCBrCH_2), 1.62 – 0.97 (m, 36H, Alkyl-*H*), 0.88 (t, *J* = 6.8 Hz, 6H, 2xCH_3). **^{13}C NMR** (76 MHz, $CDCl_3$) δ 59.2, 39.3, 32.1, 32.0(4), 32.0(1), 29.9, 29.8(2), 29.8(0), 29.7(4), 29.7(0), 29.6(6), 29.6(2), 29.5(3), 29.4(6), 29.4(1), 29.2, 27.7, 22.9, 22.8, 14.3. **FTIR** (neat): ν/cm^{-1} 2921*s*, 2852*s*, 2360*m*, 2341*m*, 1734*w*, 1716*w*, 1699*w*, 1684*w*, 1670*w*, 1653*w*, 1635*w*, 1558*w*, 1541*w*, 1521*w*, 1507*w*, 1497*w*, 1489*w*, 1457*w*, 1437*w*, 1419*w*, 1396*w*, 1375*w*, 986*m*, 907*m*, 735*m*, 617*w*, 537*w*. **HRMS** (EI): no undecomposed product detected, same spectrum as **7j**.

2-(3-Bromo-3-methylbutyl)isoindoline-1,3-dione (3k)

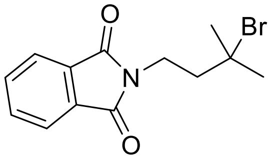

3k was prepared according to **GP-Br-2** using 2-(3-methylbut-3-en-1-yl)isoindoline-1,3-dione (43.1 mg, 0.200 mmol, 1.0 eq.) as the starting material. Flash chromatography (pentane/EtOAc 95:5→90:10) gave **3k** as a white solid (53.0 mg, 0.179 mmol, 89%).

^{1}H NMR (300 MHz, $CDCl_3$) δ 7.89 – 7.79 (m, 2H, Ar$_{H}$), 7.76 – 7.67 (m, 2H, Ar$_{H}$), 3.99 – 3.88 (m, 2H, NCH_2), 2.22 – 2.11 (m, 2H, CH_2), 1.83 (s, 6H, 2xCH_3). **^{13}C NMR** (76 MHz, $CDCl_3$) δ 168.3 (2xC$_q$), 134.1 (2xCH), 132.2 (2xC$_q$), 123.4 (2xCH), 63.7, 44.8, 35.8, 34.4 (2xCH_3).

The NMR data are in accordance with literature.[29]

tert-Butyl 4-bromo-4-methylpiperidine-1-carboxylate (3l)

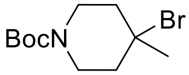

3l was prepared according to **GP-Br-2** using *tert*-butyl 4-methylenepiperidine-1-carboxylate (41 µL, 0.20 mmol, 1.0 eq.) as the starting material. Flash chromatography (pentane/DCM 8:2) gave **3l** as a clear oil (51.3 mg, 0.184 mmol, 92%).

^{1}H NMR (300 MHz, $CDCl_3$) δ 3.99 (s br, 2H, 2xNC*H*H), 3.14 (s br, 2H, 2xNCH*H*), 2.04 – 1.92 (m, 2H, 2x CH*H*), 1.85 (s, 3H, CH_3), 1.63 – 1.49 (m, 2H, 2x C*H*H), 1.45 (s, 9H, C(CH_3)$_3$). **^{13}C NMR** (76 MHz, $CDCl_3$) δ 154.8, 79.8, 68.0, 41.6 (br, 4xCH_2 overlapped), 35.2, 28.6 (3xCH_3).

The NMR data are in accordance with literature.[30]

cis-4-Bromo-4-methylcyclohexyl)benzene (3n)

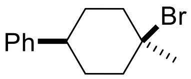

3n was prepared according to **GP-Br-2** using (4-methylenecyclohexyl)benzene (37 µL, 0.20 mmol, 1.0 eq.) as the starting material. Flash chromatography (pentane) gave **3n** as a clear liquid (38.9 mg, 0.154 mmol, 77%, single diastereoisomers, *dr* > 20:1).

Diastereomeric ratio was determined by GC analysis of the crude reaction mixture. The major isomer was assigned by comparison with **8n** and confirmed by comparison with the literature ^{1}H NMR.[31]

^{1}H NMR (300 MHz, $CDCl_3$) δ 7.42 – 7.22 (m, 5H, Ar_H), 2.54 (tt, *J* = 12.3, 3.7 Hz, 1H, Ar–C*H*), 2.33 – 2.22 (m, 2H, 2xC*H*H), 2.20 – 2.01 (m, 2H, 2xCH*H*), 1.96 (s, 3H, CH_3), 1.92 – 1.80 (m, 2H, 2xC*H*H), 1.62 (ddd, *J* = 14.5, 12.5, 3.9 Hz, 2H, 2xCH*H*). **^{13}C NMR** (76 MHz, $CDCl_3$) δ 146.8, 128.6 (2xCH), 127.1 (2xCH), 126.3, 71.0, 43.6, 43.2 (2xCH_2), 36.0, 31.0 (2xCH_2).

The NMR data are in accordance with literature.[31]

(3-Iodobutyl)benzene (5a)

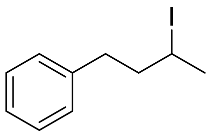

5a was prepared according to **GP-I** using but-3-en-1-ylbenzene (75 µL, 0.50 mmol, 1.0 eq.) as the starting material. Flash chromatography (pentane) gave **5a** as a clear liquid (94.2 mg, 0.362 mmol, 72%).

^{1}H NMR (300 MHz, $CDCl_3$) δ 7.35 – 7.27 (m, 2H, Ar_H), 7.25 – 7.18 (m, 3H, Ar_H), 4.12 (dqd, *J* = 9.2, 6.9, 4.5 Hz, 1H, IC*H*), 2.86 (ddd, *J* = 14.1, 9.0, 5.2 Hz, 1H, Ph–C*H*H), 2.70 (ddd, *J* = 13.8, 9.0, 6.9 Hz, 1H, Ph–CH*H*), 2.26 – 2.09 (m, 1H, C*H*H), 1.96 (d, *J* = 6.8 Hz, 3H, CH_3), 1.94 – 1.82 (m, 1H, CH*H*). **^{13}C NMR** (76 MHz, $CDCl_3$) δ 140.9, 128.7 (2xCH), 128.6 (2xCH), 126.3, 44.5, 36.0, 29.8, 29.1.

The NMR data are in accordance with literature.[32]

1-Bromo-10-iodoundecane (5c)

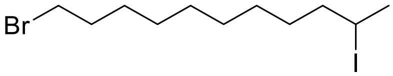

5c was prepared according to **GP-I** using 11-bromoundec-1-ene (75 µL, 0.50 mmol, 1.0 eq.) as the starting material. Flash chromatography (pentane) gave **5c** as a clear liquid (110.6 mg, 0.3063 mmol, 61%).

^{1}H NMR (300 MHz, $CDCl_3$) δ 4.18 (dqd, *J* = 8.6, 6.8, 5.0 Hz, 1H, IC*H*), 3.40 (t, *J* = 6.8 Hz, 2H, BrCH_2), 1.91 (d, *J* = 6.9 Hz, 3H, CH_3), 1.88 – 1.74 (m, 3H, CH_2+CH*H*), 1.66 – 1.51 (m, 1H, C*H*H), 1.51 – 1.33 (m, 4H, 2xCH_2), 1.29 (s, 8H, 4xCH_2). **^{13}C NMR** (76 MHz, $CDCl_3$) δ 43.0, 34.2, 32.9, 31.0, 29.8, 29.5 (2 signals overlapped), 29.1, 28.8, 28.8, 28.3. **FTIR** (neat): *v*/cm^{-1} 2923*s*, 2853*s*, 1457*m*, 1441*m*, 1377*m*, 1262*w*, 1246*w*, 1206*w*, 117*w*, 1135*m*, 722*m*, 645*m*, 560*m*, 486*m*. **HRMS** (EI): calculated for $C_{11}H_{22}BrI$: 359.99441; found: 359.99405.

4-(2-Iodopropyl)-2-methoxyphenol (5d)

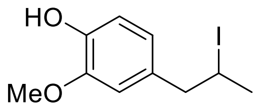

5d was prepared according to **GP-I** using eugenol (77 µL, 0.50 mmol, 1.0 eq.) as the starting material. Flash chromatography (pentane/Et_2O 9:1) gave **5d** as a clear liquid (96.0 mg, 0.329 mmol, 66%).

^{1}H NMR (300 MHz, $CDCl_3$) δ 6.88 – 6.81 (m, 1H, Ar*H*), 6.72 – 6.64 (m, 2H, Ar*H*), 5.53 (s br, 1H, O*H*), 4.39 – 4.21 (m, 1H, IC*H*), 3.89 (s, 3H, OC*H*$_3$), 3.23 (dd, *J* = 14.2, 7.1 Hz, 1H, C*H*H), 2.98 (dd, *J* = 14.1, 7.7 Hz, 1H, CH*H*), 1.89 (d, *J* = 6.7 Hz, 3H, C*H*$_3$). **^{13}C NMR** (76 MHz, $CDCl_3$) δ 146.5, 144.6, 131.8, 121.9, 114.4, 111.6, 56.1, 49.3, 29.4, 28.1. **FTIR** (neat): *v*/cm^{-1} 3486*m*, 2962*m*, 2920*m*, 2843*w*, 1605*w*, 1512*s*, 1463*m*, 1449*m*, 1430*m*, 1374*m*, 1266*s*, 1234*s*, 1203*s*, 1148*s*, 1121*s*, 1061*m*, 1031*s*, 989*w*, 933*w*, 895*w*, 816*m*, 793*s*, 738*m*, 642*m*, 555*m*. **HRMS** (ESI): calculated for [$C_{10}H_{13}O_2I+Na^+$]: 314.98524; found: 314.98526.

***N*-(3-Iodobutyl)-4-methylbenzenesulfonamide (5e)**

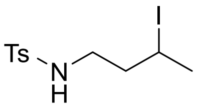

5e was prepared according to **GP-I** using *N*-(but-3-en-1-yl)-4-methylbenzenesulfonamide (112.7 mg, 0.5000 mmol, 1.0 eq.) as the starting material. Flash chromatography (pentane/EtOAc 90:10→85:15) gave **5e** as a pale-yellow solid (126.6 mg, 0.3584 mmol, 72%)

^{1}H NMR (300 MHz, $CDCl_3$) δ 7.81 – 7.71 (m, 2H, Ar*H*), 7.36 – 7.29 (m, 2H, Ar*H*), 4.84 (t br, *J* = 6.4 Hz, 1H, N*H*), 4.14 (dqd, *J* = 9.4, 6.8, 4.4 Hz, 1H, IC*H*), 3.20 – 3.07 (m, 1H, C*H*H), 3.06 – 2.92 (m, 1H, CH*H*), 2.43 (s, 3H, Ar–C*H*$_3$), 1.88 (d, *J* = 6.9 Hz, 3H, C*H*$_3$), 2.01 – 1.71 (m, 2H, C*H*$_2$). **^{13}C NMR** (76 MHz, $CDCl_3$) δ 143.8, 136.8, 129.9 (2xCH), 127.2 (2xCH), 43.6, 42.3, 28.9, 25.5, 21.7. **FTIR** (neat): *v*/cm^{-1} 3295*s*, 2981*w*, 2927*w*, 1445*w*, 1412*w*, 1382*w*, 1322*m*, 1301*m*, 1234*m*, 1147*s*, 1125*m*, 1080*s*, 1018*m*, 1002*m*, 905*w*, 854*m*, 813*s*, 757*s*, 664*s*, 616*w*, 553*m*, 539*s*. **HRMS** (ESI): calculated for [$C_{11}H_{16}NO_2SI+Na^+$]: 375.98386; found: 375.98397. **M.p.**: 78.6-80.3 °C.

2-(7-Iodooctyl)oxirane (5g)

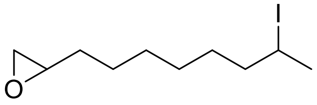

5g was prepared according to **GP-I** using 2-(oct-7-en-1-yl)oxirane (92 µL, 0.50 mmol, 1.0 eq.) as the starting material. Flash chromatography (pentane/Et_2O 95:5) gave **5g** as a clear liquid (61.0 mg, 0.220 mmol, 43%, 1:1 mixture of diastereoisomers).

^{1}H NMR (400 MHz, $CDCl_3$) δ 4.18 (dqd, *J* = 8.5, 6.8, 4.9 Hz, 1H, C*H*I), 2.90 (tdd, *J* = 5.1, 3.9, 2.7 Hz, 1H, OC*H*), 2.74 (dd, *J* = 5.1, 3.9 Hz, 1H, OCH*H*), 2.46 (dd, *J* = 5.1, 2.7 Hz, 1H, OC*H*H), 1.91 (d, *J* = 6.8 Hz, 3H, C*H*$_3$), 1.89 – 1.77 (m, 1H, OCHCH*H*), 1.65 – 1.58 (m, 1H, OCHC*H*H), 1.55 – 1.23 (m, 10H, 5xC*H*$_2$). **^{13}C NMR** (101 MHz, $CDCl_3$) δ 52.5, 47.2, 43.0, 32.6, 30.8, 29.8, 29.4, 29.1, 28.8, 26.0. **FTIR** (neat): *v*/cm^{-1} 2979*w*, 2926*s*, 2855*s*, 1457*m*, 1409*w*, 1378*m*,1260*w*, 1192*w*, 1131*m*, 914*m*, 831*s*, 804*w*, 725*m*, 585*w*, 487*m*. **HRMS** (ESI): calculated for [$C_{10}H_{19}OI+Na^+$]: 305.03728; found: 305.03729.

1-(4-((4-Iodopentyl)oxy)phenyl)ethan-1-one (5h)

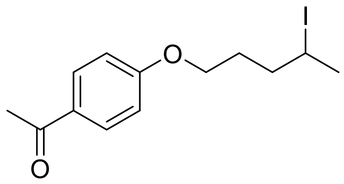

5h was prepared according to **GP-I** using 1-(4-pent-4-enoxyphenyl)ethanone (102.1 mg, 0.50 mmol, 1.0 eq.) as the starting material. Flash chromatography (pentane/EtOAc 95:5) gave **5h** as a clear liquid (101.8 mg, 0.3064 mmol, 61%).

^{1}H NMR (300 MHz, $CDCl_3$) δ 7.98 – 7.85 (m, 2H, Ar$_H$), 6.97 – 6.84 (m, 2H, Ar$_H$), 4.31 – 4.14 (m, 1H, IC*H*), 4.11 – 3.96 (m, 2H, OCH_2), 2.55 (s, 3H,C(=O)CH_3), 1.96 (d, *J* = 6.8 Hz, 3H, CH_3), 2.11 – 1.77 (m, 4H, 2xCH_2). **^{13}C NMR** (76 MHz, $CDCl_3$) δ 196.9, 162.9, 130.7 (2xCH), 130.4, 114.2 (2xCH), 67.2, 39.4, 29.6 (2xCH_2), 29.1, 26.5. **FTIR** (neat): *v*/cm^{-1} 2953*w*, 2918*w*, 2872*w*, 1672*s*, 1598*s*, 1575*m*, 1508*m*, 1469*w*, 1442*w*, 1419*w*, 1357*m*, 1306*w*, 1249*s*, 1169*s*, 1130*m*, 1115*m*, 1064*w*, 1019*m*, 979*w*, 955*m*, 831s, 756*w*, 701*w*, 589s, 499*m*. **HRMS** (ESI): calculated for [$C_{13}H_{17}O_2I+Na^+$]: 355.01654; found: 355.01657.

Iodocyclododecane (5i)

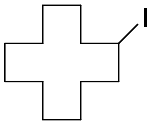

5i was prepared according to **GP-I** using cyclododecene (96 µL, 0.50 mmol, 1.0 eq.) as the starting material. Flash chromatography (pentane) gave **5i** as a clear liquid (55.3 mg, 0.188 mmol, 38%).

^{1}H NMR (300 MHz, $CDCl_3$) δ 4.35 (tt, *J* = 7.4, 5.6 Hz, 1H, IC*H*), 2.16 – 1.87 (m, 4H, 2xICHCH_2), 1.58 – 1.39 (m, 5H, Alkyl-*H*), 1.40 – 1.19 (m, 13H, Alkyl-*H*). **^{13}C NMR** (76 MHz, $CDCl_3$) δ 36.6 (2xCH_2), 34.1, 24.3 (2xCH_2), 23.9, 23.6(5) (4xCH_2), 23.5(6) (2xCH_2).

The NMR data are in accordance with literature.[33]

9-Iodotricosane (5j) / 10-Iodotricosane (5j')

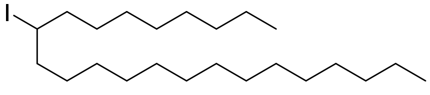

5j

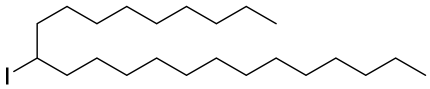

5j'

5j/**5j'** was prepared according to **GP-I** using (*Z*)-tricos-9-ene (200 µL, 0.50 mmol, 1.0 eq.) as the starting material and Methanol/THF (1:1) as solvent due to solubility. Flash chromatography (pentane) gave **5j** and **5j'** as inseparable 1:1 mixture as a clear liquid (76.3 mg, 0.169 mmol, 34%).

^{1}H NMR (300 MHz, $CDCl_3$) δ 4.12 (tt, *J* = 8.7, 4.7 Hz, 1H, CI*H*), 1.93 – 1.76 (m, 2H, 2xCICH*H*), 1.76 – 1.60 (m, 2H, 2xCIC*H*H), 1.60 – 1.12 (m, 36H, Alkyl-*H*), 0.88 (t, *J* = 6.8 Hz, 6H, 2xCH_3). **^{13}C NMR** (76 MHz, $CDCl_3$) δ 41.0, 40.8, 32.1, 32.0(4), 32.0(1), 29.8(4), 29.8(1), 29.7(8), 29.7(4), 29.7(0), 29.6(7), 29.6(4), 29.6(0), 29.5(2), 29.4(5), 29.4(1), 29.0, 22.8, 14.3. **FTIR** (neat): *v*/cm^{-1} 2921*s*, 2851*s*, 2360*m*, 2341*m*, 1734*w*, 1716*w*, 1699*w*, 1684*w*, 1653*w*, 1635*w*, 1558*w*, 1541*w*, 1521*w*, 1507*w*, 1489*w*, 1457*w*, 1437*w*, 1419*w*, 1375*w*, 988*w*, 907*w*, 735*m*, 721*m*, 591*w*. **HRMS** (EI): no undecomposed product detected, same spectrum as **7j**.

(3-Chlorobutyl)benzene (7a)

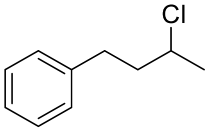

7a was prepared according to **GP-Cl-1** using but-3-en-1-ylbenzene (75 µL, 0.50 mmol, 1.0 eq.) as the starting material. Flash chromatography (pentane/DCM 99:1) gave **7a** as a clear liquid (52.0 mg, 0.308 mmol, 62%).

^{1}H NMR (300 MHz, $CDCl_3$) δ 7.37 – 7.28 (m, 2H, Ar$_H$), 7.27 – 7.18 (m, 3H, Ar$_H$), 4.08 – 3.93 (m, 1H, ClC*H*), 2.95 – 2.70 (m, 2H, Ar–CH_2), 2.10 – 1.97 (m, 2H, CH_2), 1.56 (d, *J* = 6.5 Hz, 3H, CH_3). **^{13}C NMR** (76 MHz, $CDCl_3$) δ 141.2, 128.7, 128.6, 126.2, 58.1, 42.0, 33.0, 25.6.

The NMR data are in accordance with literature.[5]

3-Chloro-3-methylbutyl benzoate (7b)

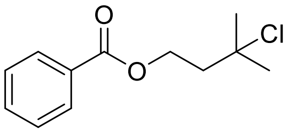

7b was prepared according to **GP-Cl-2** using 3-methylbut-3-en-1-yl benzoate (38 µL, 0.20 mmol, 1.0 eq.) as the starting material. Flash chromatography (pentane/DCM 6:4) gave **7b** as a clear liquid (33.5 mg, 0.148 mmol, 74%).

^{1}H NMR (300 MHz, $CDCl_3$) δ 8.07 – 8.00 (m, 2H, Ar$_H$), 7.61 – 7.52 (m, 1H, Ar$_H$), 7.49 – 7.39 (m, 2H, Ar$_H$), 4.57 (t, *J* = 6.8 Hz, 2H, OCH_2), 2.26 (t, *J* = 6.8 Hz, 2H, CH_2), 1.68 (s, 6H, ClC(CH_3)$_2$). **^{13}C NMR** (76 MHz, $CDCl_3$) δ 166.6, 133.2, 130.3, 129.7 (2xCH), 128.6 (2xCH), 68.7, 62.2, 44.2, 33.1 (2xCH_3).

The NMR data are in accordance with literature.[5]

1-Bromo-10-chloroundecane (7c)

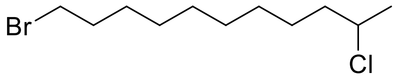

7c was prepared according to **GP-Cl-1** using 11-bromoundec-1-ene (75 µL, 0.50 mmol, 1.0 eq.) as the starting material. Flash chromatography (pentane) gave **7c** as a clear liquid (106.7 mg, 0.3957 mmol, 79%).

^{1}H NMR (300 MHz, $CDCl_3$) δ 4.10 – 3.94 (m, 1H, ClC*H*), 3.41 (t, *J* = 6.8 Hz, 2H, BrCH_2), 1.91 – 1.78 (m, 2H, CH_2), 1.77 – 1.62 (m, 2H, CH_2), 1.50 (d, *J* = 6.5 Hz, 3H, C), 1.48 – 1.35 (m, 4H, 2xCH_2), 1.33 – 1.23 (m, 8H, 4xCH_2). **^{13}C NMR** (76 MHz, $CDCl_3$) δ 59.1, 40.5, 34.2, 33.0, 29.5, 29.5, 29.2, 28.9, 28.3, 26.8, 25.5. **FTIR** (neat): *v*/cm^{-1} 2925*s*, 2854*s*, 1457*m*, 1443*w*, 1379*w*, 1266*m*, 1250*m*, 722*m*, 668*s*, 645*m*, 611*m*, 561*m*. **HRMS** (EI): no undecomposed product detected, calculated for $C_{11}H_{21}Br$ (M-HCl): 232.08266; found: 232.08201, calculated for $C_5H_{10}Cl$ (M-$C_6H_{11}Br$): 105.04710; found: 105.04657.

4-(2-Chloropropyl)-2-methoxyphenol (7d)

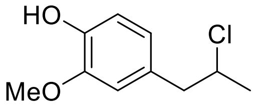

7d was prepared according to **GP-Cl-1** using eugenol (77 µL, 0.50 mmol, 1.0 eq.) as the starting material. Flash chromatography (pentane/Et_2O 9:1) gave **7d** as a clear liquid (74.5 mg, 0.371 mmol, 74%).

^{1}H NMR (300 MHz, $CDCl_3$) δ 6.90 – 6.82 (m, 1H, Ar$_H$), 6.74 – 6.65 (m, 2H, Ar$_H$), 5.53 (s br, 1H, O*H*), 4.18 (h, *J* = 6.6 Hz, 1H, ClC*H*), 3.89 (d, *J* = 0.8 Hz, 3H, OCH_3), 3.02 (dd, *J* = 13.9, 6.9 Hz, 1H, C*H*H), 2.88 (dd, *J* = 13.9, 6.8 Hz, 1H, CH*H*), 1.50 (d, *J* = 6.5 Hz, 3H, CH_3). **^{13}C NMR** (76 MHz, $CDCl_3$) δ 146.5, 144.6, 130.1, 122.2, 114.4, 112.0, 59.0, 56.0, 46.5, 24.7. **FTIR** (neat): *ν*/cm^{-1} 3509*w*, 2972*w*, 2926*w*, 1617*w*, 1512*s*, 1452*m*, 1430*m*, 1375*m*, 1267*s*, 1234*s*, 1207*m*, 1152*m*, 1124*m*, 1066*w*, 1032*m*, 1011*w*, 937*w*, 902*w*, 875*w*, 818*m*, 795*m*, 743*w*, 676*w*, 636*m*, 607*m*, 560*m*. **HRMS** (ESI): calculated for $[(C_{10}H_{12}O_2Cl)_2+Na^+]$: 421.09439; found: 421.09435.

N-(3-Chlorobutyl)-4-methylbenzenesulfonamide (7e)

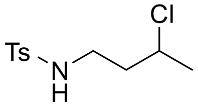

7e was prepared according to **GP-Cl-1** using *N*-(but-3-en-1-yl)-4-methylbenzenesulfonamide (112.7 mg, 0.5000 mmol, 1.0 eq.) as the starting material. Flash chromatography (pentane/EtOAc 90:10→85:15) gave **7e** as a clear oil (120.0 mg, 0.4584 mmol, 92%).

^{1}H NMR (300 MHz, $CDCl_3$) δ 7.75 (d, *J* = 8.3 Hz, 2H, Ar$_H$), 7.32 (d, *J* = 8.3 Hz, 2H, Ar$_H$), 4.85 (s br, 1H, N*H*), 4.07 (dqd, *J* = 10.2, 6.6, 3.7 Hz, 1H, ClC*H*), 3.17 – 3.04 (m br, 2H, NCH_2), 2.43 (s, 3H, Ar–CH_3), 2.02 – 1.89 (m, 1H, C*H*H), 1.86 – 1.71 (m, 1H, CH*H*), 1.47 (d, *J* = 6.6 Hz, 3H, CH_3). **^{13}C NMR** (76 MHz, $CDCl_3$) δ 143.7, 136.8, 129.9 (2xCH), 127.2 (2xCH), 55.7, 40.8, 39.9, 25.5, 21.7. **FTIR** (neat): *ν*/cm^{-1} 3295*m*, 2928*w*, 2870*w*, 1448*w*, 1412*w*, 1324*s*, 1306*m*, 1301*m*, 1266*m*, 1152*s*, 1091*s*, 1062*m*, 1018*m*, 1005*m*, 914*w*, 859*w*, 813*s*, 761*m*, 705*w*, 661*s*, 597*m*, 569*w*. **HRMS** (ESI): calculated for $[C_{11}H_{16}NO_2SCl+Na^+]$: 284.04825; found: 284.04829.

1-(4-((4-Chloropentyl)oxy)phenyl)ethan-1-one (7h)

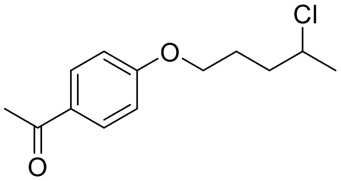

7h was prepared according to **GP-Cl-1** using 1-(4-pent-4-enoxyphenyl)ethanone (102.1 mg, 0.5000 mmol, 1.0 eq.) as the starting material. Flash chromatography (pentane/EtOAc 9:1) gave **7h** as a clear liquid (93.3 mg, 0.388 mmol, 78%).

^{1}H NMR (300 MHz, $CDCl_3$) δ 7.97 – 7.86 (m, 2H, Ar$_H$), 6.95 – 6.86 (m, 2H, Ar$_H$), 4.16 – 3.98 (m, 3H, CCl*H* + OCH_2 overlapped), 2.55 (s, 3H, C(=O)CH_3), 2.13 – 1.77 (m, 4H, 2xCH_2), 1.55 (dd, *J* = 6.6, 1.3 Hz, 3H, CH_3). **^{13}C NMR** (76 MHz, $CDCl_3$) δ 196.9, 162.9, 130.7 (2xCH), 130.4, 114.2 (2xCH), 67.5, 58.4, 36.9, 26.5 (2xCH_2), 25.6. **FTIR** (neat): *ν*/cm^{-1} 2957*w*, 2925*w*, 2872*w*, 2360*w*, 2338*w*, 1673*s*, 1599*s*, 1576*m*, 1559*w*, 1508*m*, 1472*w*, 1418*w*, 1358*m*, 1306*w*, 1249*s*, 1170*s*, 1143*w*, 1116*w*, 1052*w*, 1020*m*, 985*w*, 956*m*, 832*s*, 761*w*, 670*w*, 632*w*, 588*s*, 502*w*. **HRMS** (ESI): calculated for $[C_{13}H_{17}O_2Cl+Na^+]$: 263.08093; found: 263.08093.

Chlorocyclododecane (7i)

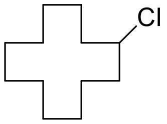

7i was prepared according to **GP-Cl-1** using cyclododecene (96 µg, 0.50 mmol, 1.0 eq.) as the starting material. Flash chromatography (pentane) gave **7i** as a clear liquid (41.1 mg, 0.203 mmol, 41%).

^{1}H NMR (300 MHz, $CDCl_3$) δ 4.12 (tt, *J* = 7.3, 5.3 Hz, 1H, CCl*H*), 2.01 – 1.85 (m, 2H, 2xCClCH*H*), 1.81 – 1.67 (m, 2H, 2xCClC*H*H), 1.60 – 1.48 (m, 2H, Alkyl-*H*), 1.48 – 1.17 (m, 16H, Alkyl-*H*). **^{13}C NMR** (76 MHz, $CDCl_3$) δ 60.4, 33.9 (2xCH_2), 23.8(4) (2xCH_2), 23.7(8), 23.5(2) (2xCH_2), 23.4(7) (2xCH_2), 22.0 (2xCH_2).

The NMR data are in accordance with literature.[34]

9-Chlorotricosane (7j) / 10-Chlorotricosane (7j')

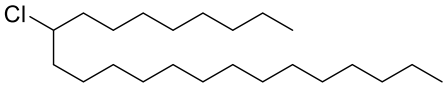

7j

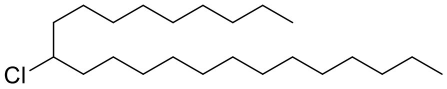

7j'

7j/**7j'** was prepared according to **GP-Cl-1** using (*Z*)-tricos-9-ene (120 µL, 0.300 mmol, 1.0 eq.) as the starting material. Flash chromatography (pentane) gave **7j** and **7j'** as inseparable 1:1 mixture as a clear liquid (44.0 mg, 0.123 mmol, 41%).

^{1}H NMR (300 MHz, $CDCl_3$) δ 3.97 – 3.81 (m, 1H, CCl*H*), 1.82 – 1.61 (m, 4H, 2xCClC*H*$_2$), 1.59 – 1.10 (m, 36H, Alkyl-*H*), 0.88 (t, *J* = 6.8 Hz, 6H, 2xC*H*$_3$). **^{13}C NMR** (76 MHz, $CDCl_3$) δ 64.6, 38.7, 32.0(9), 32.0(4), 32.0(2), 29.8(5), 29.8(2), 29.7(4), 29.7(0), 29.6(8), 29.6(4), 29.5(3), 29.4(7), 29.4(1), 29.3, 26.7, 22.9, 22.8, 14.3. **FTIR** (neat): *v*/cm^{-1} 2921*s*, 2852*s*, 2360*m*, 2341*m*, 1734*w*, 1716*w*, 1699*w*, 1684*w*, 1670*w*, 1653*w*, 1635*w*, 1558*w*, 1541*w*, 1521*w*, 1507*w*, 1497*w*, 1489*w*, 1457*w*, 1437*w*, 1418*w*, 1396*w*, 1375*w*, 1362*w*, 988*m*, 721*m*, 668*m*, 613*w*. **HRMS** (EI): no undecomposed product detected, calculated for $C_{23}H_{46}$ (M-HCl): 322.35940; found: 322.35923.

2-(3-Chloro-3-methylbutyl)isoindoline-1,3-dione (7k)

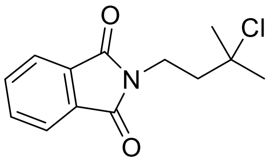

7k was prepared according to **GP-Cl-2** using 2-(3-methylbut-3-en-1-yl)isoindoline-1,3-dione (43.1 mg, 0.200 mmol, 1.0 eq.) as the starting material. Flash chromatography (pentane/EtOAc 95:5→90:10) gave **7k** as a white solid (40.1 mg, 0.159 mmol, 79%).

^{1}H NMR (300 MHz, $CDCl_3$) δ 7.87 – 7.79 (m, 2H, Ar$_H$), 7.75 – 7.66 (m, 2H, Ar$_H$), 3.96 – 3.84 (m, 2H, NC*H*$_2$), 2.18 – 2.06 (m, 2H, C*H*$_2$), 1.65 (s, 6H, C(C*H*$_3$)$_2$). **^{13}C NMR** (76 MHz, $CDCl_3$) δ 168.3 (2xC=O), 134.1 (2xCH), 132.2 (2xC_q), 123.3 (2xCH), 68.2, 43.4, 34.7, 32.6 (2xCH_3).

The NMR data are in accordance with literature.[29]

tert-Butyl 4-chloro-4-methylpiperidine-1-carboxylate (7l)

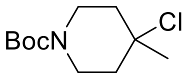

7l was prepared according to **GP-Cl-2** using *tert*-butyl 4-methylenepiperidine-1-carboxylate (41 µL, 0.20 mmol, 1.0 eq.) as the starting material. Flash chromatography (pentane/Et_2O 95:5) gave **7l** as a clear oil (37.4 mg, 0.160 mmol, 80%).

^{1}H NMR (300 MHz, $CDCl_3$) δ 3.93 (s br, 2H, 2xC*H*H), 3.15 (t br, *J* = 11.8 Hz, 2H, 2xCH*H*), 1.92 – 1.79 (m, 2H, 2xC*H*H), 1.73 – 1.56 (m, 2H, 2xCH*H*), 1.63 (s, 3H, CH_3), 1.45 (s, 9H, $C(CH_3)_3$). **^{13}C NMR** (76 MHz, $CDCl_3$) δ 154.8, 79.8, 69.6, 40.5 (br, $4xCH_2$), 33.4, 28.6 ($3xCH_3$). **FTIR** (neat): ν/cm^{-1} 2974*m*, 2927*m*, 2872*w*, 2360*m*, 2342*w*, 1692*s*, 1654*w*, 1558*w*, 1541*w*, 1507*w*, 1474*w*, 1456*w*, 1417*s*, 1364*m*, 1282*m*, 1248*m*, 1233*s*, 1173*s*, 1139*s*, 1080*w*, 990*m*, 966*m*, 864*m*, 832*w*, 787*m*, 768*m*, 584*w*, 549*m*. **HRMS** (ESI): calculated for [$C_{11}H_{20}NO_2Cl+Na^+$]: 256.10748; found: 256.10739.

7-Chloro-3,7-dimethyloctan-1-ol (7m)

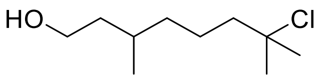

7m was prepared according to **GP-Cl-2** using *β*-Citronellol (36 µL, 0.20 mmol, 1.0 eq.) as the starting material. Flash chromatography (pentane/Et_2O 9:1) gave **7m** as a clear liquid (28.9 mg, 0.150 mmol, 75%).

^{1}H NMR (300 MHz, $CDCl_3$) δ 3.76 – 3.61 (m, 2H, OCH_2), 1.75 – 1.60 (m, 3H, Alkyl-C*H*), 1.57 (s, 6H, $C(CH_3)_2$), 1.62 – 1.08 (m, 6H, Alkyl-C*H*), 0.92 (d, *J* = 6.6 Hz, 3H, CH_3). **^{13}C NMR** (76 MHz, $CDCl_3$) δ 71.4, 61.3, 46.4, 40.1, 37.2, 32.6(1), 32.5(7), 29.5, 22.6, 19.7.

The NMR data are in accordance with literature.[35]

(4-Chloro-4-methylcyclohexyl)benzene (7n)

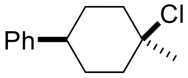

major

7n was prepared according to **GP-Cl-2** using (4-methylenecyclohexyl)benzene (37 µL, 0.20 mmol, 1.0 eq.) as the starting material. Flash chromatography (pentane) gave **7n** as a clear liquid (27.2 mg, 0.130 mmol, 65%, mixture of diastereoisomers, *dr* = 12:1).

Diastereomeric ratio was determined by GC analysis of the crude reaction mixture. The major isomer was assigned by comparison with **8n**.

^{1}H NMR (300 MHz, $CDCl_3$) δ 7.37 – 7.18 (m, 5H, Ar_H), 2.48 (tt, *J* = 12.1, 3.6 Hz, 1H, Ph–CH), 2.17 – 2.05 (m, 2H, 2xC*H*H), 2.05 – 1.95 (m, 2H, 2xCH*H*), 1.86 – 1.61 (m, 4H, $2xCH_2$), 1.69 (s, 3H, CH_3). **^{13}C NMR** (76 MHz, $CDCl_3$) δ 146.9, 128.5 (2xCH), 127.0 (2xCH), 126.3, 71.8, 43.6, 41.7 ($2xCH_2$), 34.4, 30.0 ($2xCH_2$).

The NMR data are in accordance with literature.[5]

2-(4-Chloro-4-methylcyclohexyl)propan-2-ol (7o)

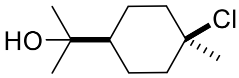
major

7o was prepared according to **GP-Cl-2** using *α*-terpineol (30.9 mg, 0.200 mmol, 1.0 eq.) as the starting material. Flash chromatography (pentane/EtOAc 9:1→85:15) gave **7o** as a white solid (18.8 mg, 98.6 µmol, 49%, mixture of diastereoisomers, *dr* = 12:1).

Diastereomeric ratio was determined by GC analysis of the crude reaction mixture. The major isomer was assigned by comparison with **8n**.

^{1}H NMR (300 MHz, $CDCl_3$) δ 2.12 – 1.97 (m, 2H), 1.77 – 1.66 (m, 2H), 1.66 – 1.58 (m, 1H), 1.61 (s, 3H, C*H*$_3$), 1.58 – 1.40 (m, 4H), 1.34 – 1.22 (m, 2H), 1.20 (s, 6H, C(C*H*$_3$)$_2$). **^{13}C NMR** (76 MHz, $CDCl_3$) δ 72.9, 72.2, 48.3, 41.5, 34.2, 27.2, 23.4. **FTIR** (neat): *v*/cm^{-1} 3387*m*, 2966*s*, 2937*s*, 2868*m*, 2839*w*, 1441*m*, 1380*m*, 1366*m*, 1307*w*, 1192*m*, 1152*s*, 1129*m*, 1101*w*, 1086*w*, 1021*w*, 984*m*, 950*w*, 910*s*, 853*s*, 792*m*, 759*m*, 734*w*, 599*w*, 550*s*, 469*w*. **HRMS** (ESI): calculated for [$C_{10}H_{19}OCl+Na^+$]: 213.10166; found: 213.10161. **M.p.**: 54.9-56.3 °C.

(5*R*)-5-Chloro-cholesteryl acetate (7q)

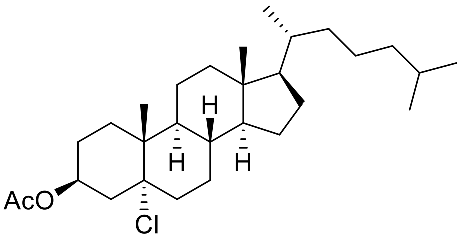

7q was prepared according to **GP-Cl-2** using cholesteryl acetate (85.7 mg, 0.200 mmol, 1.0 eq.) as the starting material. Flash chromatography (pentane/EtOAc 9:1→85:15) gave **7q** as a white solid (54.0 mg, 0.116 mmol, 58%, single diastereoisomers, *dr* > 20:1).

Diastereomeric ratio was determined by GC analysis of the crude reaction mixture. The major isomer was assigned by comparison with **8q**.

^{1}H NMR (300 MHz, $CDCl_3$) δ 5.37 (tt, *J* = 10.9, 5.6 Hz, 1H, OC*H*), 2.12 (ddd, *J* = 13.5, 5.4, 1.8 Hz, 1H), 2.02 (s, 3H), 2.00 – 1.73 (m, 5H), 1.73 – 1.10 (m, 21H), 1.08 (s, 3H, OCC*H*$_3$), 1.07 – 0.93 (m, 3H), 0.89 (d, *J* = 6.5 Hz, 3H), 0.86 (dd, *J* = 6.7, 1.5 Hz, 6H), 0.64 (s, 3H). **^{13}C NMR** (76 MHz, $CDCl_3$) δ 170.5, 84.5, 70.7, 56.2, 56.0, 46.3, 42.8, 41.5, 40.7, 39.9, 39.6, 36.5, 36.3, 35.9, 34.8, 31.6, 28.4, 28.2, 26.6, 26.4, 24.2, 24.0, 23.0, 22.7, 21.5, 18.8, 16.3, 12.3. **FTIR** (neat): *v*/cm^{-1} 2932*s*, 2867*m*, 2846*m*, 2360*m*, 2341*w*, 1728*s*, 1699*w*, 1653*w*, 1558*w*, 1541*w*, 1507*w*, 1466*m*, 1457*m*, 1376*m*, 1243*s*, 1166*w*, 1135*w*, 1093*w*, 1029*s*, 959*w*, 913*w*, 796*w*, 757*w*, 732*w*, 670*w*, 609*w*, 591*w*, 569*m*. **HRMS** (ESI): calculated for [$C_{29}H_{49}O_2Cl+Na^+$]: 487.33133; found: 487.33138. **M.p.**: 144.7-145.9 °C.

(3-Azidobutyl)benzene (8a)

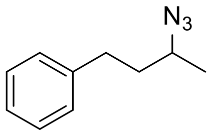

8a was prepared according to **GP-N3** using but-3-en-1-ylbenzene (30 µL, 0.20 mmol, 1.0 eq.) as the starting material. Flash chromatography (pentane/DCM 99:1→95:5) gave **8a** as a clear liquid (16.8 mg, 95.9 µmol, 48%).

^{1}H NMR (300 MHz, $CDCl_3$) δ 7.35 – 7.27 (m, 2H, Ar*H*), 7.25 – 7.14 (m, 3H, Ar*H*), 3.52 – 3.37 (m, 1H, NC*H*), 2.83 – 2.58 (m, 2H, Ph–CH_2), 1.92 – 1.68 (m, 2H, CH_2), 1.30 (d, *J* = 6.5 Hz, 3H, CH_3). **^{13}C NMR** (76 MHz, $CDCl_3$) δ 141.4, 128.6(2) (2xCH), 128.5(6) (2xCH), 126.2, 57.3, 38.0, 32.5, 19.7.

The NMR data are in accordance with literature.[1]

3-Azido-3-methylbutyl benzoate (8b)

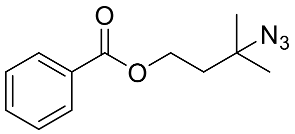

8b was prepared according to **GP-N3** using 3-methylbut-3-en-1-yl benzoate (38 µL, 0.20 mmol, 1.0 eq.) as the starting material. Flash chromatography (pentane/DCM 5:5) gave **8b** as a clear liquid (34.2 mg, 0.147 mmol, 74%).

^{1}H NMR (300 MHz, $CDCl_3$) δ 8.12 – 7.96 (m, 2H, Ar*H*), 7.62 – 7.50 (m, 1H, Ar*H*), 7.48 – 7.39 (m, 2H, Ar*H*), 4.44 (t, *J* = 6.8 Hz, 2H, OCH_2), 1.98 (t, *J* = 6.8 Hz, 2H, CH_2), 1.38 (s, 6H, C(CH_3)$_2$). **^{13}C NMR** (76 MHz, $CDCl_3$) δ 166.6, 133.1, 130.3, 129.7 (2xCH), 128.5 (2xCH), 61.4, 60.4, 39.9, 26.5 (2xCH)$_3$.

The NMR data are in accordance with literature.[29]

10-Azido-1-bromoundecane (8c)

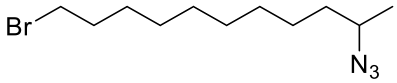

8c was prepared according to **GP-N3** using 11-bromoundec-1-ene (44 µL, 0.20 mmol, 1.0 eq.) as the starting material. Flash chromatography (pentane/Et_2O 100:0→99:1) gave **8c** as a clear liquid (30.3 mg, 0.110 mmol, 55%).

^{1}H NMR (300 MHz, $CDCl_3$) δ 3.40 (t, *J* = 6.9 Hz, 2H, BrCH_2), 1.91 – 1.79 (m, 2H, CH_2), 1.56 – 1.26 (m, 15H, Alkyl-C*H*), 1.24 (d, *J* = 6.5 Hz, 3H, CH_3). **^{13}C NMR** (76 MHz, $CDCl_3$) δ 58.2, 36.3, 34.1, 33.0, 29.5(1), 29.4(5) (2 signals overlapped), 28.9, 28.3, 26.2, 19.6. **FTIR** (neat): *v*/cm^{-1} 2926s, 2854m, 2095s, 1457m, 1379w, 1327w, 1247m, 1123w, 1041w, 912w, 723m, 646m, 562m. **HRMS** (ESI): calculated for [$C_{11}H_{22}N_3Br+Ag^+$]: 384.00307; found: 384.00294.

4-(2-Azidopropyl)-2-methoxyphenol (8d)

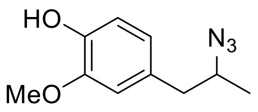

8d was prepared according to **GP-N3** using eugenol (31 µL, 0.20 mmol, 1.0 eq.) as the starting material. Flash chromatography (pentane/DCM 1:1) gave **8d** as a clear liquid (18.4 mg, 88.8 µmol, 44%).

^{1}H NMR (300 MHz, $CDCl_3$) δ 6.91 – 6.82 (m, 1H, Ar$_{H}$), 6.75 – 6.66 (m, 2H, Ar$_{H}$), 5.55 (s, 1H, O*H*), 3.89 (s, 3H, OCH_3), 3.71 – 3.56 (m, 1H, NC*H*), 2.81 – 2.56 (m, 2H, CH_2), 1.26 (d, *J* = 6.5 Hz, 3H, CH_3). **^{13}C NMR** (76 MHz, $CDCl_3$) δ 146.4, 144.4, 129.7, 122.0, 114.4, 111.7, 59.3, 55.9, 42.3, 19.1.

The NMR data are in accordance with literature.[36]

***N*-(3-Azidobutyl)-4-methylbenzenesulfonamide (8e)**

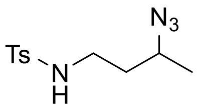

8e was prepared according to **GP-N3** using *N*-(but-3-en-1-yl)-4-methylbenzenesulfonamide (45.1 mg, 0.200 mmol, 1.0 eq.) as the starting material. Flash chromatography (pentane/EtOAc 9:1→8:2) gave **8e** as a clear oil (21.0 mg, 78.3 µmol, 39%).

^{1}H NMR (300 MHz, $CDCl_3$) δ 7.79 – 7.69 (m, 2H, Ar$_{H}$), 7.35 – 7.28 (m, 2H, Ar$_{H}$), 4.89 (s br, 1H, N*H*), 3.54 (dqd, *J* = 9.0, 6.5, 4.4 Hz, 1H, N_3C*H*), 3.09 – 2.95 (m, 2H, NCH_2), 2.43 (s, 3H, Ar–CH_3), 1.71 – 1.47 (m, 2H, CH_2), 1.24 (d, *J* = 6.6 Hz, 3H, CH_3). **^{13}C NMR** (76 MHz, $CDCl_3$) δ 143.7, 136.8, 129.9 (2xCH), 127.2 (2xCH), 55.6, 40.4, 35.9, 21.7, 19.4. **FTIR** (neat): *v*/cm^{-1} 3278m, 2974w, 2926w, 2870w, 2096s, 1419m, 1380w, 1321m, 1305m, 1244m, 1154s, 1091s, 1019w, 980w, 872w, 813m, 760w, 707w, 660s, 569m. **HRMS** (ESI): calculated for [$C_{11}H_{16}N_4O_2S$+Na^{+}]: 291.08862; found: 291.08878.

((4-Azidopentyl)oxy)(*tert*-butyl)dimethylsilane (8f)

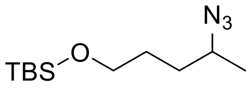

8f was prepared according to **GP-N3** using *tert*-butyldimethyl(pent-4-en-1-yloxy)silane (40.1 mg, 0.200 mmol, 1.0 eq.) as the starting material. Flash chromatography (pentane/Et_2O 99:1→98:2) gave **8f** as a clear liquid (19.7 mg, 80.9 µmol, 40%).

^{1}H NMR (300 MHz, $CDCl_3$) δ 3.68 – 3.57 (m, 2H, OCH_2), 3.53 – 3.38 (m, 1H, N*H*), 1.65 – 1.48 (m, 4H, 2xCH_2), 1.26 (d, *J* = 6.5 Hz, 3H, CH_3), 0.89 (s, 9H, C(CH_3)$_3$), 0.05 (s, 6H, Si(CH_3)$_2$). **^{13}C NMR** (76 MHz, $CDCl_3$) δ 62.8, 58.0, 32.8, 29.4, 26.1 (3xCH_3), 19.7, 18.5, -5.2 (2xCH_3). **^{29}Si NMR** (60 MHz, $CDCl_3$) δ 18.90. **FTIR** (neat): *v*/cm^{-1} 2972*w*, 2929*s*, 2857*m*, 2360*w*, 2094*s*, 1462*w*, 1410*w*, 1380*w*, 1327*w*, 1248*s*, 1129*w*, 1011*w*, 913*m*, 834*m*, 726*w*, 652*w*, 560*w*. **HRMS** (EI): calculated for $C_7H_{16}N_3OSi$ (M-tBu): 186.10572; found: 186.10574.

2-(7-azidooctyl)oxirane (8g)

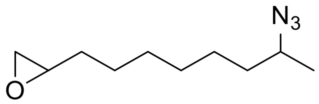

8g was prepared according to **GP-N3** using 2-(oct-7-en-1-yl)oxirane (37 µL, 0.20 mmol, 1.0 eq.) as the starting material. Flash chromatography (pentane/Et_2O 96:4) gave **8g** as a clear liquid (16.3 mg, 94.9 µmol, 47%, 1:1 mixture of diastereoisomers).

^{1}H NMR (300 MHz, $CDCl_3$) δ 3.49 – 3.34 (m, 1H, NC*H*), 2.90 (tdd, *J* = 5.0, 4.0, 2.7 Hz, 1H, OC*H*), 2.74 (dd, *J* = 5.1, 4.0 Hz, 1H, OC*H*H), 2.46 (dd, *J* = 5.1, 2.7 Hz, 1H, OCH*H*), 1.61 – 1.29 (m, 12H, Alkyl-*H*), 1.24 (d, *J* = 6.5 Hz, 3H, C*H*$_3$). **^{13}C NMR** (76 MHz, $CDCl_3$) δ 58.1, 52.5, 47.2, 36.3, 32.6, 29.4, 29.4, 26.1, 26.0, 19.6. **FTIR** (neat): *v*/cm^{-1} 2953*m*, 2929*m*, 2857*m*, 2360*m*, 2341*w*, 2098*s*, 1472*m*, 1386*w*, 1362*w*, 1254*s*, 1098*s*, 1038*w*, 10005*m*, 938*w*, 835*s*, 811*w*, 775*s*, 714*w*, 661*m*. **HRMS** (ESI): calculated for [$C_{10}H_{19}N_3O+Na^+$]: 220.14203; found: 220.14192.

1-(4-((4-Azidopentyl)oxy)phenyl)ethan-1-one (8h)

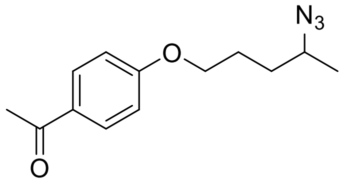

8h was prepared according to **GP-N3** using 1-(4-pent-4-enoxyphenyl)ethanone (40.9 mg, 0.200 mmol, 1.0 eq.) as the starting material. Flash chromatography (pentane/DCM 5:5→3:7) gave **8h** as a clear liquid (26.8 mg, 0.108 mmol, 54%).

^{1}H NMR (300 MHz, $CDCl_3$) δ 7.98 – 7.86 (m, 2H, Ar$_H$), 6.97 – 6.85 (m, 2H, Ar$_H$), 4.03 (td, *J* = 6.0, 1.8 Hz, 2H, OC*H*$_2$), 3.60 – 3.45 (m, 1H, CN_3*H*), 2.55 (s, 3H, C(=O)C*H*$_3$), 2.05 – 1.77 (m, 2H, C*H*$_2$), 1.77 – 1.54 (m, 2H, C*H*$_2$), 1.30 (d, *J* = 6.5 Hz, 3H, C*H*$_3$). **^{13}C NMR** (76 MHz, $CDCl_3$) δ 196.9, 162.9, 130.7 (2xCH_2), 130.4, 114.2 (2xCH_2), 67.7, 57.8, 32.9, 26.5, 25.9, 19.6. **FTIR** (neat): *v*/cm^{-1} 2928*w*, 2874*w*, 1362*w*, 2096*s*, 1674*s*, 1599*s*, 1576*m*, 1508*m*, 1471*w*, 1419*w*, 1358*m*, 1306*w*, 1248*s*, 1170*s*, 1115*w*, 1059*w*, 1020*m*, 993*m*, 956*m*, 918*w*, 832*s*, 808*w*, 732*m*, 632*w*, 589*s*, 502*w*. **HRMS** (ESI): calculated for [$C_{13}H_{17}N_3O_2Na^+$]: 270.12130; found: 270.12109.

Azidocyclododecane (8i)

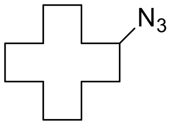

8i was prepared according to **GP-N3** using cyclododecene (39 µL, 0.20 mmol, 1.0 eq.) as the starting material. Flash chromatography (pentane) gave **8i** as a clear liquid (19.9 mg, 95.1 µmol, 48%).

^{1}H NMR (300 MHz, $CDCl_3$) δ 3.48 (tt, *J* = 7.5, 4.7 Hz, 1H, CN_3H), 1.82 – 1.59 (m, 3H, Alkyl-*H*), 1.58 – 1.19 (m, 19H, Alkyl-*H*). **^{13}C NMR** (76 MHz, $CDCl_3$) δ 59.3, 29.2 (2xCH_2), 24.1 (2xCH_2), 23.8, 23.5 (2xCH_2), 23.4 (2xCH_2), 21.4 (2xCH_2).

The NMR data are in accordance with literature.[37]

9-Azidotricosane (8j) / 10-Azidotricosane (8j')

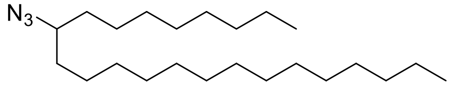

8j

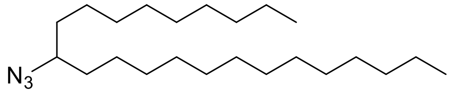

8j'

8j/8j' was prepared according to **GP-N3** using (*Z*)-tricos-9-ene (80 µL, 0.20 mmol, 1.0 eq.) as the starting material. Flash chromatography (pentane) gave **8j** and **8j'** as 1:1 mixture as a clear liquid (35.0 mg, 95.7 µmol, 48%).

^{1}H NMR (300 MHz, $CDCl_3$) δ 3.34 – 3.10 (m, 1H, CN_3H), 1.59 – 1.11 (m, 40H, Alkyl-*H*), 0.88 (t, *J* = 6.8 Hz, 6H, 2xCH_3). **^{13}C NMR** (76 MHz, $CDCl_3$) δ 63.3, 34.6, 32.0(9), 32.0(4), 32.0(1), 29.8(5), 29.8(2), 29.8(0), 29.7(3), 29.6(8), 29.6(4), 29.6(1), 29.5(3), 29.4(6), 29.3(9), 26.2(9), 22.8(6), 22.8(3), 22.8(2), 14.3.**FTIR** (neat): v/cm^{-1} 2922*s*, 2852*s*, 2360*m*, 2341*m*, 2095*s*, 1734*w*, 1716*w*, 1699*w*, 1684*w*, 1653*w*, 1635*w*, 1558*w*, 1541*w*, 1521*w*, 1507*w*, 1489*w*, 1457*w*, 1437*w*, 1418*w*, 1375*w*, 1339*w*, 1273*m*, 1252*m*, 985*m*, 721*m*, 668*w*. **HRMS** (ESI): calculated for [$C_{23}H_{47}N_3$+Ag^+]: 472.28154; found: 472.28166.

2-(3-Azido-3-methylbutyl)isoindoline-1,3-dione (8k)

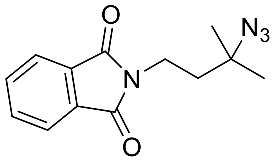

8k was prepared according to **GP-N3** using 2-(3-methylbut-3-en-1-yl)isoindoline-1,3-dione (43.1 mg, 0.200 mmol, 1.0 eq.) as the starting material. Flash chromatography (pentane/DCM 4:6) gave **8k** as a clear oil (36.1 mg, 0.140 mmol, 70%).

^{1}H NMR (300 MHz, $CDCl_3$) δ 7.88 – 7.78 (m, 2H, Ar_H), 7.74 – 7.65 (m, 2H, Ar_H), 3.84 – 3.72 (m, 2H, NCH_2), 1.89 – 1.80 (m, 2H, CH_2), 1.36 (s, 6H, $C(CH_3)_2$). **^{13}C NMR** (75 MHz, CDCl3) δ 168.3 (2xCO), 134.1 (2xCH), 132.2 (2xC), 123.3 (2xCH), 60.3, 39.3, 33.9, 26.0 (2xCH3).

The NMR data are in accordance with literature.[29]

***tert*-Butyl 4-azido-4-methylpiperidine-1-carboxylate (8l)**

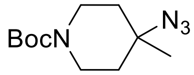

8l was prepared according to **GP-N3** using *tert*-butyl 4-methylenepiperidine-1-carboxylate (41 µL, 0.20 mmol, 1.0 eq.) as the starting material. Flash chromatography (pentane/Et_2O 9:1) gave **7l** as a clear oil (34.9 mg, 0.145 mmol, 73%).

^{1}H NMR (400 MHz, $CDCl_3$) δ 3.79 (s br, 2H, 2xCH*H*), 3.16 – 2.99 (m, 2H, 2xC*H*H), 1.66 – 1.59 (m, 2H, 2xC*H*H), 1.53 – 1.47 (m, 2H, 2xCH*H*), 1.44 (s, 9H, $C(CH_3)_3$), 1.33 (s, 3H, CH_3). **^{13}C NMR** (100 MHz, $CDCl_3$) δ 154.8, 79.7, 59.9, 40.0 (br, 2xCH_2), 35.9 (2xCH_2), 28.5 (3xCH_3), 26.2. **FTIR** (neat): v/cm^{-1} 2975*m*, 2930*m*, 2871*m*, 2090*s*, 1691*s*, 1478*m*, 1451*m*, 1418*s*, 1364*s*, 1283*m*, 1254*s*, 1238*s*, 1175*s*, 1147*s*, 1131*s*, 1083*m*, 990*m*, 966*m*, 912*m*, 868*m*, 851*m*, 822*m*, 799*w*, 769*m*, 746*m*, 733*m*, 647*w*, 599*m*, 564*w*. **HRMS** (ESI): calculated for [$C_{11}H_{20}N_4O_2$+Na^+]: 263.1479; found: 263.1476.

7-Azido-3,7-dimethyloctan-1-ol (8m)

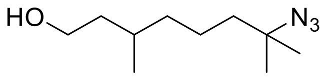

8m was prepared according to **GP-N3** using *β*-citronellol (36 µL, 0.20 mmol, 1.0 eq.) as the starting material. Flash chromatography (pentane/Et_2O 7:3) gave **8m** as a clear liquid (30.0 mg, 0.151 mmol, 75%).

^{1}H NMR (300 MHz, $CDCl_3$) δ 3.81 – 3.51 (m, 2H, OC*H*$_2$), 1.68 – 1.30 (m, 8H, Alkyl-C*H*), 1.24 (s, 6H, C(C*H*$_3$)$_2$), 1.21 – 1.07 (m, 1H, C*H*), 0.90 (d, *J* = 6.7 Hz, 3H, C*H*$_3$). **^{13}C NMR** (76 MHz, $CDCl_3$) δ 61.9, 61.3, 41.8, 40.0, 37.4, 29.5, 26.2, 26.1, 21.7, 19.7.

The NMR data are in accordance with literature.[36]

(4-Azido-4-methylcyclohexyl)benzene (8n)

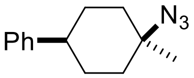

major

8n was prepared according to **GP-N3** using (4-methylenecyclohexyl)benzene (37 µL, 0.20 mmol, 1.0 eq.) as the starting material. Flash chromatography (pentane/Et_2O 100:0→98:2) gave **8n** as a clear liquid (29.9 mg, 0.139 mmol, 69%, only major diastereoisomers could be isolated, *dr* = 9:1).

Diastereomeric ratio was determined by GC analysis of the crude reaction mixture. The major isomer was assigned by X-ray analysis of a single crystal, obtained through the X-ray general procedure described under chapter 3.

^{1}H NMR (300 MHz, $CDCl_3$) δ 7.37 – 7.28 (m, 2H, Ar$_H$), 7.28 – 7.17 (m, 3H, Ar$_H$), 2.55 – 2.40 (m, 1H, Ar–C*H*), 1.95 – 1.84 (m, 2H, 2xC*H*H), 1.85 – 1.70 (m, 4H, 2xC*H*$_2$), 1.58 – 1.44 (m, 2H, 2xC*H*H), 1.38 (s, 3H, C*H*$_3$). **^{13}C NMR** (76 MHz, $CDCl_3$) δ 146.8, 128.5 (2xCH), 127.0 (2xCH), 126.3, 61.1, 43.6, 37.0 (2xCH_2), 29.8 (2xCH_2), 27.5. **FTIR** (neat): *ν*/cm^{-1} 3027w, 2963w, 2927m, 2859w, 2110m, 2089s, 1738m, 1602w, 1494w, 1447m, 1379w, 1258m, 1238w, 1126m, 1032w, 1009w, 962w, 929w, 849w, 755m, 699s, 599w, 566w. **HRMS** (ESI): calculated for [$C_{13}H_{17}N+H^+$] (M-N_2+H^+): 188.14338; found: 188.14331.

2-(4-Azido-4-methylcyclohexyl)propan-2-ol (8o)

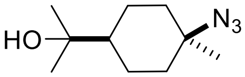

major

8o was prepared according to **GP-N3** using *α*-terpineol (30.9 mg, 0.200 mmol, 1.0 eq.) as the starting material. Flash chromatography (pentane/EtOAc 9:1→8:2) gave **8o** as a clear liquid (18.8 mg, 98.6 µmol, 49%, mixture of diastereoisomers, *dr* = 8:1).

Diastereomeric ratio was determined by GC analysis of the crude reaction mixture. The major isomer was assigned by comparison with **8n**.

^{1}H NMR (300 MHz, $CDCl_3$) δ 1.88 – 1.74 (m, 2H, 2xCH*H*), 1.72 – 1.56 (m, 2H, 2xC*H*H), 1.43 – 1.20 (m, 4H, 2xC*H*$_2$), 1.30 (s, 3H, C*H*$_3$), 1.18 (s, 6H, C(C*H*$_3$)$_2$). **^{13}C NMR** (76 MHz, $CDCl_3$) δ 72.8, 61.3, 48.2, 36.7 (2xCH), 27.4, 27.2 (2xCH_3), 23.1 (2xCH_2).

The NMR data are in accordance with literature.[36]

(3*R*,3a*S*,6*S*,7*R*,8a*S*)-6-Azido-3,6,8,8-tetramethyloctahydro-1H-3a,7-methanoazulene (8p)

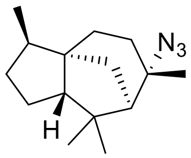

8p was prepared according to **GP-N3** using (–)-*α*-cedrene (44 µL, 0.20 mmol, 1.0 eq.) as the starting material. Flash chromatography (pentane) gave **8p** as a clear liquid (31.5 mg, 0.127 mmol, 64%, single diastereoisomers, *dr* > 20:1).

Diastereomeric ratio was determined by GC analysis of the crude reaction mixture. The major isomer was assigned by X-ray analysis of a single crystal, obtained through the X-ray general procedure described in chapter 3.

^{1}H NMR (300 MHz, $CDCl_3$) δ 1.95 – 1.81 (m, 2H, Alkyl-C*H*), 1.78 – 1.47 (m, 8H, Alkyl-C*H*), 1.44 (s, 3H, CH_3), 1.41 – 1.21 (m, 3H, Alkyl-C*H*), 1.16 (s, 3H, CH_3), 1.03 (s, 3H, CH_3), 0.84 (d, *J* = 7.1 Hz, 3H, CH_3). **^{13}C NMR** (76 MHz, $CDCl_3$) δ 67.2, 58.6, 57.2, 53.5, 42.9, 41.8, 41.1, 37.0, 32.0, 30.8, 29.3, 28.3, 26.3, 25.5, 15.5. **FTIR** (neat): *v*/cm^{-1} 2950s, 2871m,2159w, 2095s, 2031w, 1974w, 1460m, 1379w, 1364w, 1258m, 1246m, 1227w, 1182w, 1147w, 1103w, 1066w, 1045w, 1022w, 995w, 970w, 877w, 822m, 752w, 650w, 626w, 566w. **HRMS** (EI): calculated for $C_{15}H_{25}$ (M-N_3): 205.19563; found: 205.19503.

(5*R*)-5-Azido-cholesteryl acetate (8q)

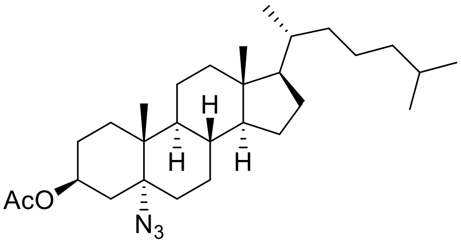

8q was prepared according to **GP-N3** using cholesteryl acetate (85.7 mg, 0.200 mmol, 1.0 eq.) as the starting material. Flash chromatography (pentane/EtOAc 9:1→85:15) gave **8q** as a white solid (54.0 mg, 0.116 mmol, 58%, single diastereoisomer).

dr > 20:1. Diastereomeric ratio was determined by GC analysis of the crude reaction mixture. The major isomer was assigned by X-ray analysis of a single crystal.

^{1}H NMR (300 MHz, $CDCl_3$) δ 5.21 – 4.87 (m, 1H, OC*H*), 2.08 – 1.98 (m, 3H, CH_3), 1.99 – 1.03 (m, 30H, Alkyl-C*H*), 1.00 (s, 3H, CH_3), 0.89 (d, *J* = 6.4 Hz, 3H, CH_3), 0.85 (d, *J* = 6.6 Hz, 6H, 2xCH_3), 0.63 (s, 3H, CH_3). **^{13}C NMR** (76 MHz, $CDCl_3$) δ 170.6, 70.1, 69.8, 56.3, 56.1, 46.0, 42.8, 39.9, 39.6, 38.4, 36.3 (2xCH_2 overlapped), 35.9, 34.7, 30.6, 30.2, 28.4, 28.1, 26.7, 26.5, 24.2, 24.0, 23.0, 22.7, 21.5, 21.3, 18.8, 15.8, 12.2. **FTIR** (neat): *v*/cm^{-1} 2935s, 2866m, 2852m, 2095s, 1737s, 1467m, 1448w, 1376m, 1363w, 1259w, 1237s, 1168w, 1139w, 1025s, 970w, 960w, 932w, 913w, 865w, 805w, 743w, 684w, 607w. **HRMS** (ESI): calculated for [$C_{29}H_{49}N_3O_2$+Na^+]: 494.37170; found: 494.37109. **M.p.**: 138.8-141.2 °C.

(1a*R*,4a*R*,7*R*,7a*S*,7b*S*)-4-Azido-1,1,4,7-tetramethyldecahydro-1H-cyclopropa[e]azulene (8r)

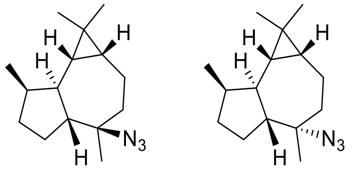

major minor

8r was prepared according to **GP-N3** using (+)-aromadendrene (45 µL, 0.20 mmol, 1.0 eq.) as the starting material. Flash chromatography (pentane) gave **8r** as a clear liquid as two separable diastereoisomers (*dr* = 4:1).

dr = 4:1. Diastereomeric ratio was determined by GC analysis of the crude reaction mixture. The major isomer was assigned by 1D-NOESY experiment and comparison with literature.

Major (**8r-1**): 28.6 mg, 0.116 mmol, 58%, clear liquid.

^{1}H NMR (300 MHz, $CDCl_3$) δ 2.10 – 1.94 (m, 2H), 1.91 – 1.74 (m, 3H), 1.74 – 1.60 (m, 2H), 1.48 – 1.18 (m, 4H), 1.11 (s, 3H), 1.01 (s, 3H), 0.98 (s, 3H), 0.92 (d, *J* = 7.1 Hz, 3H), 0.67 – 0.48 (m, 2H). **^{13}C NMR** (76 MHz, $CDCl_3$) δ 67.0 (C_q), 54.7 (CH), 40.7 (CH_2), 39.5 (CH), 36.6 (CH), 34.7 (CH_2), 28.7 (CH_3), 28.5 (CH), 27.1 (CH_2), 26.8 (CH), 20.2 (C_q), 20.1 (CH_2), 17.2 (CH_3), 16.2 (CH_3), 16.0 (CH_3). **FTIR** (neat): ν/cm^{-1} 2952s, 2925s, 2867m, 2083s, 1456m, 1383m, 1249s, 1181w, 1169w, 1116w, 1090w, 1064w, 983w, 855w, 688w, 670m, 566w, 522w. **HRMS** (EI): calculated for $C_{15}H_{25}N_3$: 247.20430; found: 247.20501.

Minor (**8r-2**): 8.0 mg, 32 µmol, 16%, clear liquid.

^{1}H NMR (300 MHz, $CDCl_3$) δ 2.08 – 1.94 (m, 1H), 1.89 (dd, *J* = 14.2, 6.3 Hz, 1H), 1.79 – 1.60 (m, 5H), 1.53 – 1.44 (m, 1H), 1.43 – 1.22 (m, 2H), 1.33 (s, 3H), 1.14 (ddd, *J* = 11.3, 7.8, 5.7 Hz, 1H), 1.03 (s, 3H), 1.00 (s, 3H), 0.89 (d, *J* = 7.1 Hz, 3H), 0.61 – 0.41 (m, 2H). **^{13}C NMR** (76 MHz, $CDCl_3$) δ 66.4 (C_q), 56.1 (CH), 40.2 (CH_2), 38.6 (CH), 35.8 (CH), 34.4 (CH_2), 28.9 (CH), 28.8 (CH_3), 27.5 (CH_2), 26.9(2) (CH), 26.8(5) (CH_3), 20.9 (C_q), 20.0 (CH_2), 16.6 (CH_3), 16.0 (CH_3).

1.7 X-Ray Data

X-Ray diffraction: Data sets for compounds **9n•HCl**, **9p•HCl** and **8q** were collected with a Bruker D8 Venture Photon III Diffractometer. Programs used: data collection: *APEX4* Version 2021.4-0[38] (Bruker AXS Inc., **2021**); cell refinement: *SAINT* Version 8.40B (Bruker AXS Inc., **2021**); data reduction: *SAINT* Version 8.40B (Bruker AXS Inc., **2021**); absorption correction, *SADABS* Version 2016/2 (Bruker AXS Inc., **2021**); structure solution *SHELXT*-Version 2018-3[39] (Sheldrick, G. M. *Acta Cryst.,* **2015**, *A71*, 3-8); structure refinement *SHELXL*- Version 2018-3[40] (Sheldrick, G. M. *Acta Cryst.,* **2015**, *C71* (1), 3-8) and graphics, *XP*[41] (Version 5.1, Bruker AXS Inc., Madison, Wisconsin, USA, **1998**). *R*-values are given for observed reflections, and wR^2 values are given for all reflections.

X-ray crystal structure analysis of 9n•HCL (stu10605): A colorless, needle-like specimen of $C_{13}H_{20}ClN$, approximate dimensions 0.067 mm x 0.073 mm x 0.213 mm, was used for the X-ray crystallographic analysis. The X-ray intensity data were measured on a single crystal diffractometer Bruker D8 Venture Photon III system equipped with a micro focus tube Cu ImS (CuKα, λ = 1.54178 Å) and a MX mirror monochromator.

A total of 1764 frames were collected. The total exposure time was 17.15 hours. The frames were integrated with the Bruker SAINT software package using a wide-frame algorithm. The integration of the data using a monoclinic unit cell yielded a total of 43200 reflections to a maximum θ angle of 66.96° (0.84 Å resolution), of which 6703 were independent (average redundancy 6.445, completeness = 98.8%, R_{int} = 3.99%, R_{sig} = 3.15%) and 6543 (97.61%) were greater than $2\sigma(F^2)$. The final cell constants of a = 14.0504(4) Å, b = 9.6225(3) Å, c = 14.3171(4) Å, β = 95.0060(10)°, volume = 1928.29(10) Å^3, are based upon the refinement of the XYZ-centroids of 9090 reflections above 20 σ(I) with 6.197° < 2θ < 133.8°. Data were corrected for absorption effects using the multi-scan method (SADABS). The ratio of minimum to maximum apparent transmission was 0.845. The calculated minimum and maximum transmission coefficients (based on crystal size) are 0.6330 and 0.8580.

The structure was solved and refined using the Bruker SHELXTL Software Package, using the space group $P2_1$, with Z = 6 for the formula unit, $C_{13}H_{20}ClN$. The final anisotropic full-matrix least-squares refinement on F^2 with 445 variables converged at R1 = 2.28%, for the observed data and wR2 = 5.46% for all data. The goodness-of-fit was 1.034. The largest peak in the final difference electron density synthesis was 0.145 e^-/Å^3 and the largest hole was -0.136 e^-/Å^3 with an RMS deviation of 0.030 e^-/Å^3. On the basis of the final model, the calculated density was 1.166 g/cm^3 and F(000), 732 e^-.

CCDC number: 2357454.

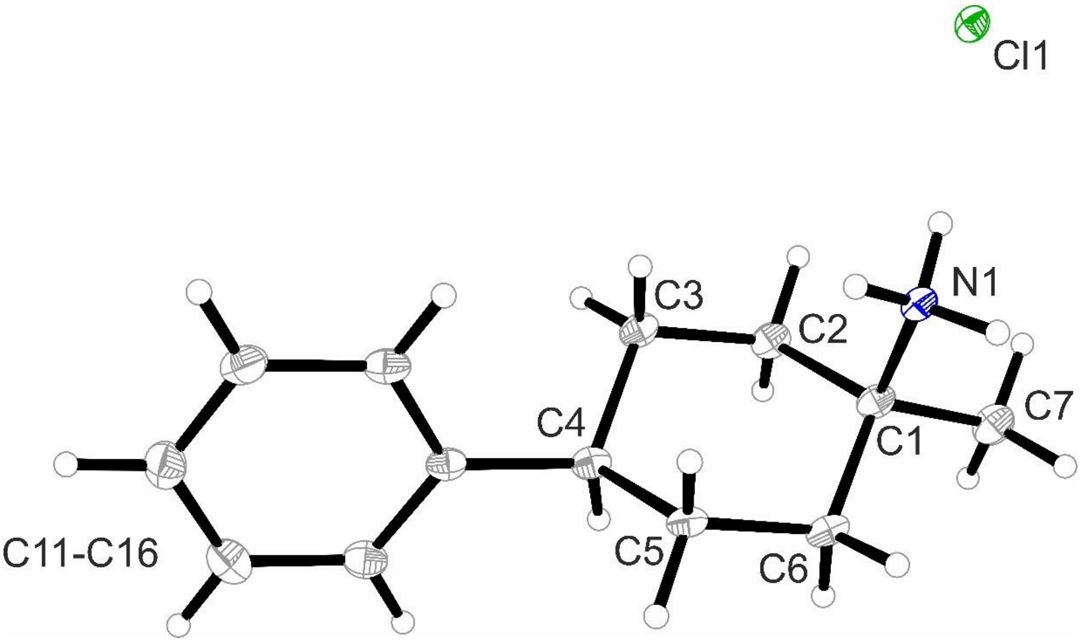

Supplementary Figure 4: Crystal structure of compound **9n•HCl**. Thermal ellipsoids are set at 30% probability. Only one (molecule with suffix A) of three independent molecules found in the asymmetric unit is shown.

X-ray crystal structure analysis of 9p•HCl (stu10608): A colorless, prism-like specimen of $C_{16}H_{29.50}ClN_{1.50}$, approximate dimensions 0.042 mm x 0.042 mm x 0.092 mm, was used for the X-ray crystallographic analysis. The X-ray intensity data were measured on a single crystal diffractometer Bruker D8 Venture Photon III system equipped with a micro focus tube Cu ImS (CuKα, λ = 1.54178 Å) and a MX mirror monochromator.

A total of 1523 frames were collected. The total exposure time was 18.35 hours. The frames were integrated with the Bruker SAINT software package using a wide-frame algorithm. The integration of the data using a monoclinic unit cell yielded a total of 34467 reflections to a maximum θ angle of 66.62° (0.84 Å resolution), of which 5904 were independent (average redundancy 5.838, completeness = 99.9%, R_{int} = 12.47%, R_{sig} = 7.31%) and 4542 (76.93%) were greater than $2\sigma(F^2)$. The final cell constants of a = 22.4999(6) Å, b = 9.5878(2) Å, c = 15.7525(4) Å, β = 99.833(2)°, volume = 3348.28(14) Å^3, are based upon the refinement of the XYZ-centroids of 7331 reflections above 20 σ(I) with 8.976° < 2θ < 132.1°. Data were corrected for absorption effects using the multi-scan method (SADABS). The ratio of minimum to maximum apparent transmission was 0.900. The calculated minimum and maximum transmission coefficients (based on crystal size) are 0.8450 and 0.9240.

The structure was solved and refined using the Bruker SHELXTL Software Package, using the space group *C* 2, with Z = 8 for the formula unit, $C_{16}H_{29.50}ClN_{1.50}$. The final anisotropic full-matrix least-squares refinement on F^2 with 378 variables converged at R1 = 4.82%, for the observed data and wR2 = 11.26% for all data. The goodness-of-fit was 1.028. The largest peak in the final difference electron density synthesis was 0.239 e$^-$/Å^3 and the largest hole was -0.323 e$^-$

/Å^3 with an RMS deviation of 0.049 e$^-$/Å^3. On the basis of the final model, the calculated density was 1.104 g/cm^3 and F(000), 1224 e$^-$.

CCDC number: 2357455.

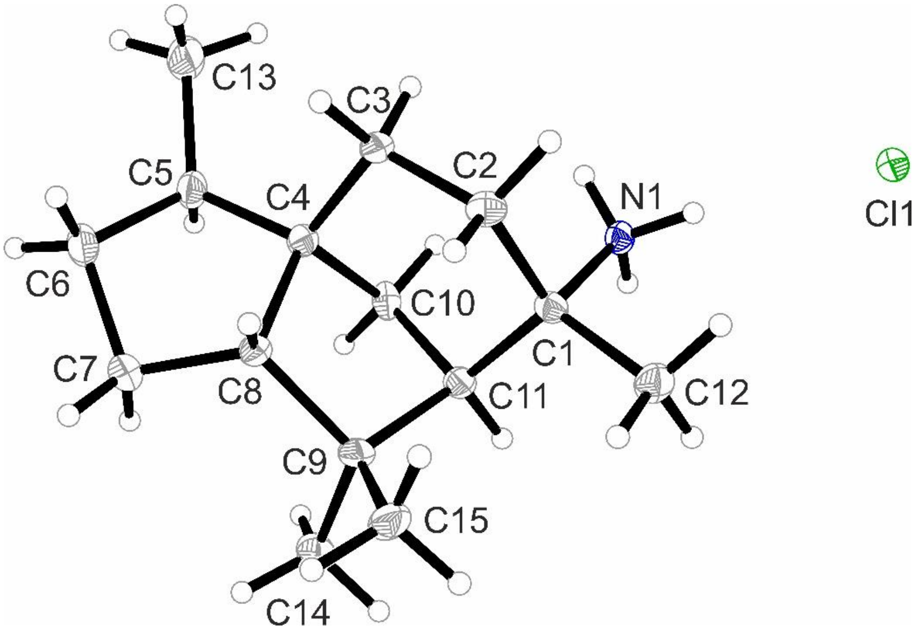

Supplementary Figure 5: Crystal structure of compound **9p•HCl**. Thermal ellipsoids are set at 30% probability. The acetonitrile molecule is omitted for clarity. Only one (molecule with suffix A) of two independent molecules found in the asymmetric unit is shown.

X-ray crystal structure analysis of 8q (stu10589): A colorless, prism-like specimen of $C_{29}H_{49}N_3O_2$, approximate dimensions 0.082 mm x 0.150 mm x 0.166 mm, was used for the X-ray crystallographic analysis. The X-ray intensity data were measured on a single crystal diffractometer Bruker D8 Venture Photon III system equipped with a micro focus tube Cu ImS (CuKα, λ = 1.54178 Å) and a MX mirror monochromator.

A total of 2228 frames were collected. The total exposure time was 23.01 hours. The frames were integrated with the Bruker SAINT software package using a wide-frame algorithm. The integration of the data using a monoclinic unit cell yielded a total of 57741 reflections to a maximum θ angle of 67.06° (0.84 Å resolution), of which 9722 were independent (average redundancy 5.939, completeness = 99.2%, R_{int} = 4.77%, R_{sig} = 3.17%) and 8995 (92.52%) were greater than 2σ(F^2). The final cell constants of a = 18.9781(6) Å, b = 7.5359(2) Å, c = 19.2708(6) Å, β = 91.253(2)°, volume = 2755.39(14) Å^3, are based upon the refinement of the XYZ-centroids of 9936 reflections above 20 σ(I) with 6.609° < 2θ < 133.6°. Data were corrected for absorption effects using the multi-scan method (SADABS). The ratio of minimum to maximum apparent transmission was 0.900. The calculated minimum and maximum transmission coefficients (based on crystal size) are 0.9150 and 0.9570.

The structure was solved and refined using the Bruker SHELXTL Software Package, using the space group $P2_1$, with Z = 4 for the formula unit, $C_{29}H_{49}N_3O_2$. The final anisotropic full-matrix least-squares refinement on F^2 with 625 variables converged at R1 = 3.19%, for the observed data and wR2 = 8.15% for all data. The goodness-of-fit was 1.036. The largest peak in the final difference electron density synthesis was 0.161 $e^-/Å^3$ and the largest hole was -0.128 $e^-/Å^3$ with an RMS deviation of 0.027 $e^-/Å^3$. On the basis of the final model, the calculated density was 1.137 g/cm^3 and F(000), 1040 e^-.

CCDC number: 2357456

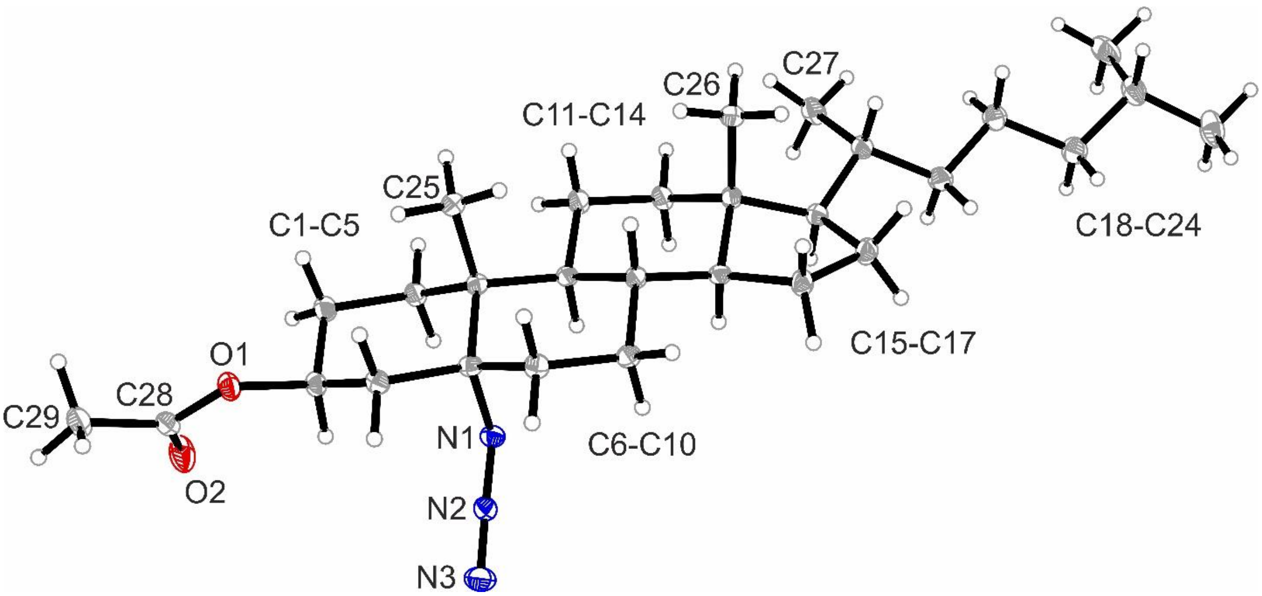

Supplementary Figure 6: Crystal structure of compound **8q**. Thermal ellipsoids are set at 30% probability. Only one (molecule with suffix A) of two independent molecules found in the asymmetric unit is shown.

2. Supplementary Tables

2.1 Optimization Studies

General procedure for optimization reactions

An oven-dried Schlenk tube equipped with a magnetic stir bar was charged with the iron precatalyst and the solvents were added under argon atmosphere (1 mL total). The alkene (0.1 mmol) was added, followed by the radical trap and then the silane. Conversion and yield were determined by GC-FID with *n*-dodecane as internal standard. Reactions were run until no further conversion is observed (GC check).

On the following pages are the tables of optimization. Yellow colors signify the important changes in reaction conditions. Green colors indicate significant results.

Optimization Hydrobromination

Supplementary Table 1: Optimization of the hydrobromination of terminal alkenes. Reactions done on 0.1 mmol scale and run until completion. Yields were determined by GC analysis using *n*-dodecane as internal standard.

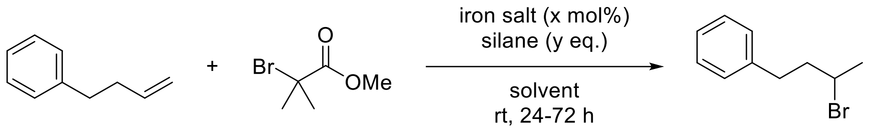

Entry	Precatalyst	Silane	Radical trap eq.	Solvent	Other deviation	GC Conversion	GC Yield
1	$Fe(dpm)_3$ (10 mol%)	$PhSiOiPrH_2$ (2 eq.)	1 eq.	THF/iPrOH		88%	71%
2	$Fe(dpm)_3$ (10 mol%)	$PhSiOiPrH_2$ (2 eq.)	2 eq.	THF/iPrOH		99%	86%
3	$Fe(dpm)_3$ (10 mol%)	$PhSiOiPrH_2$ (2 eq.)	3 eq.	THF/iPrOH		99%	88%
4	$Fe(dpm)_3$ (10 mol%)	$PhSiOiPrH_2$ (2 eq.)	4 eq.	THF/iPrOH		99%	89%
5	$Fe(dpm)_3$ (10 mol%)	$PhSiOiPrH_2$ (2 eq.)	1.5 eq.	THF/iPrOH		98%	85%
6	$Fe(dpm)_3$ (10 mol%)	$PhSiH_3$ (2 eq.)	1.5 eq.	THF/iPrOH		99%	79%
7	$Fe(dpm)_3$ (10 mol%)	$Si(OEt)_3H$ (2 eq.)	1.5 eq.	THF/iPrOH		7%	8%
8	$Fe(dpm)_3$ (10 mol%)	TMDSO (2 eq.)	1.5 eq.	THF/iPrOH		2%	2%
9	$Fe(dpm)_3$ (1 mol%)	$PhSiOiPrH_2$ (2 eq.)	1.5 eq.	THF/iPrOH		40%	34%
10	$Fe(dpm)_3$ (2.5 mol%)	$PhSiOiPrH_2$ (2 eq.)	1.5 eq.	THF/iPrOH		75%	63%
11	$Fe(dpm)_3$ (5 mol%)	$PhSiOiPrH_2$ (2 eq.)	1.5 eq.	THF/iPrOH		91%	74%
12	$Fe(acac)_3$ (10 mol%)	$PhSiOiPrH_2$ (2 eq.)	1.5 eq.	THF/iPrOH		90%	79%
13	$Fe(acac)_3$ (10 mol%)	$PhSiH_3$ (2 eq.)	1.5 eq.	THF/iPrOH		93%	56%
14	$Fe(acac)_3$ (10 mol%)	$PhSiH_3$ (2 eq.)	1.5 eq.	Methanol		98%	76%
15	$Fe(acac)_3$ (5 mol%)	$PhSiH_3$ (2 eq.)	1.5 eq.	Methanol		48%	40%
16	$Fe(acac)_3$ (2.5 mol%)	$PhSiH_3$ (2 eq.)	1.5 eq.	Methanol		21%	17%
17	$Fe(acac)_3$ (2.5 mol%)	$PhSiH_3$ (4 eq.)	1.5 eq.	Methanol		10%	7%
18	$Fe(acac)_3$ (2.5 mol%)	$PhSiH_3$ (2 eq.)	3 eq.	Methanol		18%	17%
19	$Fe(acac)_3$ (5 mol%)	$PhSiH_3$ (1.5 eq.)	1.5 eq.	Methanol		55%	48%
20	$Fe(acac)_3$ (5 mol%)	$PhSiH_3$ (4 eq.)	1.5 eq.	Methanol		37%	31%
21	$Fe(acac)_3$ (10 mol%)	$PhSiH_3$ (1.5 eq.)	1.5 eq.	Methanol		85%	76%
22	$Fe(acac)_3$ (10 mol%)	$PhSiH_3$ (1.2 eq.)	1.5 eq.	Methanol		99%	82%
24	$Fe(acac)_3$ (10 mol%)	$PhSiH_3$ (1.2 eq.)	1.5 eq.	Methanol	40 °C (24 h)	96%	81%
23	$Fe(acac)_3$ (10 mol%)	$PhSiH_3$ (1 eq.)	1.5 eq.	Methanol		94%	84%
25	$Fe(acac)_3$ (10 mol%)	$PhSiH_3$ (1 eq.)	1.5 eq.	Methanol	50 °C (2 h)	72%	63%
26	Fe(acac)3 (10 mol%)	$PhSiH_3$ (1 eq.)	1.2 eq.	Methanol		98%	98%
27	$Fe(acac)_3$ (10 mol%)	$PhSiH_3$ (1 eq.)	1.2 eq.	Methanol	0.5 mmol, double concentration	96%	94%

Supplementary Table 2: Optimization of the hydrobromination of substituted alkenes. Reactions done on 0.1 mmol scale and run until completion. Yields were determined by GC analysis using *n*-dodecane as internal standard.

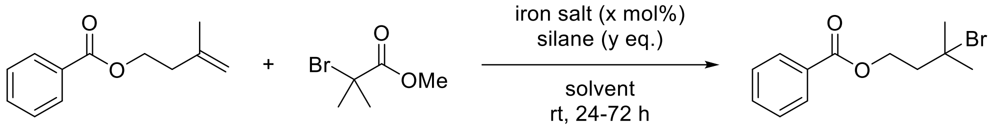

Entry	Precatalyst	Silane	Radical trap eq.	Solvent	Other deviation	GC Conversion	GC Yield
1	$Fe(dpm)_3$ (10 mol%)	$PhSiOiPrH_2$ (2 eq.)	2 eq.	THF/iPrOH		57%	27%
2	$Fe(acac)_3$ (10 mol%)	$PhSiH_3$ (1.5 eq)	1.5 eq.	MeOH		41%	9%
3	$Fe(acac)_3$ (10 mol%)	$PhSiH_3$ (1.5 eq)	5.0 eq.	MeOH		48%	13%
4	$Fe(dpm)_3$ (10 mol%)	$PhSiOiPrH_2$ (2 eq.)	5.0 eq.	THF/iPrOH		52%	22%
5	$Fe(dpm)_3$ (10 mol%)	$PhSiOiPrH_2$ (2 eq.)	2 eq.	THF/iPrOH	40 °C	64%	26%
6	$Fe(dpm)_3$ (10 mol%)	$PhSiOiPrH_2$ (2 eq.)	2 eq.	THF/iPrOH	Methyl 2-Br-acetate as trap	28%	3%
7	$Fe(acac)_3$ (20 mol%)	$PhSiH_3$ (1.5 eq)	1.5 eq.	MeOH		66%	14%
8	$Fe(dpm)_3$ (10 mol%)	$PhSiOiPrH_2$ (2 eq.)	1.1 eq.	THF/iPrOH		92%	80%
9	$Fe(dpm)_3$ (10 mol%)	$PhSiOiPrH_2$ (1 eq.)	1.1 eq.	THF/iPrOH		77%	75%
10	$Fe(dpm)_3$ (10 mol%)	$PhSiOiPrH_2$ (3 eq.)	1.1 eq.	THF/iPrOH		92%	78%
11	$Fe(dpm)_3$ (10 mol%)	$PhSiH_3$ (2 eq.)	1.1 eq.	THF/MeOH		75%	46%
12	$Fe(dpm)_3$ (10 mol%)	$PhSiOiPrH_2$ (2 eq.)	1.1 eq.	THF/iPrOH		89%	79%
13	$Fe(dpm)_3$ (10 mol%)	$PhSiOiPrH_2$ (1.2 eq.)	1.1 eq.	THF/iPrOH		90%	81%
14	$Fe(dpm)_3$ (10 mol%)	$PhSiOiPrH_2$ (1.2 eq.)	1.45 eq.	THF/iPrOH		80%	63%
15	$Fe(dpm)_3$ (10 mol%)	$PhSiOiPrH_2$ (1.2 eq.)	2.2 eq.	THF/iPrOH		77%	63%
16	$Fe(dpm)_3$ (10 mol%)	$PhSiOiPrH_2$ (2 eq.)	1.5 eq.	THF/iPrOH	*p*TsBr as trap	15%	2%
17	$Fe(dpm)_3$ (10 mol%)	$PhSiH_3$ (1.2 eq)	1.1 eq.	THF/iPrOH	$Na_2HPO_4•7H_2O$ (1.2 eq.)[a]	55%	18%
18	$Fe(acac)_3$ (10 mol%)	$PhSiH_3$ (1.2 eq)	1.1 eq.	THF/iPrOH	$Na_2HPO_4•7H_2O$ (1.2 eq.)[a]	11%	0%
19	$Fe(dpm)_3$ (10 mol%)	$PhSiH_3$ (1.2 eq)	1.1 eq.	THF/MeOH	$Na_2HPO_4•7H_2O$ (1.2 eq.)[a]	32%	8%

[a]Additive based on *Li* et al. for in situ generation of monoalkoxysilanes.[42]

Optimization Hydroiodination

Supplementary Table 3: Optimization of the hydroiodination. Reactions done on 0.1 mmol scale and run until completion. Yields were determined by GC analysis using *n*-dodecane as internal standard. n.d. = not determinable because of signal overlap of IS with the radical trap.

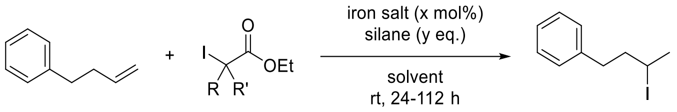

2b (R = Me, R' = Me)
2c (R = H, R' = H)
2d (R = F, R' = F)
2e (R = H, R' = Me)
2f (R = H, R' = Ph)

Entry	Precatalyst	Silane	Radical trap eq.	Solvent	Other deviation	GC Conversion	GC Yield
1	$Fe(acac)_3$ (10 mol%)	$PhSiH_3$ (2 eq.)	**2c** (3 eq.)	Methanol		33%	12%
2	$Fe(acac)_3$ (10 mol%)	$PhSiH_3$ (2 eq.)	**CH_2I_2** (3 eq.)	Methanol		11%	6%
3	$Fe(acac)_3$ (10 mol%)	$PhSiH_3$ (2 eq.)	**2b** (3 eq.)	Methanol		n.d.	8%
4	$Fe(dpm)_3$ (10 mol%)	$PhSiOiPrH_2$ (2 eq.)	**CHI_3** (3 eq.)	THF/iPrOH		0%	0%
5	$Fe(dpm)_3$ (10 mol%)	$PhSiOiPrH_2$ (2 eq.)	**2c** (3 eq.)	THF/iPrOH		49%	39%
6	$Fe(dpm)_3$ (10 mol%)	$PhSiOiPrH_2$ (2 eq.)	**2b** (3 eq.)	THF/iPrOH		n.d.	0%
7	$Fe(dpm)_3$ (10 mol%)	$PhSiOiPrH_2$ (2 eq.)	**2d** (3 eq.)	THF/iPrOH		49%	0%
8	$Fe(acac)_3$ (10 mol%)	$PhSiH_3$ (2 eq.)	**2e** (3 eq.)	Methanol		33%	25%
9	$Fe(acac)_3$ (10 mol%)	$PhSiH_3$ (2 eq.)	**2f** (3 eq.)	Methanol		0%	0%
10	$Fe(acac)_3$ (10 mol%)	$PhSiH_3$ (2 eq.)	**2e** (1.5 eq.)	Methanol		42%	38%
11	$Fe(acac)_3$ (10 mol%)	$PhSiH_3$ (2 eq.)	**2e** (1 eq.)	Methanol		89%	85%
12	$Fe(acac)_3$ (10 mol%)	$PhSiH_3$ (1 eq.)	**2e** (1 eq.)	Methanol		89%	89%
13	$Fe(acac)_3$ (10 mol%)	$PhSiH_3$ (4 eq.)	**2e** (1 eq.)	Methanol		50%	41%
14	$Fe(acac)_3$ (10 mol%)	$PhSiH_3$ (1 eq.)	**2e** (1 eq.)	Methanol	40 °C	83%	81%
15	$Fe(acac)_3$ (10 mol%)	$PhSiH_3$ (1 eq.)	**2e** (1 eq.)	Methanol	0.5 mmol, double concentration	87%	84%

Optimization Hydrochlorination

Supplementary Table 4: Optimization of the hydrochlorination of terminal alkenes. Reactions done on 0.1 mmol scale and run until completion. Yields were determined by GC analysis using *n*-dodecane as internal standard.

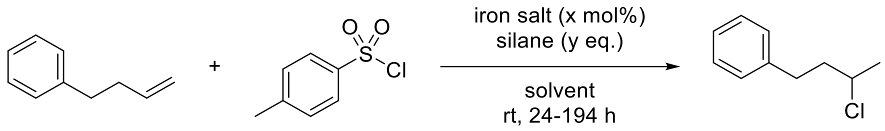

Entry	Precatalyst	Silane	Radical trap eq.	Solvent	Other deviation	GC Conversion	GC Yield
1	$Fe(dpm)_3$ (10 mol%)	$PhSiOiPrH_2$ (2 eq.)	1.5 eq.	THF/iPrOH		75%	62%
2	$Fe(dpm)_3$ (10 mol%)	$PhSiOiPrH_2$ (2 eq.)	1.1 eq.	THF/iPrOH		79%	61%
3	$Fe(dpm)_3$ (10 mol%)	$PhSiOiPrH_2$ (1 eq.)	1.5 eq.	THF/iPrOH		51%	46%
4	$Fe(dpm)_3$ (10 mol%)	$PhSiOiPrH_2$ (1 eq.)	1.1 eq.	THF/iPrOH		52%	47%
5	$Fe(acac)_3$ (10 mol%)	$PhSiH_3$ (1 eq)	1.1 eq.	MeOH		10%	10%
6	$Fe(dpm)_3$ (10 mol%)	$PhSiOiPrH_2$ (1 eq.)	1.1 eq.	THF/iPrOH	less iPrOH	42%	38%
7	$Fe(dpm)_3$ (10 mol%)	$PhSiOiPrH_2$ (1 eq.)	1.1 eq.	THF/iPrOH	40 °C	51%	47%
8	$Fe(dpm)_3$ (20 mol%)	$PhSiOiPrH_2$ (1 eq.)	1.1 eq.	THF/iPrOH		57%	51%
9	$Fe(dpm)_3$ (10 mol%)	$PhSiOiPrH_2$ (1 eq.)	1.1 eq.	THF/iPrOH	*p*-OMe-C_6H_4-SO_2Cl as trap	47%	40%
10	$Fe(dpm)_3$ (10 mol%)	$PhSiOiPrH_2$ (1 eq.)	1.1 eq.	THF/iPrOH	*p*-CF_3-C_6H_4-SO_2Cl as trap	40%	33%
11	$Fe(dpm)_3$ (5 mol%)	$PhSiOiPrH_2$ (1 eq.)	1.1 eq.	THF/iPrOH		46%	42%
12	$Fe(acac)_3$ (10 mol%)	$PhSiH_3$ (1 eq)	1.1 eq.	MeOH	Methyl 2-Cl-2-Me-propanoate as trap	34%	0%
13	$Fe(dpm)_3$ (10 mol%)	$PhSiOiPrH_2$ (1 eq.)	1.1 eq.	THF/iPrOH	added molecular sieve 3A	54%	49%
14	$Fe(dpm)_3$ (10 mol%)	$PhSiH_3$ (2 eq)	1.5 eq.	THF/iPrOH		43%	38%
15	$Fe(dpm)_3$ (10 mol%)	$PhSiOiPrH_2$ (3 eq.)	1.1 eq.	THF/iPrOH		79%	71%
16	$Fe(acac)_3$ (10 mol%)	$PhSiH_3$ (4 eq)	1.1 eq.	THF/iPrOH		63%	56%
17	$Fe(acac)_3$ (10 mol%)	$PhSiH_3$ (4 eq)	1.5 eq.	THF/iPrOH		81%	80%
18	$Fe(acac)_3$ (10 mol%)	$PhSiH_3$ (3 eq)	1.5 eq.	THF/iPrOH	0.5 mmol scale	75%	75%
19	$Fe(dpm)_3$ (10 mol%)	$PhSiOiPrH_2$ (3 eq.)	1.5 eq.	THF/iPrOH	0.2 mmol scale	72%	67%
20	$Fe(acac)_3$ (10 mol%)	$PhSiH_3$ (4 eq)	1.5 eq.	THF/iPrOH	0.5 mmol scale	86%	69%
21	$Fe(acac)_3$ (10 mol%)	$PhSiH_3$ (3 eq)	1.5 eq.	THF/iPrOH	40°C 0.5 mmol scale	67%	62%
22	$Fe(acac)_3$ (10 mol%)	$PhSiH_3$ (4 eq)	1.5 eq.	THF/iPrOH	40°C 0.5 mmol scale	81%	77%

Supplementary Table 5: Optimization of the hydrochlorination of substituted alkenes. Reactions done on 0.1 mmol scale and run until completion. Yields were determined by GC analysis using *n*-dodecane as internal standard.

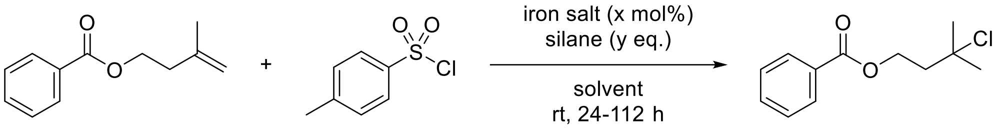

Entry	Precatalyst	Silane	Radical trap eq.	Solvent	Other deviation	GC Conversion	GC yield
1	$Fe(dpm)_3$ (10 mol%)	$PhSiOiPrH_2$ (2 eq.)	3 eq.	THF/iPrOH		77%	77%
2	$Fe(dpm)_3$ (10 mol%)	$PhSiOiPrH_2$ (2 eq.)	1.5 eq.	THF/iPrOH		76%	74%
3	$Fe(dpm)_3$ (10 mol%)	$PhSiOiPrH_2$ (2 eq.)	1.5 eq.	THF/iPrOH	50°C	67%	56%
4	$Fe(dpm)_3$ (5 mol%)	$PhSiOiPrH_2$ (2 eq.)	1.5 eq.	THF/iPrOH		58%	63%
5	$Fe(dpm)_3$ (10 mol%)	$PhSiH_3$ (2 eq)	1.5 eq.	THF/iPrOH		33%	32%
6	$Fe(dpm)_3$ (10 mol%)	$PhSiH_3$ (2 eq)	1.1 eq.	THF/iPrOH		51%	47%
7	$Fe(acac)_3$ (10 mol%)	$PhSiH_3$ (2 eq)	1.5 eq.	THF/iPrOH		36%	33%
8	$Fe(dpm)_3$ (10 mol%)	$PhSiOiPrH_2$ (1.2 eq.)	1.5 eq.	THF/iPrOH		63%	63%
9	$Fe(dpm)_3$ (10 mol%)	$PhSiOiPrH_2$ (2 eq.)	1.5 eq.	THF/iPrOH	connected to air with needle	57%	50%
10	$Fe(dpm)_3$ (10 mol%)	$PhSiOiPrH_2$ (2 eq.)	1.5 eq.	only THF		51%	21%
11	$Fe(dpm)_3$ (10 mol%)	$PhSiOiPrH_2$ (2 eq.)	1.5 eq.	only iPrOH		59%	51%
12	$Fe(dpm)_3$ (10 mol%)	$PhSiOiPrH_2$ (2 eq.)	1.5 eq.	MeOH		26%	17%
13	$Fe(dpm)_3$ (10 mol%)	$PhSiOiPrH_2$ (2 eq.)	1.5 eq.	DCM/iPrOH		41%	40%
14	$Fe(dpm)_3$ (10 mol%)	$PhSiOiPrH_2$ (2 eq.)	1.5 eq.	THF/tBuOH		33%	32%
15	$Fe(dpm)_3$ (10 mol%)	$PhSiOiPrH_2$ (2 eq.)	1.5 eq.	DCE/iPrOH		46%	41%
16	$Fe(dpm)_3$ (10 mol%)	$PhSiOiPrH_2$ (2 eq.)	1.5 eq.	EtOAc/iPrOH		56%	52%

Optimization Hydroazidation

Supplementary Table 6: Optimization of the hydroazidation of terminal alkenes. Reactions done on 0.1 mmol scale and run until completion. Yields were determined by GC analysis using *n*-dodecane as internal standard.

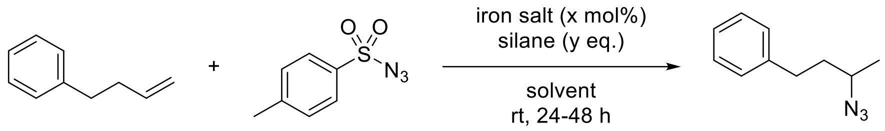

Entry	Precatalyst	Silane	Radical trap eq.	Solvent	Other Deviation	GC Conversion	GC Yield
1	$Fe(acac)_3$ (10mol %)	$PhSiH_3$ (4 eq.)	1.5 eq	THF/iPrOH	40 °C	full conversion	0%
2	$Fe(acac)_3$ (10mol %)	$PhSiH_3$ (1.5 eq.)	1.5 eq	Methanol		37%	26%
3	$Fe(dpm)_3$ (10mol %)	$PhSiOiPrH_2$ (1 eq.)	1.5 eq	THF/iPrOH		59%	48%
4	$Fe(dpm)_3$ (10mol %)	$PhSiOiPrH_2$ (2 eq.)	1.5 eq	THF/iPrOH		76%	62%
5	$Fe(dpm)_3$ (10mol %)	$PhSiH_3$ (2 eq.)	1.5 eq	THF/iPrOH		58%	34%

Supplementary Table 7: Optimization of the hydroazidation of substituted alkenes. Reactions done on 0.1 mmol scale and run until completion. Yields were determined by GC analysis using *n*-dodecane as internal standard.

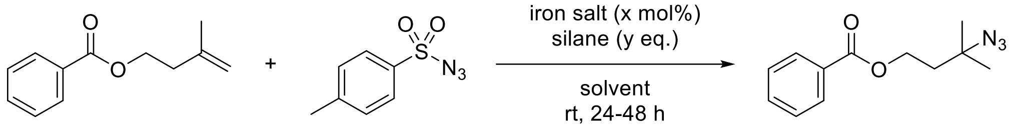

Entry	Precatalyst	Silane	Radical trap eq.	Solvent	Other Deviation	GC Conversion	GC Yield
1	$Fe(dpm)_3$ (10 mol%)	PhSiOiPrH2 (2 eq.)	1.5 eq.	THF/iPrOH		86%	71%
2	$Fe(acac)_3$ (10 mol%)	PhSiOiPrH2 (2 eq.)	1.5 eq.	THF/iPrOH		67%	67%
3	$Fe(3\text{-}Me\text{-}acac)_3$ (10 mol%)	PhSiOiPrH2 (2 eq.)	1.5 eq.	THF/iPrOH		54%	46%
4	$Fe(dpm)_3$ (10 mol%)	PhSiOiPrH2 (1.5 eq.)	1.5 eq.	THF/iPrOH		70%	68%
5	$Fe(dpm)_3$ (10 mol%)	PhSiOiPrH2 (2 eq.)	1.5 eq.	THF/iPrOH	40 °C	84%	67%
6	$Fe(dpm)_3$ (10 mol%)	PhSiOiPrH2 (3 eq.)	1.5 eq.	THF/iPrOH		87%	55%

3. Supplementary Figures

3.1 NMR Spectra

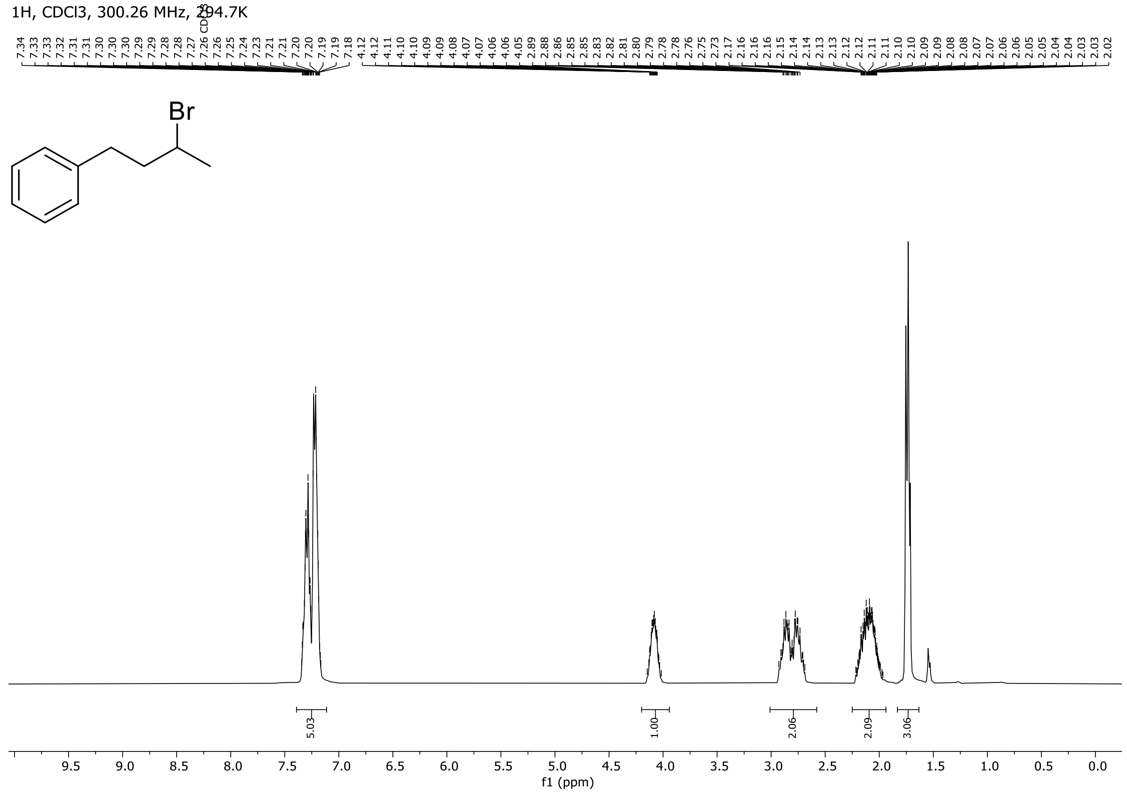

Supplementary Figure 7: ^{1}H NMR of (3-Bromobutyl)benzene (**3a**)

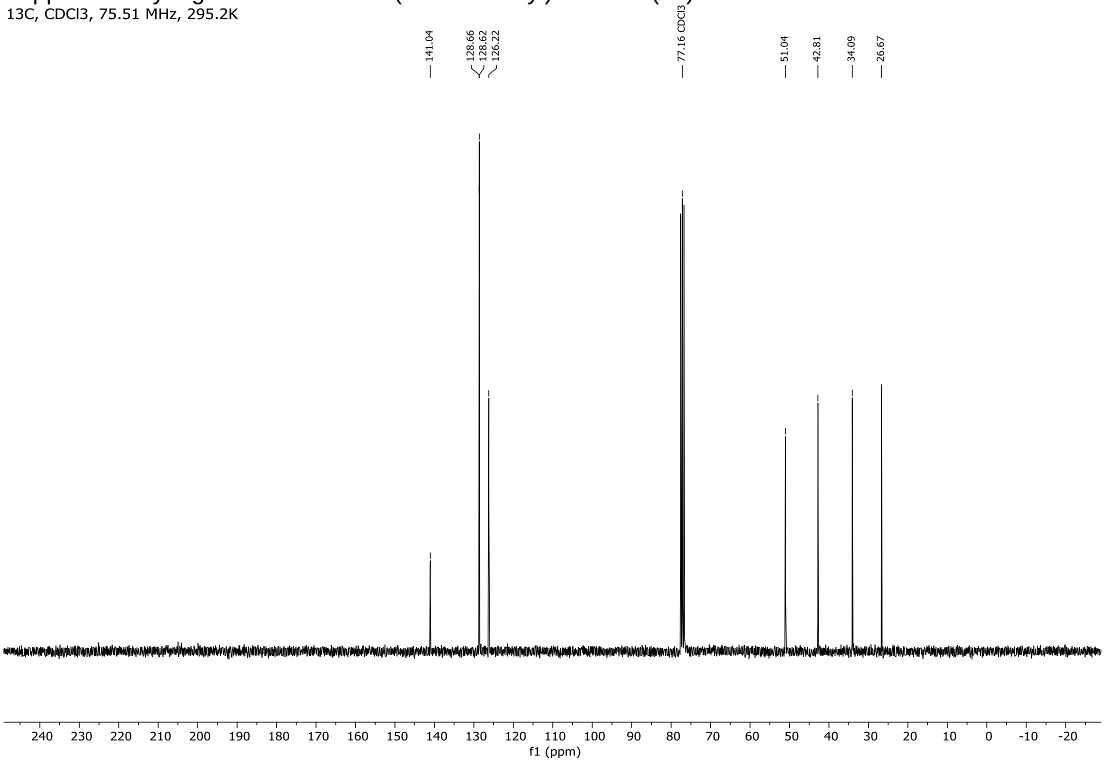

Supplementary Figure 8: ^{13}C NMR of (3-Bromobutyl)benzene (**3a**)

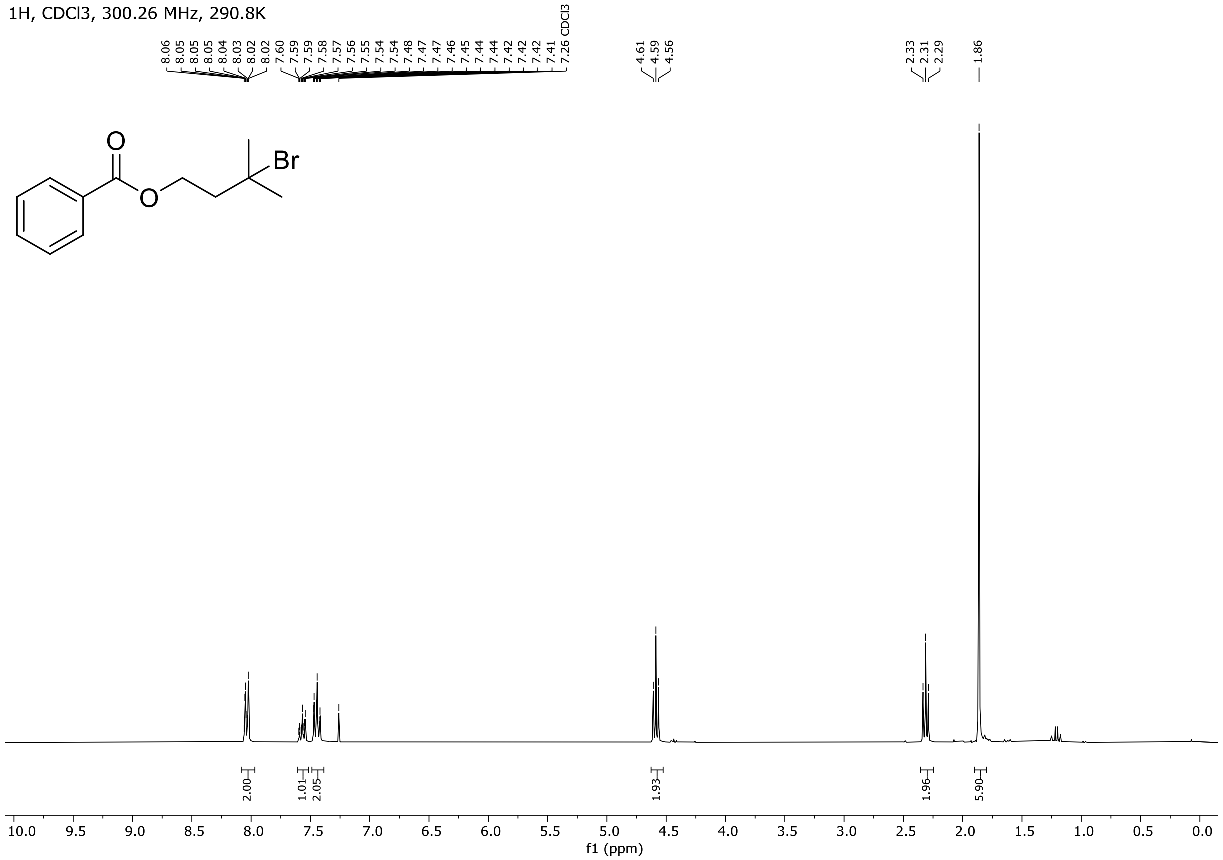

Supplementary Figure 9: ^{1}H NMR of 3-Bromo-3-methylbutyl benzoate (**3b**)

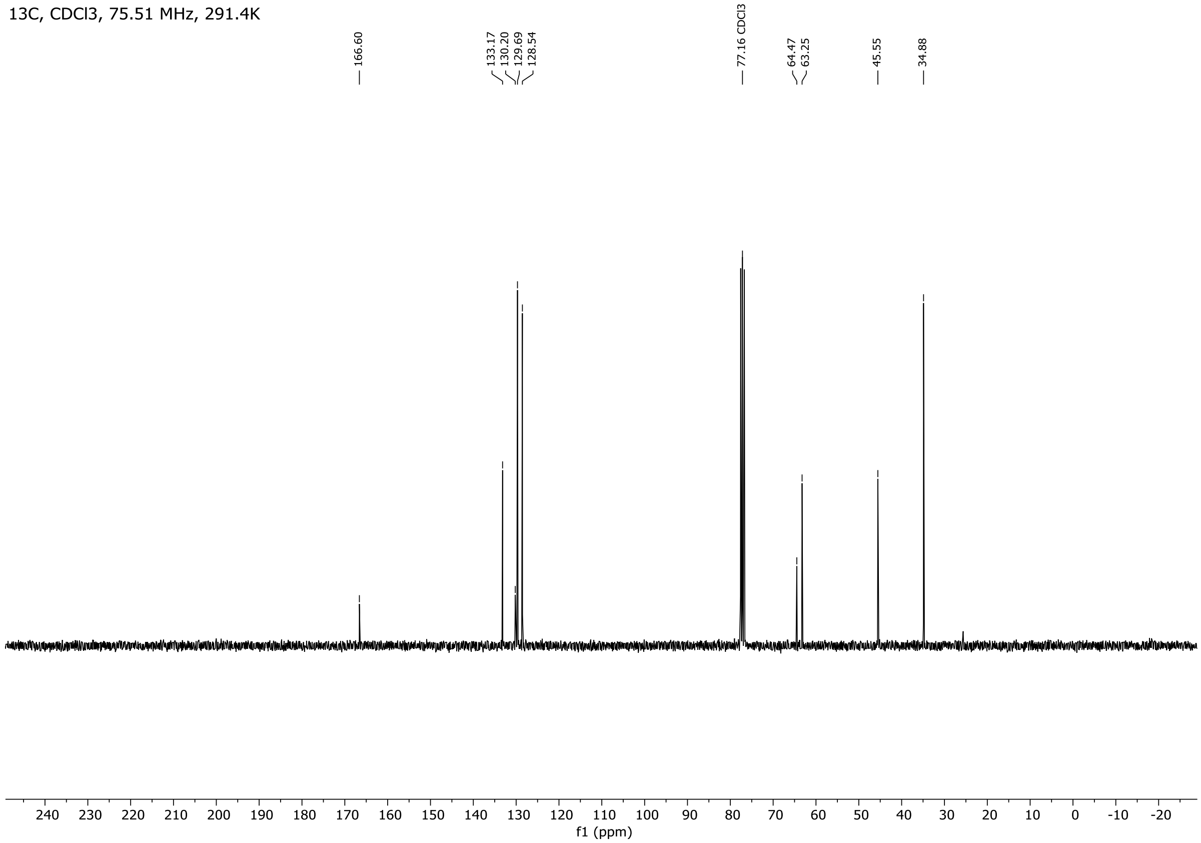

Supplementary Figure 10: ^{13}C NMR of 3-Bromo-3-methylbutyl benzoate (**3b**)

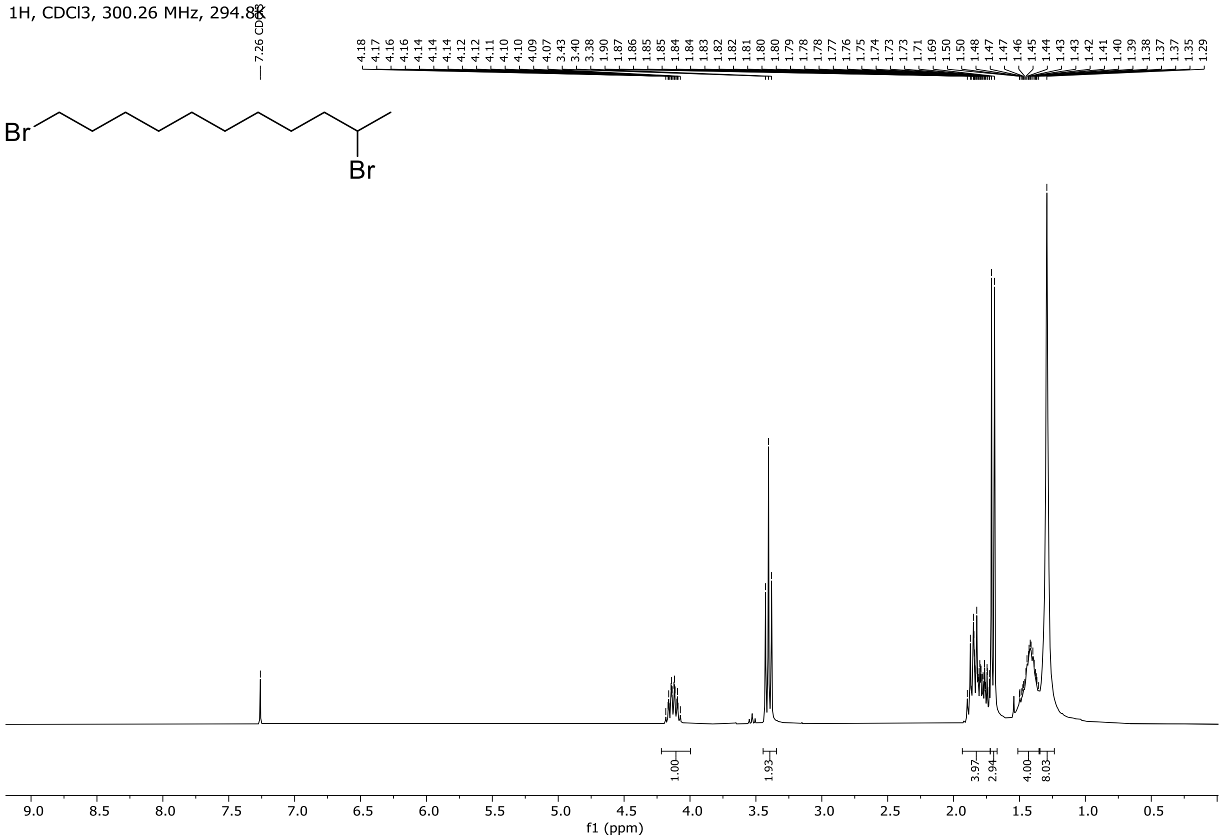

Supplementary Figure 11: ^{1}H NMR of 1,10-Dibromoundecane (**3c**)

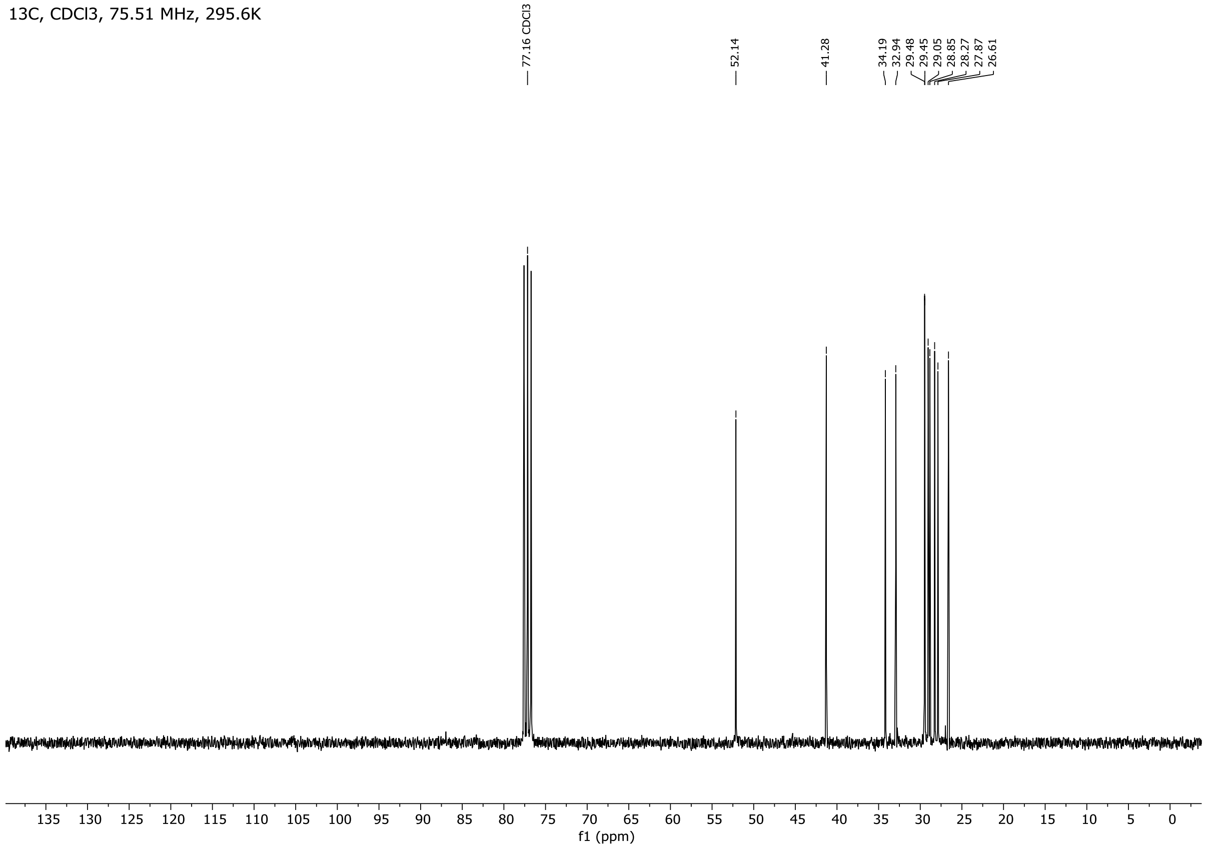

Supplementary Figure 12: ^{13}C NMR of 1,10-Dibromoundecane (**3c**)

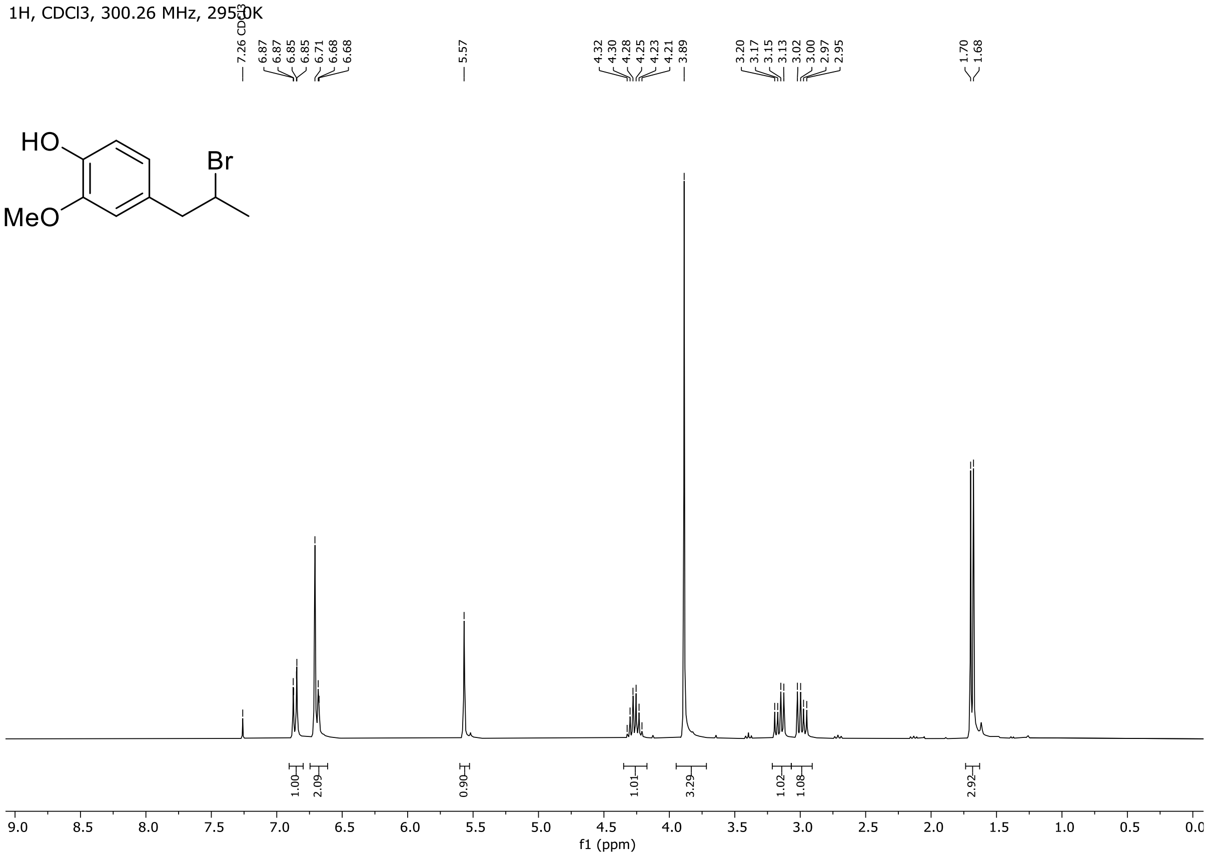

Supplementary Figure 13: ^{1}H NMR of 4-(2-Bromopropyl)-2-methoxyphenol (**3d**)

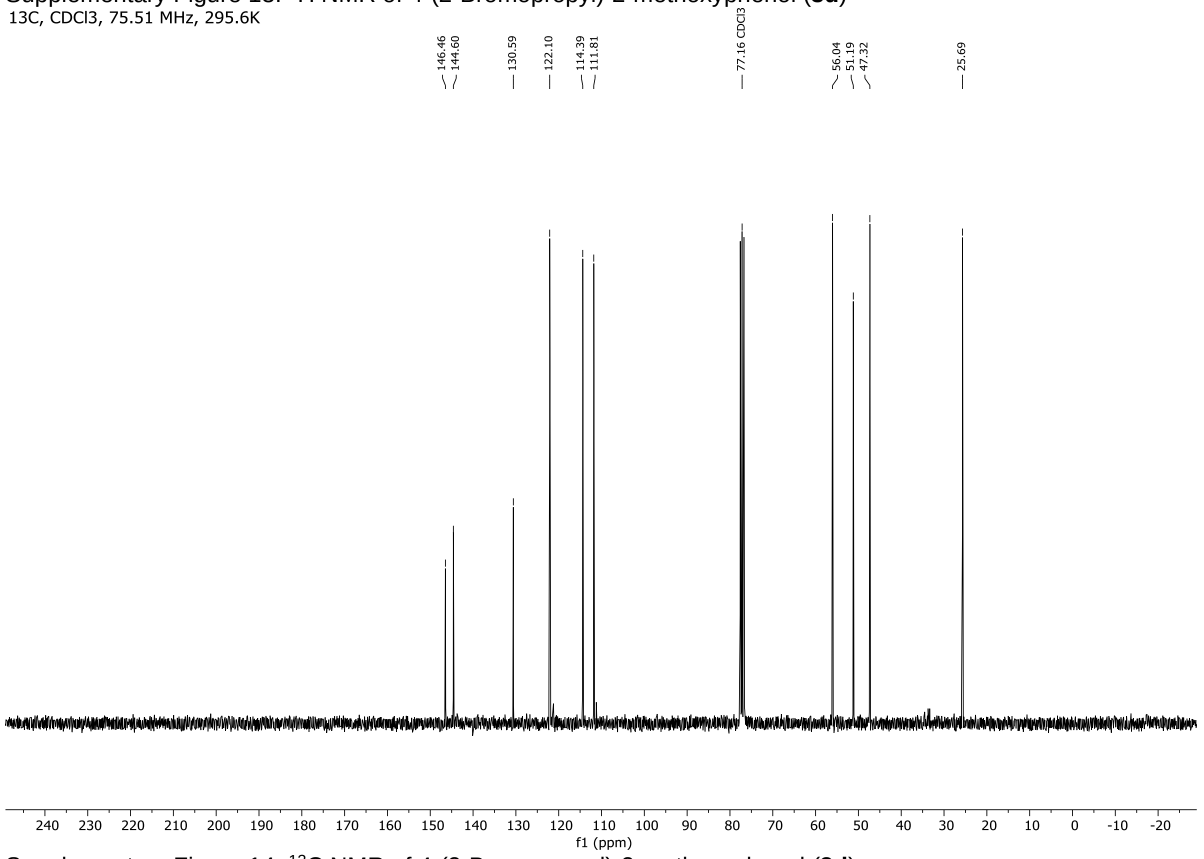

Supplementary Figure 14: ^{13}C NMR of 4-(2-Bromopropyl)-2-methoxyphenol (**3d**)

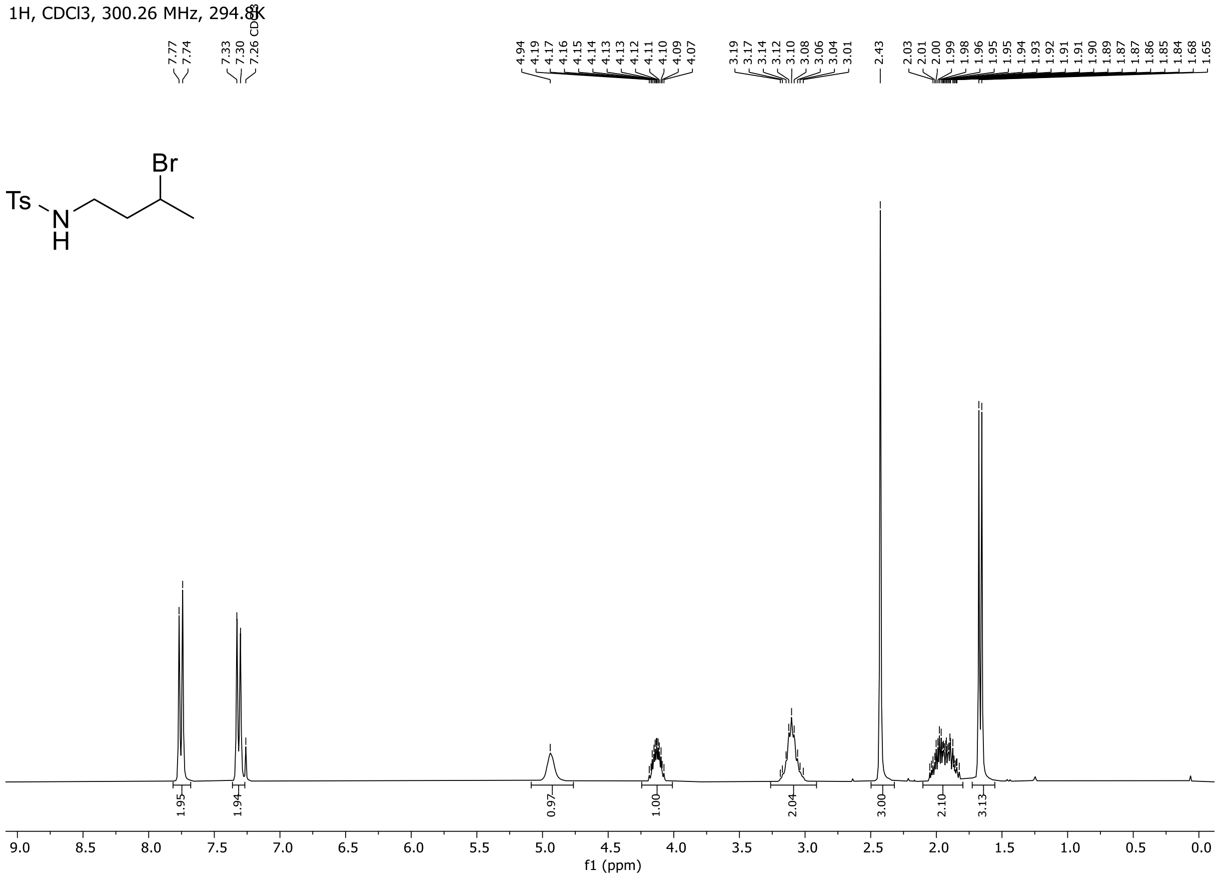

Supplementary Figure 15: ^{1}H NMR of *N*-(3-Bromobutyl)-4-methylbenzenesulfonamide (**3e**)

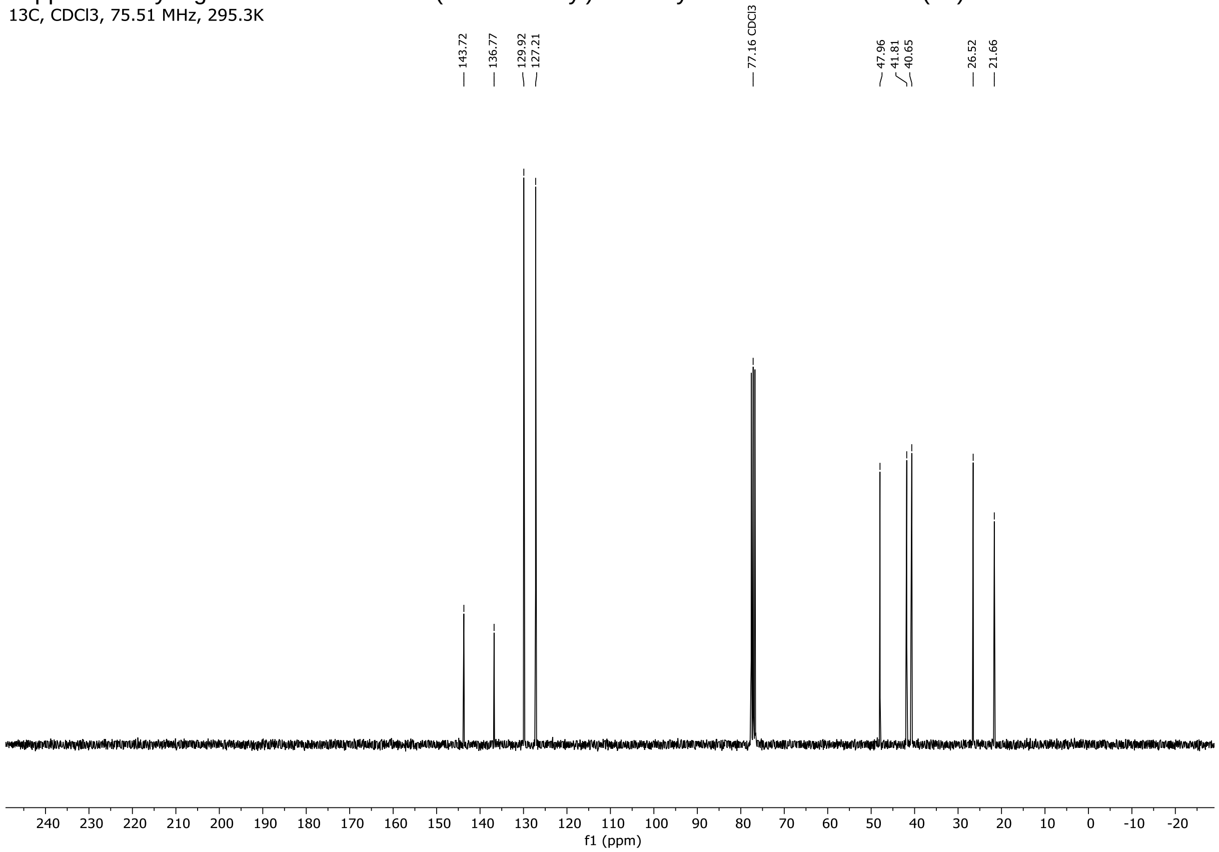

Supplementary Figure 16: ^{13}C NMR of *N*-(3-Bromobutyl)-4-methylbenzenesulfonamide (**3e**)

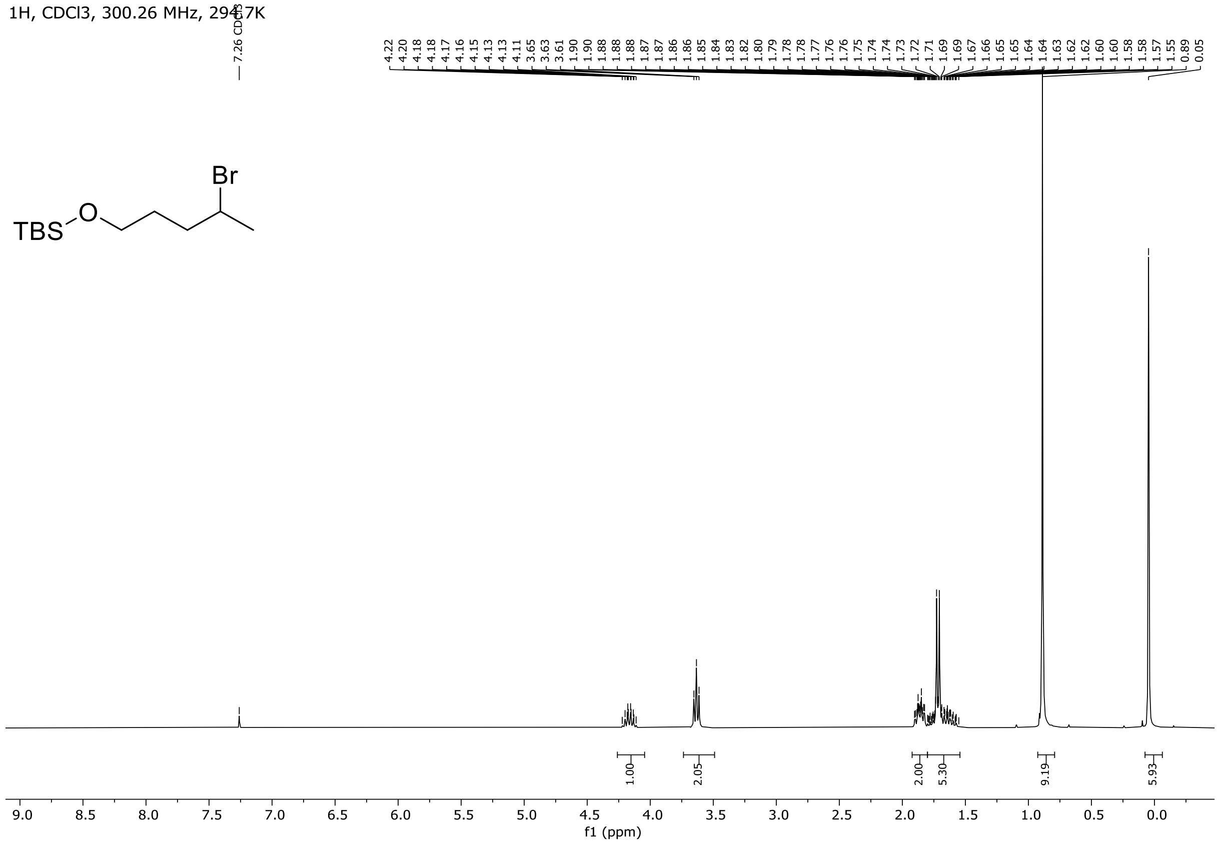

Supplementary Figure 17: ^{1}H NMR of ((4-Bromopentyl)oxy)(*tert*-butyl)dimethylsilane (**3f**)

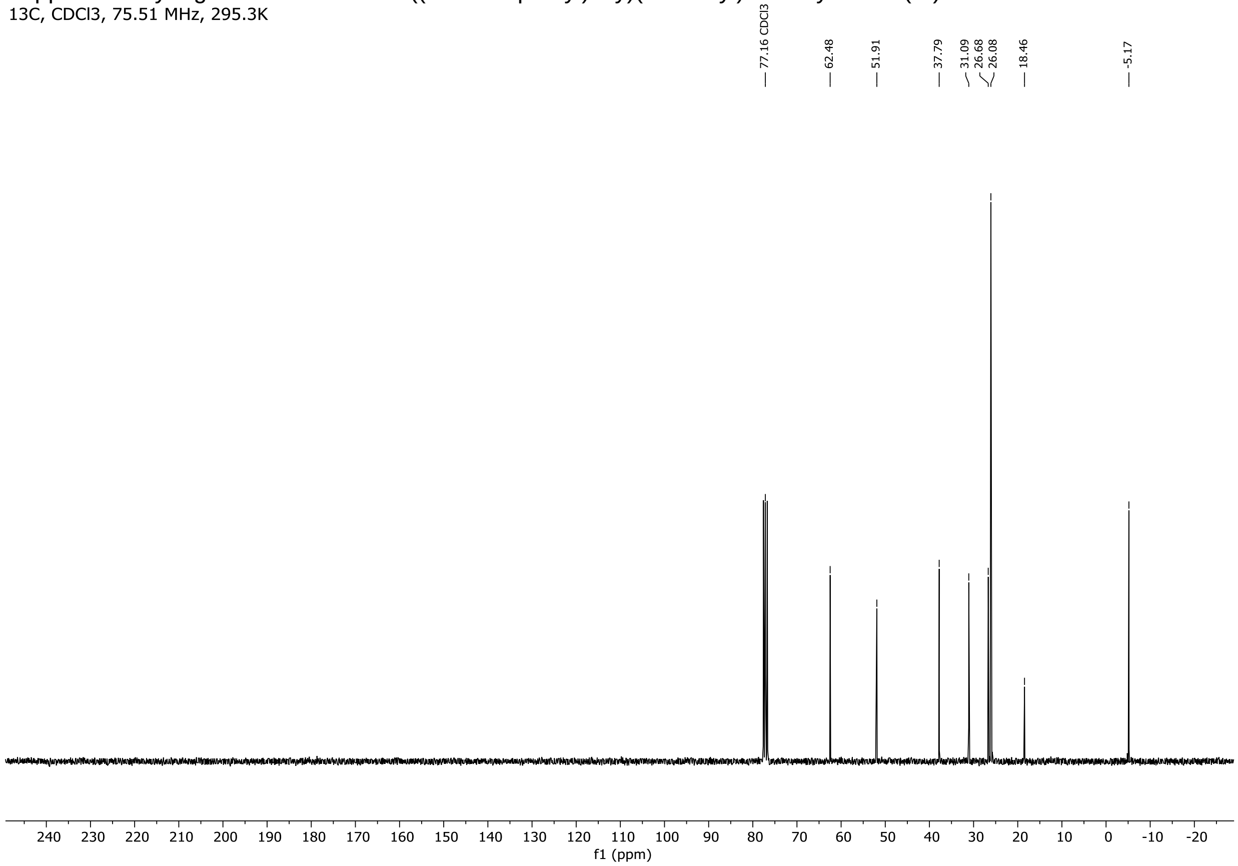

Supplementary Figure 18: ^{13}C NMR of ((4-Bromopentyl)oxy)(*tert*-butyl)dimethylsilane (**3f**)

29Si dept, CDCl3, 59.65 MHz, 296.2 K

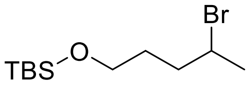

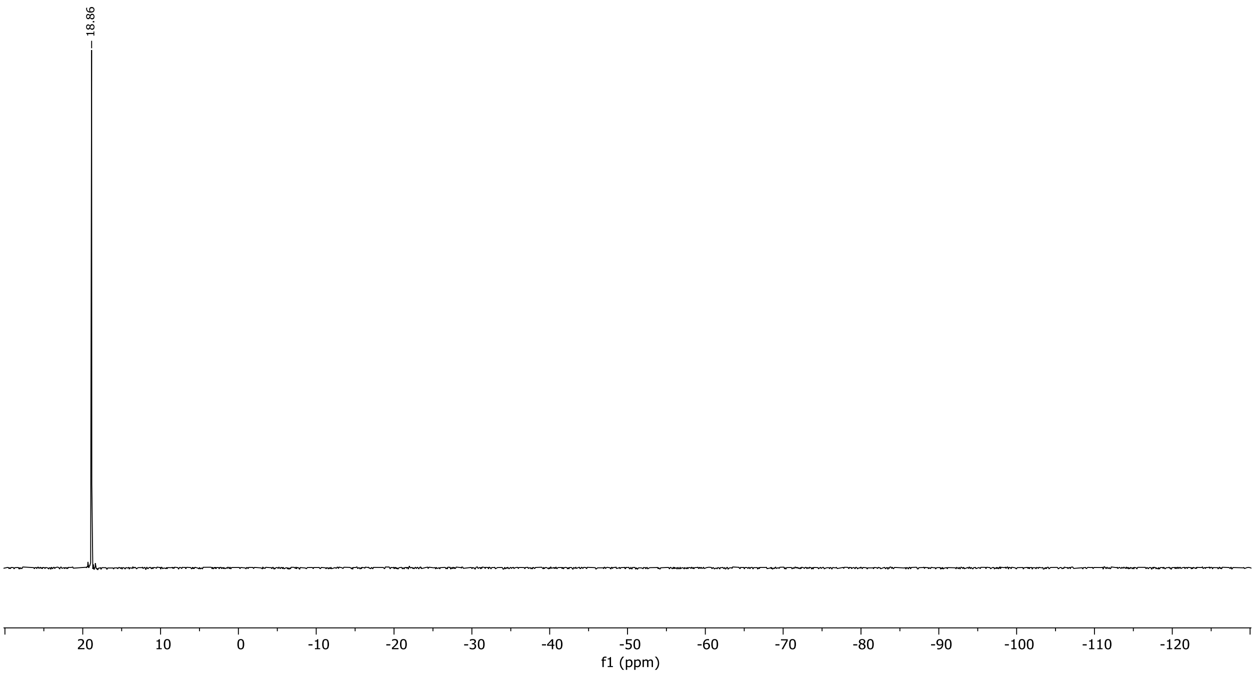

Supplementary Figure 19: ^{29}Si NMR of ((4-Bromopentyl)oxy)(*tert*-butyl)dimethylsilane (**3f**)

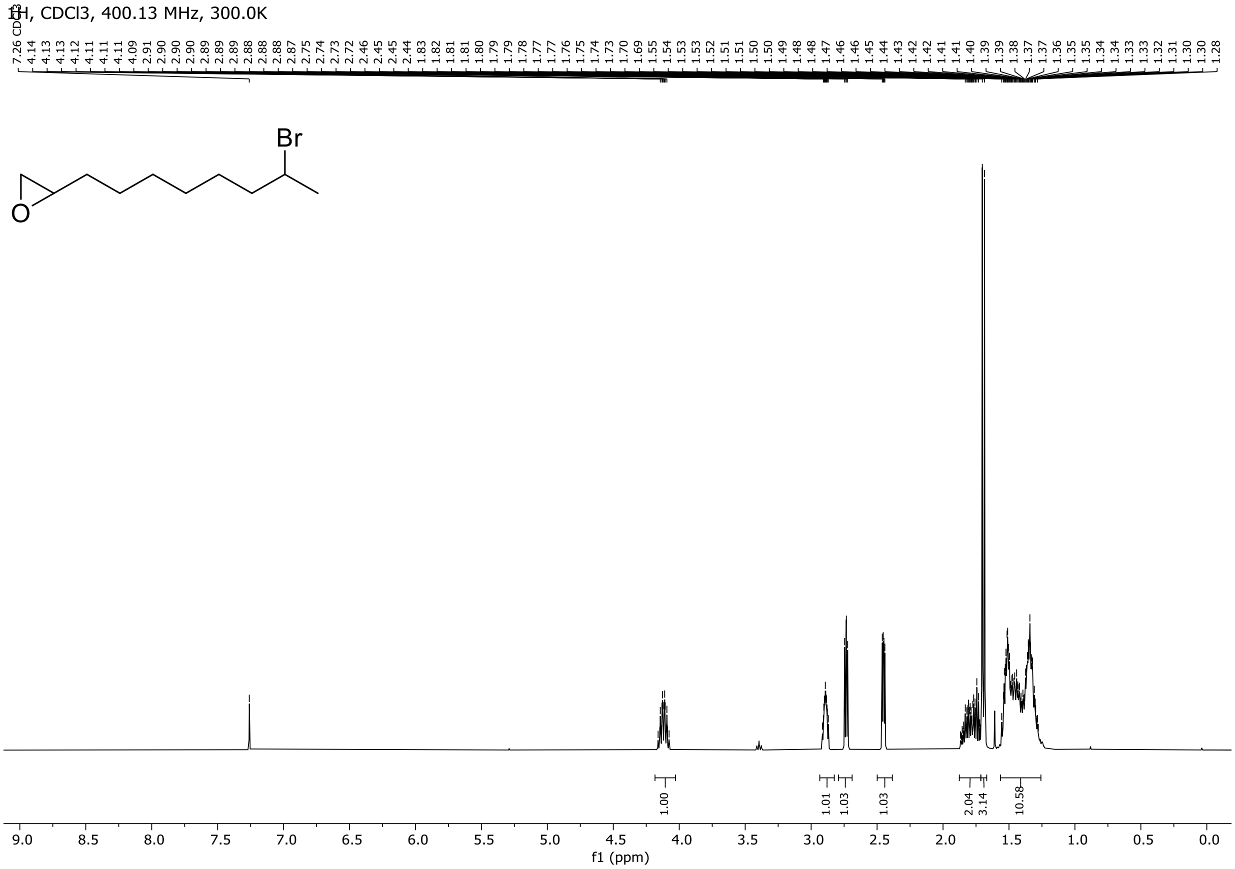

Supplementary Figure 20: ^{1}H NMR of 2-(7-Bromooctyl)oxirane (**3g**)

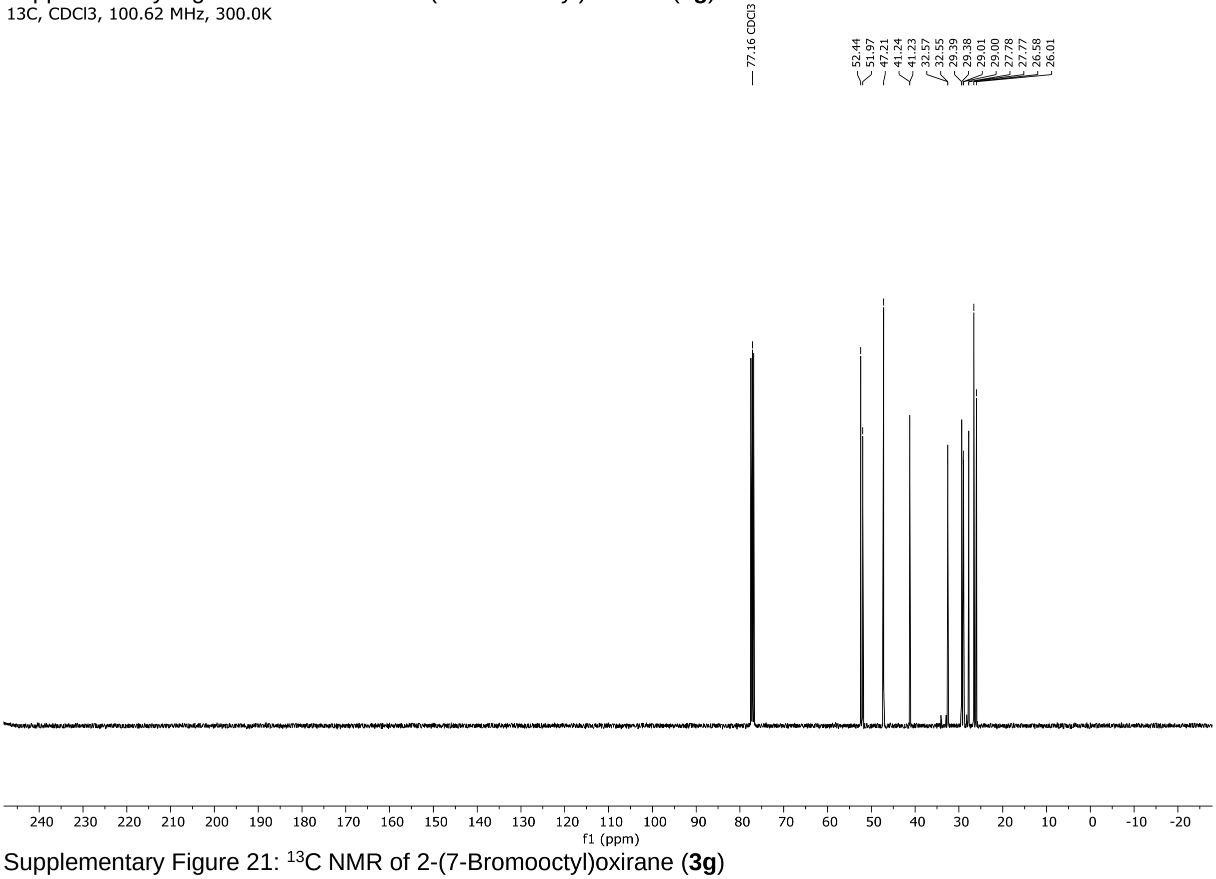

Supplementary Figure 21: ^{13}C NMR of 2-(7-Bromooctyl)oxirane (**3g**)

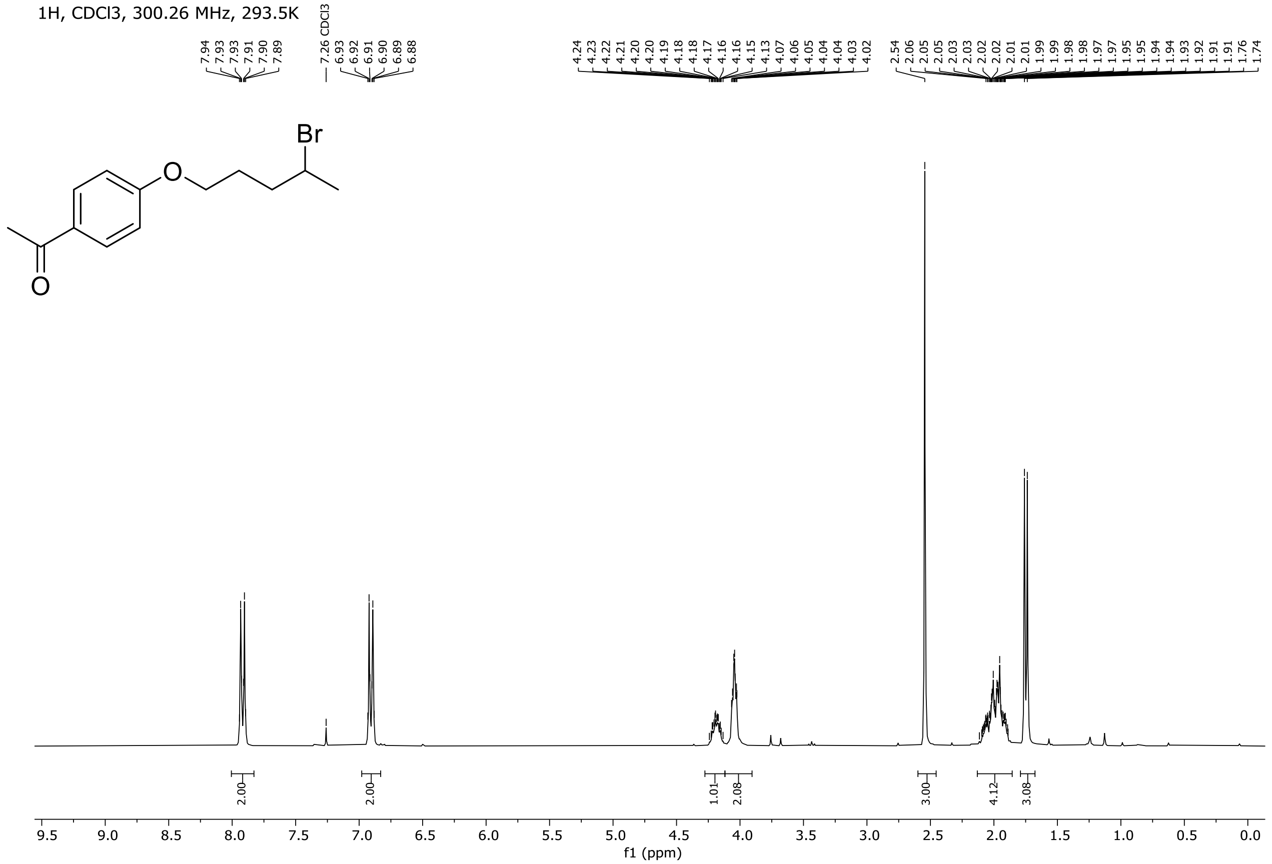

Supplementary Figure 22: ^{1}H NMR of 1-(4-((4-Bromopentyl)oxy)phenyl)ethan-1-one (**3h**)

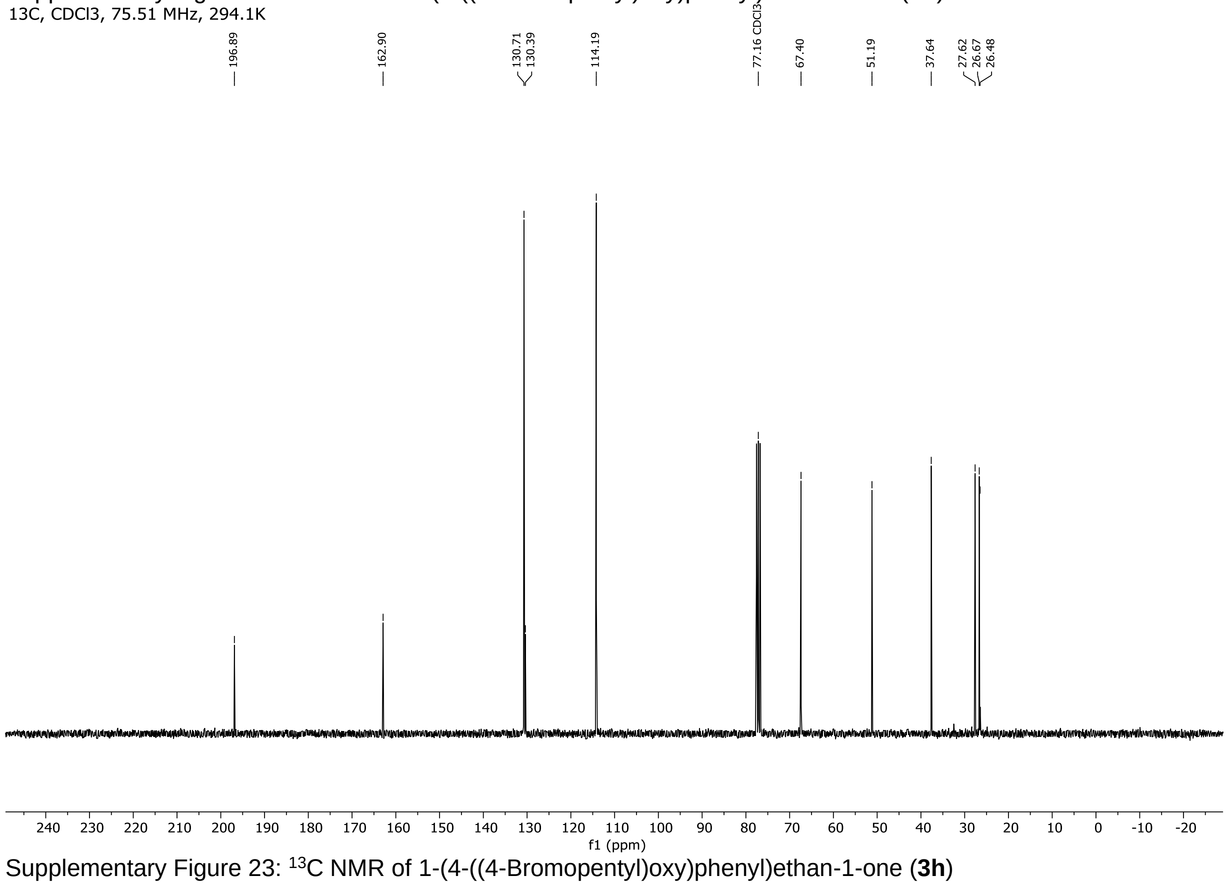

Supplementary Figure 23: ^{13}C NMR of 1-(4-((4-Bromopentyl)oxy)phenyl)ethan-1-one (**3h**)

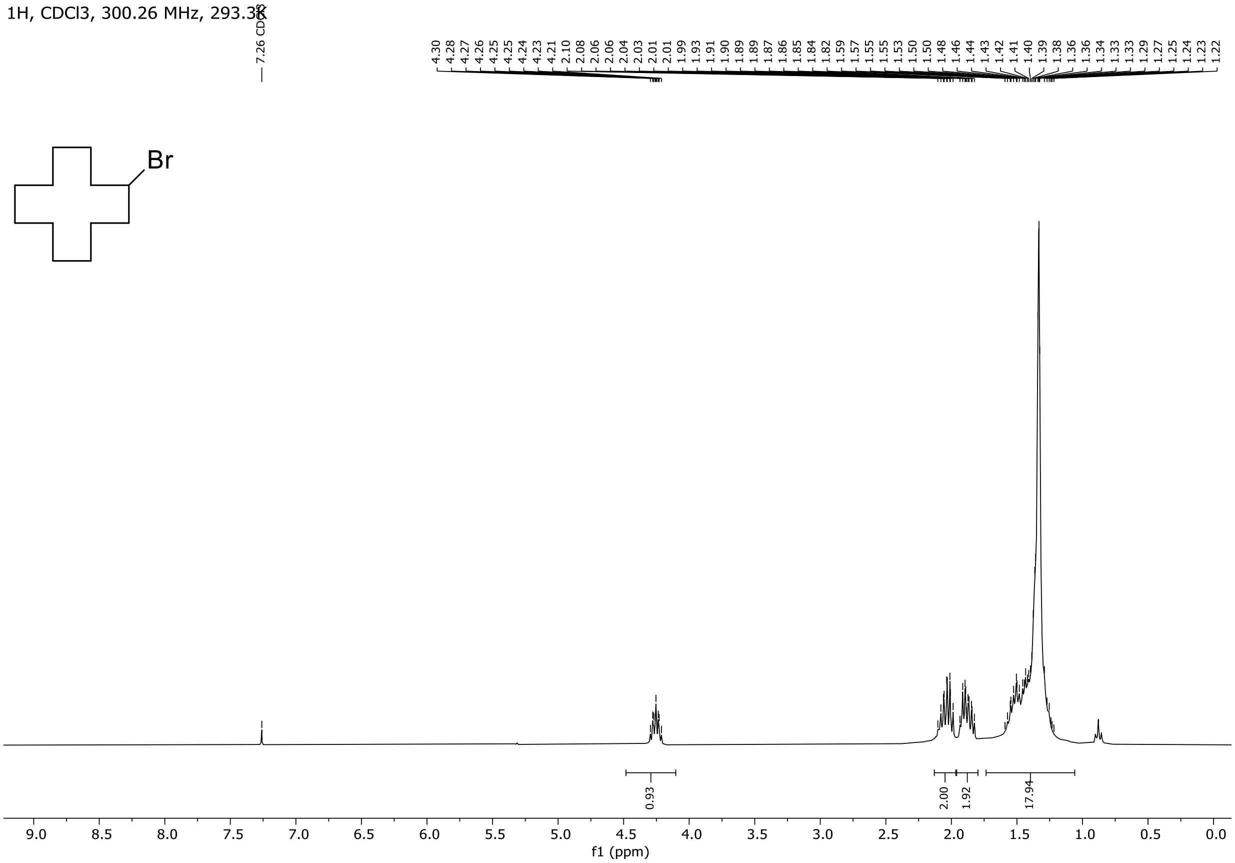

Supplementary Figure 24: ^{1}H NMR of Bromocyclododecane (**3i**)

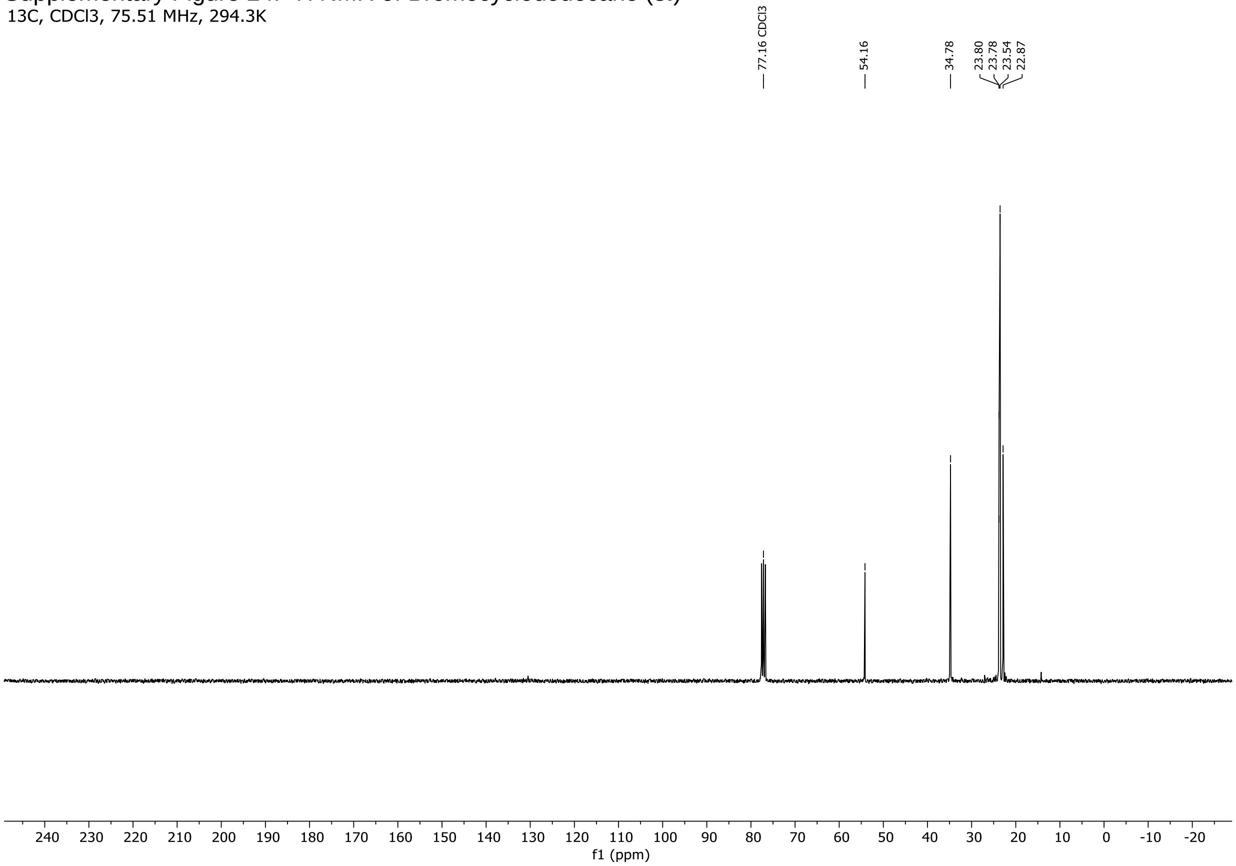

Supplementary Figure 25: ^{13}C NMR of Bromocyclododecane (**3i**)

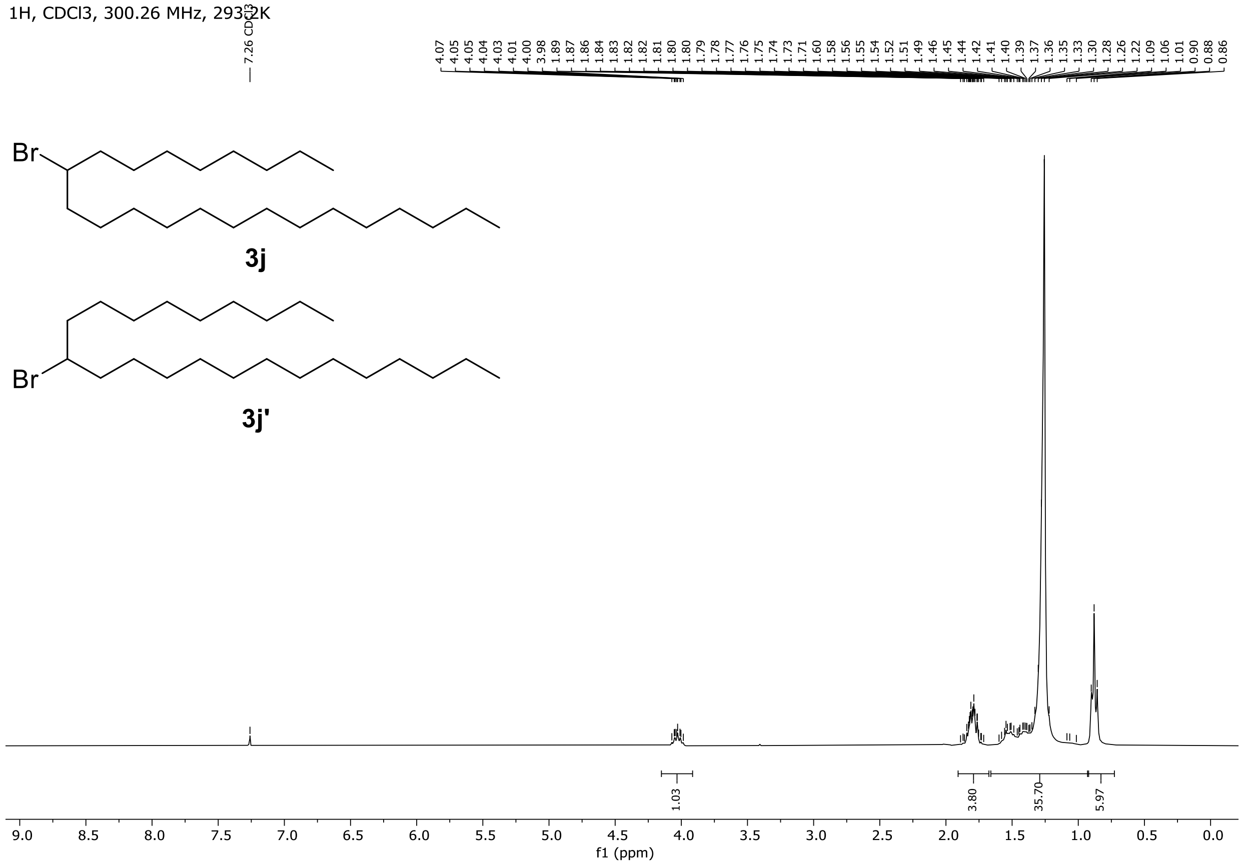

Supplementary Figure 26: ^{1}H NMR of 9-Bromotricosane (**3j**) / 10-Bromotricosan (**3j'**) 1:1

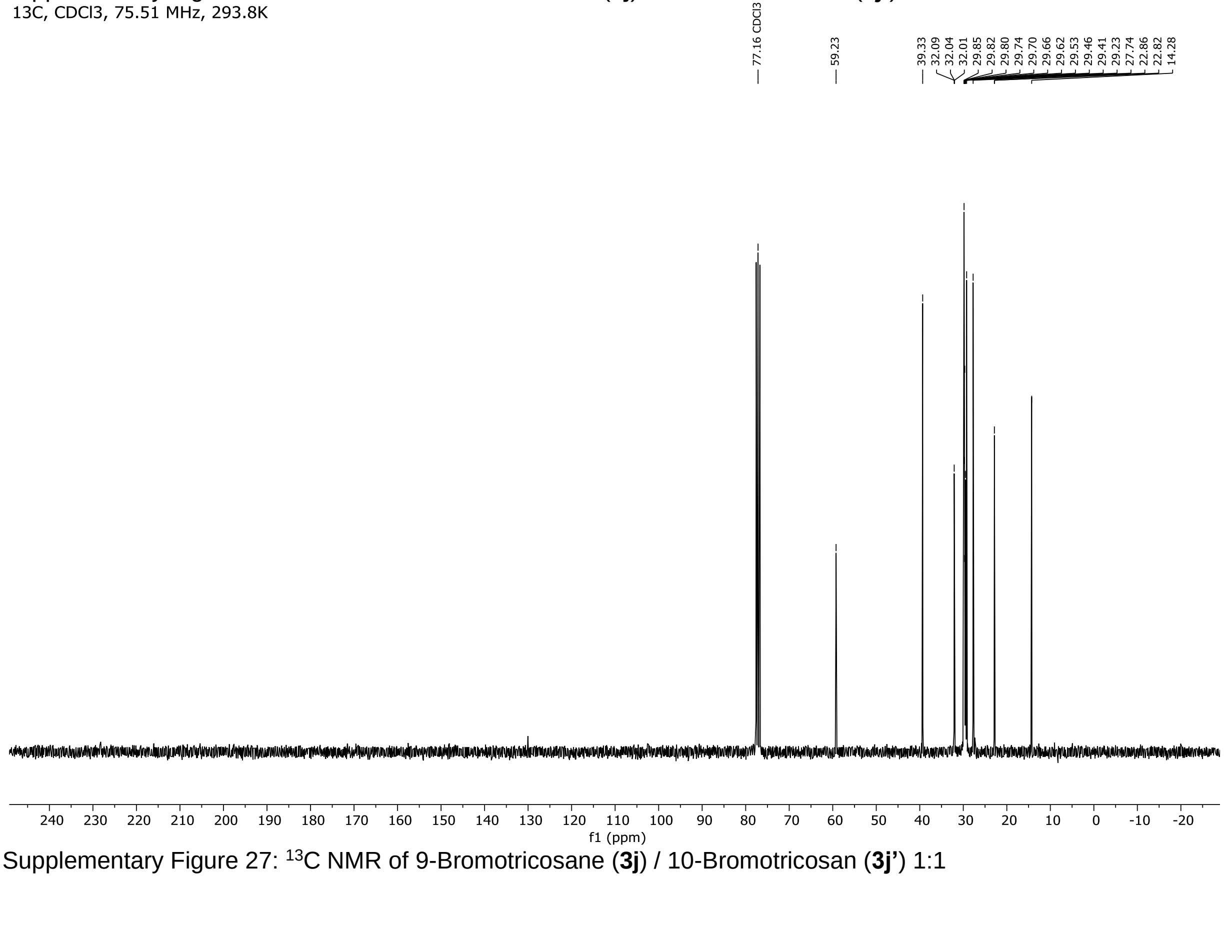

Supplementary Figure 27: ^{13}C NMR of 9-Bromotricosane (**3j**) / 10-Bromotricosan (**3j'**) 1:1

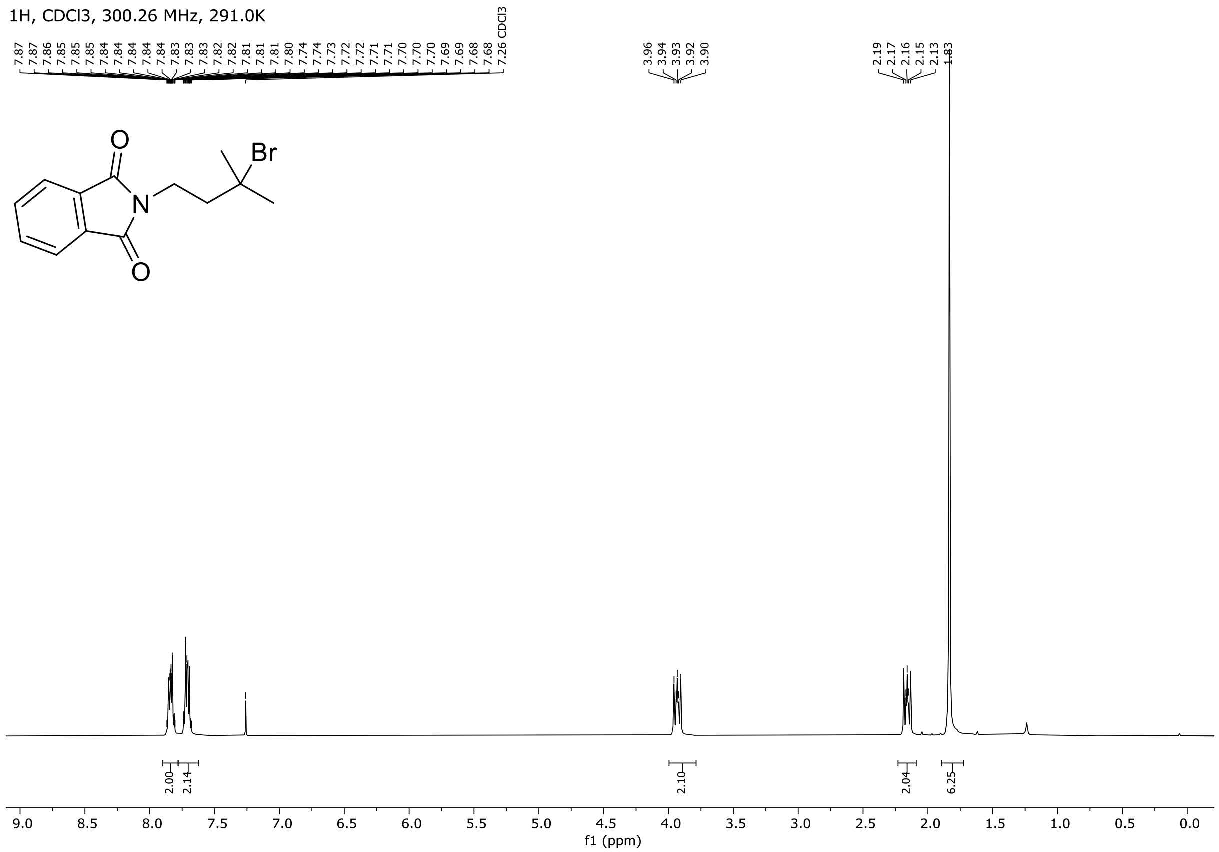

Supplementary Figure 28: ^{1}H NMR of 2-(3-Bromo-3-methylbutyl)isoindoline-1,3-dione (**3k**)

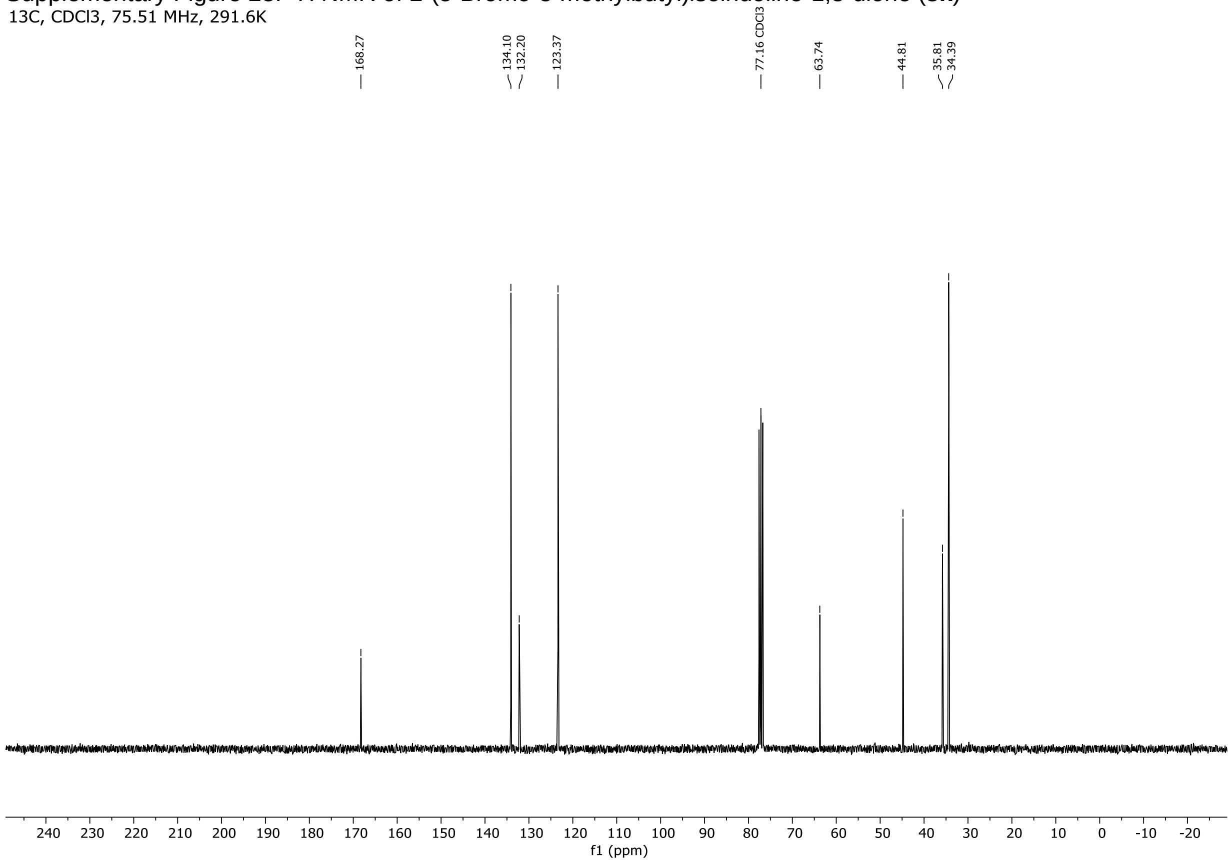

Supplementary Figure 29: ^{13}C NMR of 2-(3-Bromo-3-methylbutyl)isoindoline-1,3-dione (**3k**)

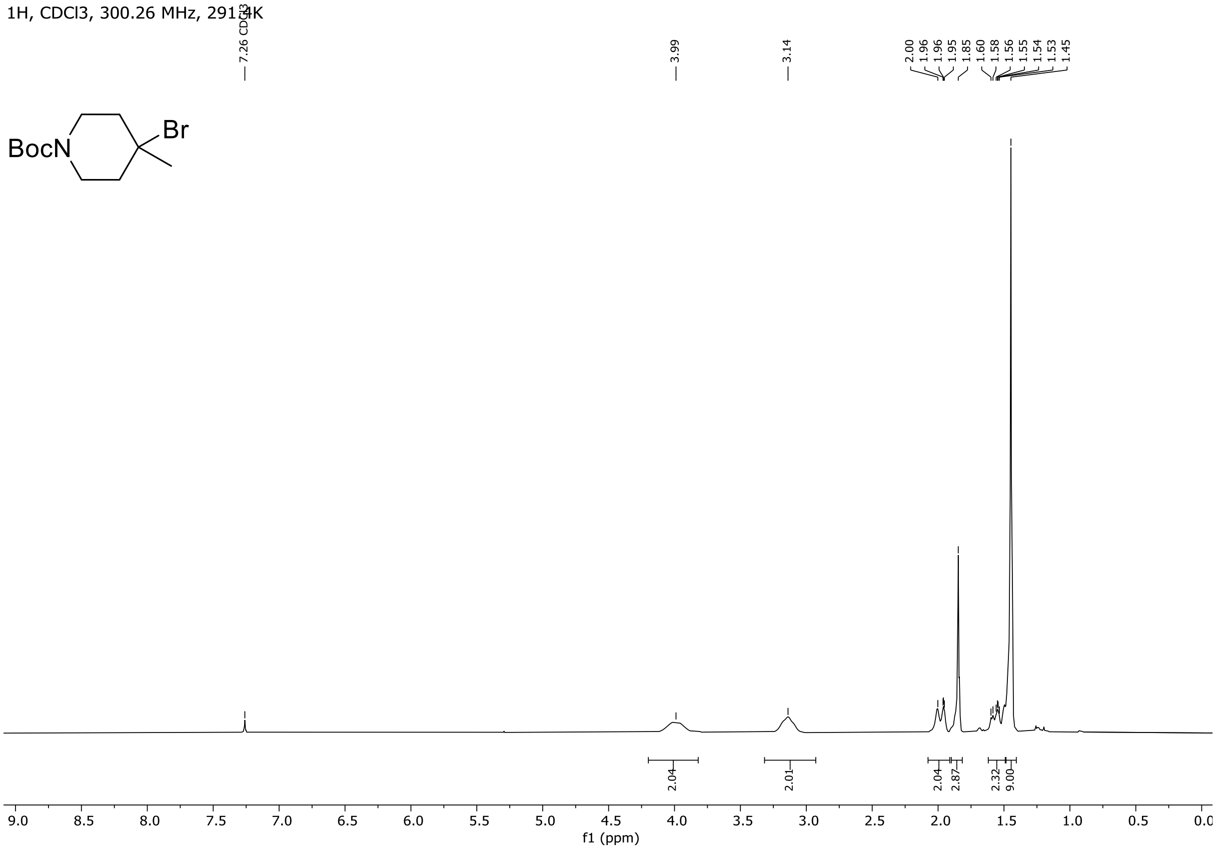

Supplementary Figure 30: ^{1}H NMR of *tert*-Butyl 4-bromo-4-methylpiperidine-1-carboxylate (**3l**)

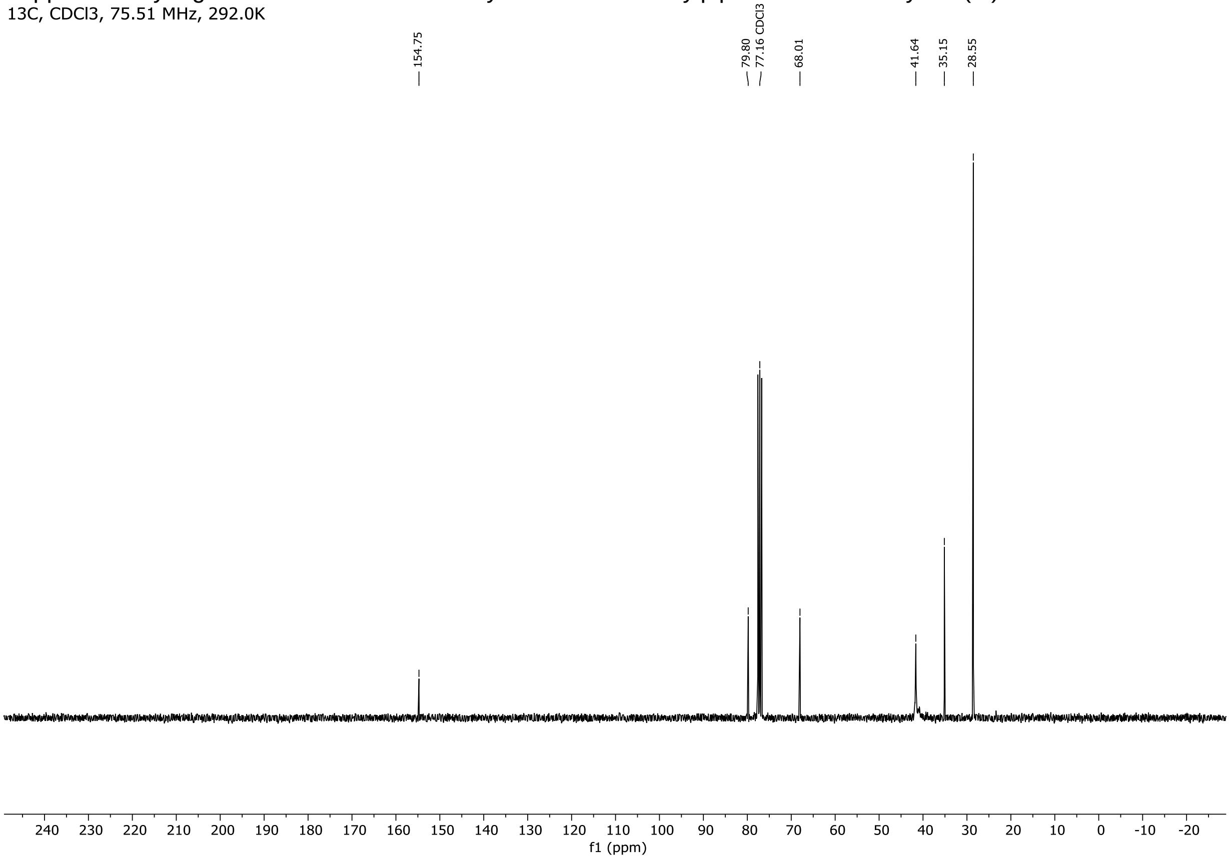

Supplementary Figure 31: ^{13}C NMR of *tert*-Butyl 4-bromo-4-methylpiperidine-1-carboxylate (**3l**)

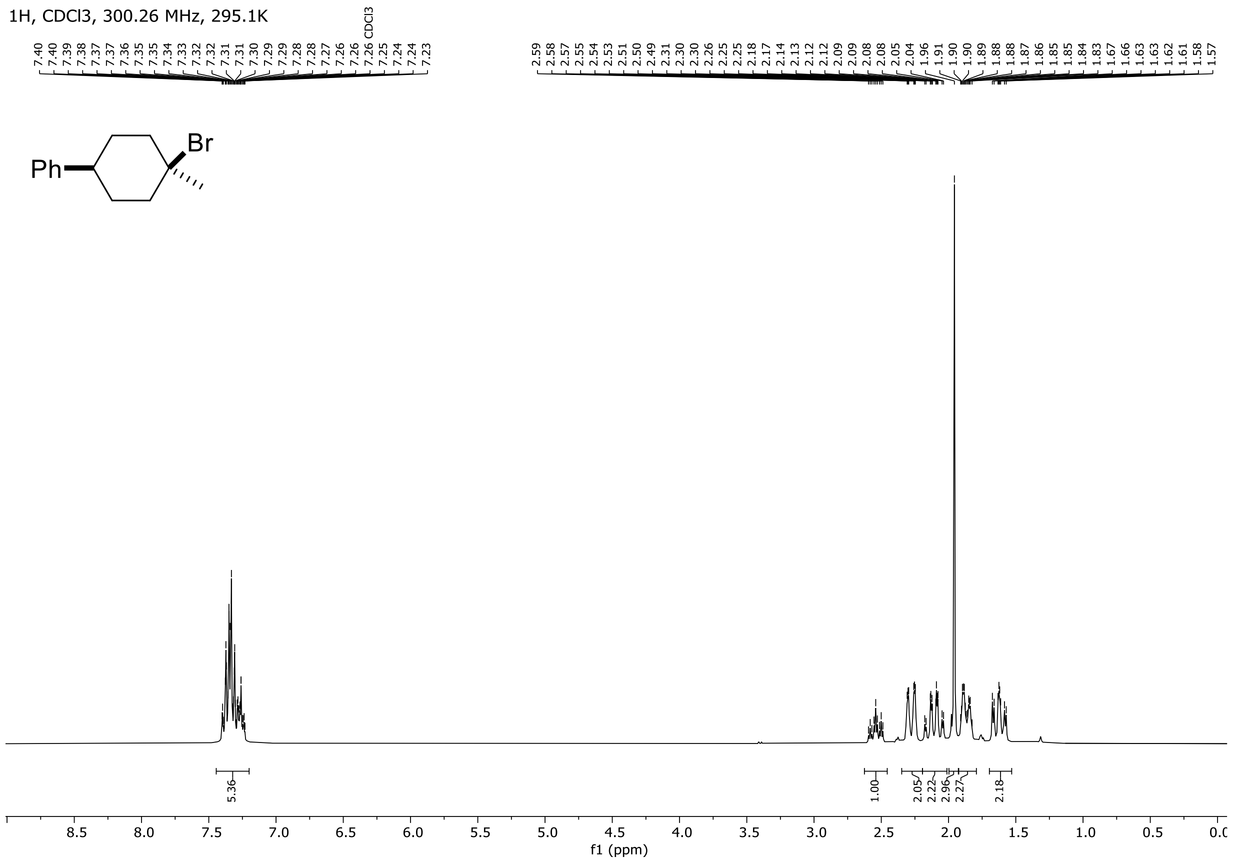

Supplementary Figure 32: ^{1}H NMR of *cis*-4-Bromo-4-methylcyclohexyl)benzene (**3n**)

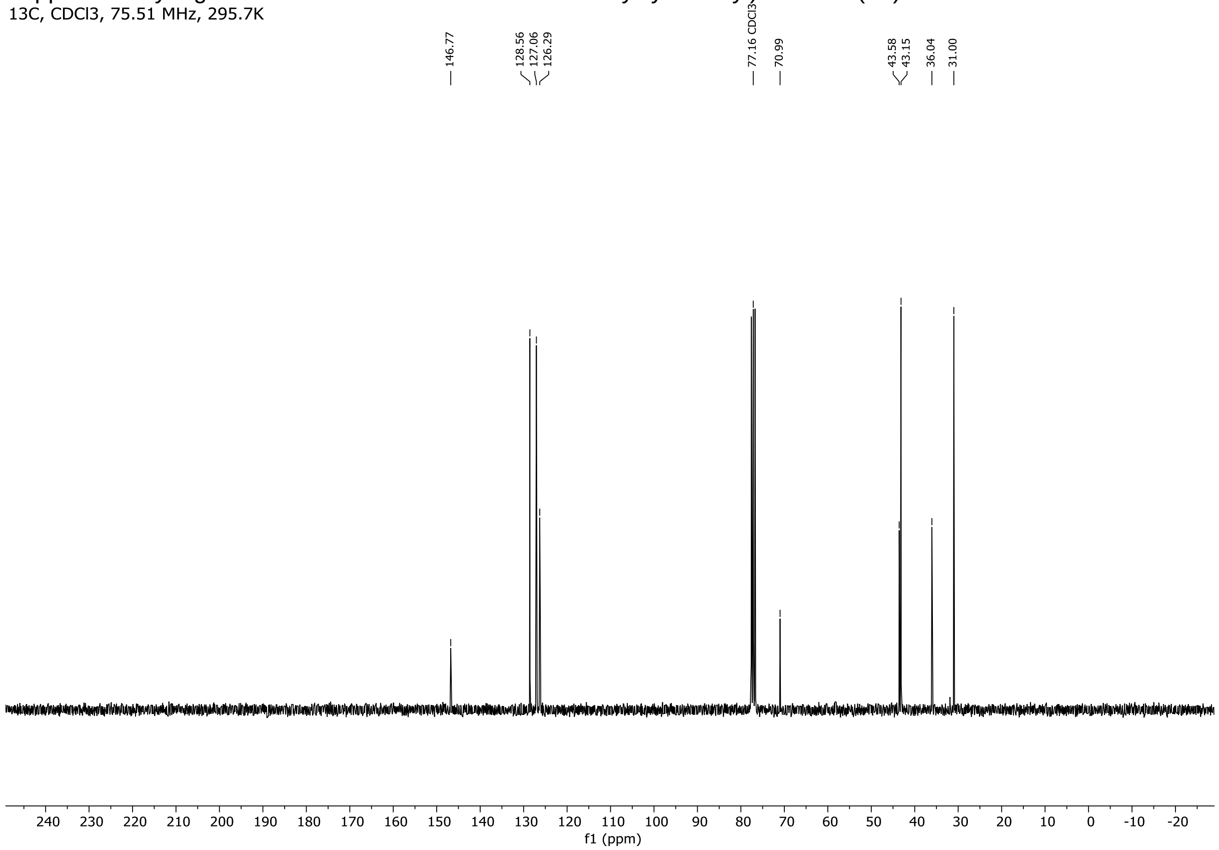

Supplementary Figure 33: ^{13}C NMR of *cis*-4-Bromo-4-methylcyclohexyl)benzene (**3n**)

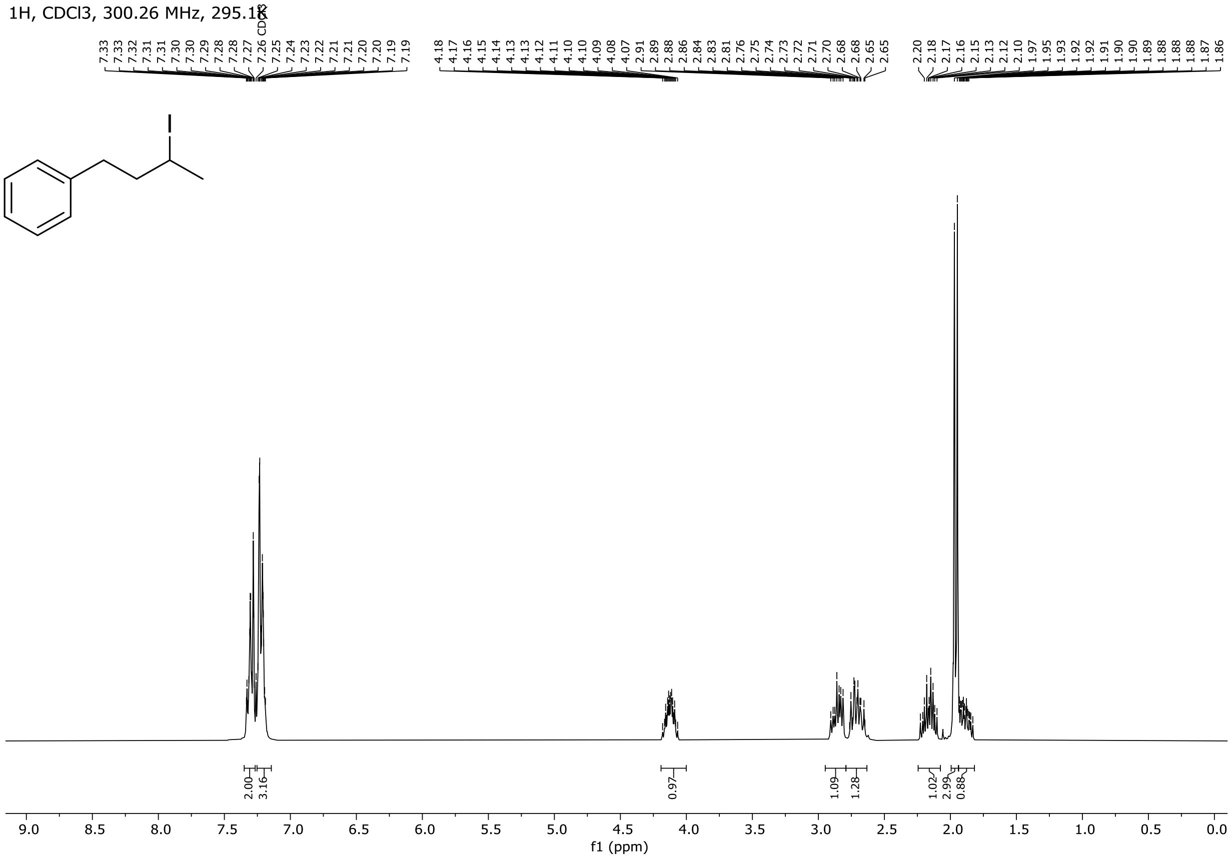

Supplementary Figure 34: ^{1}H NMR of (3-Iodobutyl)benzene (**5a**)

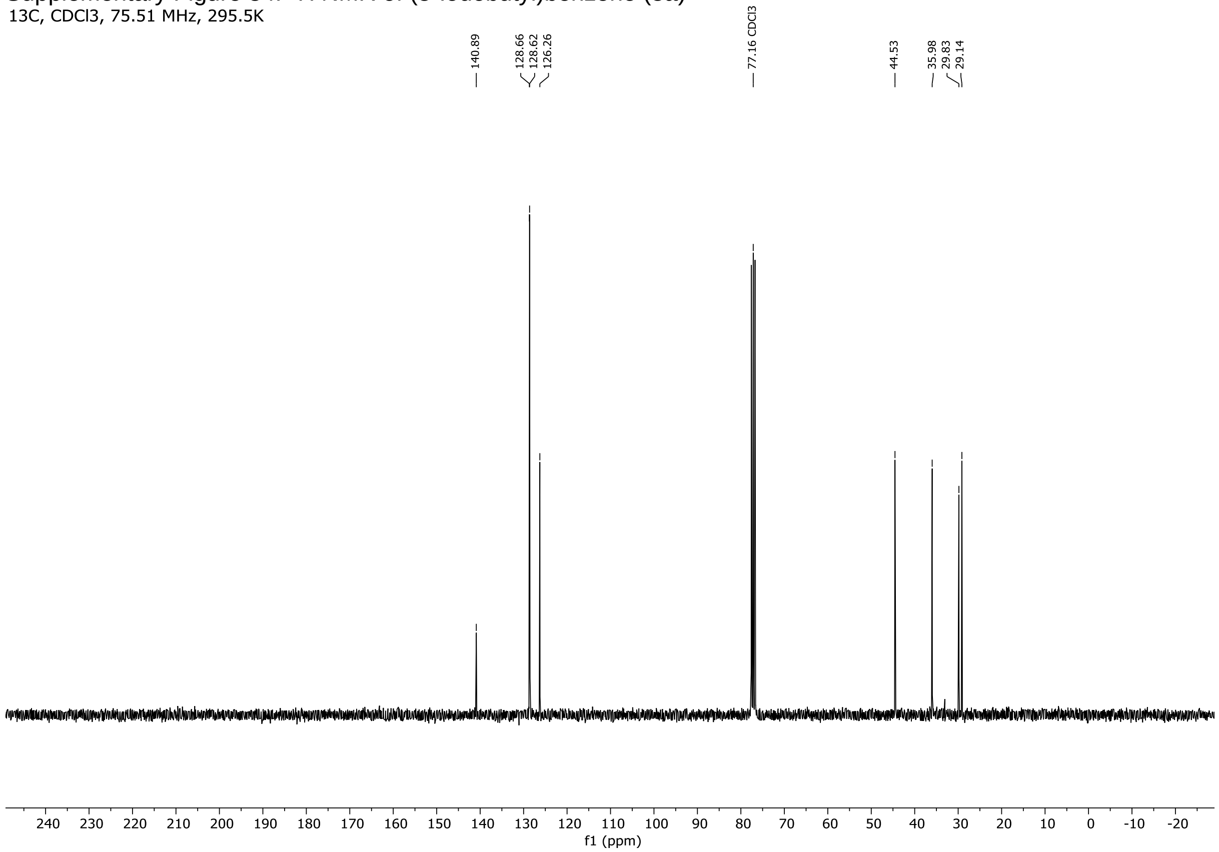

Supplementary Figure 35: ^{13}C NMR of (3-Iodobutyl)benzene (**5a**)

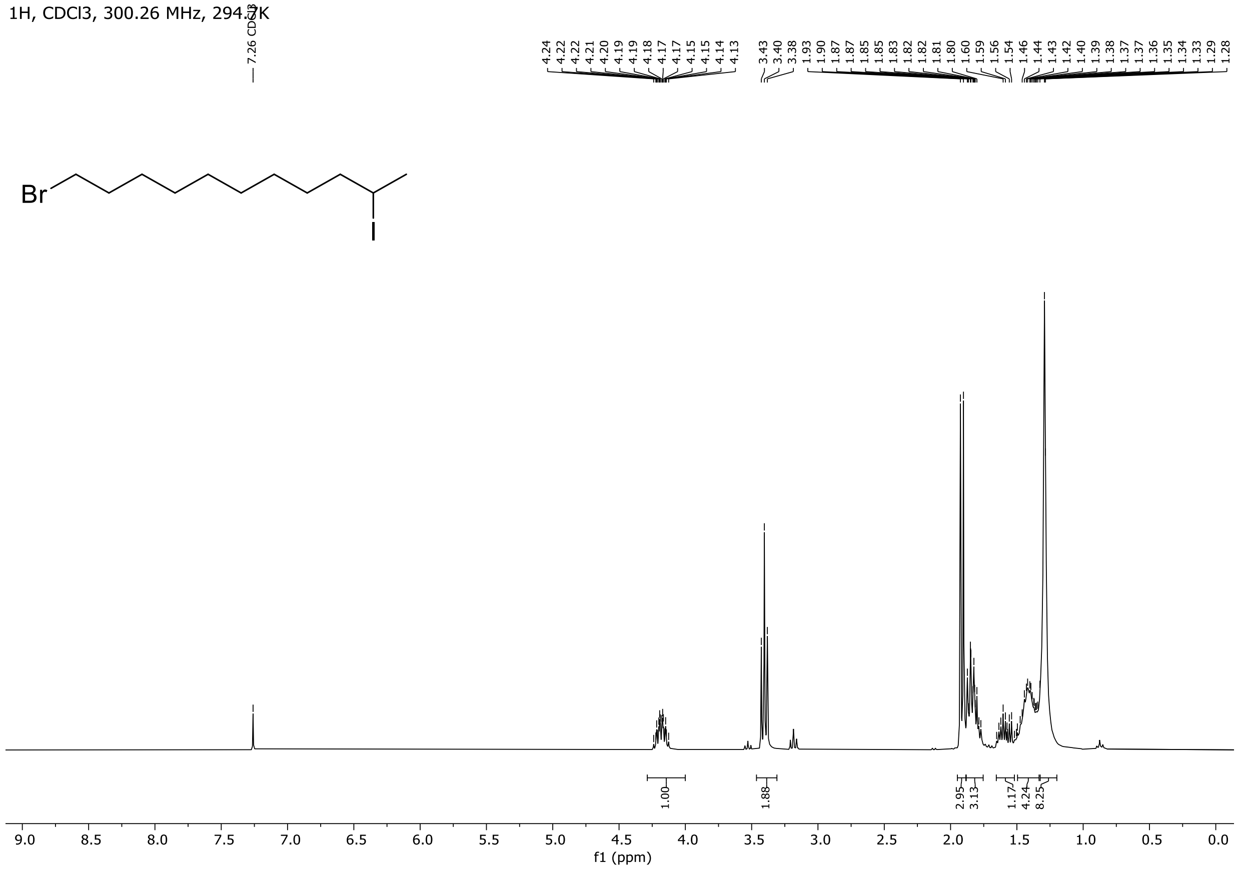

Supplementary Figure 36: ^{1}H NMR of 1-Bromo-10-iodoundecane (**5c**)

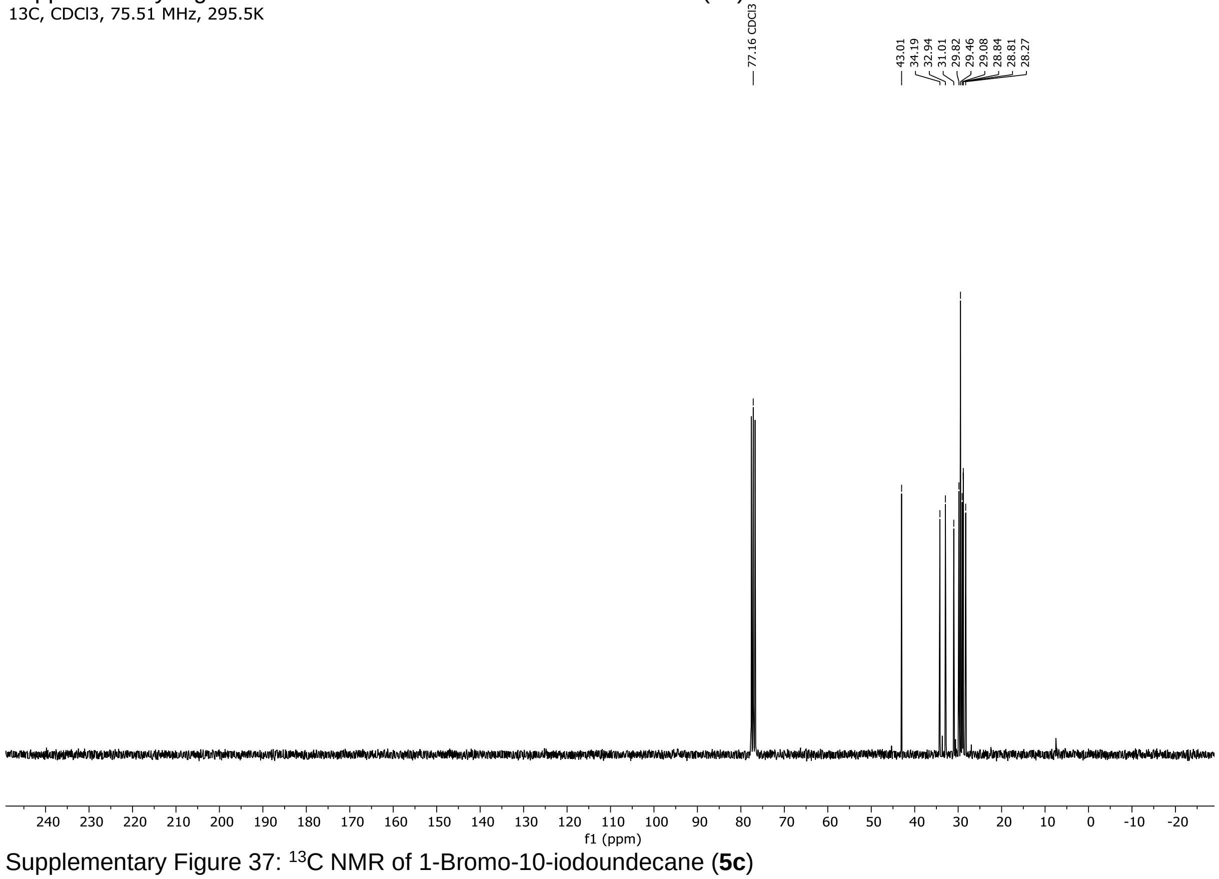

Supplementary Figure 37: ^{13}C NMR of 1-Bromo-10-iodoundecane (**5c**)

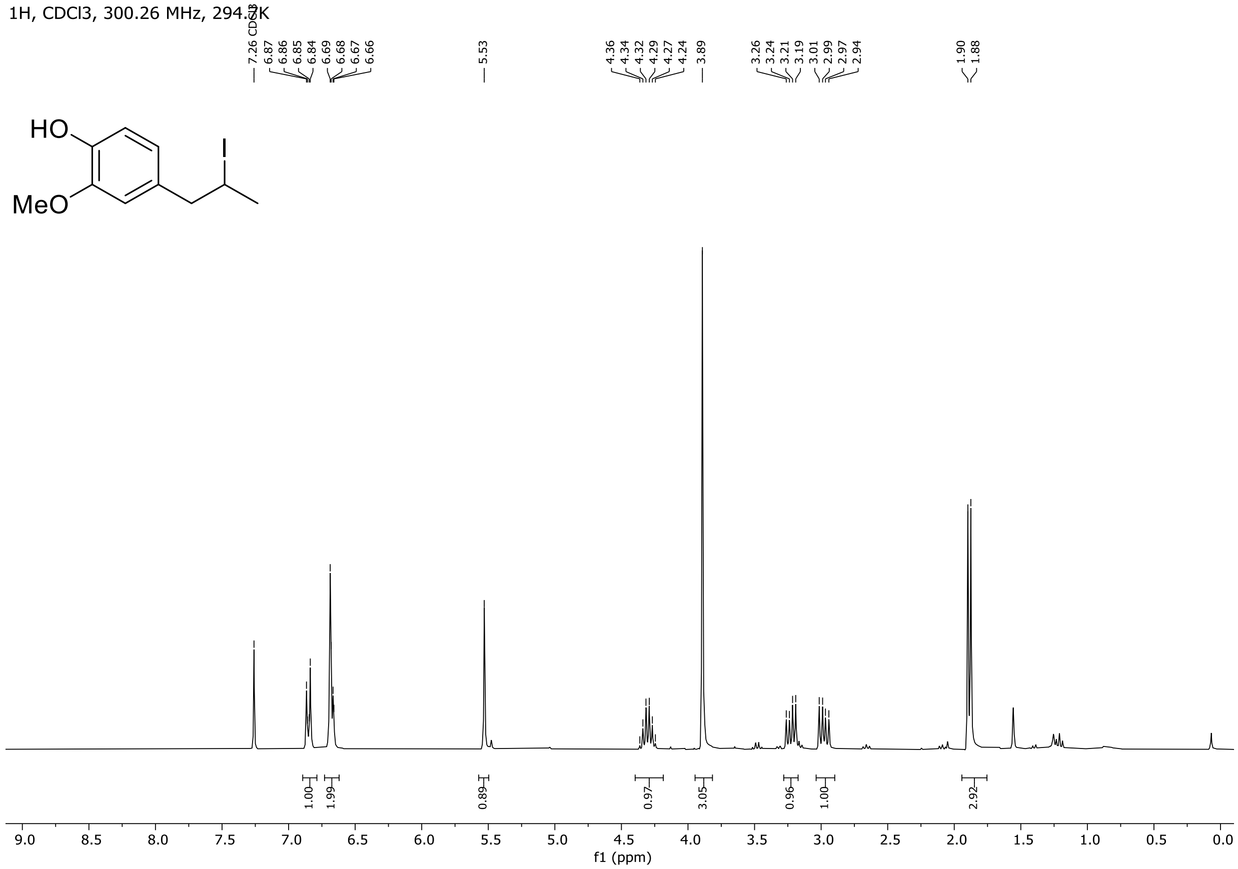

Supplementary Figure 38: ^{1}H NMR of 4-(2-Iodopropyl)-2-methoxyphenol (**5d**)

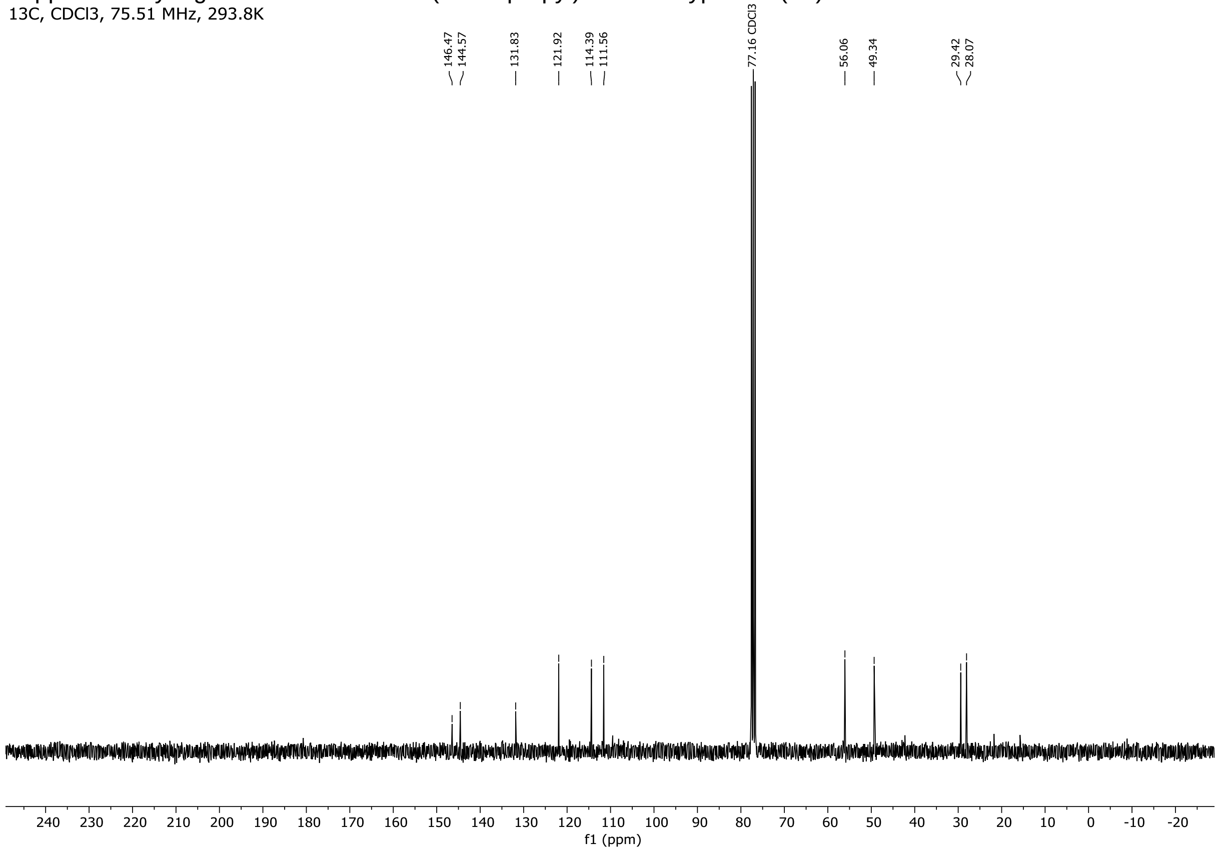

Supplementary Figure 39: ^{13}C NMR of 4-(2-Iodopropyl)-2-methoxyphenol (**5d**)

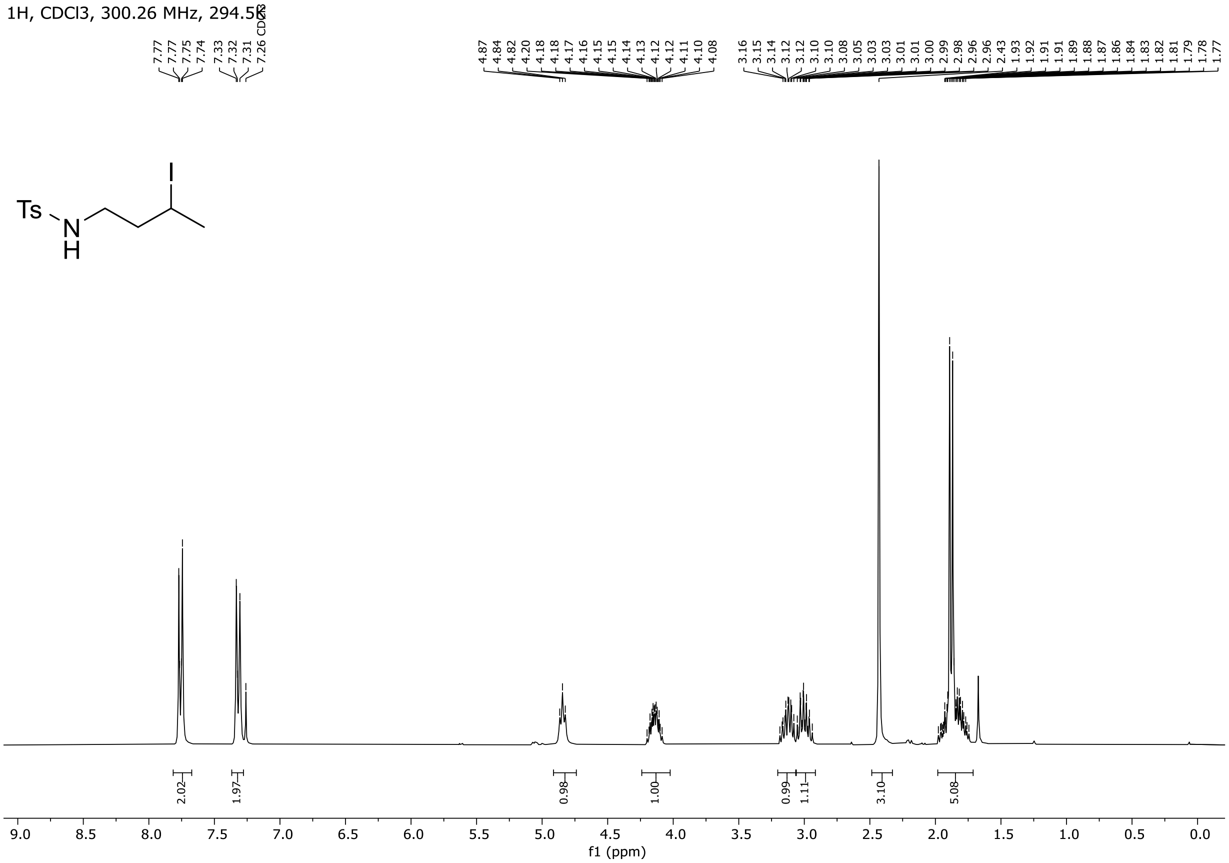

Supplementary Figure 40: ^{1}H NMR of *N*-(3-Iodobutyl)-4-methylbenzenesulfonamide (**5e**)

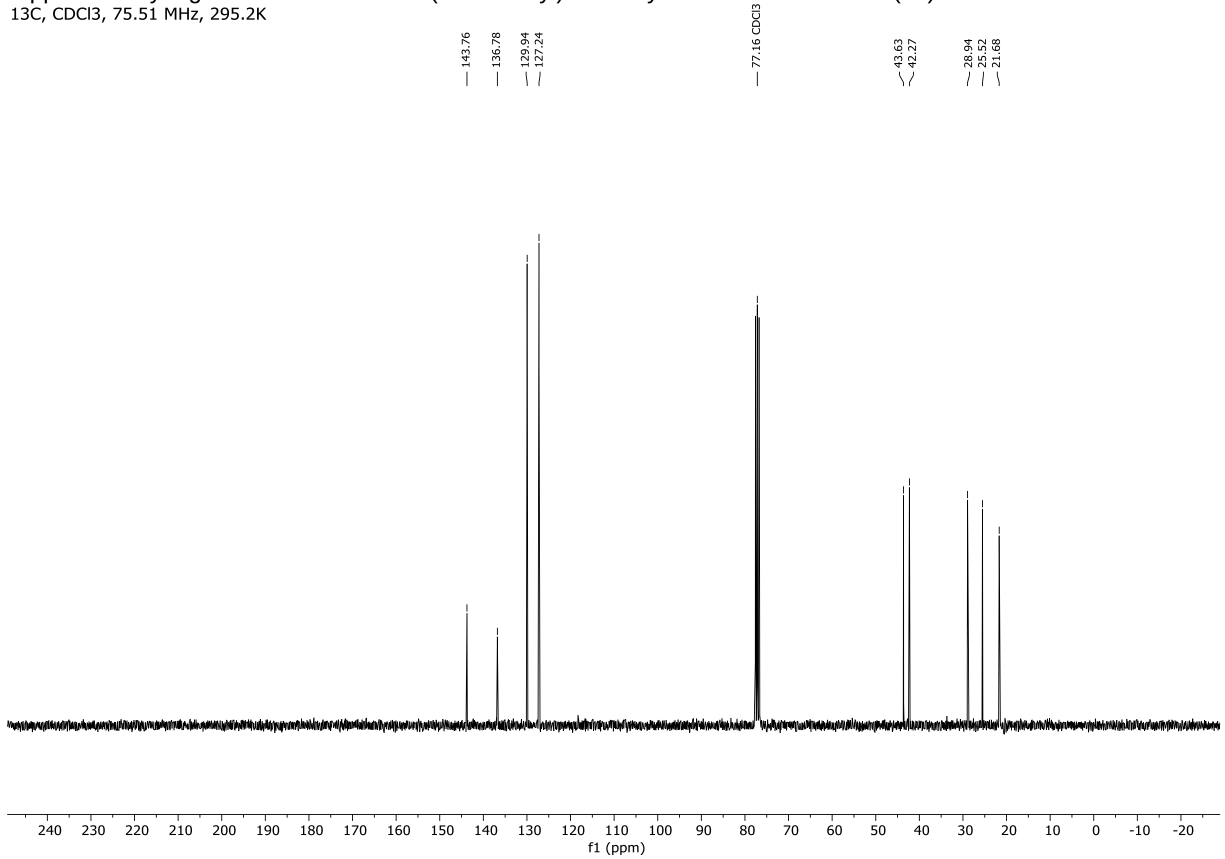

Supplementary Figure 41: ^{13}C NMR of *N*-(3-Iodobutyl)-4-methylbenzenesulfonamide (**5e**)

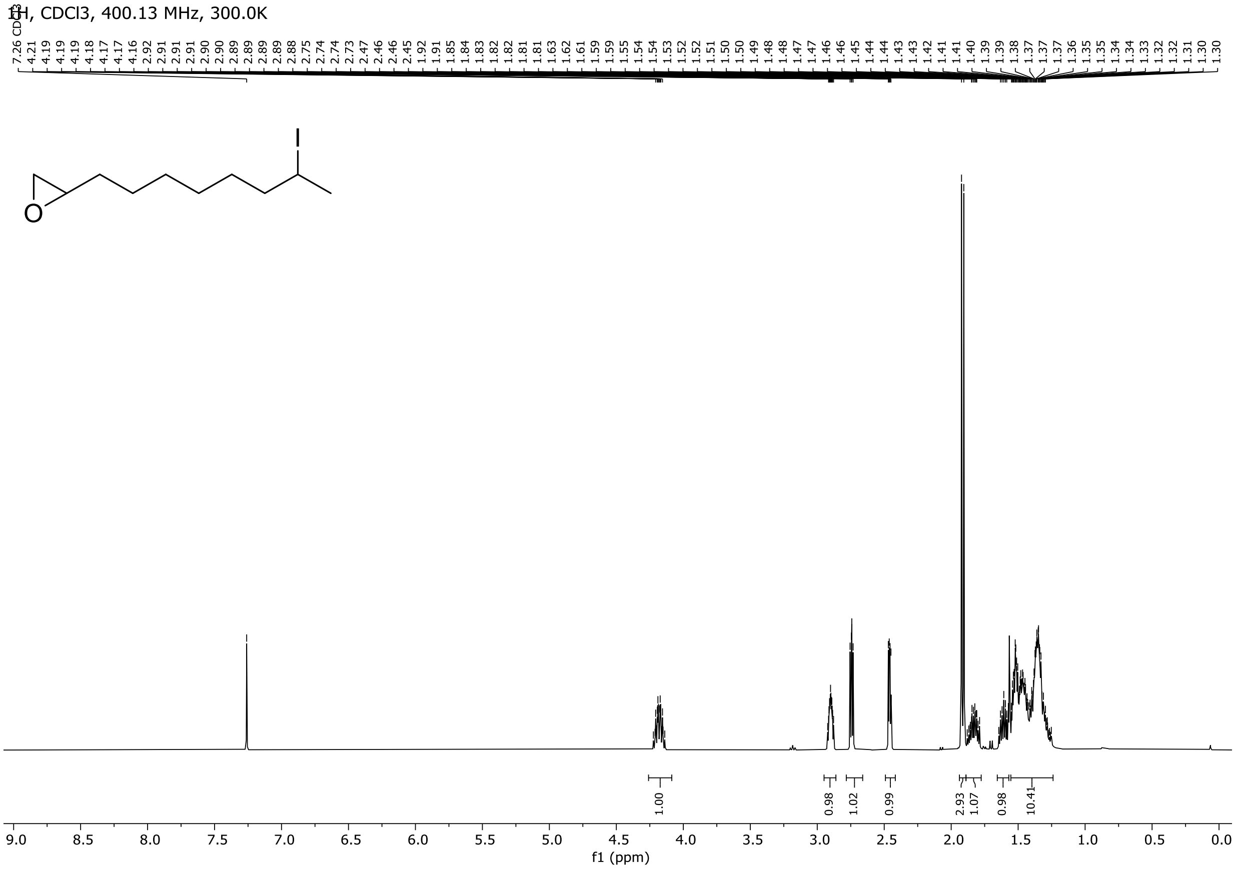

Supplementary Figure 42: ^{1}H NMR of 2-(7-Iodooctyl)oxirane (**5g**)

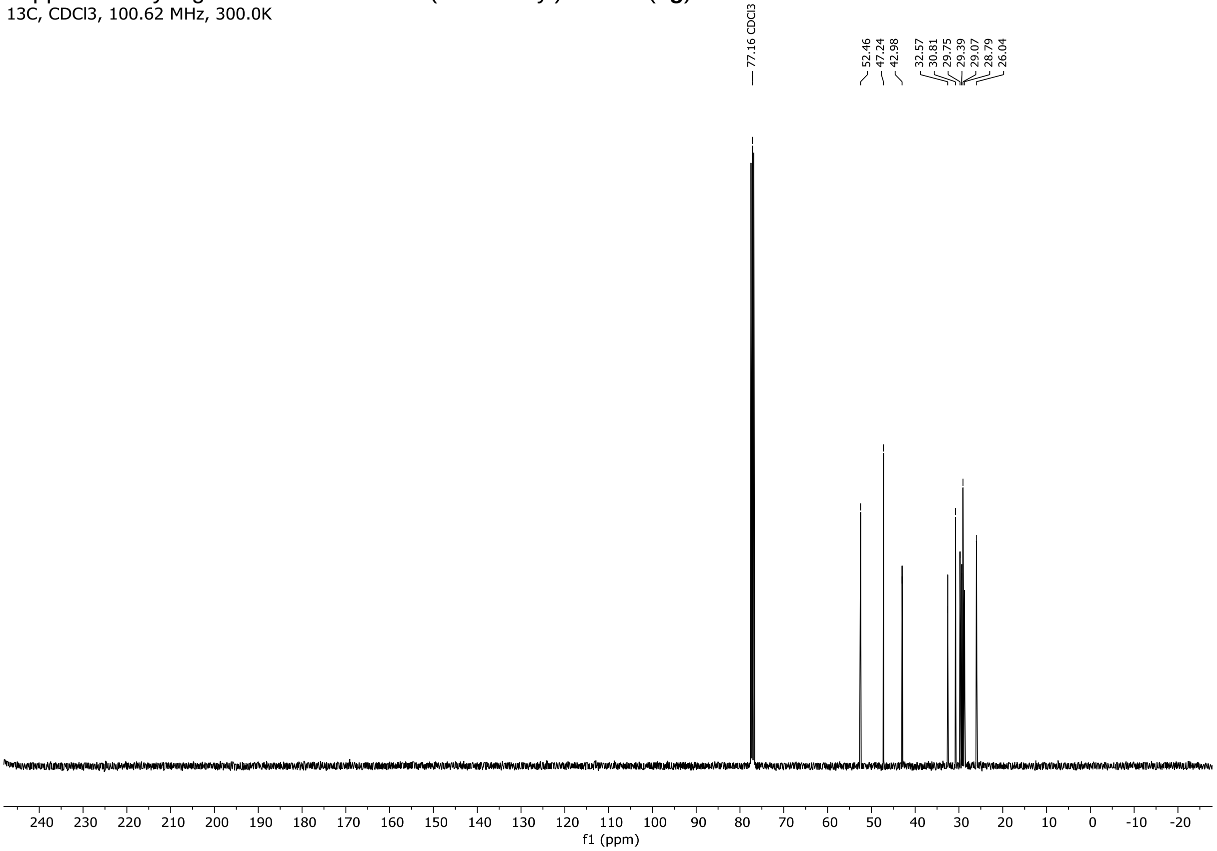

Supplementary Figure 43: ^{13}C NMR of 2-(7-Iodooctyl)oxirane (**5g**)

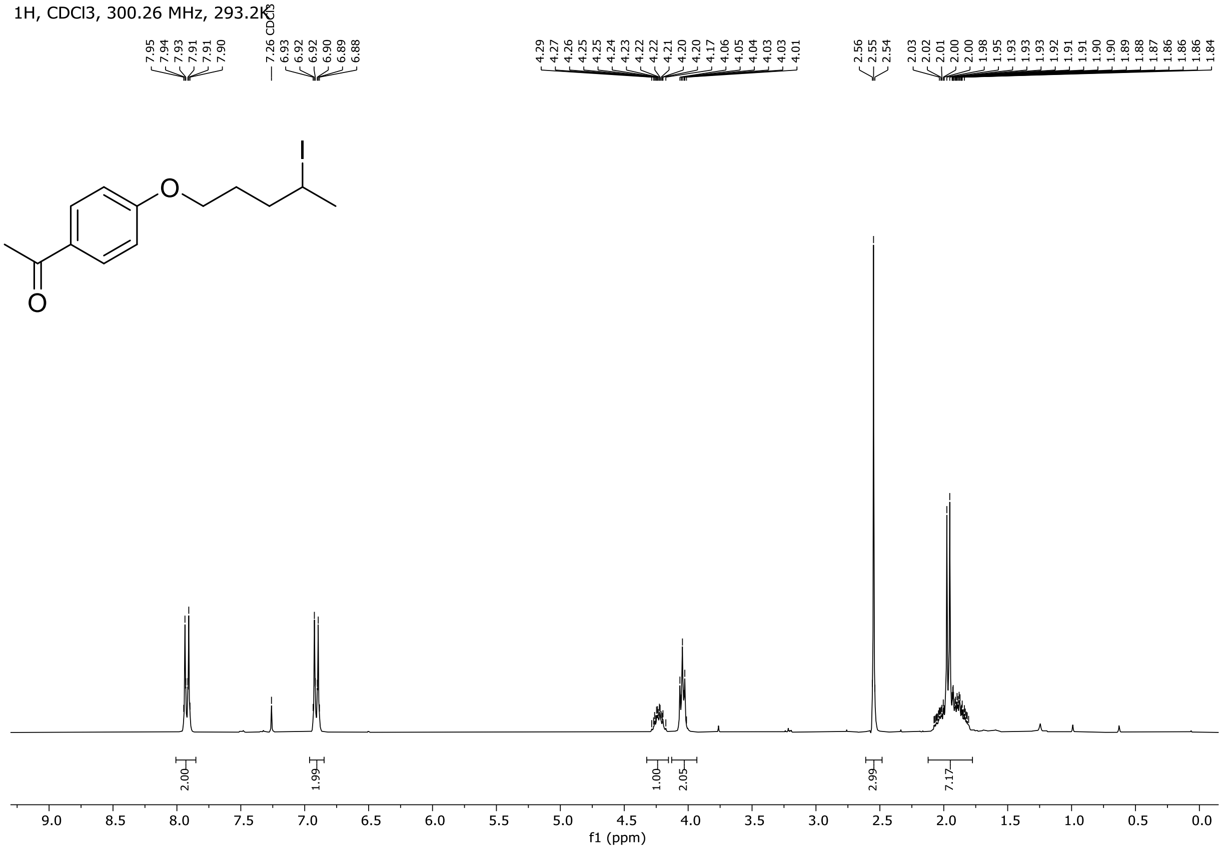

Supplementary Figure 44: ^{1}H NMR of 1-(4-((4-Iodopentyl)oxy)phenyl)ethan-1-one (**5h**)

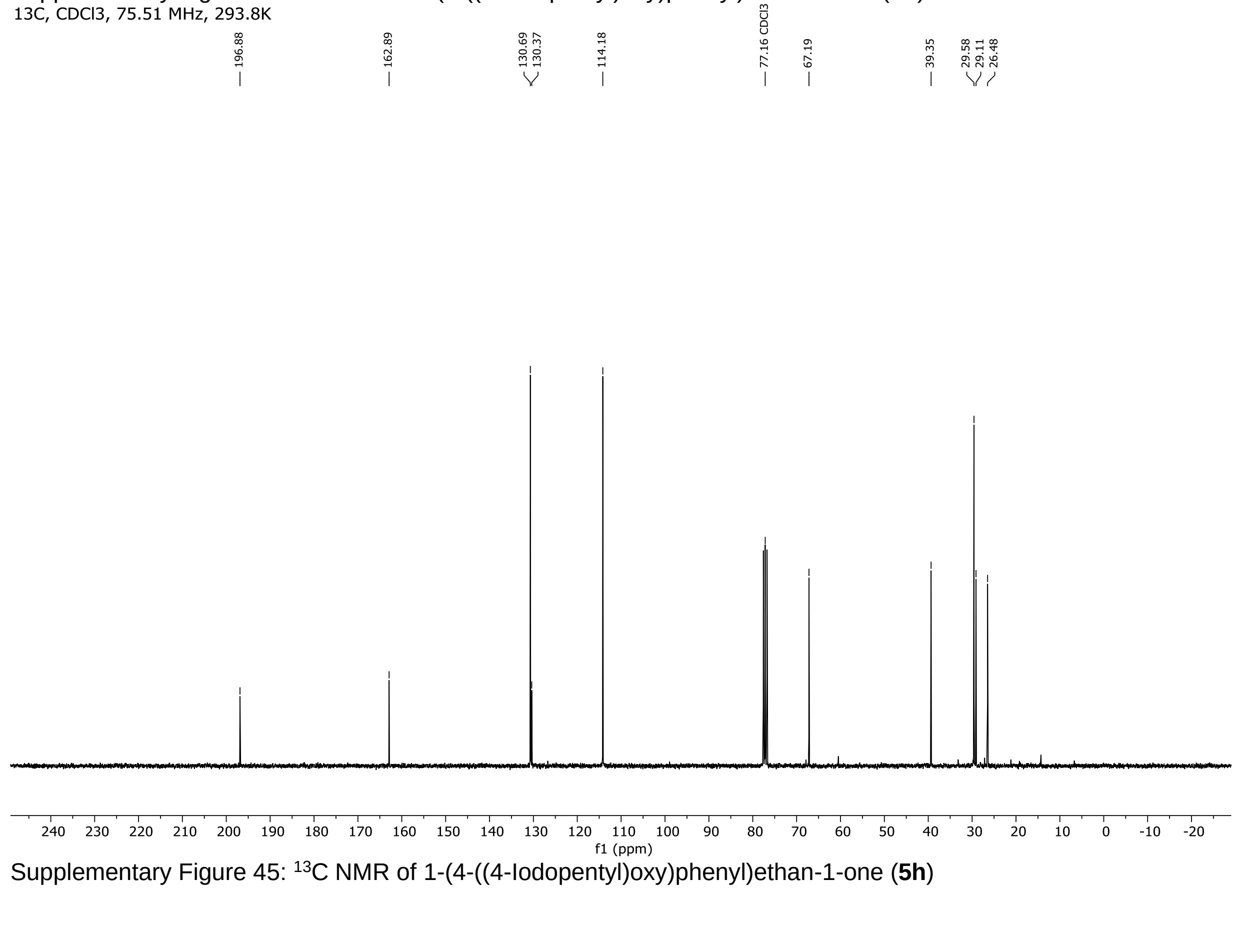

Supplementary Figure 45: ^{13}C NMR of 1-(4-((4-Iodopentyl)oxy)phenyl)ethan-1-one (**5h**)

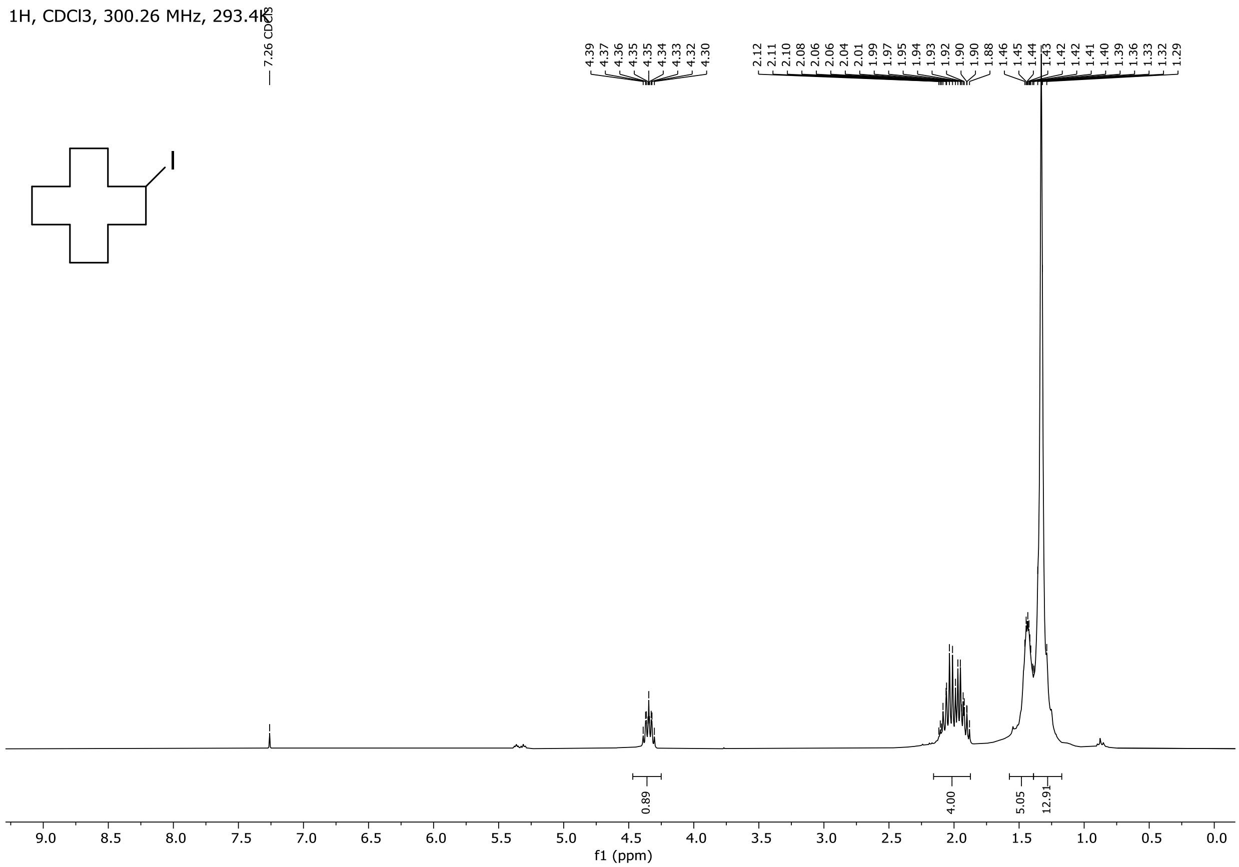

Supplementary Figure 46: ^{1}H NMR of Iodocyclododecane (**5i**)

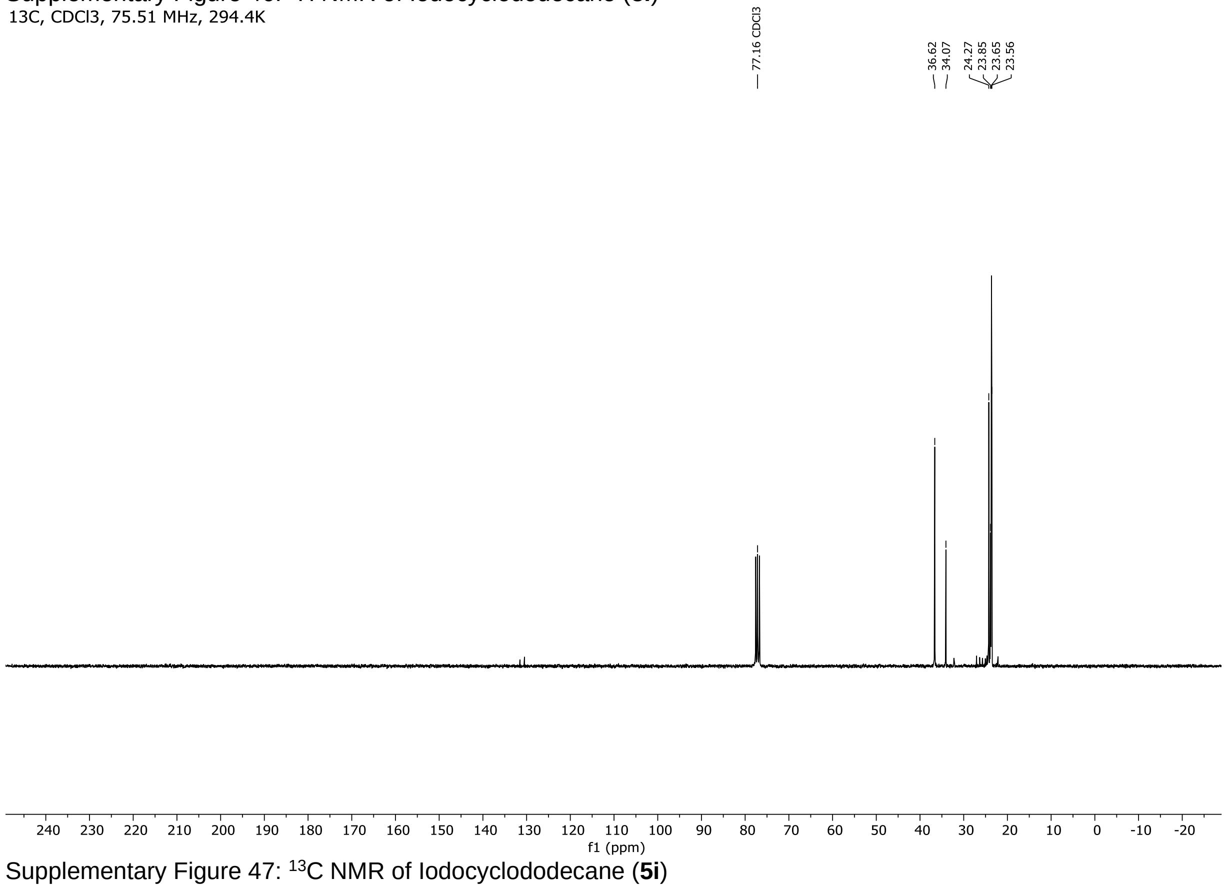

Supplementary Figure 47: ^{13}C NMR of Iodocyclododecane (**5i**)

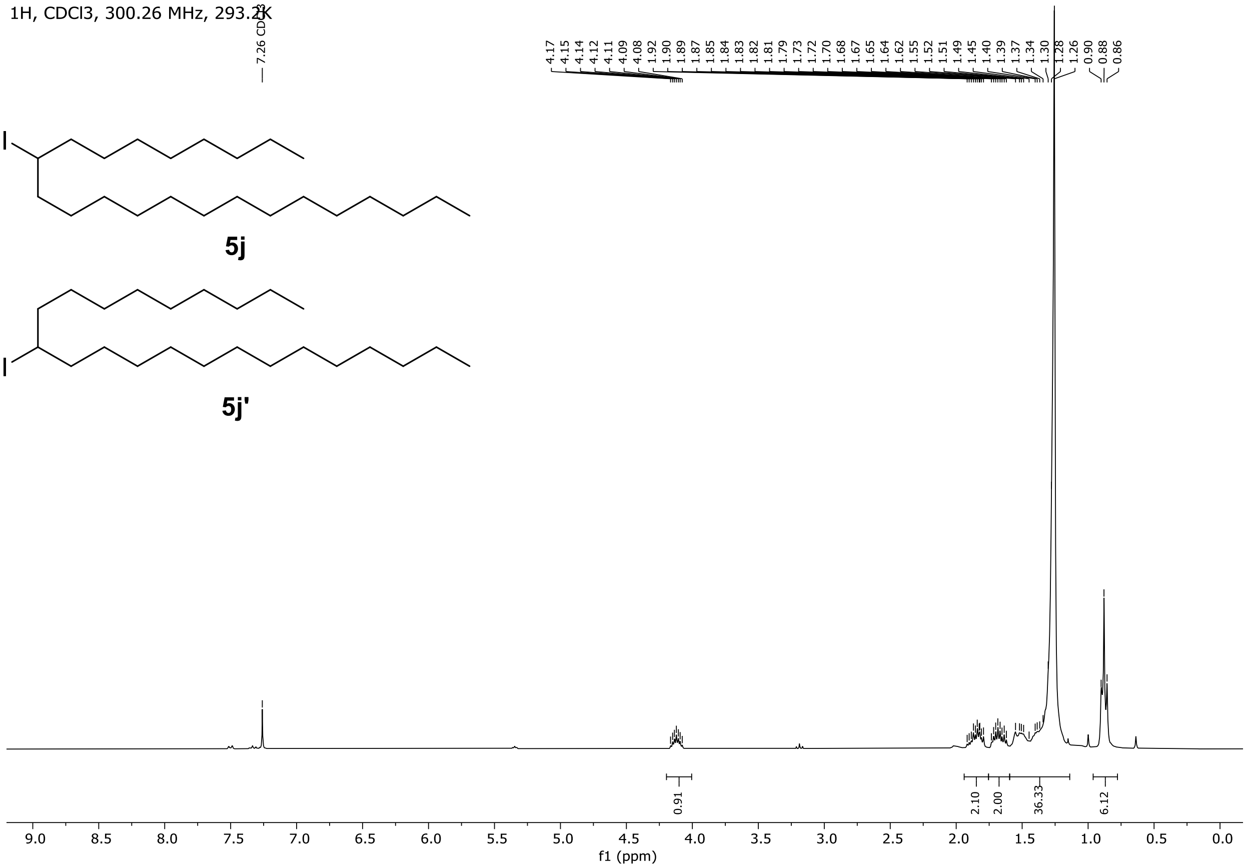

Supplementary Figure 48: ^{1}H NMR of 9-Iodotricosane (**5j**) / 10-Iodotricosane (**5j'**) 1:1

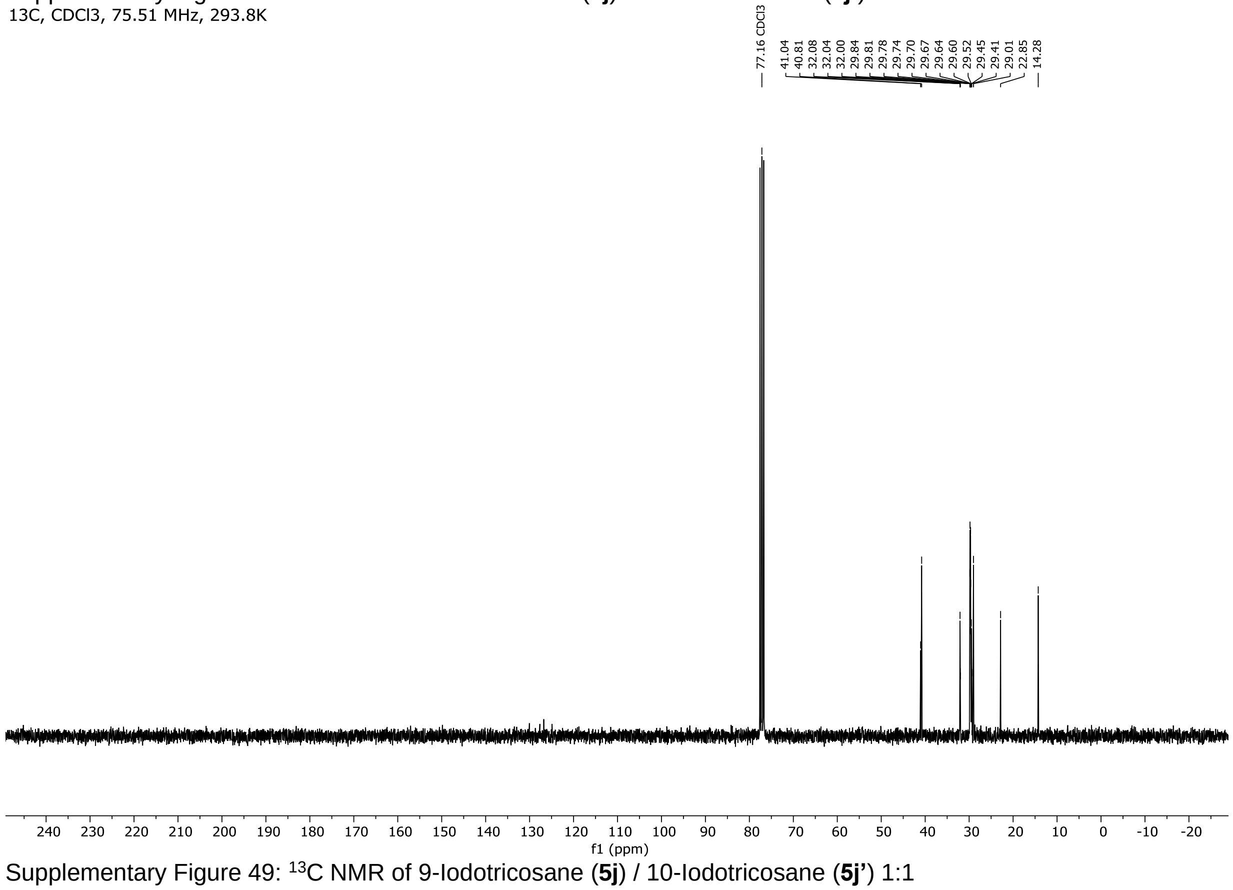

Supplementary Figure 49: ^{13}C NMR of 9-Iodotricosane (**5j**) / 10-Iodotricosane (**5j'**) 1:1

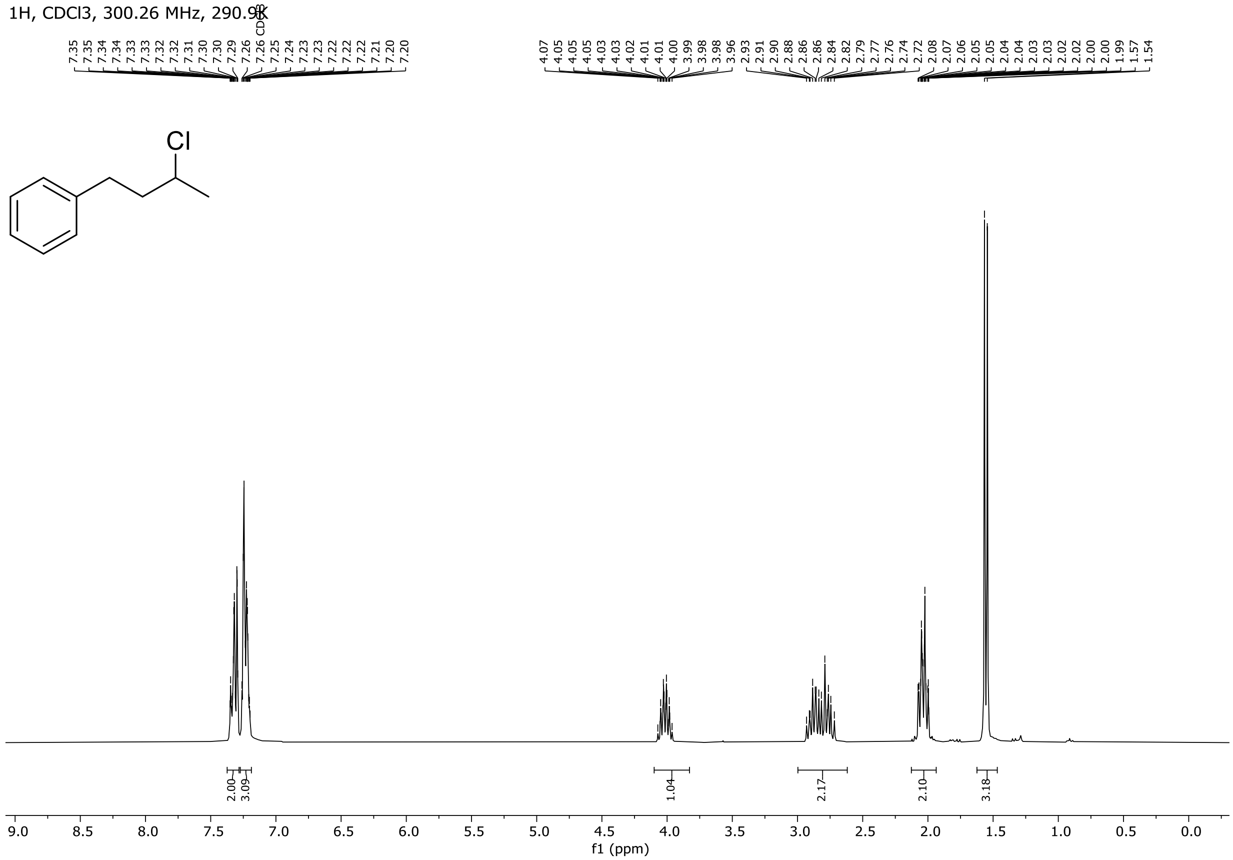

Supplementary Figure 50: ^{1}H NMR of (3-Chlorobutyl)benzene (**7a**)

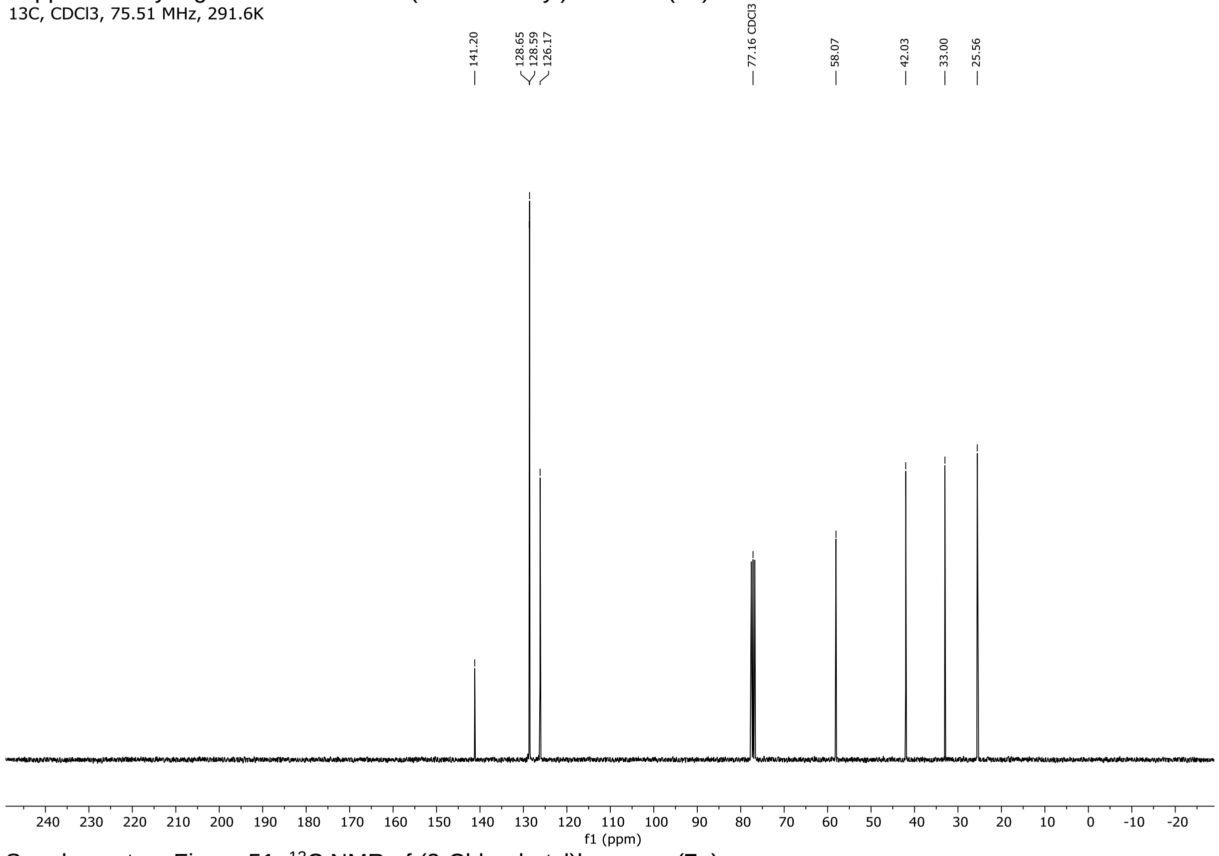

Supplementary Figure 51: ^{13}C NMR of (3-Chlorobutyl)benzene (**7a**)

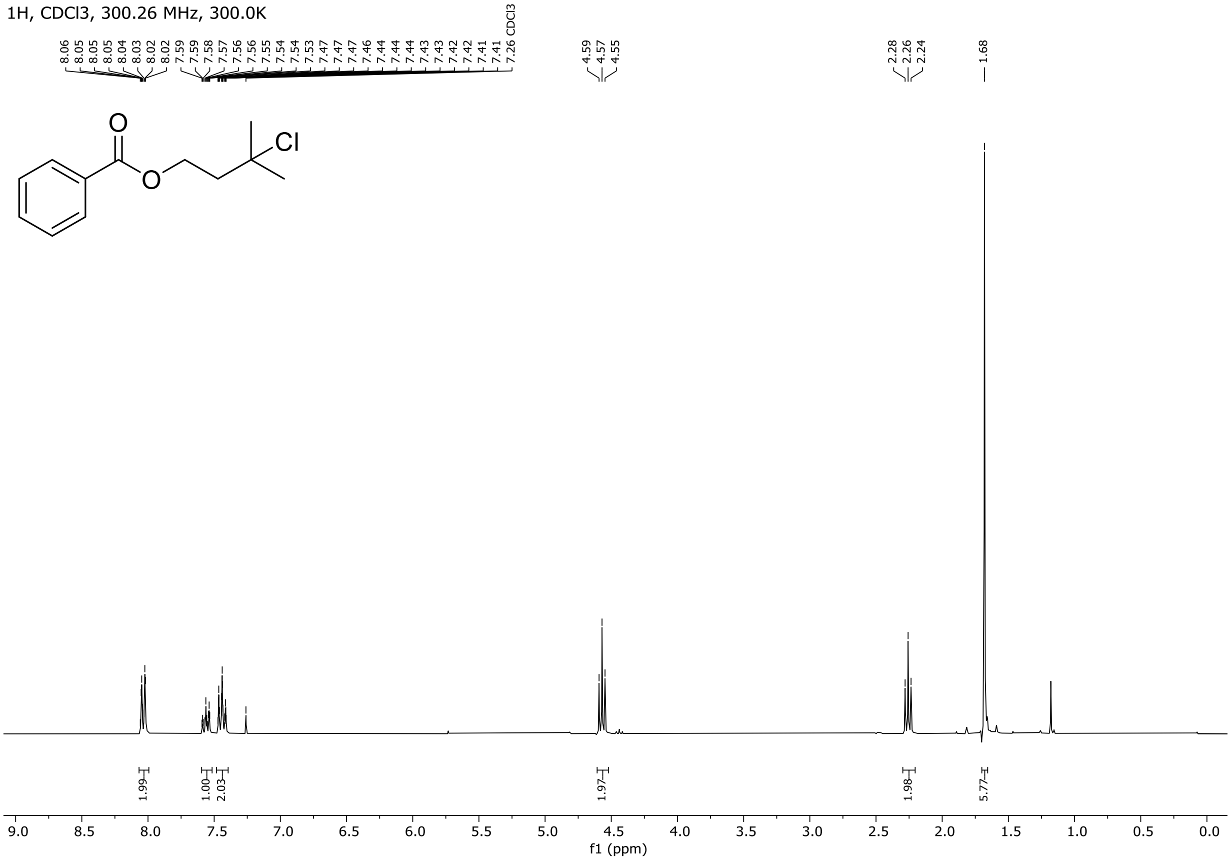

Supplementary Figure 52: ^{1}H NMR of 3-Chloro-3-methylbutyl benzoate (**7b**)

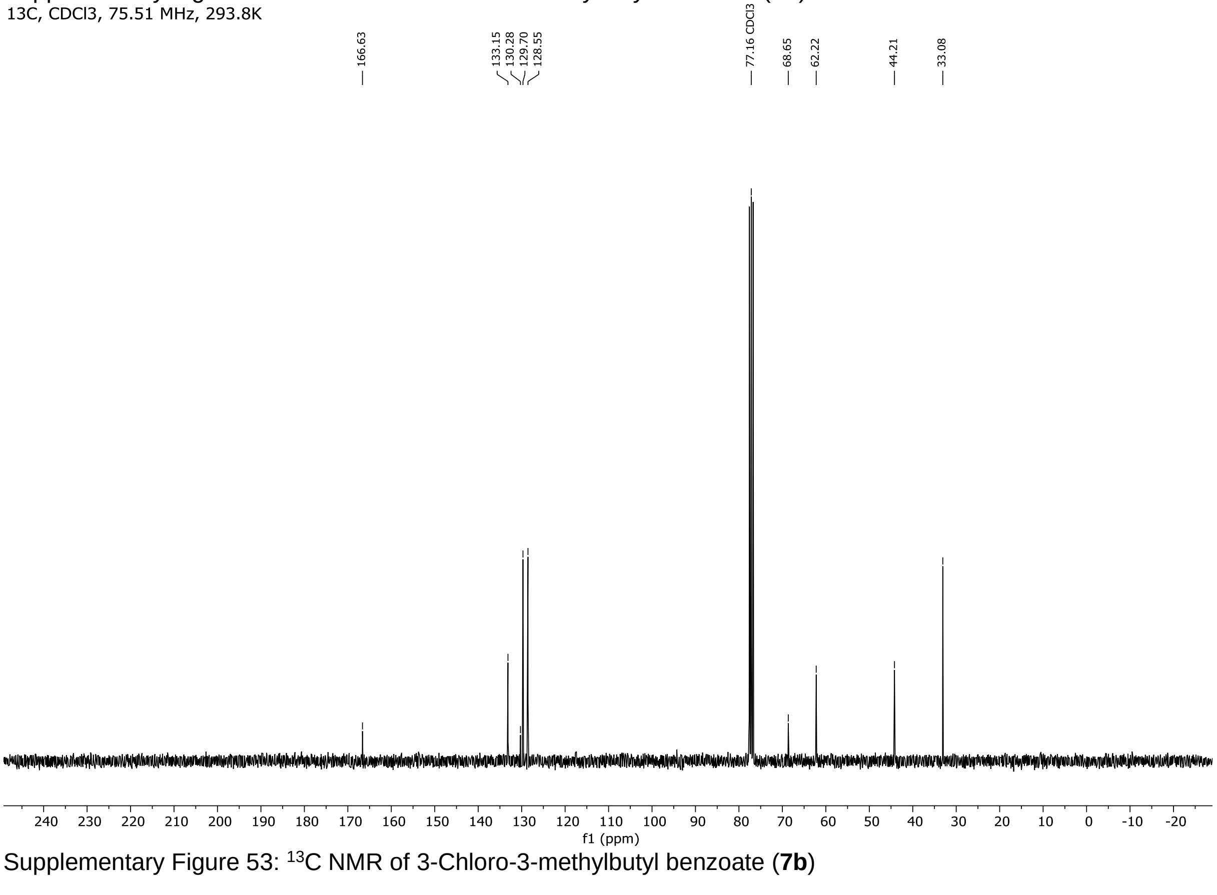

Supplementary Figure 53: ^{13}C NMR of 3-Chloro-3-methylbutyl benzoate (**7b**)

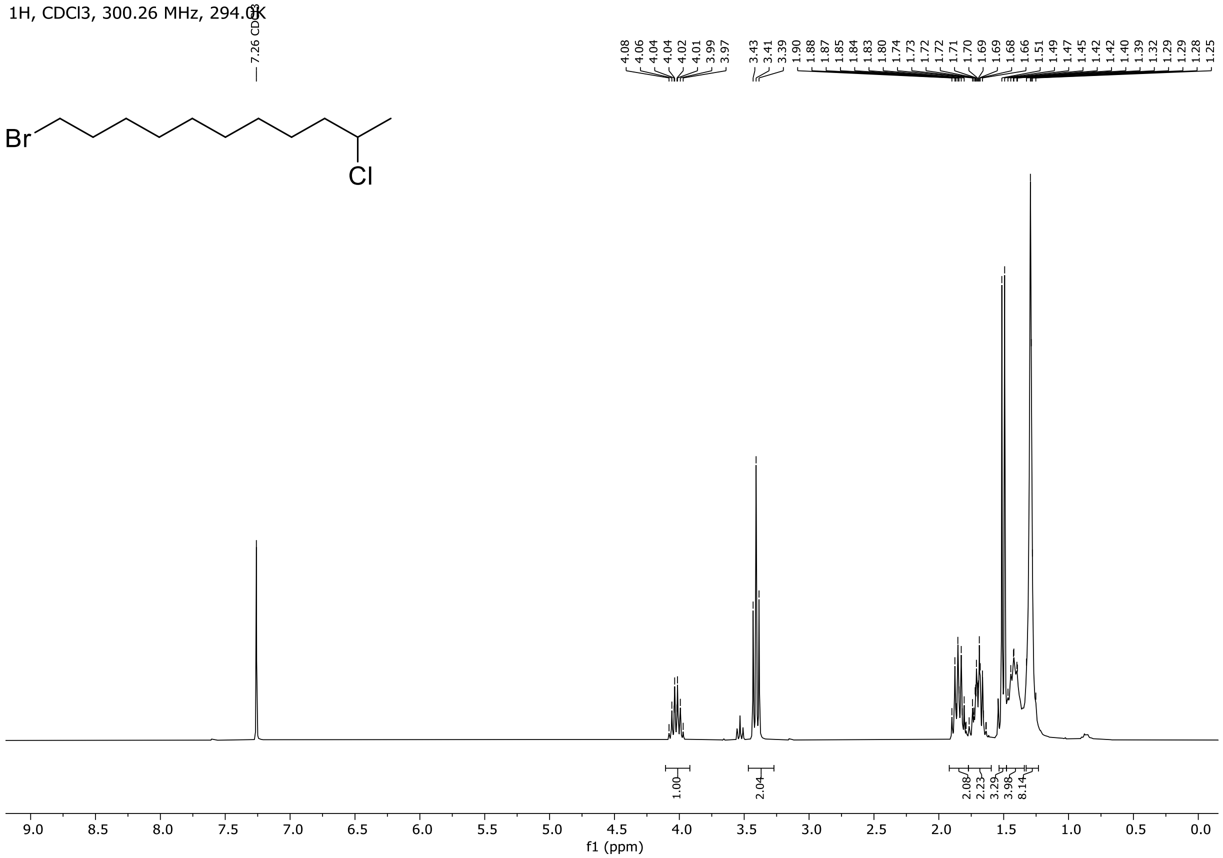

Supplementary Figure 54: ^{1}H NMR of 1-Bromo-10-chloroundecane (**7c**)

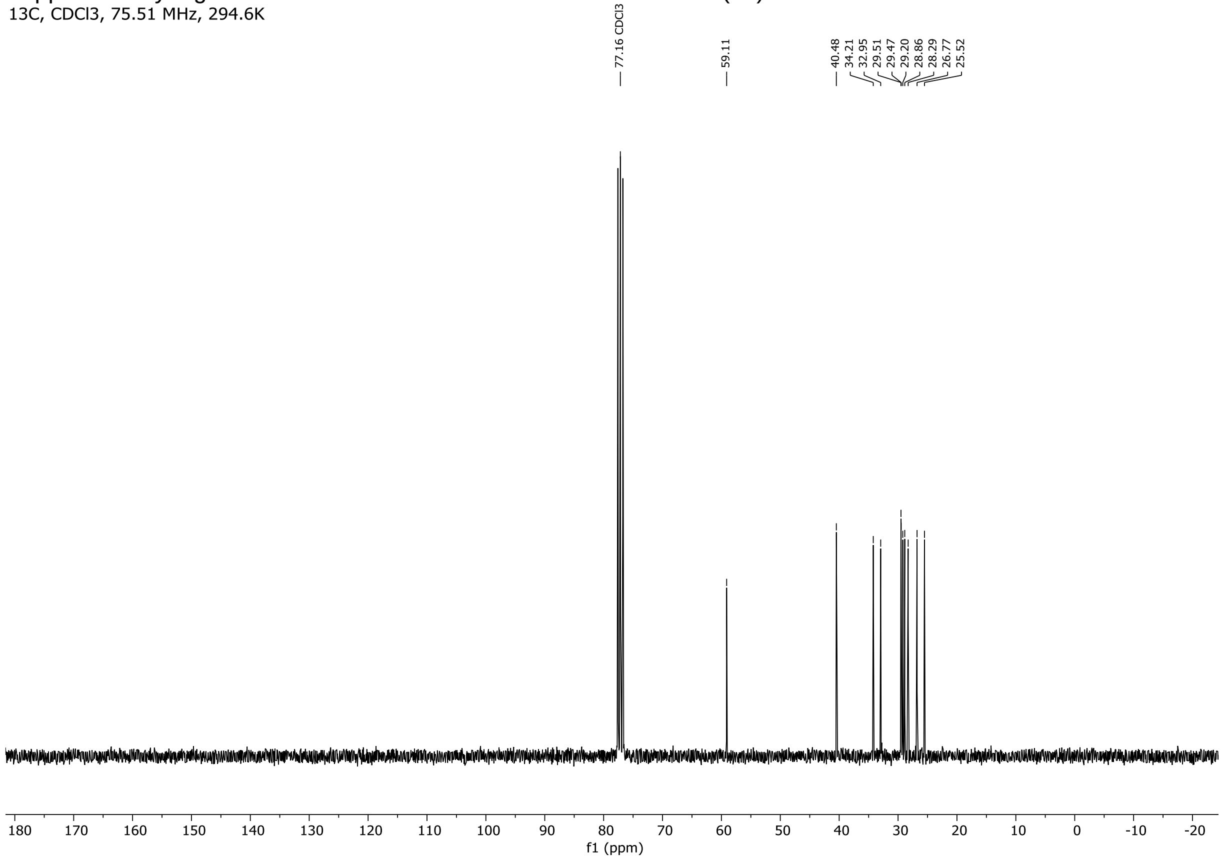

Supplementary Figure 55: ^{13}C NMR of 1-Bromo-10-chloroundecane (**7c**)

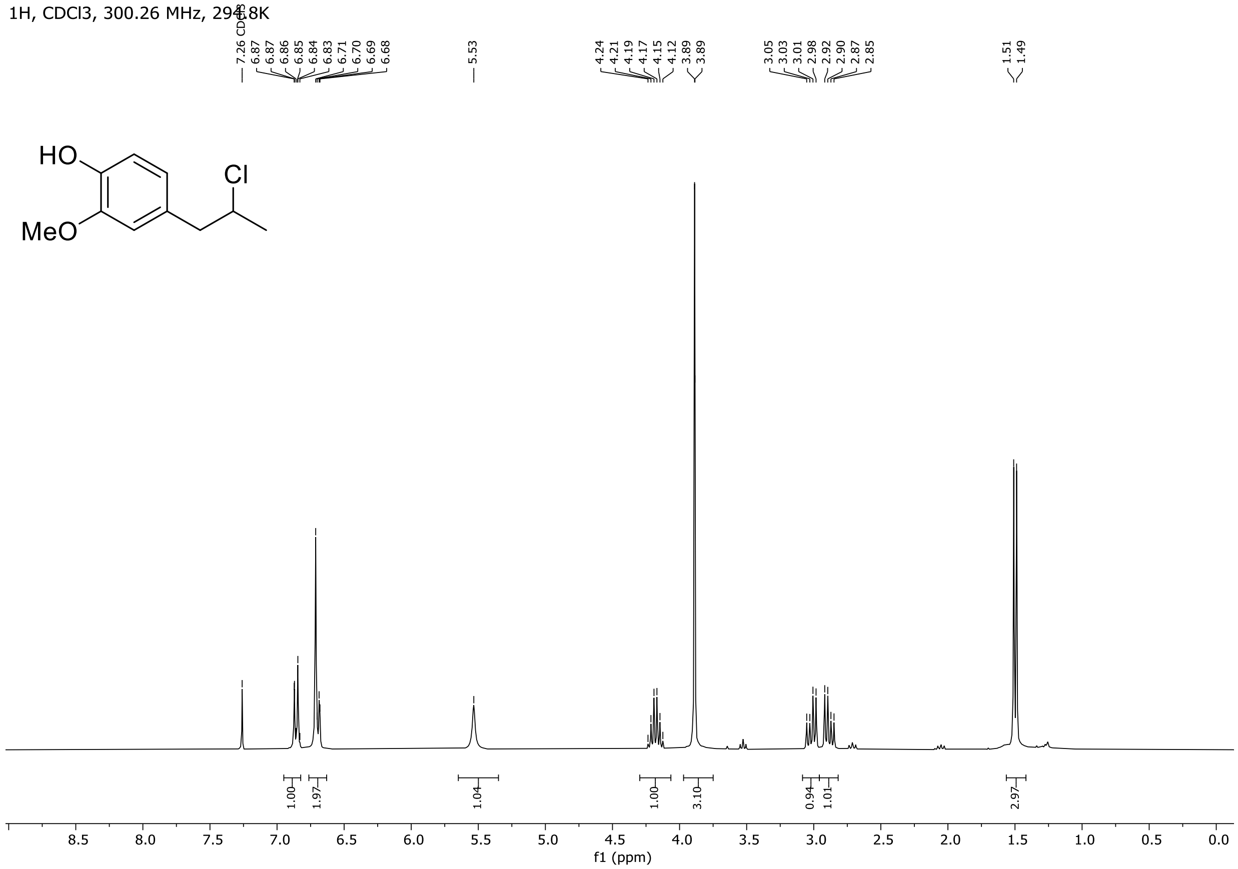

Supplementary Figure 56: ^{1}H NMR of 4-(2-Chloropropyl)-2-methoxyphenol (**7d**)

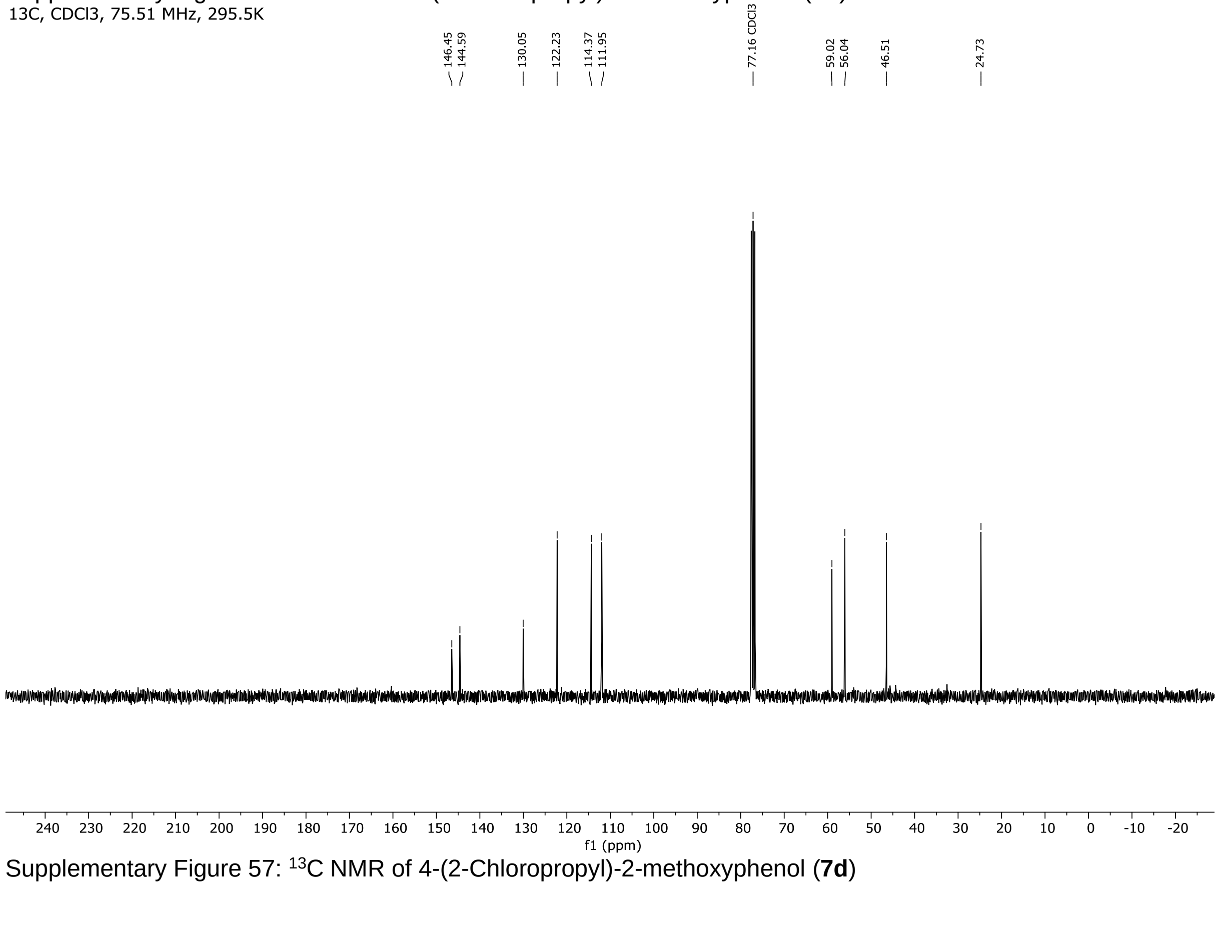

Supplementary Figure 57: ^{13}C NMR of 4-(2-Chloropropyl)-2-methoxyphenol (**7d**)

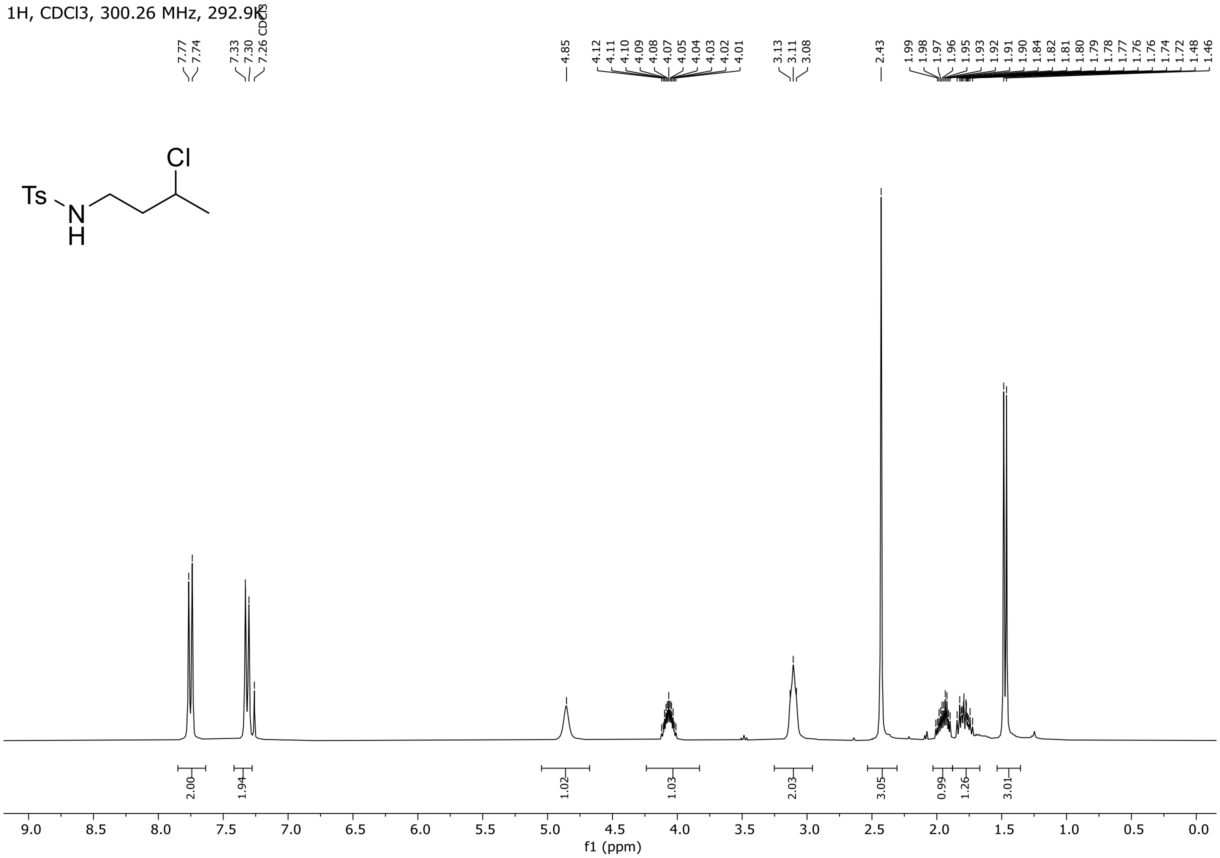

Supplementary Figure 58: ^{1}H NMR of *N*-(3-Chlorobutyl)-4-methylbenzenesulfonamide (**7e**)

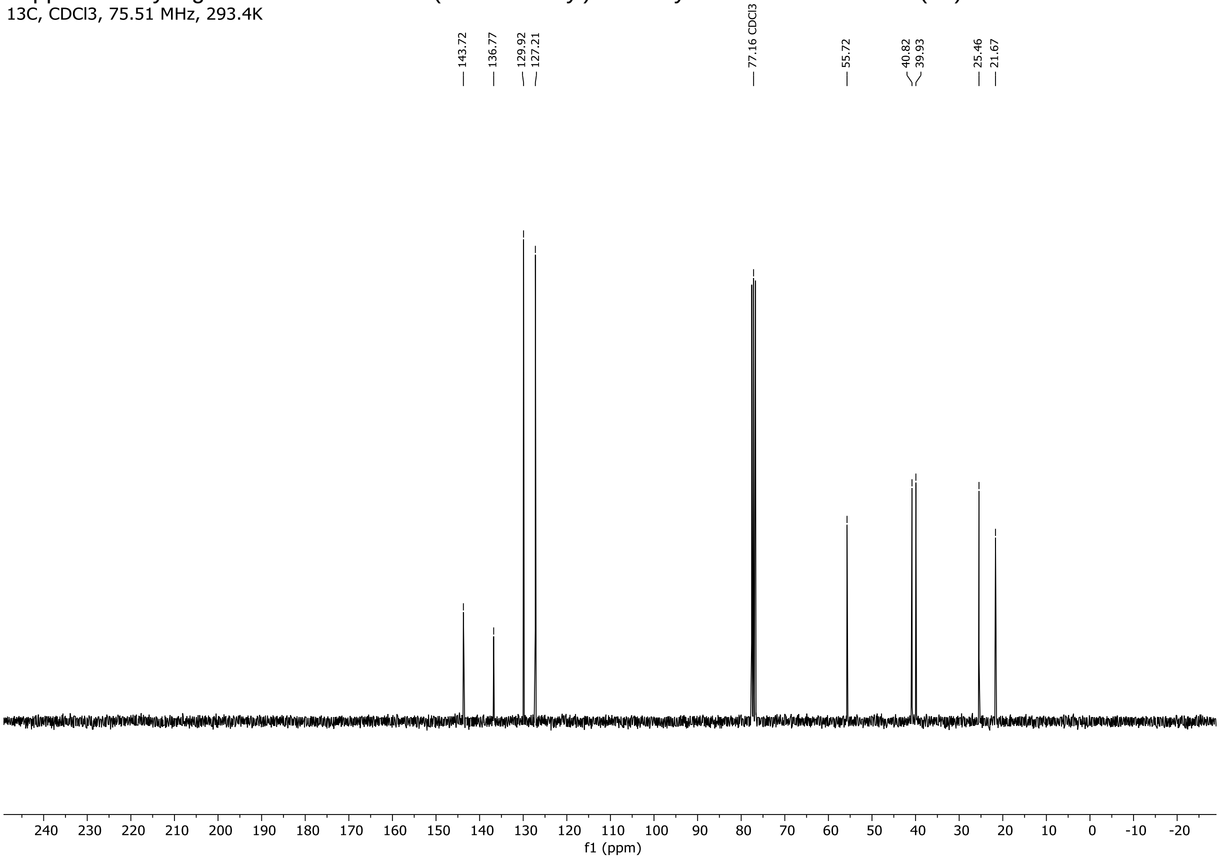

Supplementary Figure 59: ^{13}C NMR of *N*-(3-Chlorobutyl)-4-methylbenzenesulfonamide (**7e**)

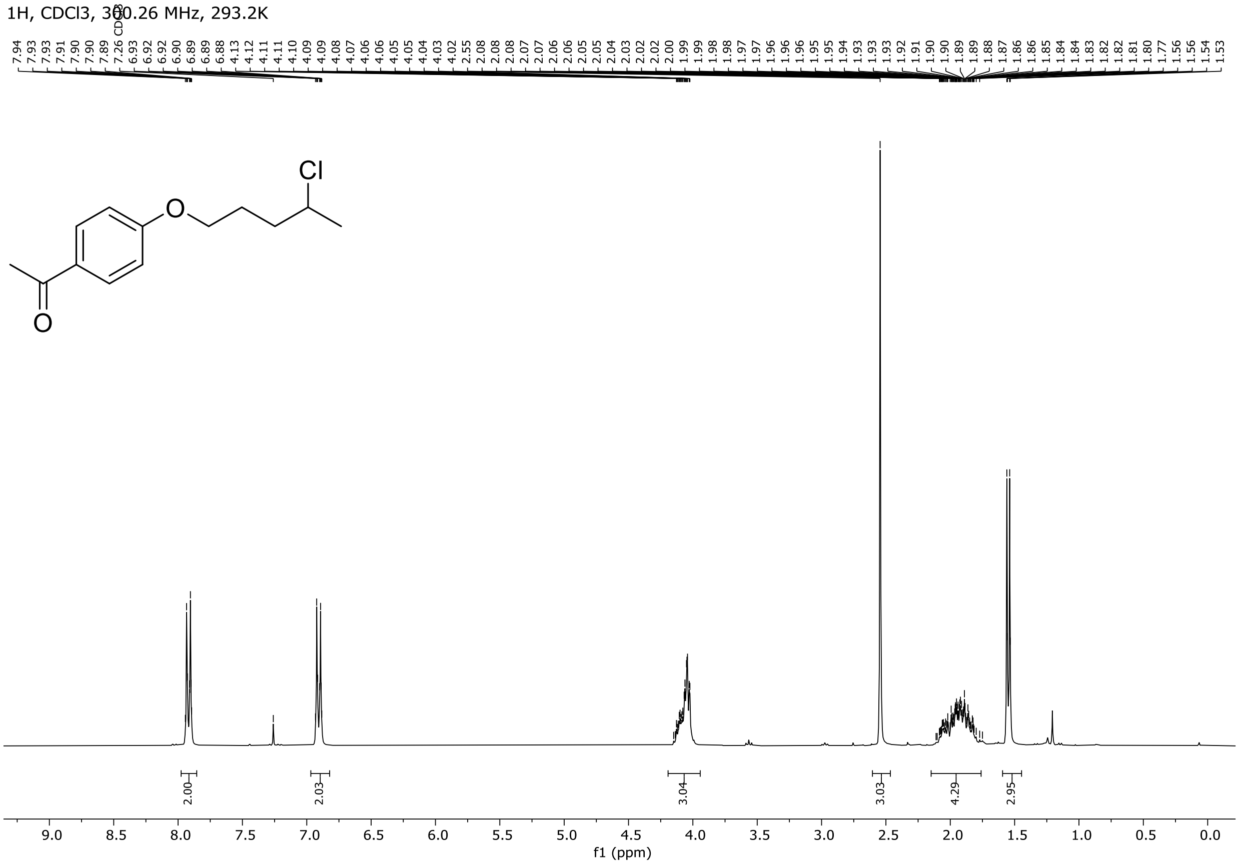

Supplementary Figure 60: ^{1}H NMR of 1-(4-((4-Chloropentyl)oxy)phenyl)ethan-1-one (**7h**)

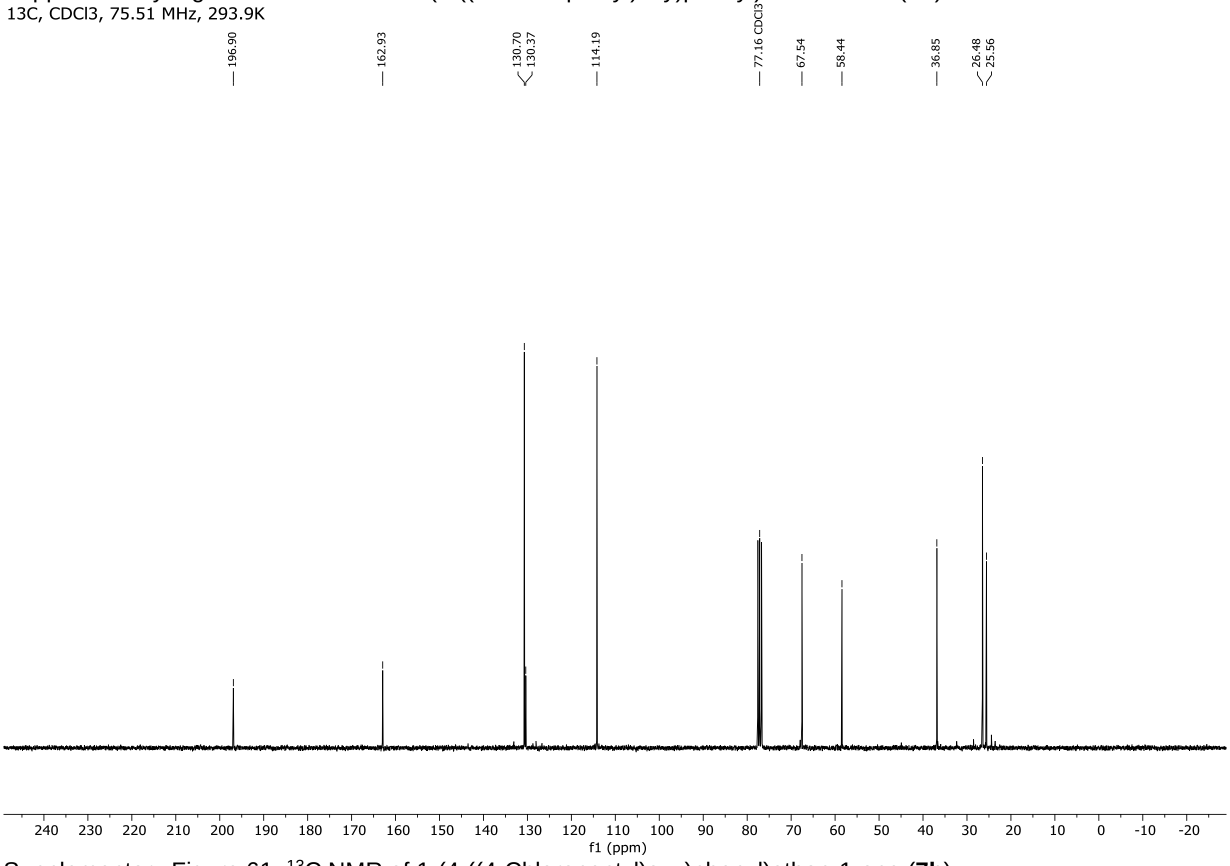

Supplementary Figure 61: ^{13}C NMR of 1-(4-((4-Chloropentyl)oxy)phenyl)ethan-1-one (**7h**)

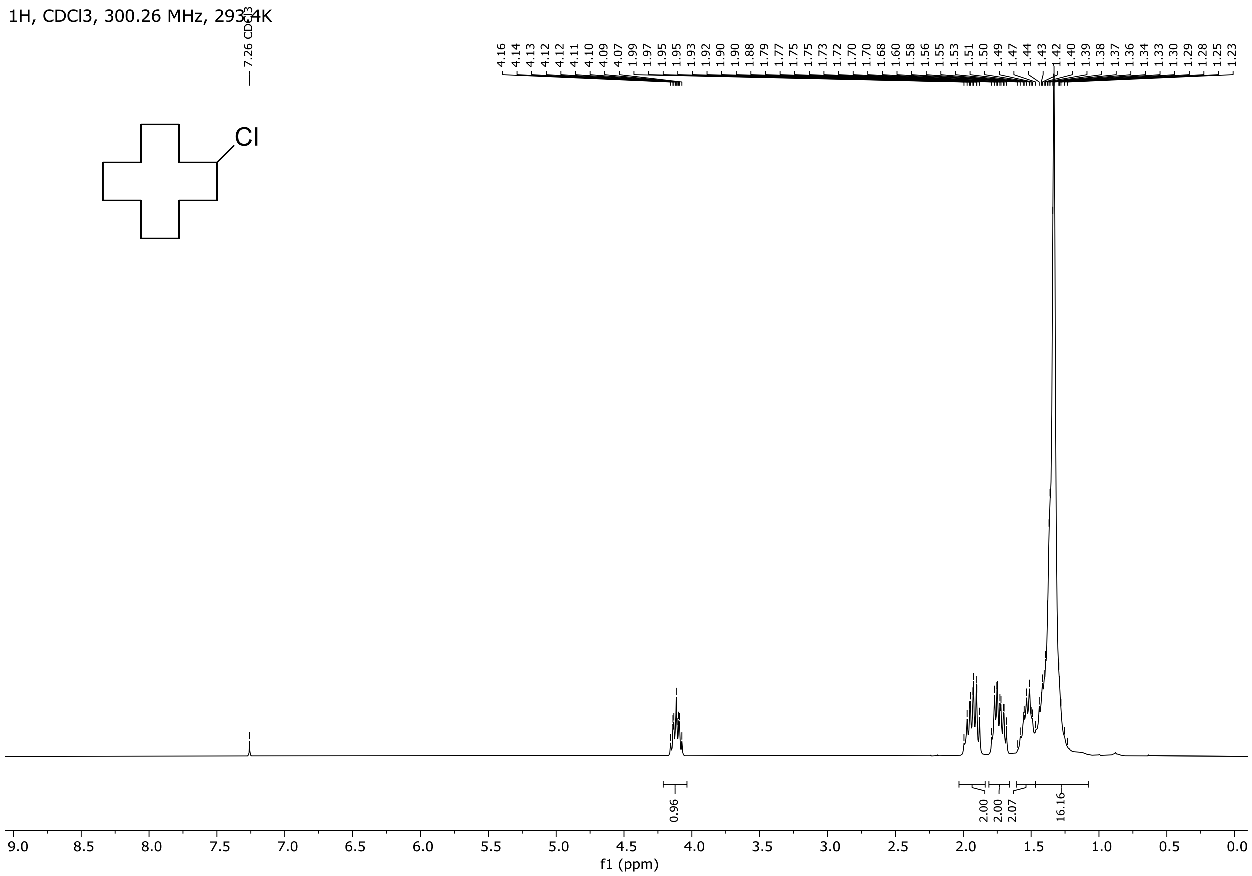

Supplementary Figure 62: ^{1}H NMR of Chlorocyclododecane (**7i**)

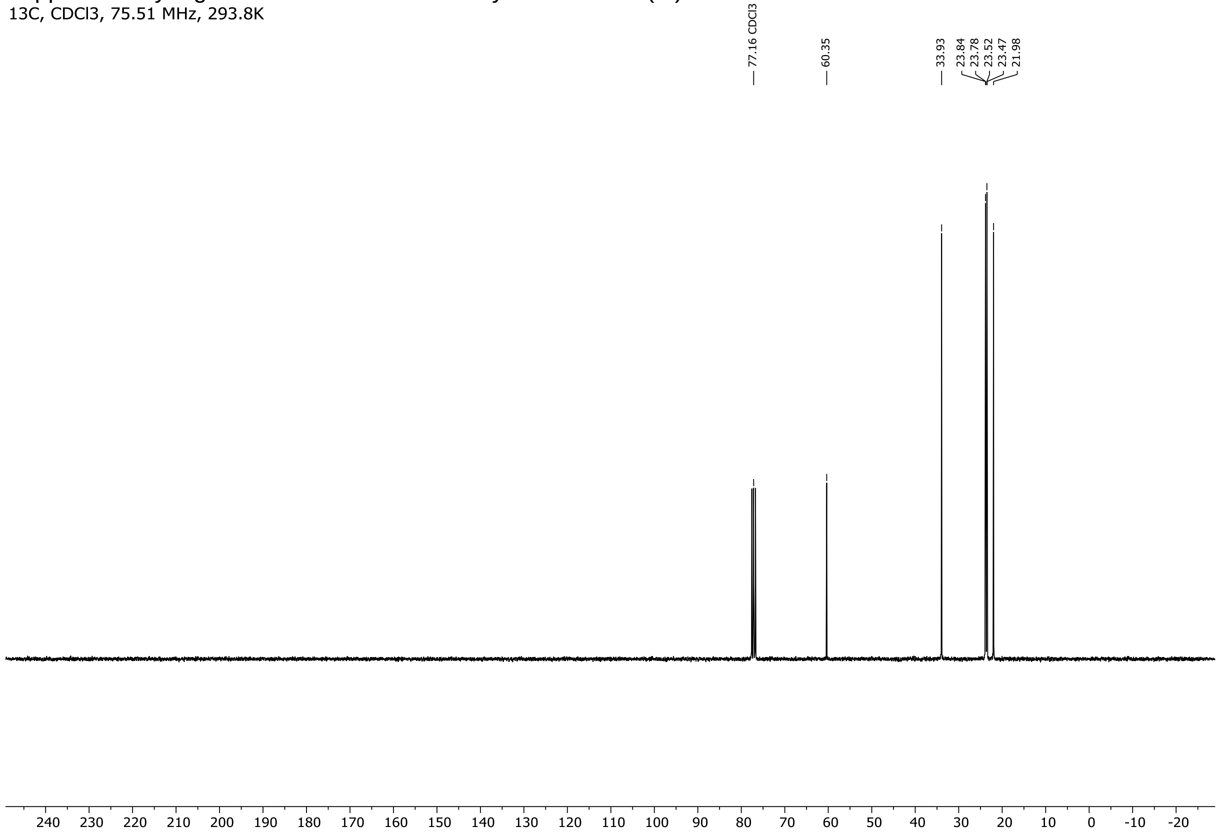

Supplementary Figure 63: ^{13}C NMR of Chlorocyclododecane (**7i**)

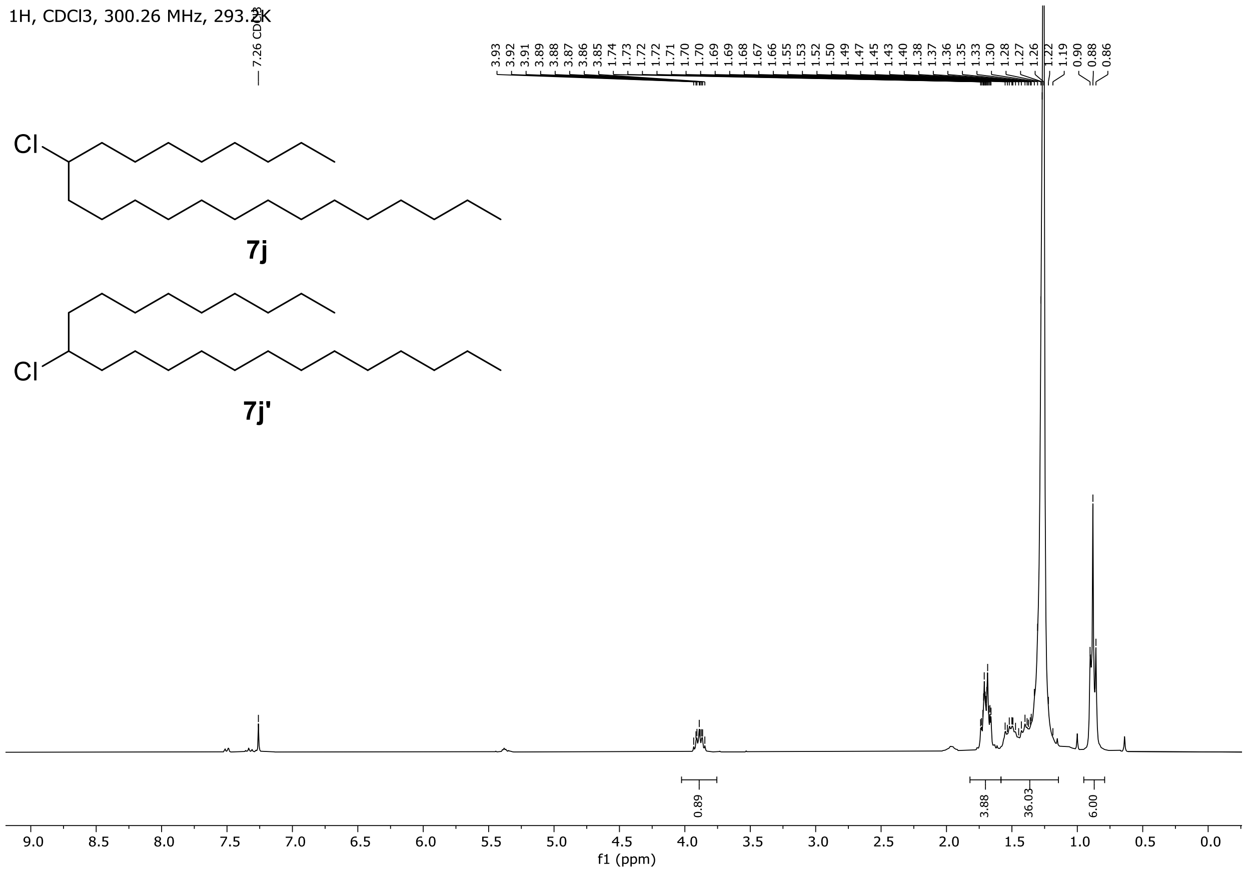

Supplementary Figure 64: ^{1}H NMR of 9-Chlorotricosane (**7j**) / 10-Chlorotricosane (**7j’**) 1:1

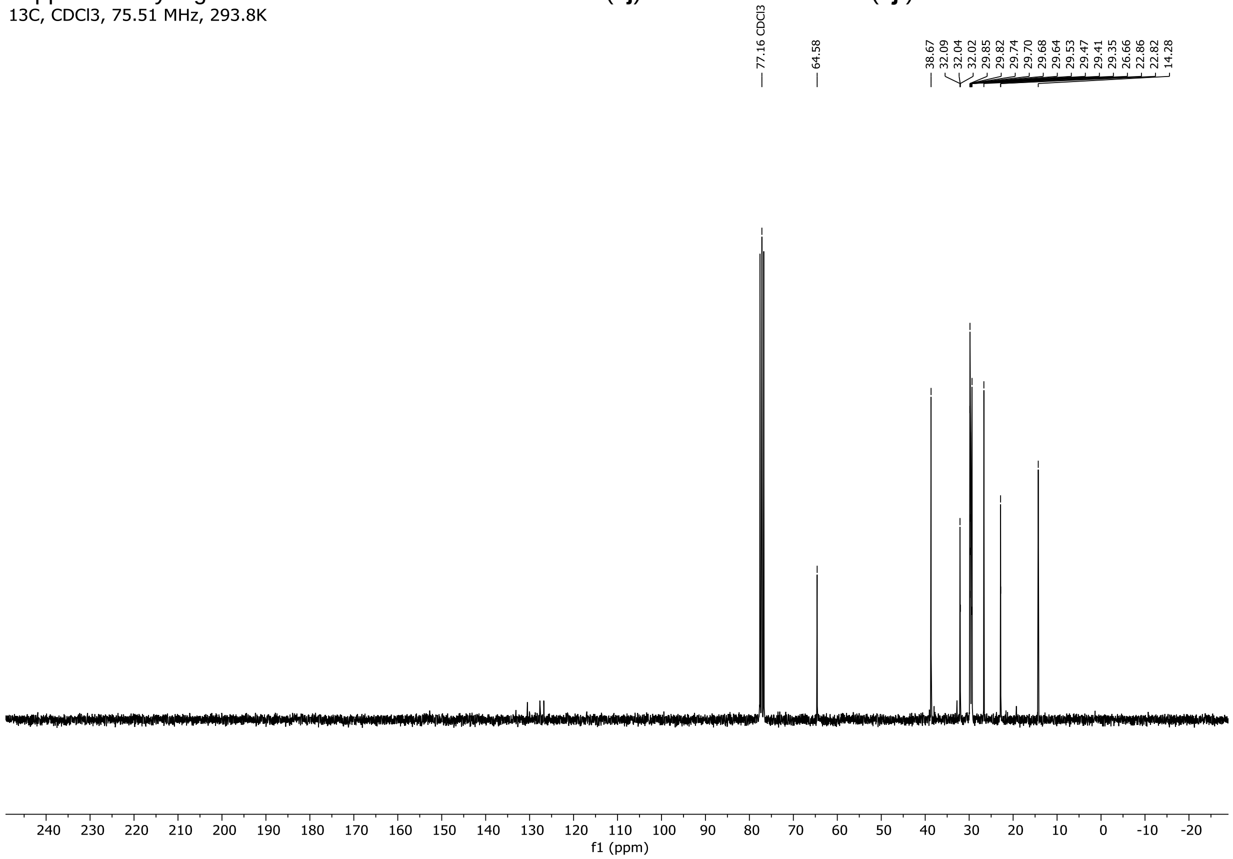

Supplementary Figure 65: ^{13}C NMR of 9-Chlorotricosane (**7j**) / 10-Chlorotricosane (**7j’**) 1:1

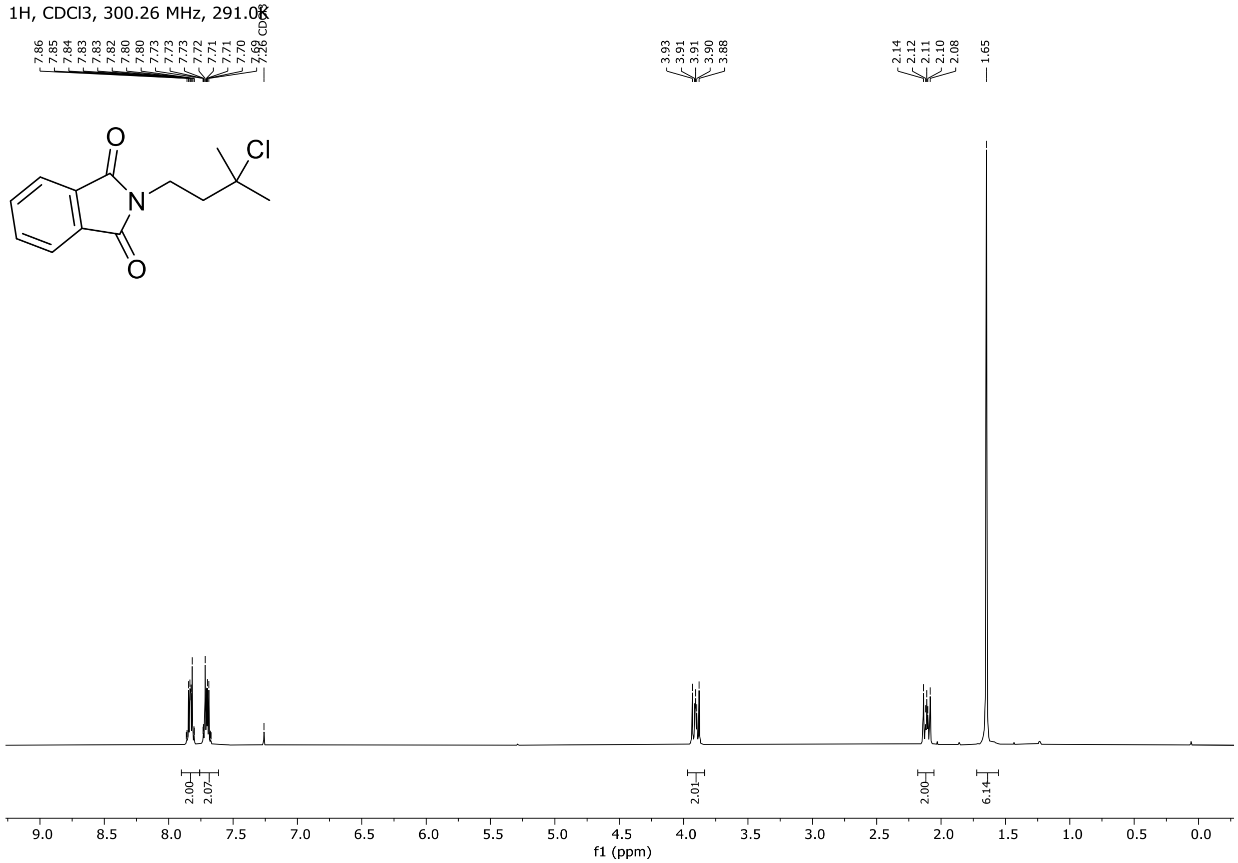

Supplementary Figure 66: ^{1}H NMR of 2-(3-Chloro-3-methylbutyl)isoindoline-1,3-dione (**7k**)

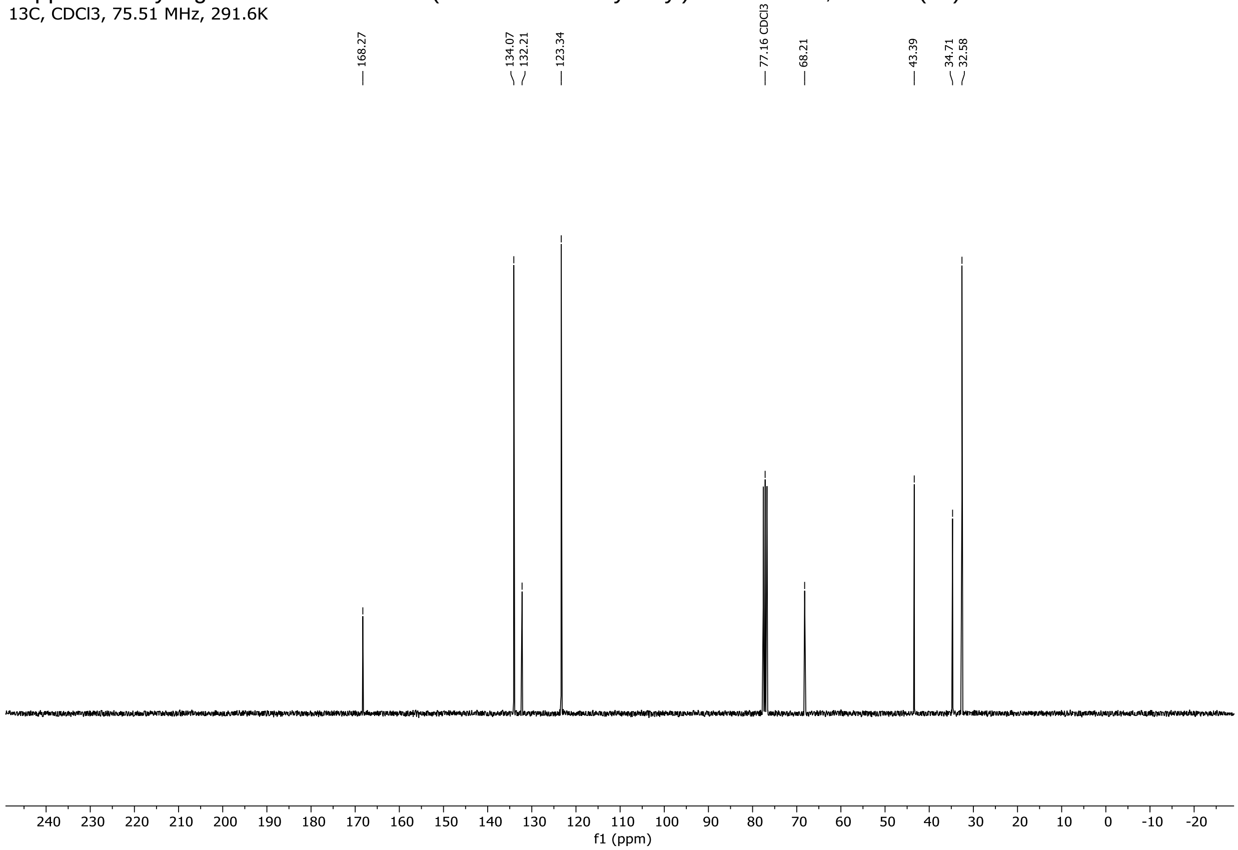

Supplementary Figure 67: ^{13}C NMR of 2-(3-Chloro-3-methylbutyl)isoindoline-1,3-dione (**7k**)

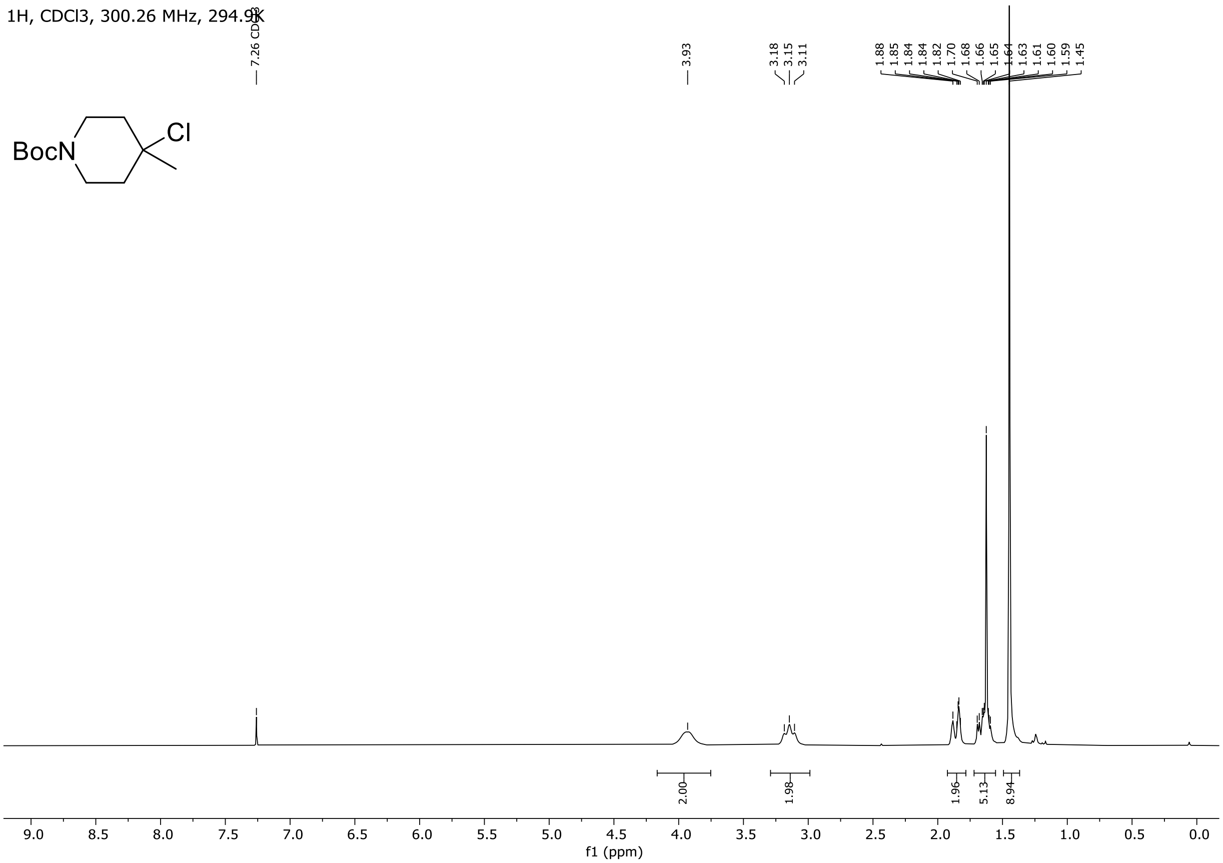

Supplementary Figure 68: ^{1}H NMR of *tert*-Butyl 4-chloro-4-methylpiperidine-1-carboxylate (**7l**)

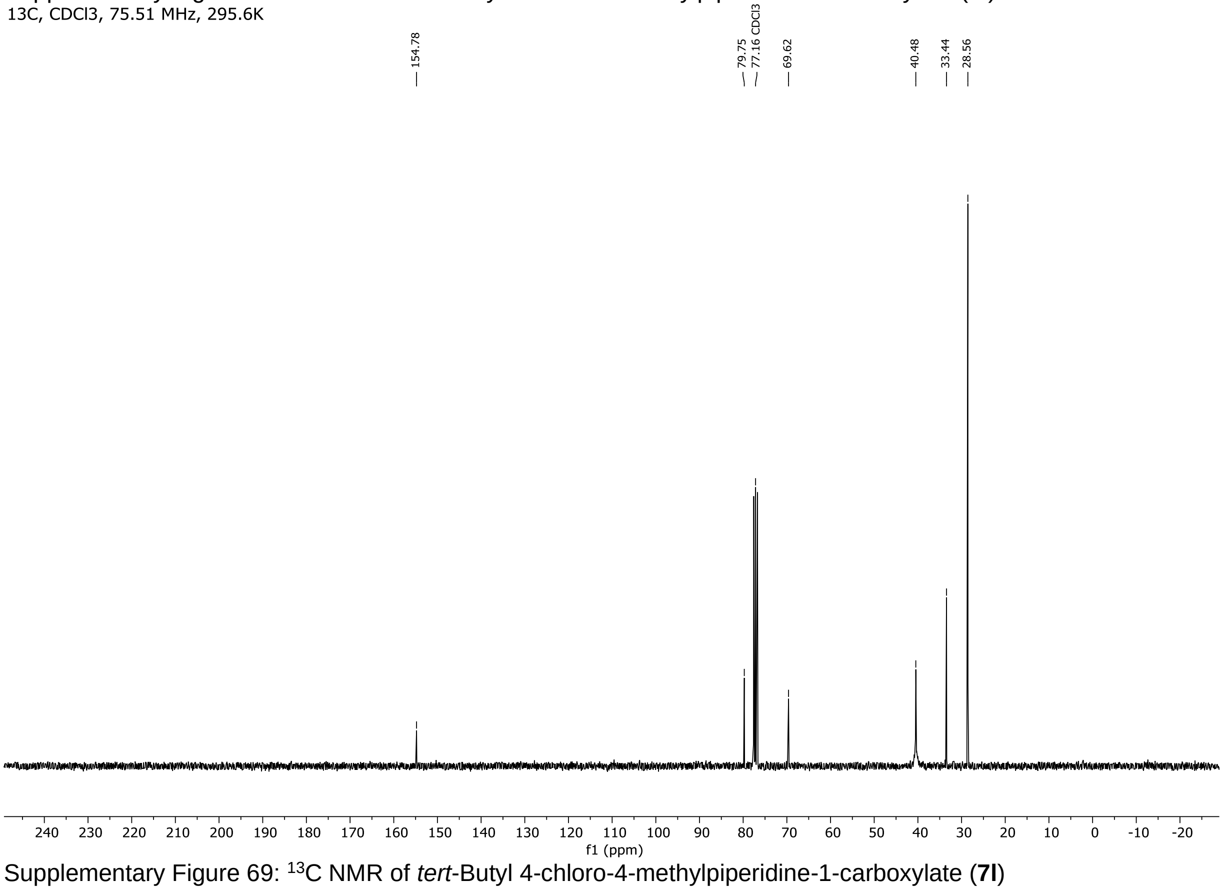

Supplementary Figure 69: ^{13}C NMR of *tert*-Butyl 4-chloro-4-methylpiperidine-1-carboxylate (**7l**)

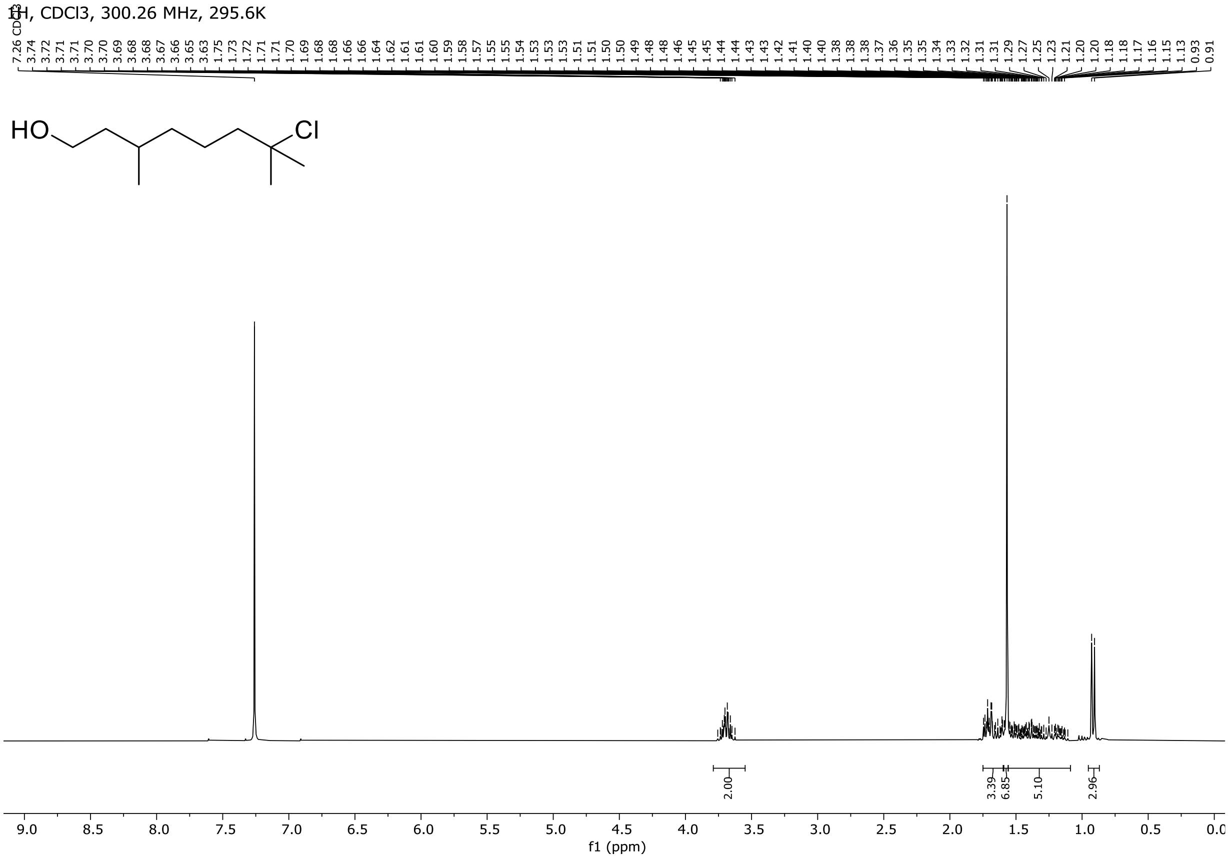

Supplementary Figure 70: ^{1}H NMR of 7-Chloro-3,7-dimethyloctan-1-ol (**7m**)

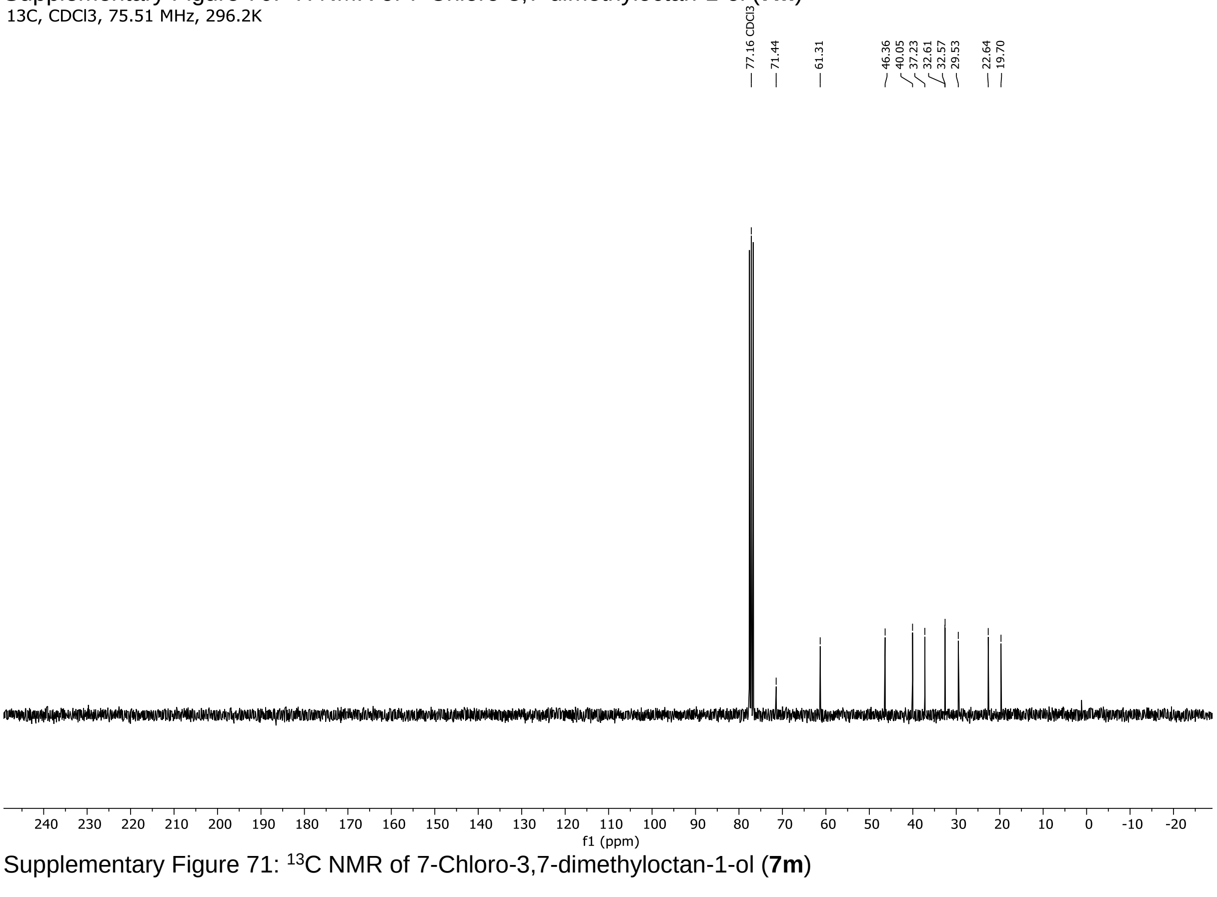

Supplementary Figure 71: ^{13}C NMR of 7-Chloro-3,7-dimethyloctan-1-ol (**7m**)

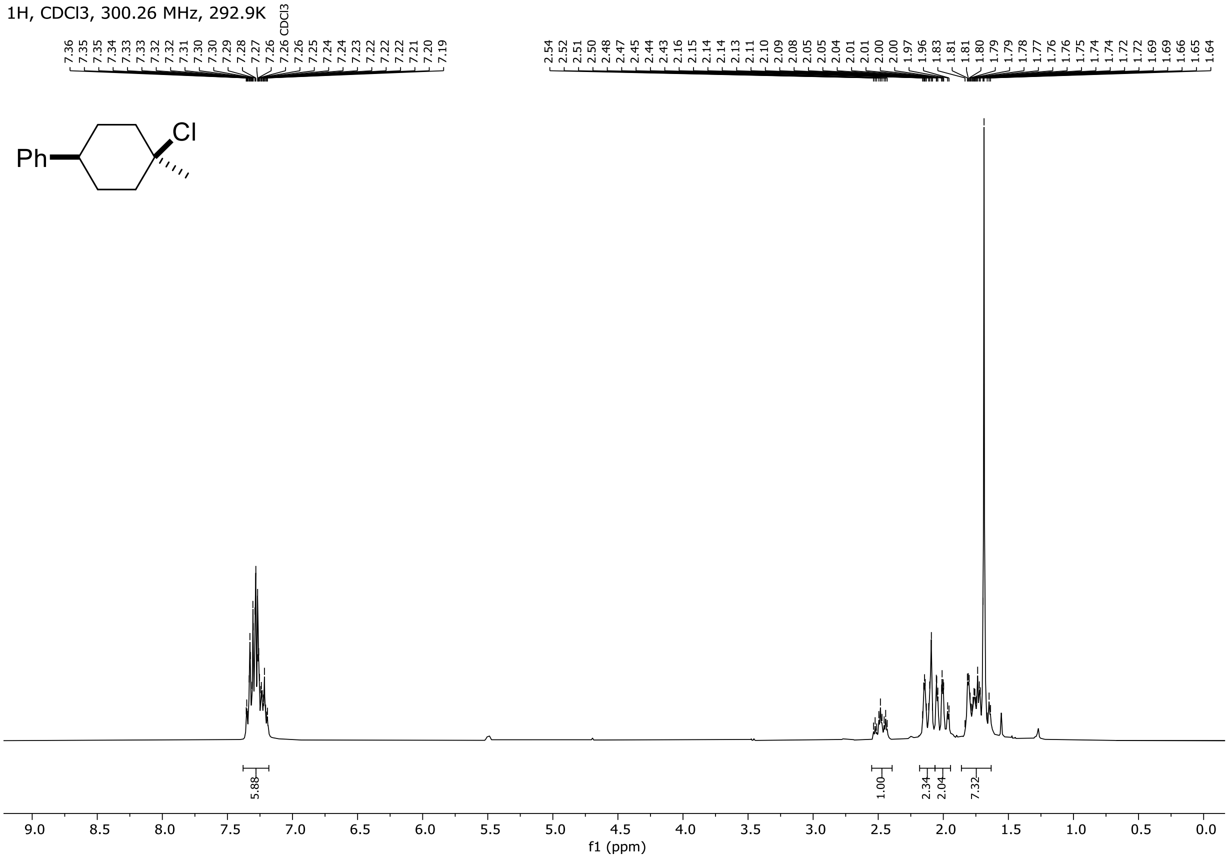

Supplementary Figure 72: ^{1}H NMR of (4-Chloro-4-methylcyclohexyl)benzene (**7n**)

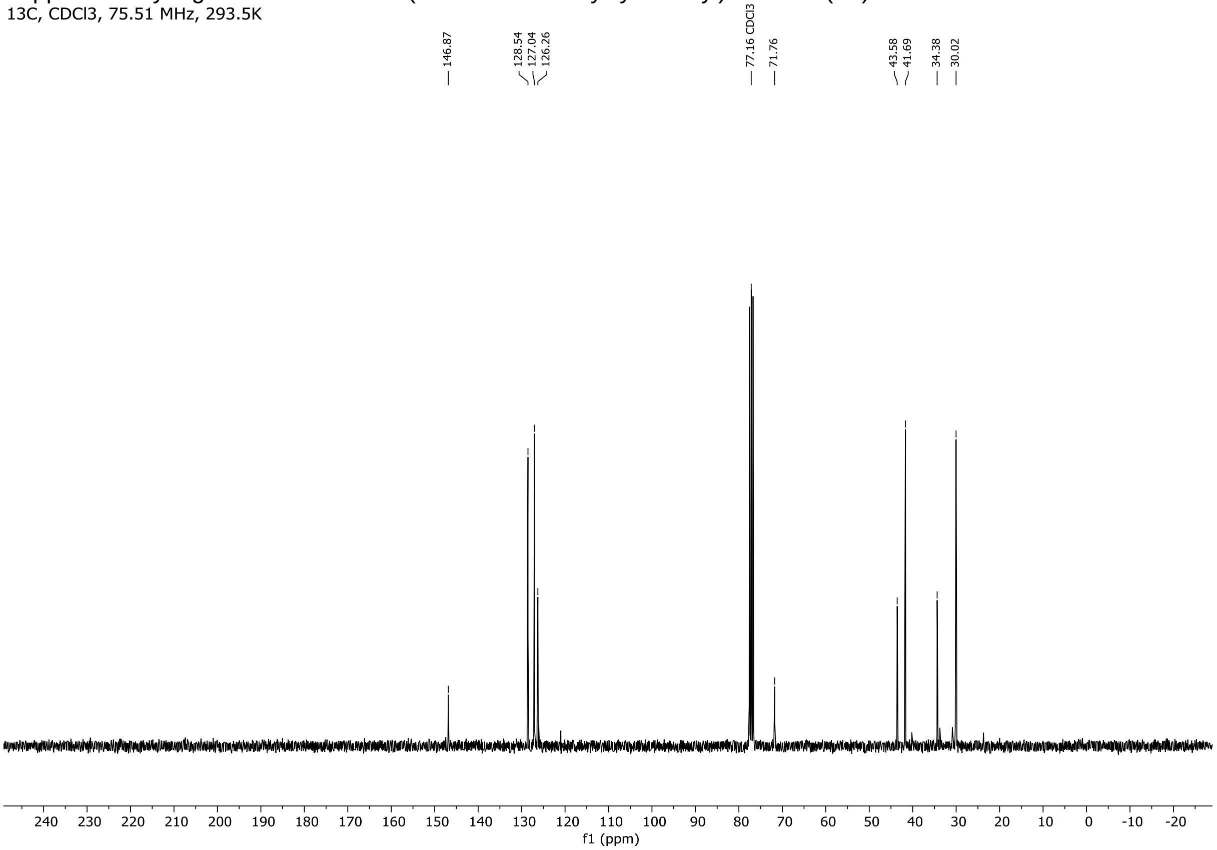

Supplementary Figure 73: ^{13}C NMR of (4-Chloro-4-methylcyclohexyl)benzene (**7n**)

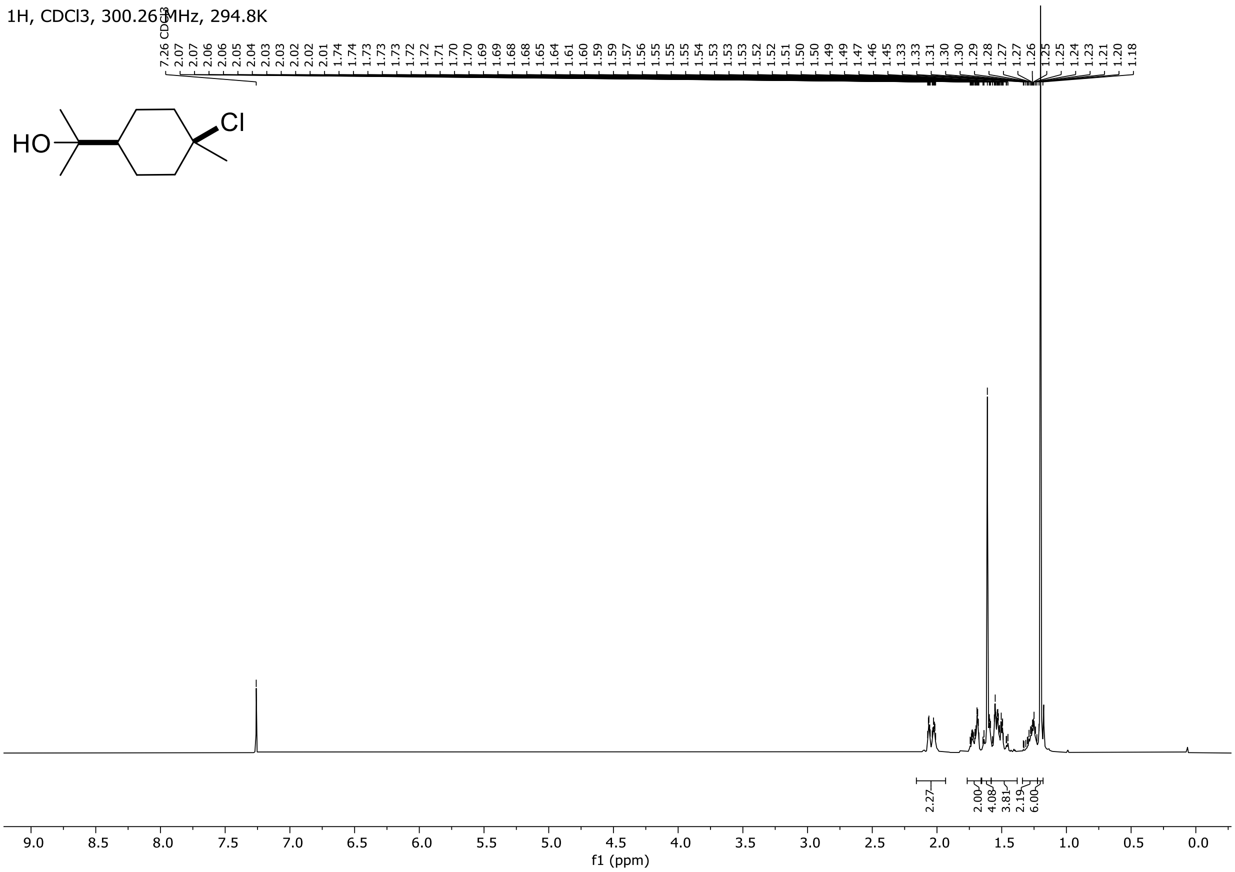

Supplementary Figure 74: ^{1}H NMR of 2-(4-Chloro-4-methylcyclohexyl)propan-2-ol (**7o**)

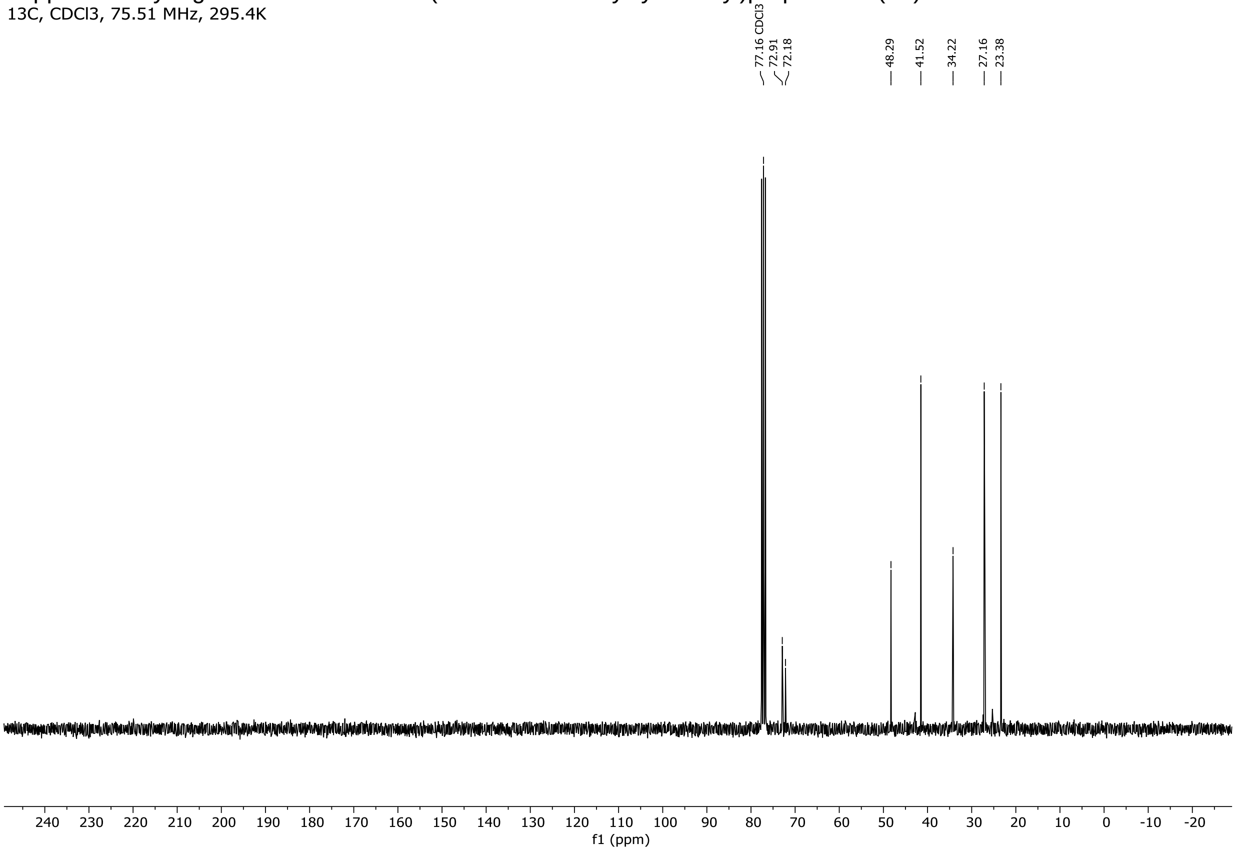

Supplementary Figure 75: ^{13}C NMR of 2-(4-Chloro-4-methylcyclohexyl)propan-2-ol (**7o**)

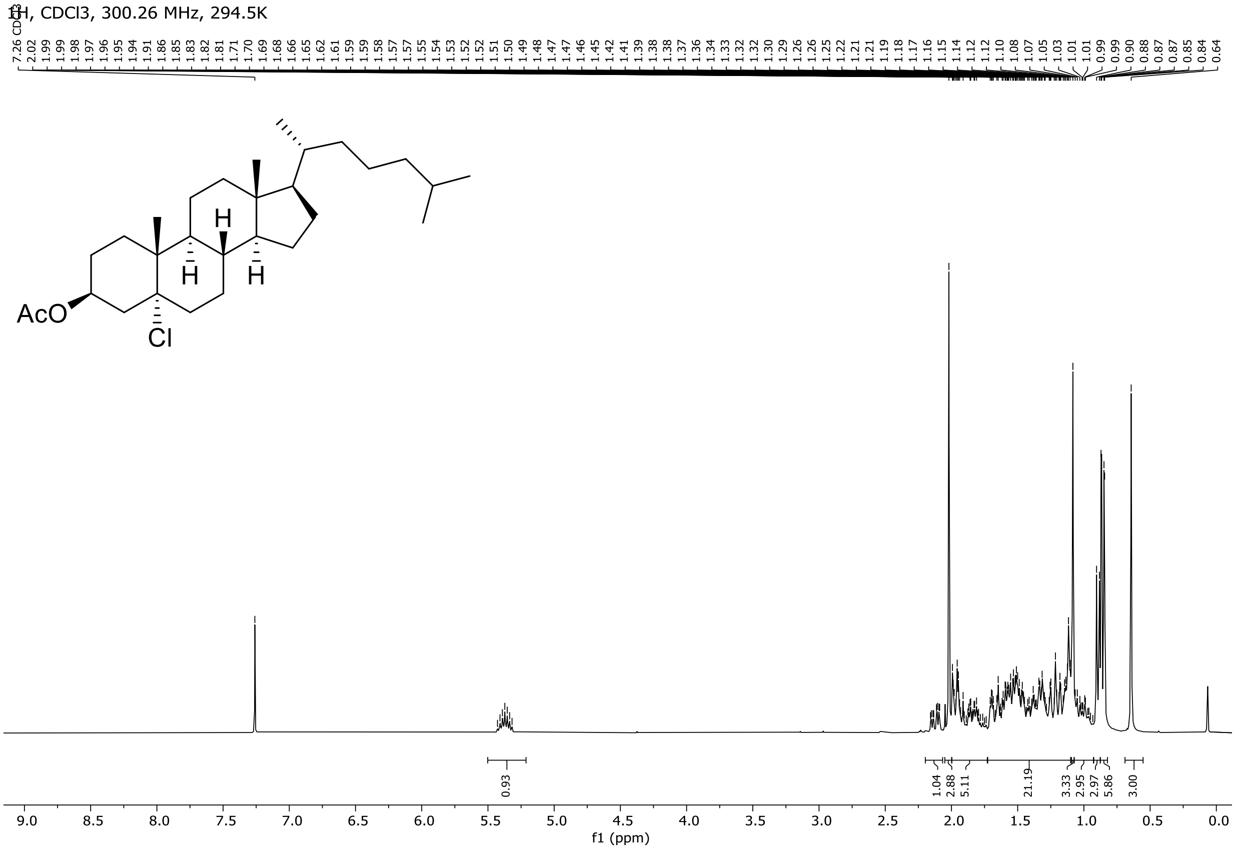

Supplementary Figure 76: ^{1}H NMR of (5*R*)-5-Chloro-cholesteryl acetate (**7q**)

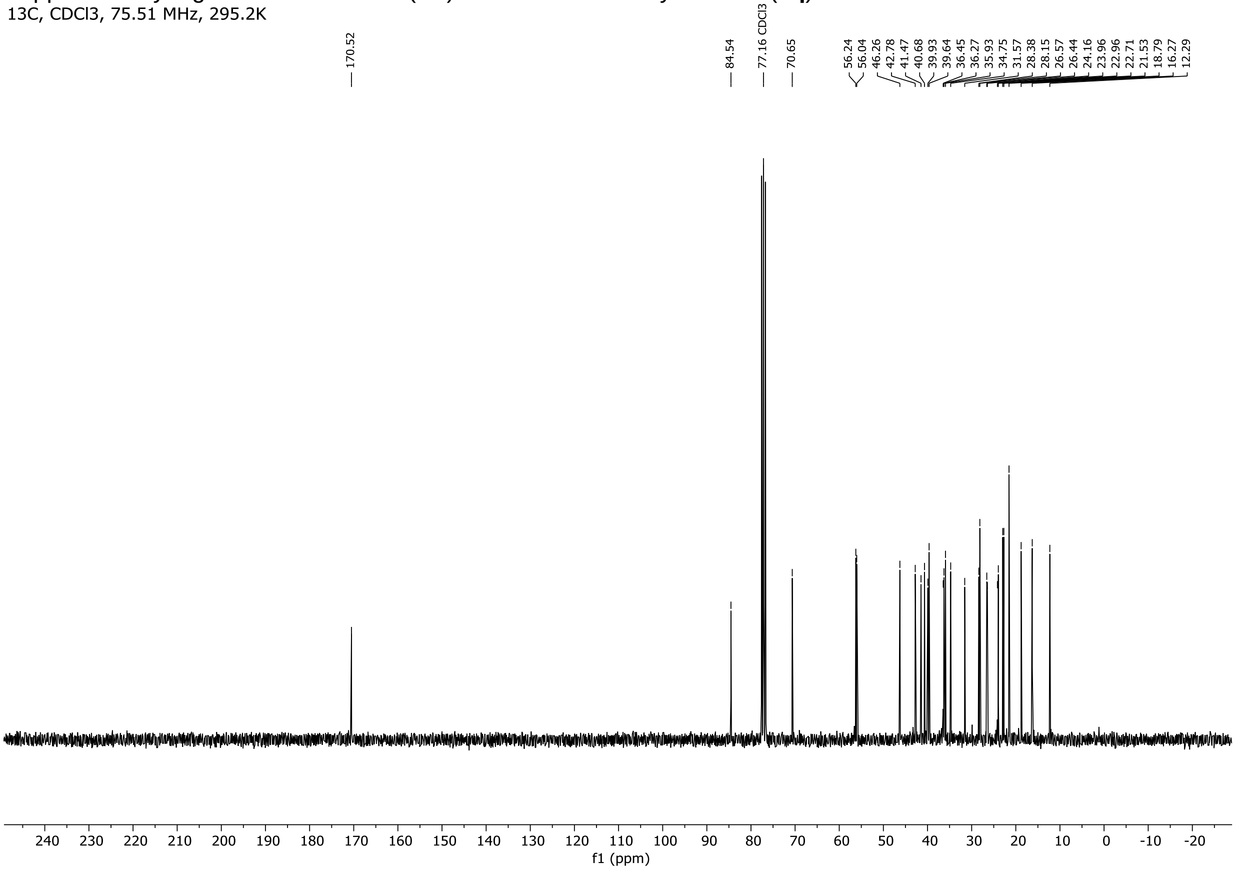

Supplementary Figure 77: ^{13}C NMR of (5*R*)-5-Chloro-cholesteryl acetate (**7q**)

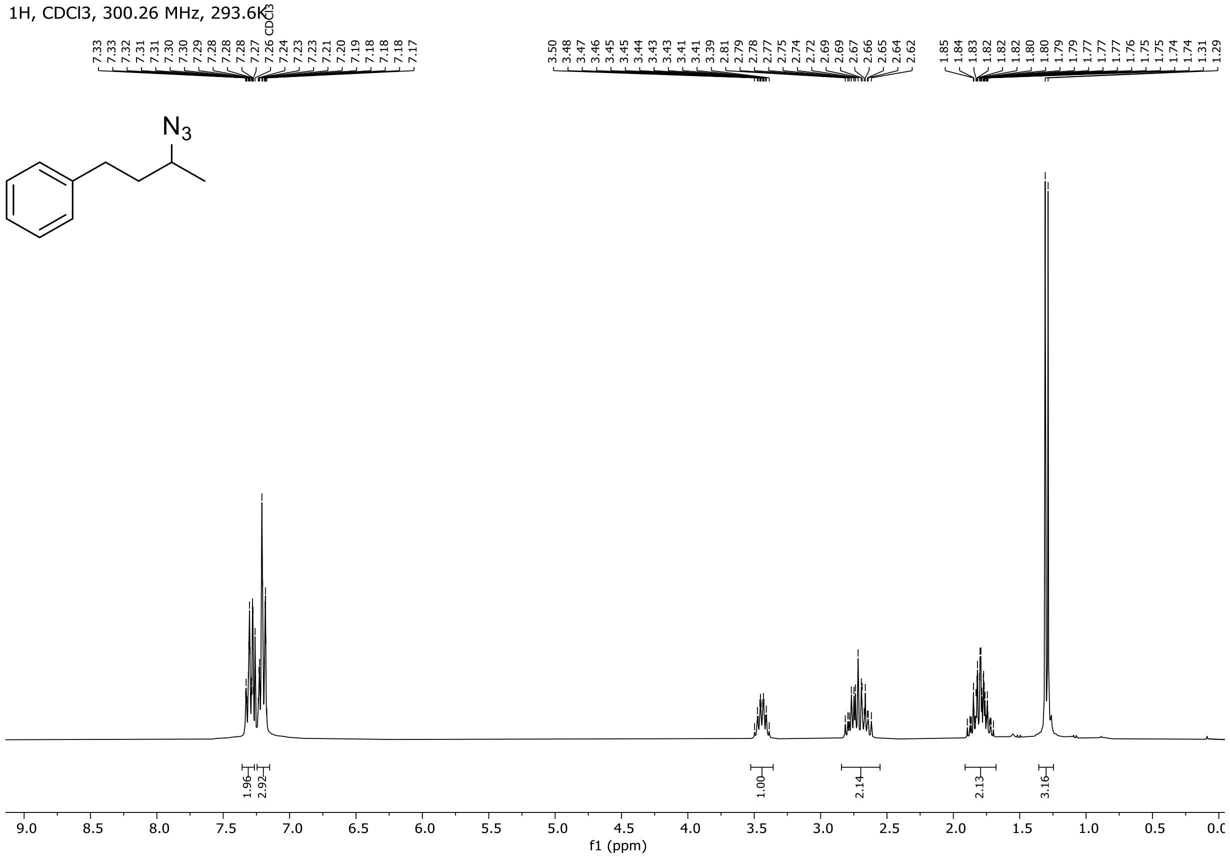

Supplementary Figure 78: ^{1}H NMR of (3-Azidobutyl)benzene (**8a**)

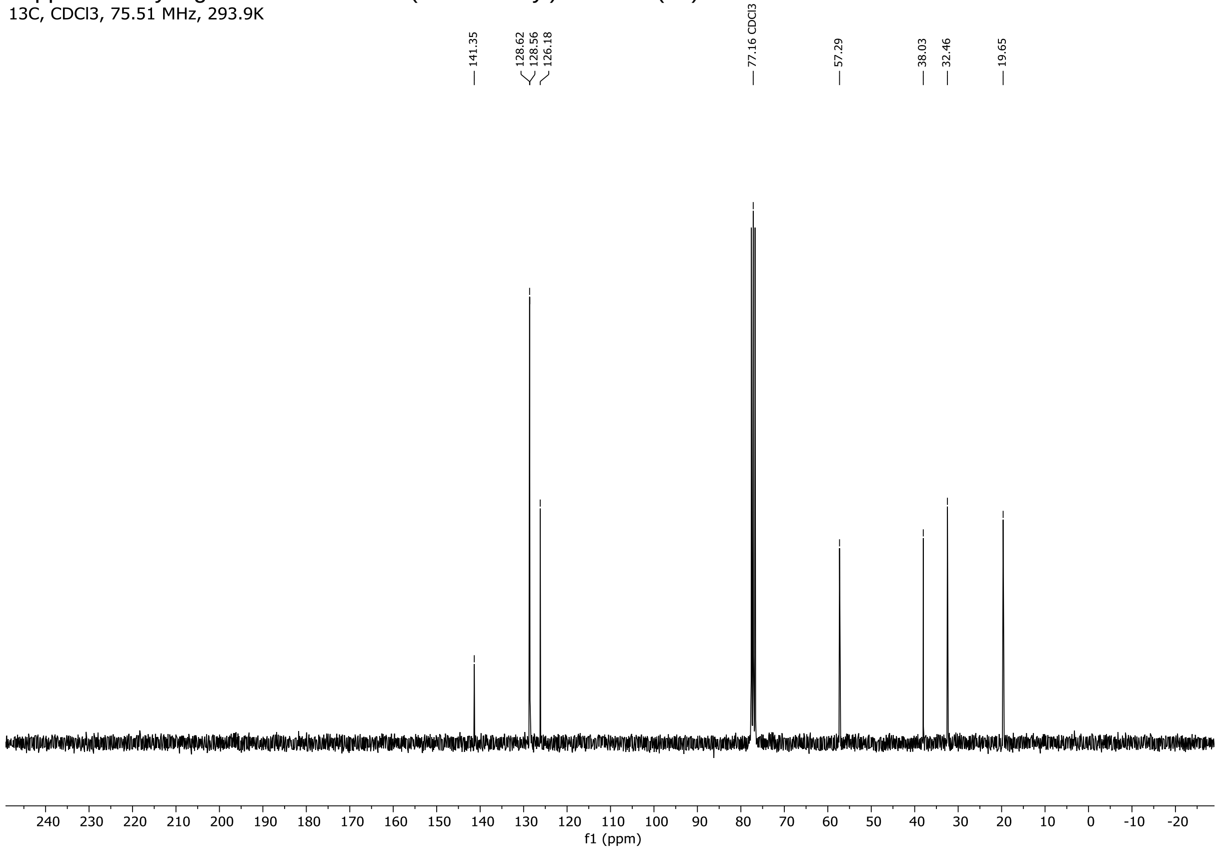

Supplementary Figure 79: ^{13}C NMR of (3-Azidobutyl)benzene (**8a**)

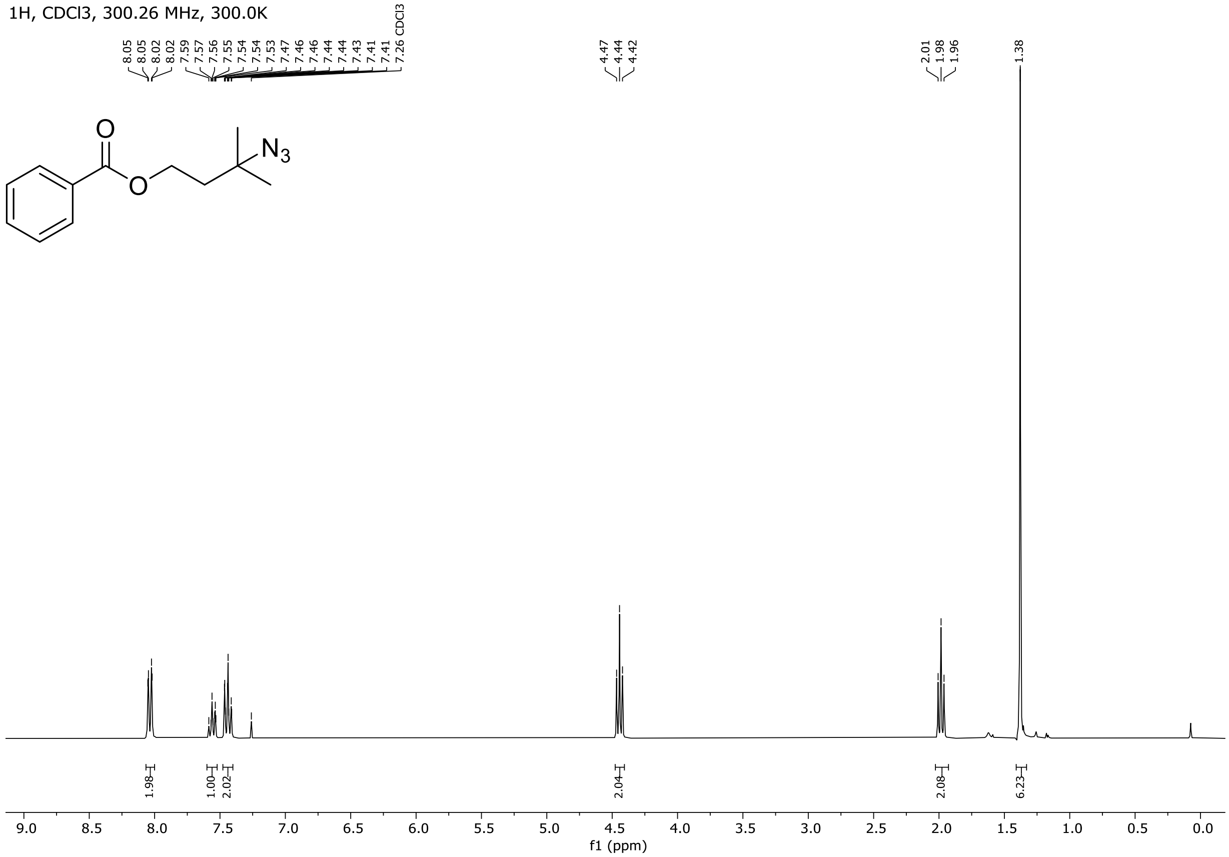

Supplementary Figure 80: ^{1}H NMR of 3-Azido-3-methylbutyl benzoate (**8b**)

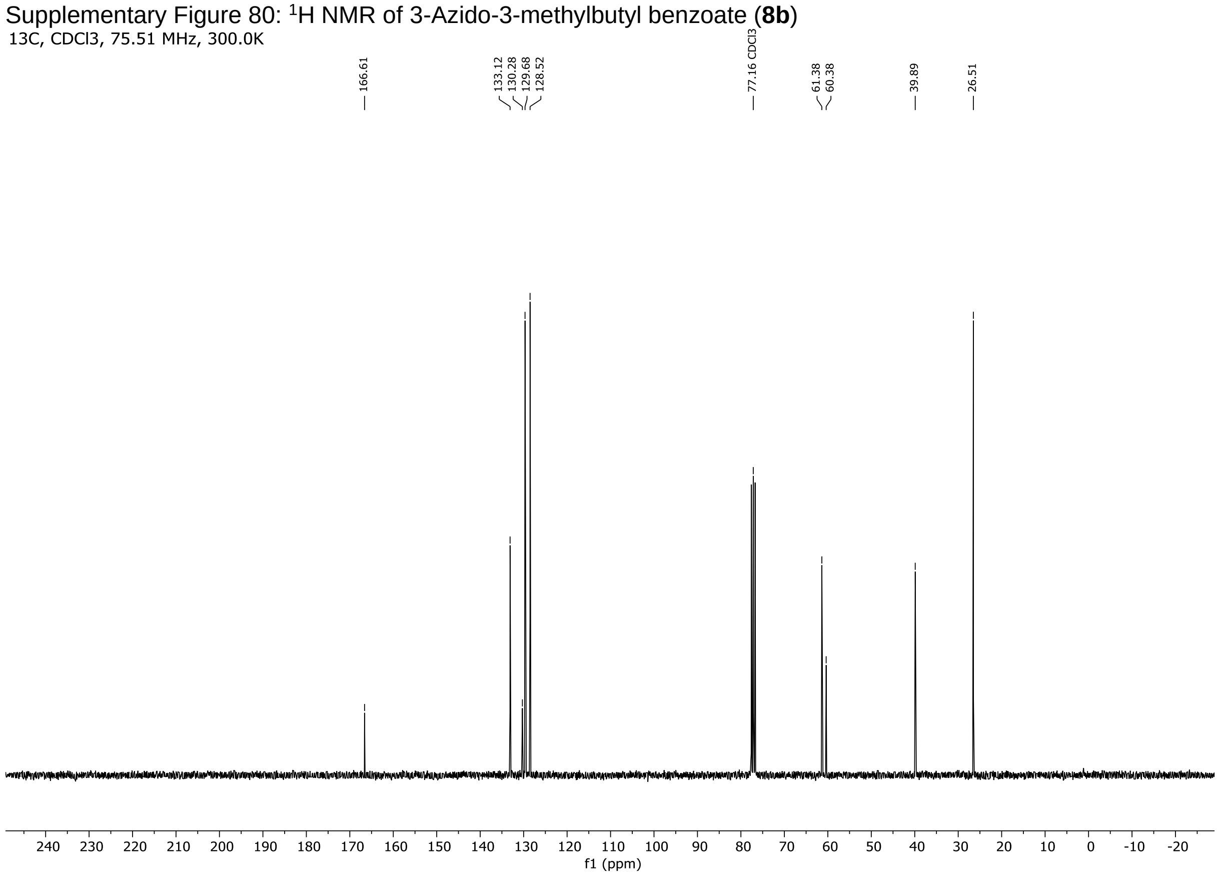

Supplementary Figure 81: ^{13}C NMR of 3-Azido-3-methylbutyl benzoate (**8b**)

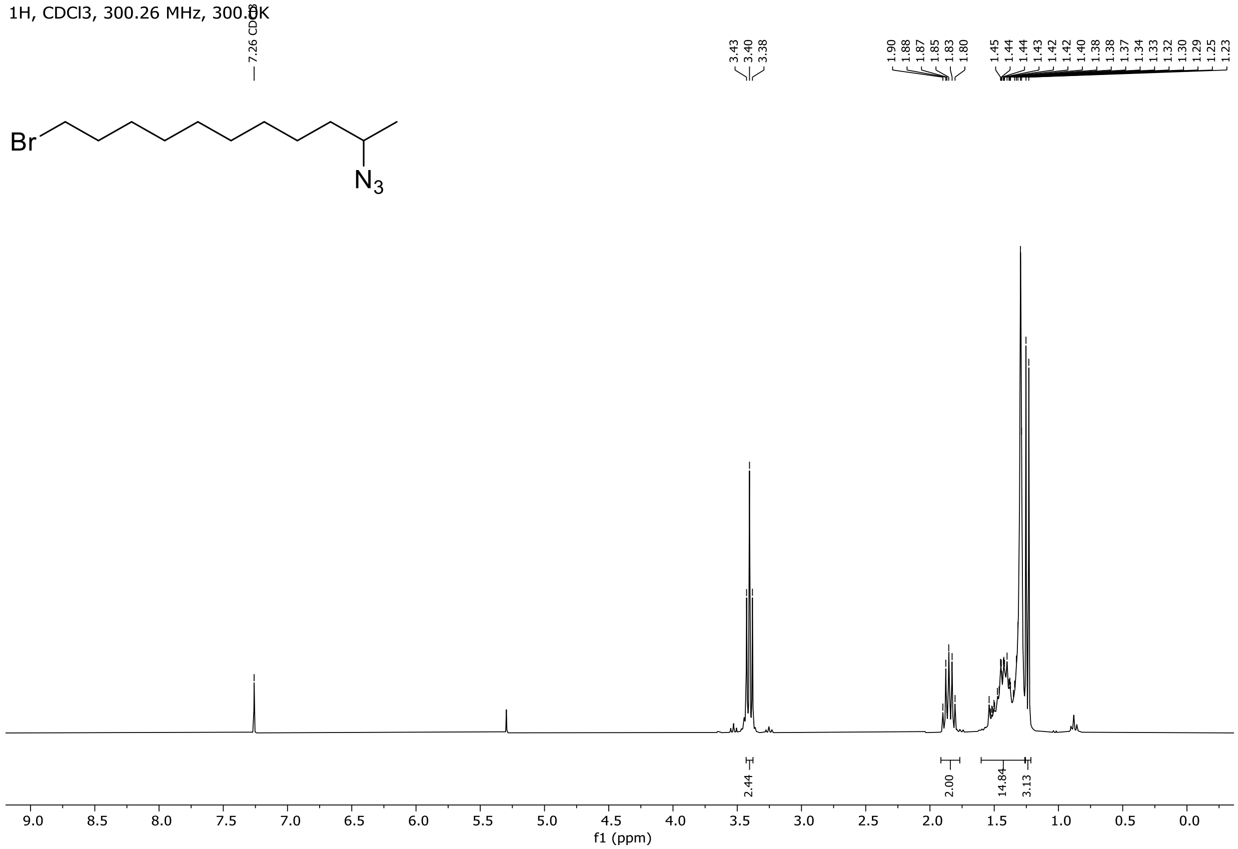

Supplementary Figure 82: ^{1}H NMR of 10-Azido-1-bromoundecane (**8c**)

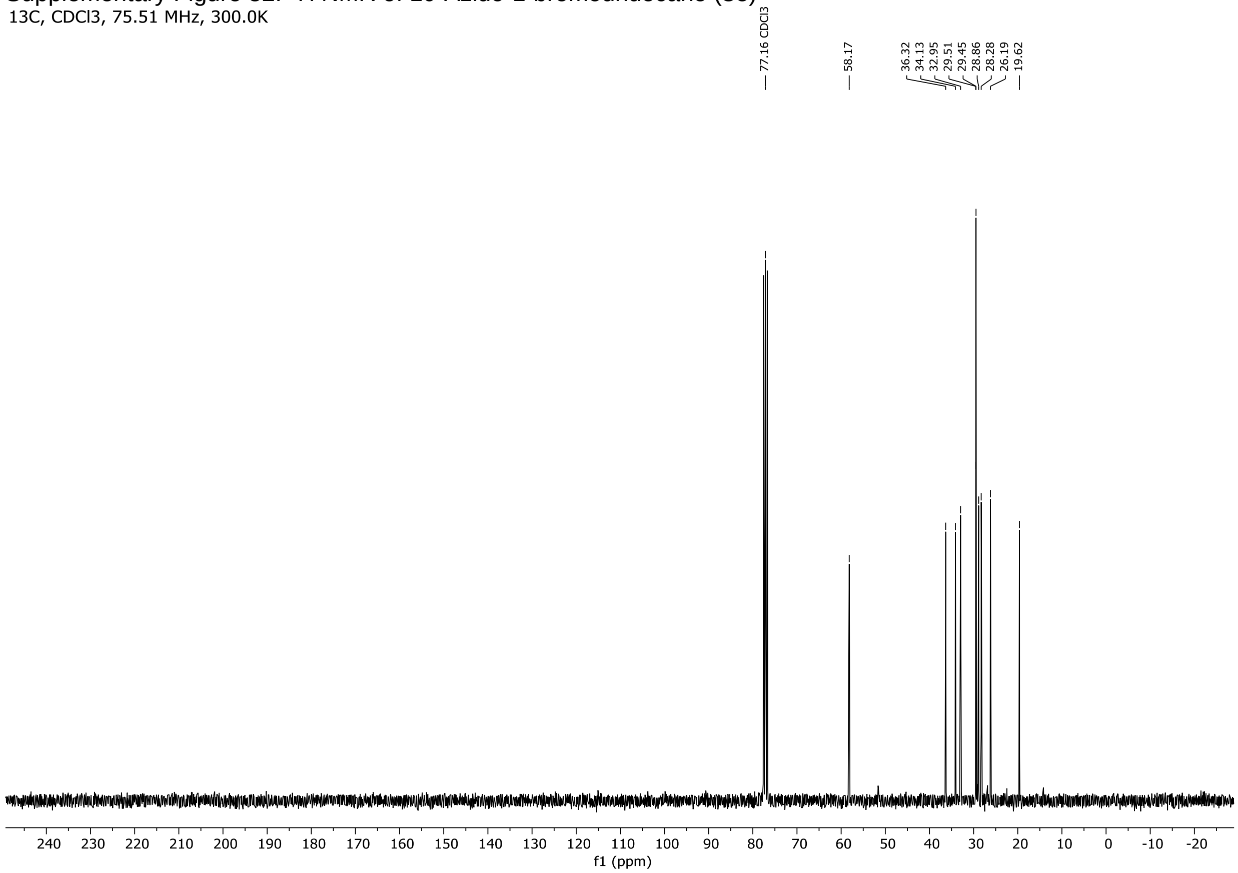

Supplementary Figure 83: ^{13}C NMR of 10-Azido-1-bromoundecane (**8c**)

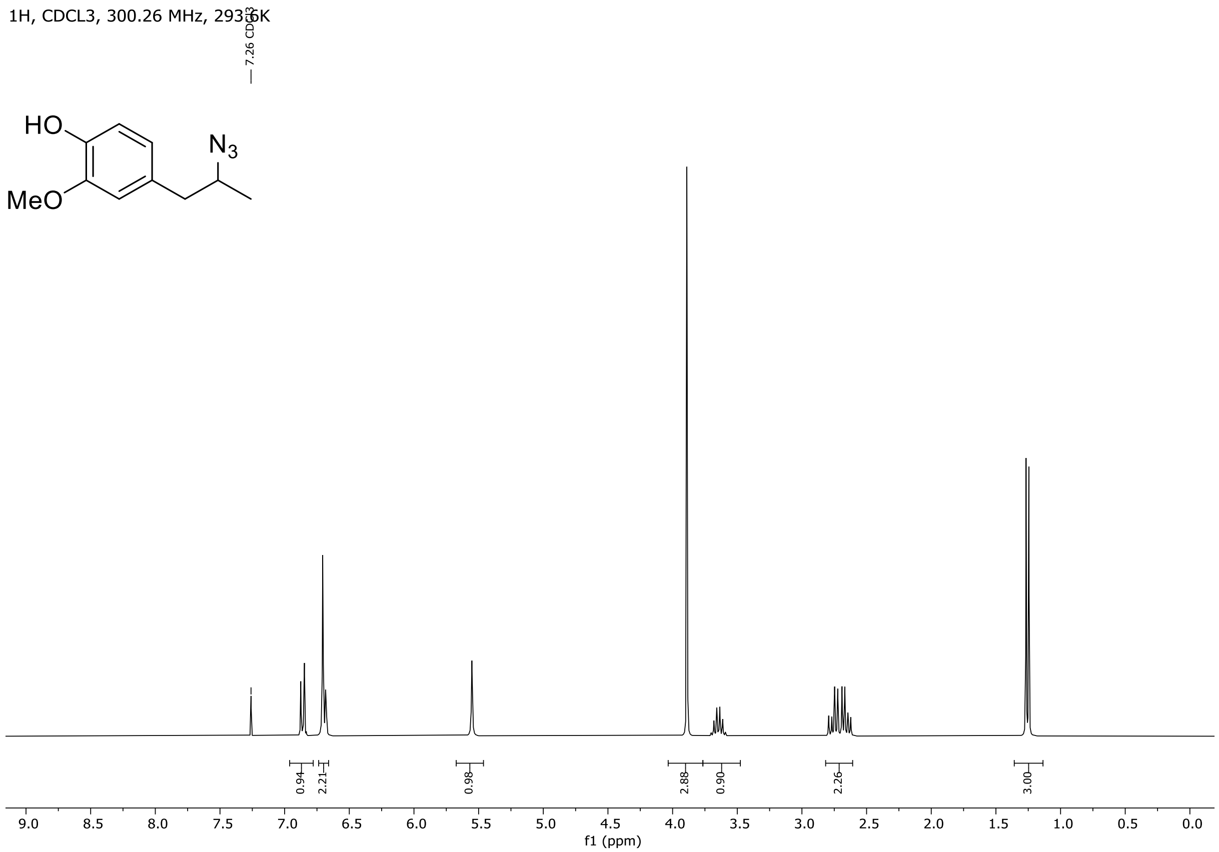

Supplementary Figure 84: ^{1}H NMR of 4-(2-Azidopropyl)-2-methoxyphenol (**8d**)

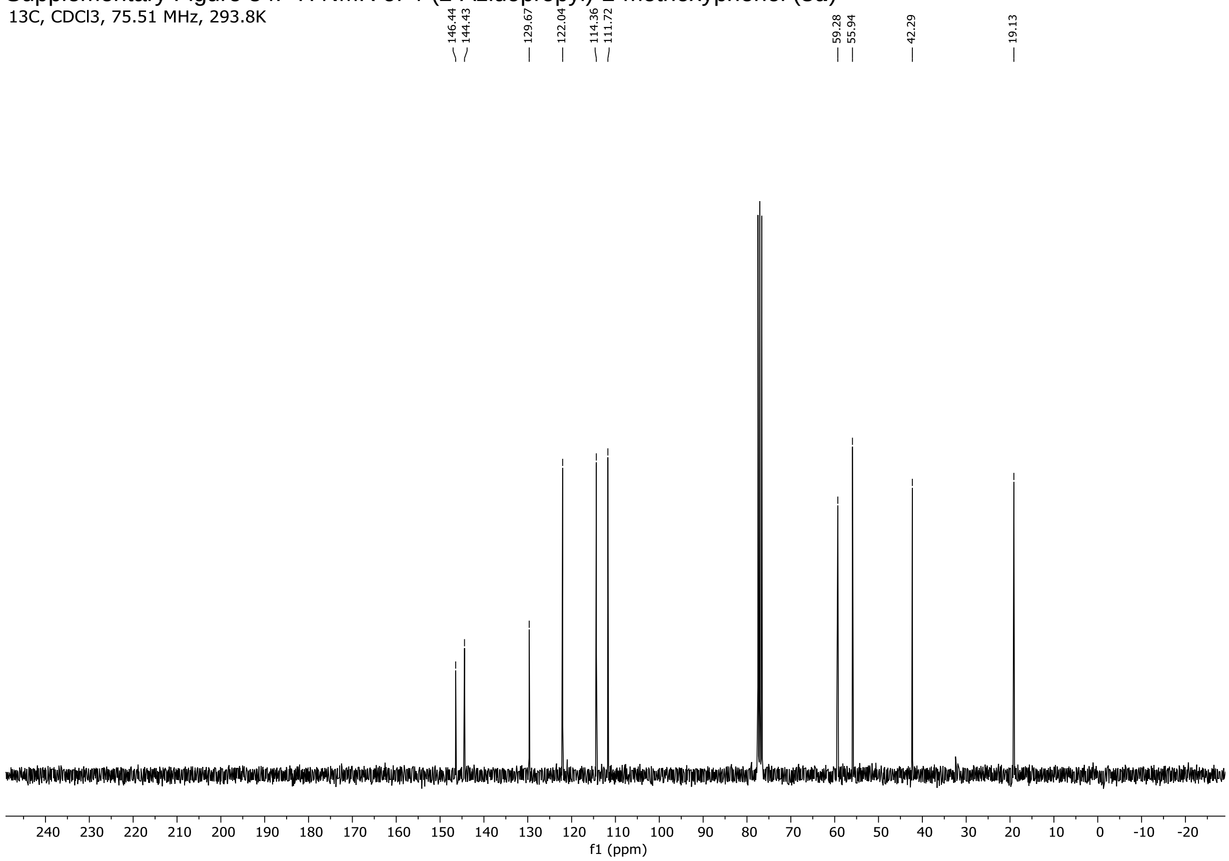

Supplementary Figure 85: ^{13}C NMR of 4-(2-Azidopropyl)-2-methoxyphenol (**8d**)

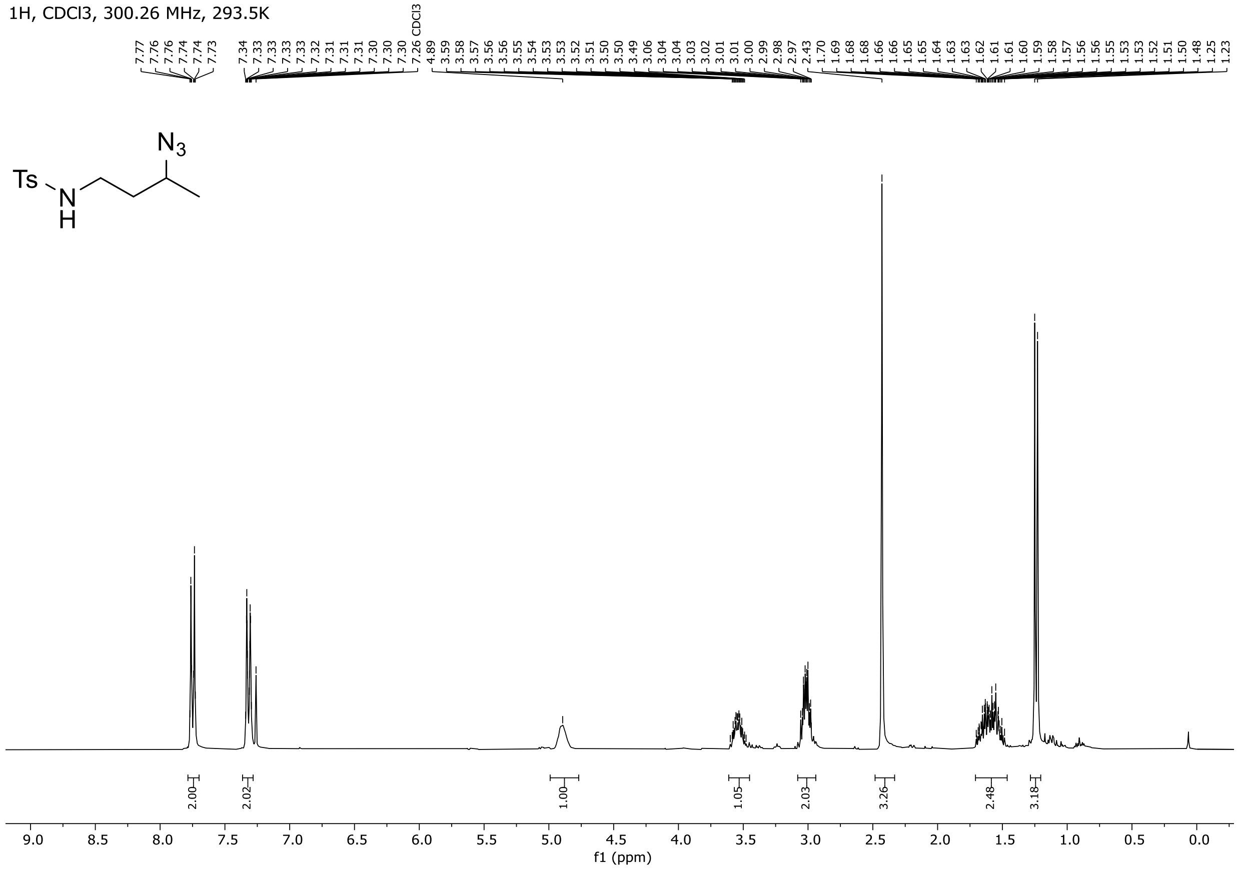

Supplementary Figure 86: ^{1}H NMR of *N*-(3-Azidobutyl)-4-methylbenzenesulfonamide (**8e**)

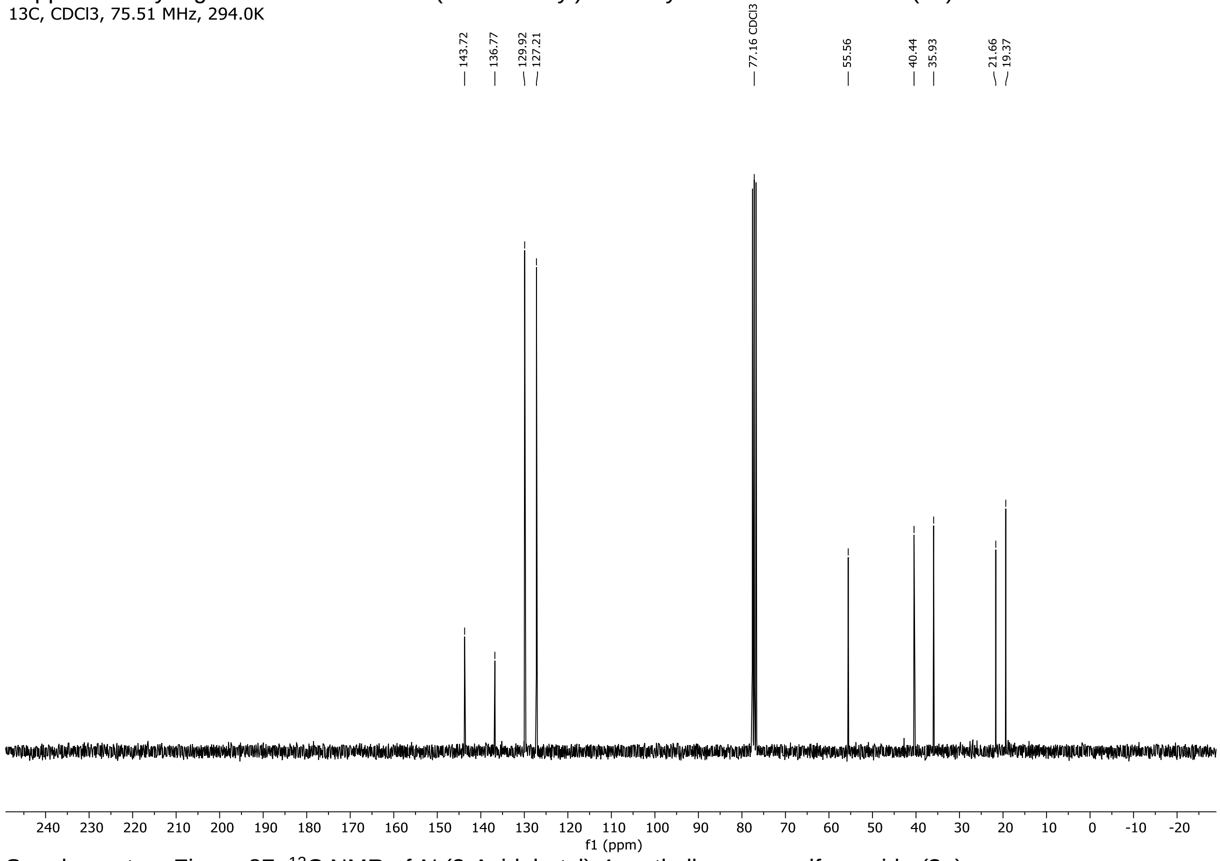

Supplementary Figure 87: ^{13}C NMR of *N*-(3-Azidobutyl)-4-methylbenzenesulfonamide (**8e**)

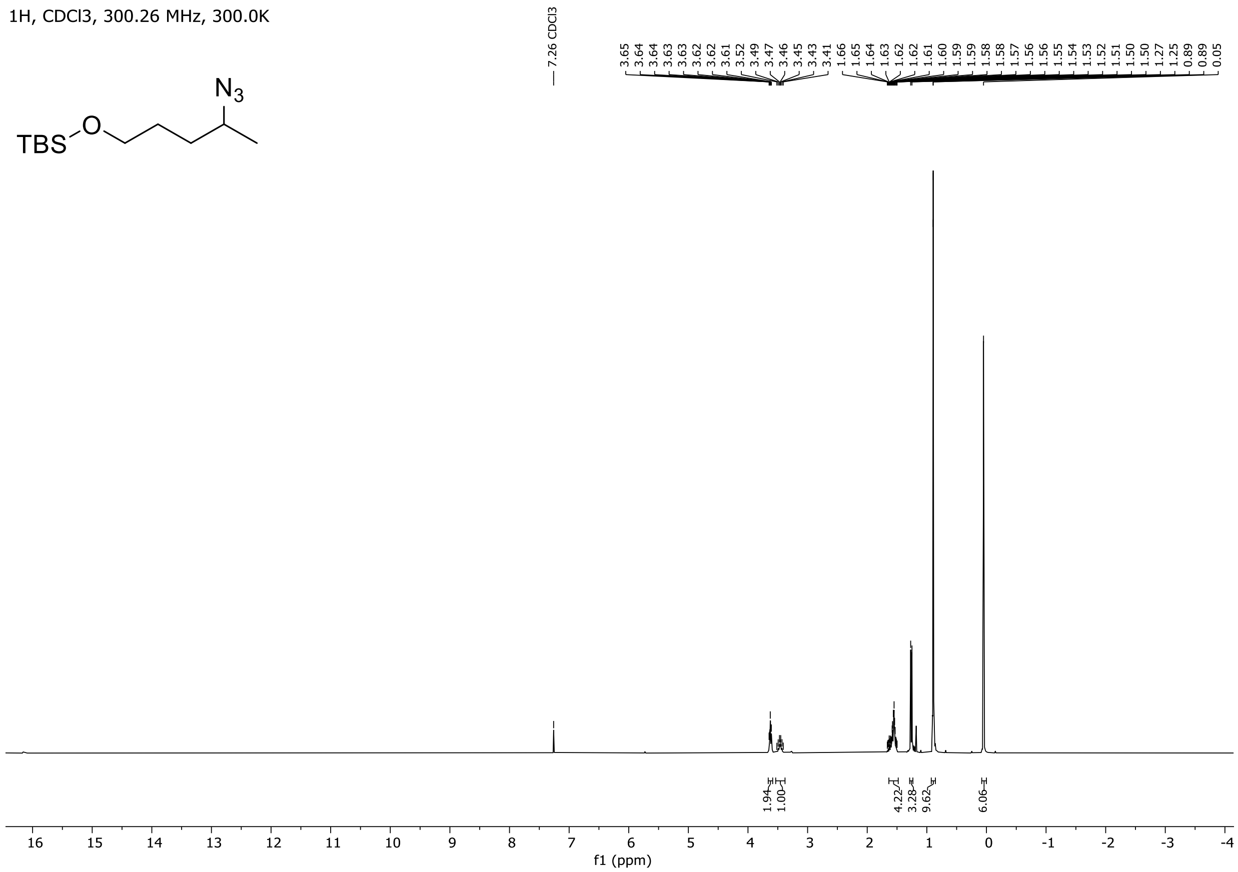

Supplementary Figure 88: ^{1}H NMR of ((4-Azidopentyl)oxy)(*tert*-butyl)dimethylsilane (**8f**)

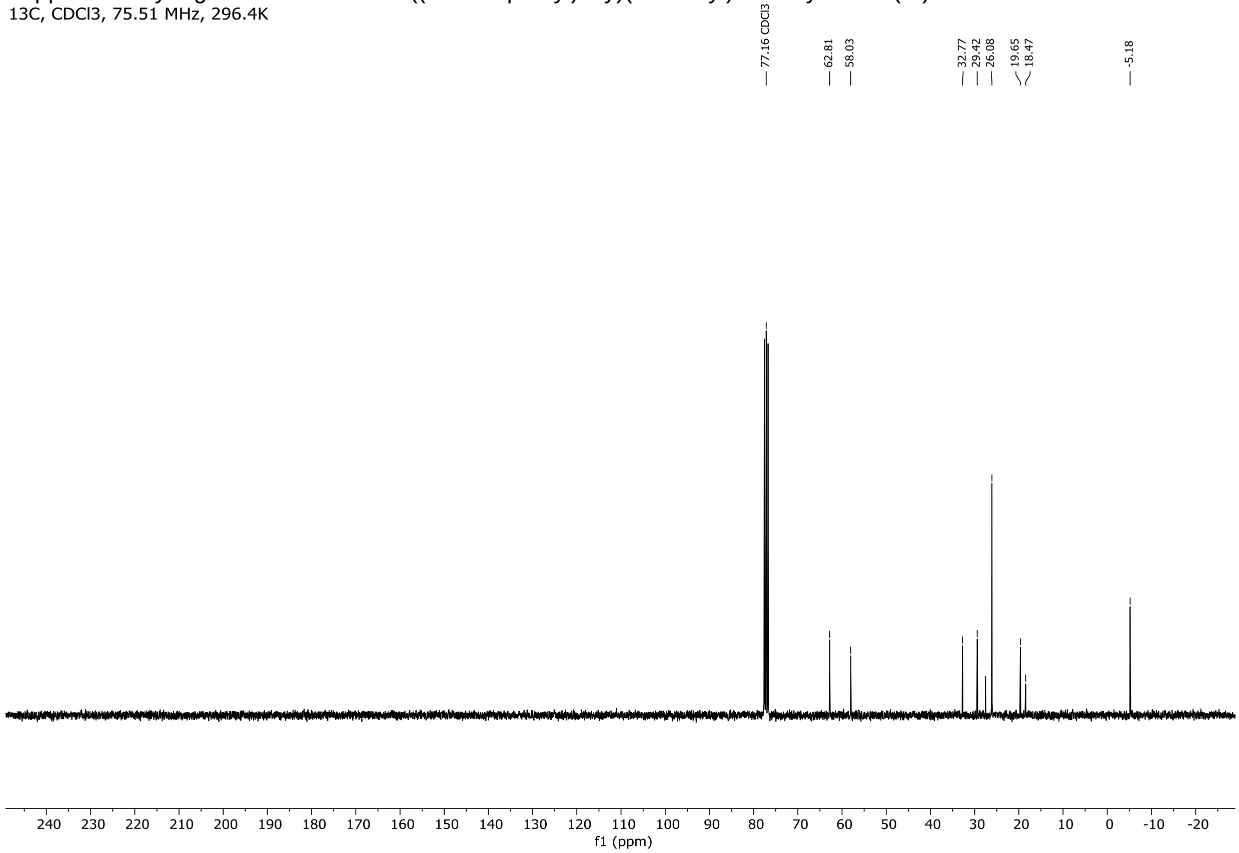

Supplementary Figure 89: ^{13}C NMR of ((4-Azidopentyl)oxy)(*tert*-butyl)dimethylsilane (**8f**)

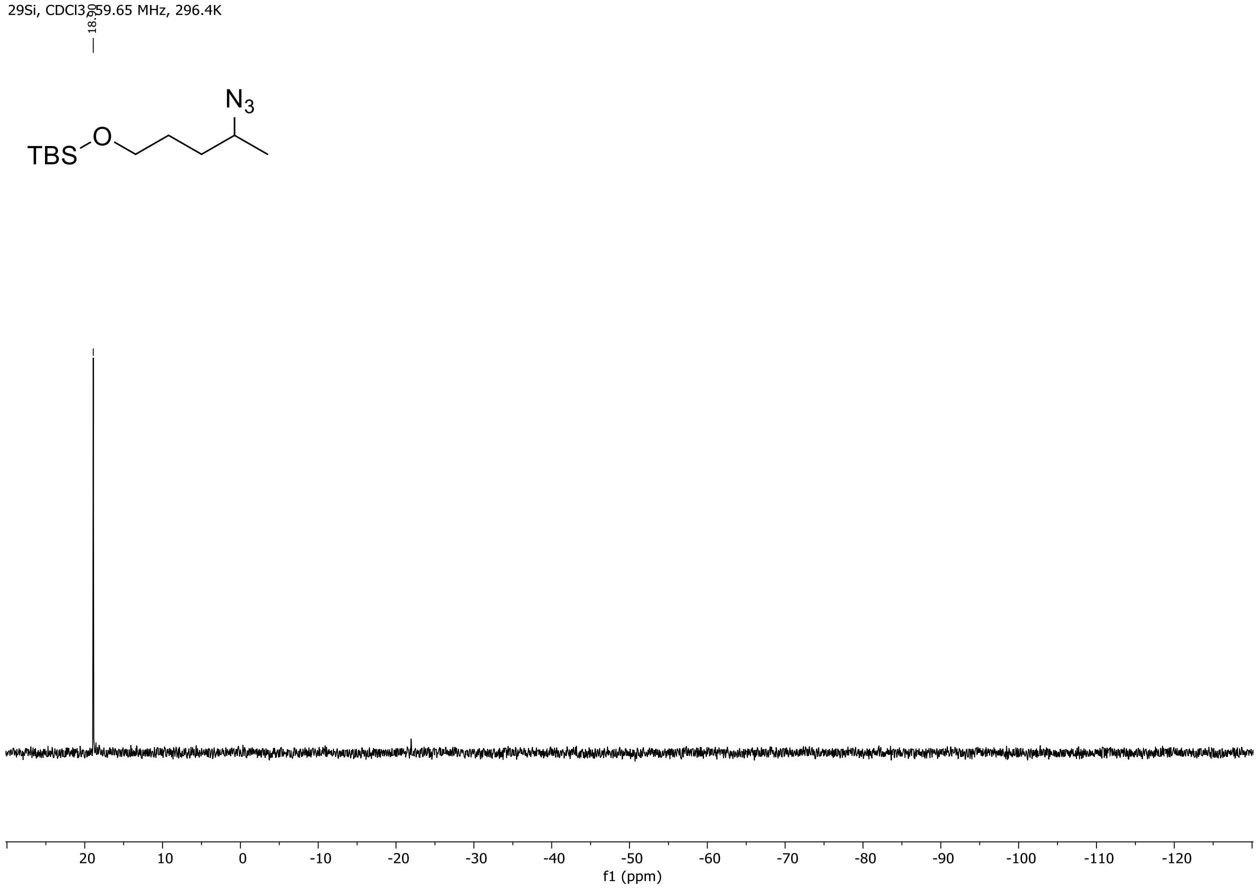

Supplementary Figure 90: ^{29}Si NMR of ((4-Azidopentyl)oxy)(*tert*-butyl)dimethylsilane (**8f**)

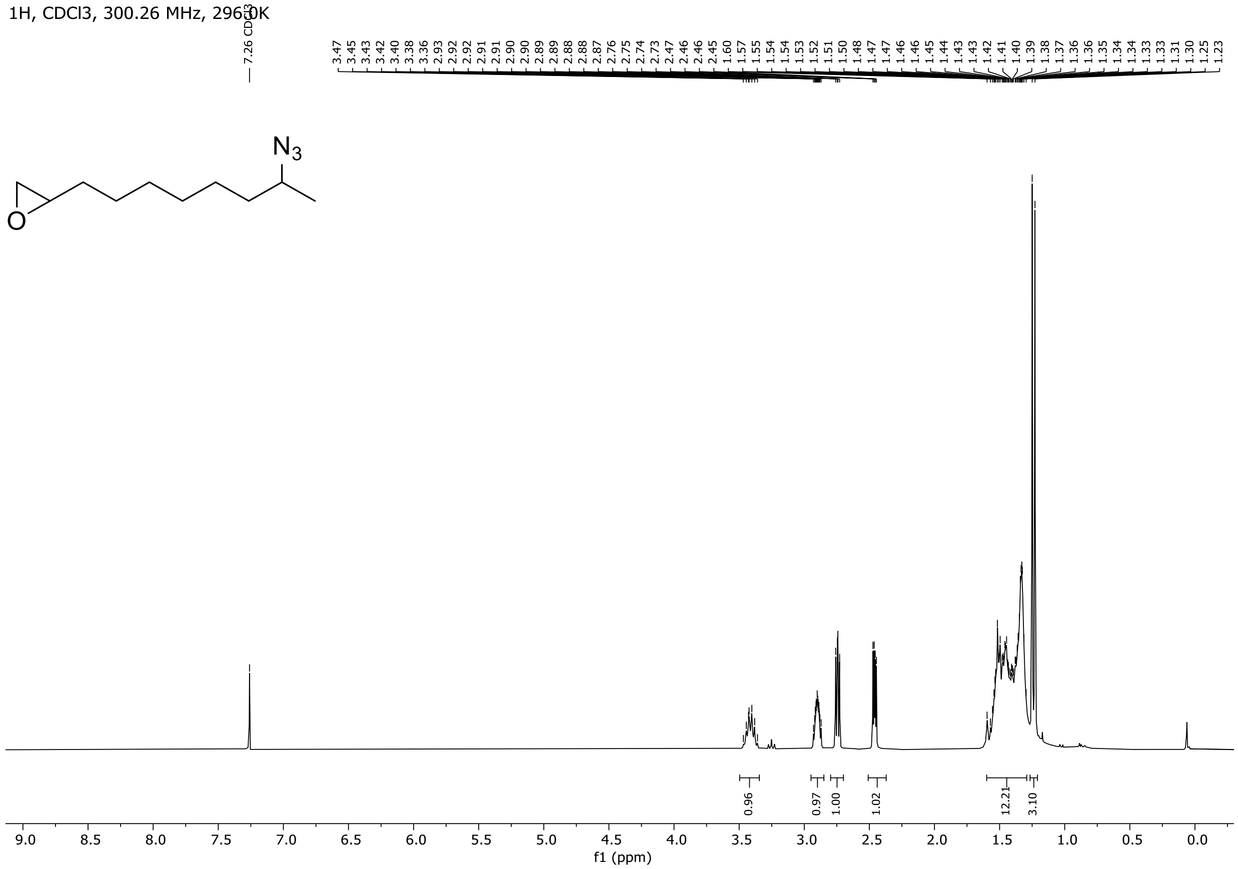

Supplementary Figure 91: ^{1}H NMR of 2-(7-azidooctyl)oxirane (**8g**)

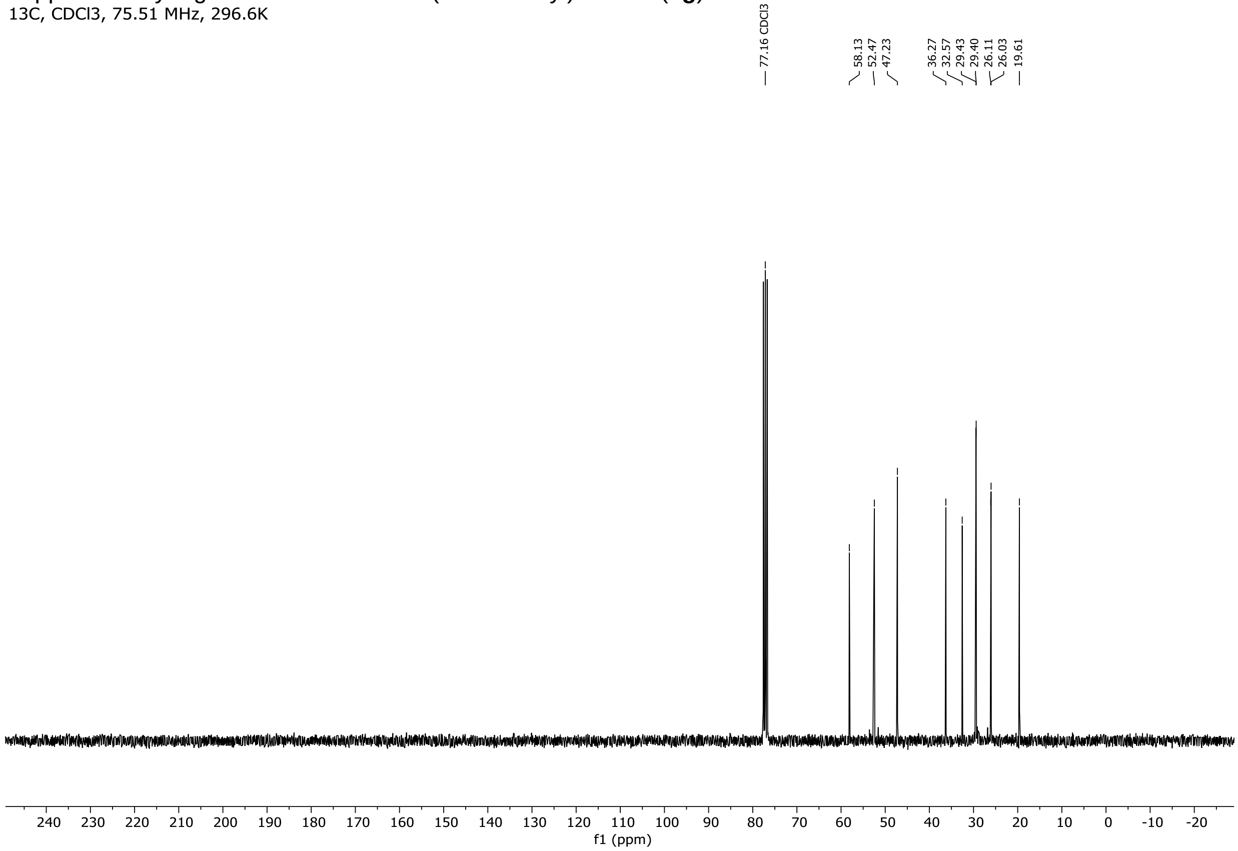

Supplementary Figure 92: ^{13}C NMR of 2-(7-azidooctyl)oxirane (**8g**)

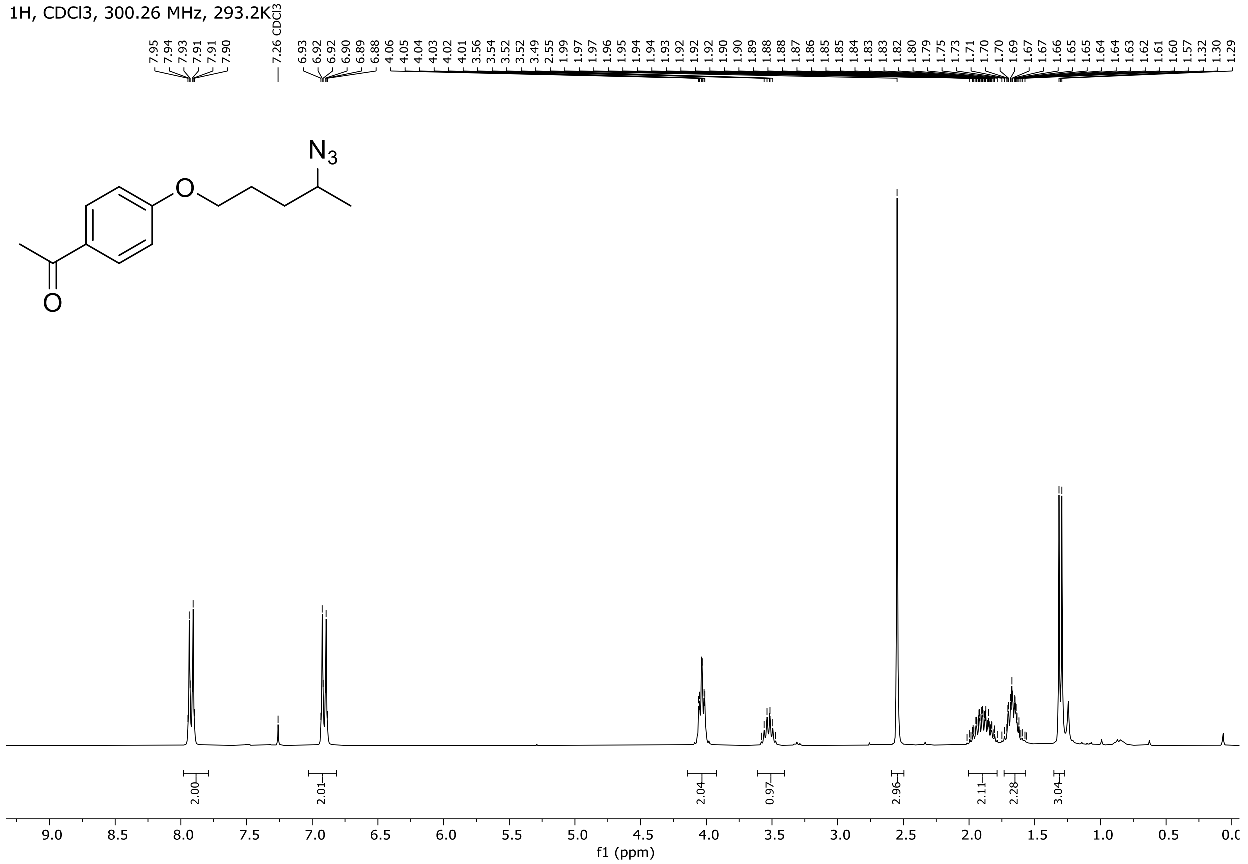

Supplementary Figure 93: ^{1}H NMR of 1-(4-((4-Azidopentyl)oxy)phenyl)ethan-1-one (**8h**)

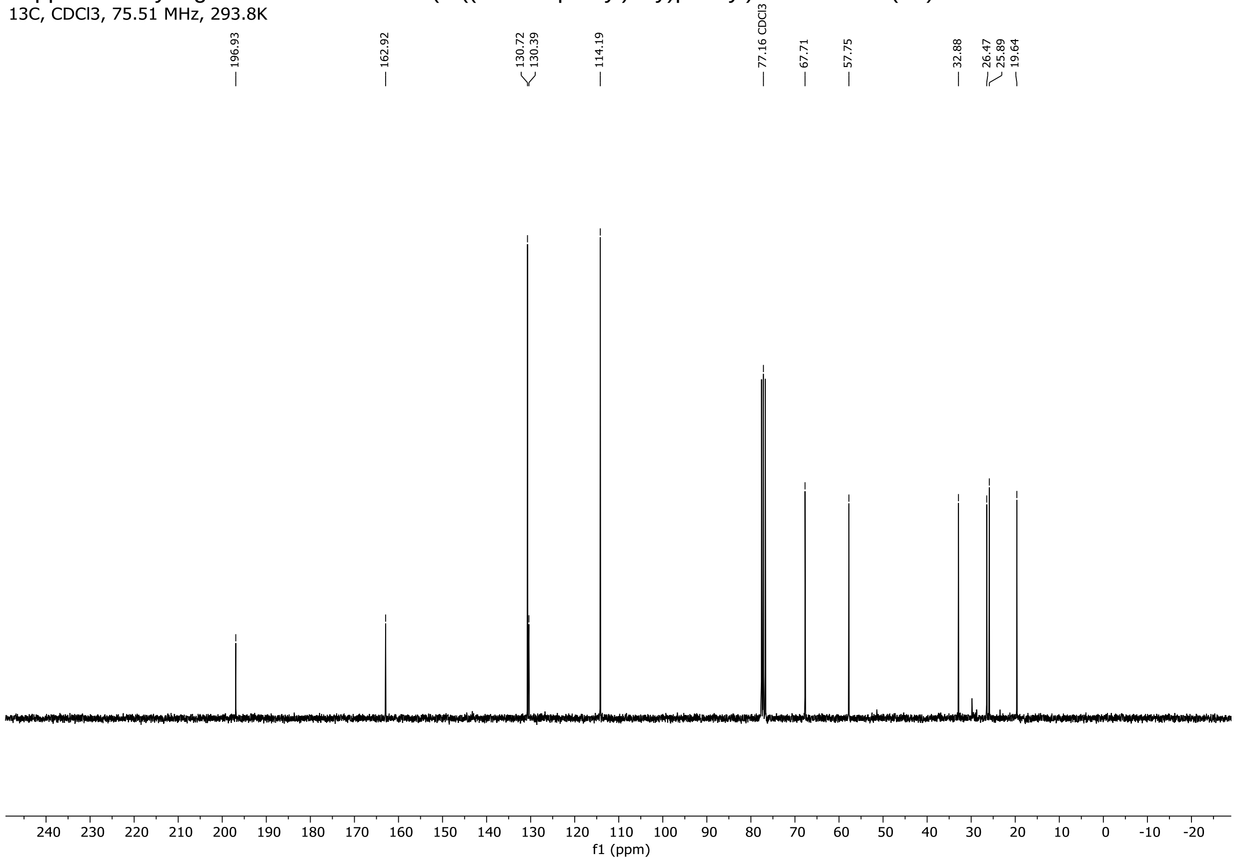

Supplementary Figure 94: ^{13}C NMR of 1-(4-((4-Azidopentyl)oxy)phenyl)ethan-1-one (**8h**)

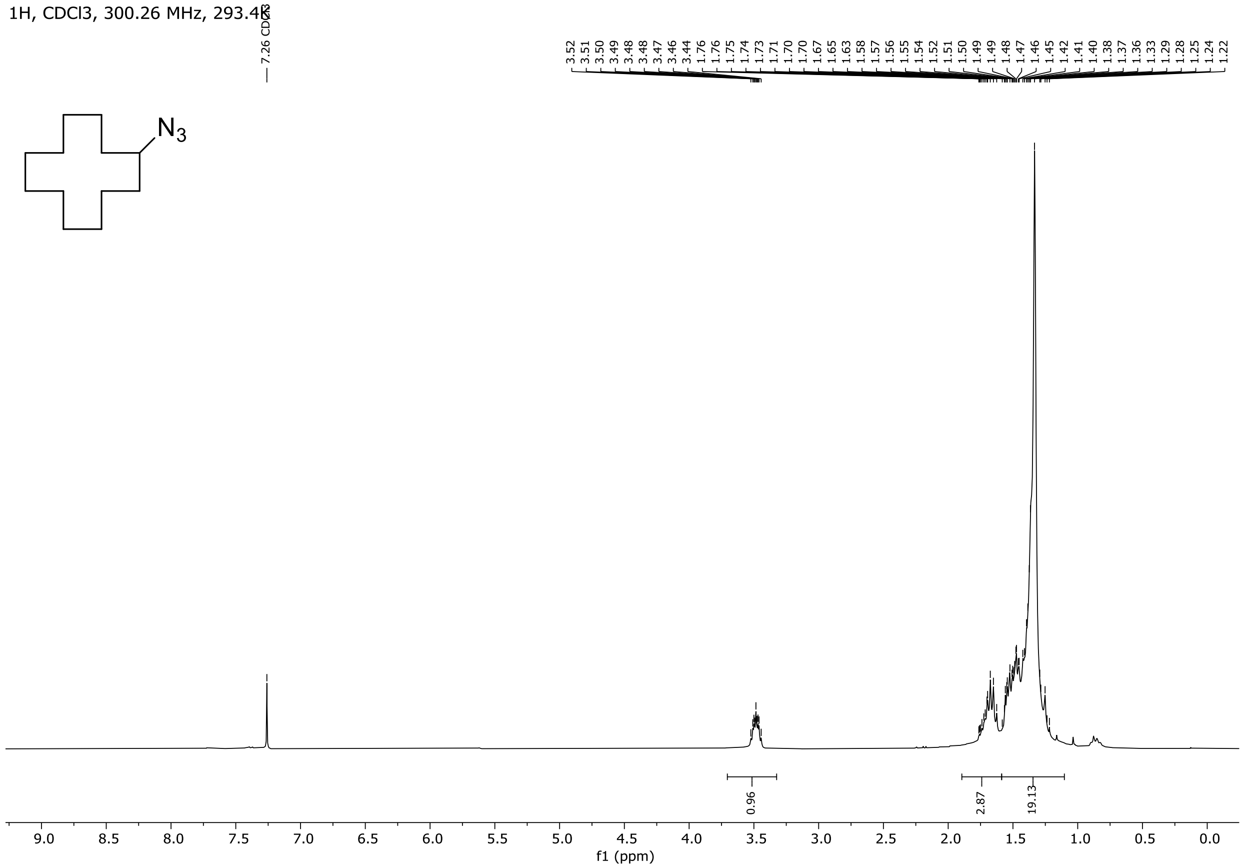

Supplementary Figure 95: ^{1}H NMR of Azidocyclododecane (**8i**)

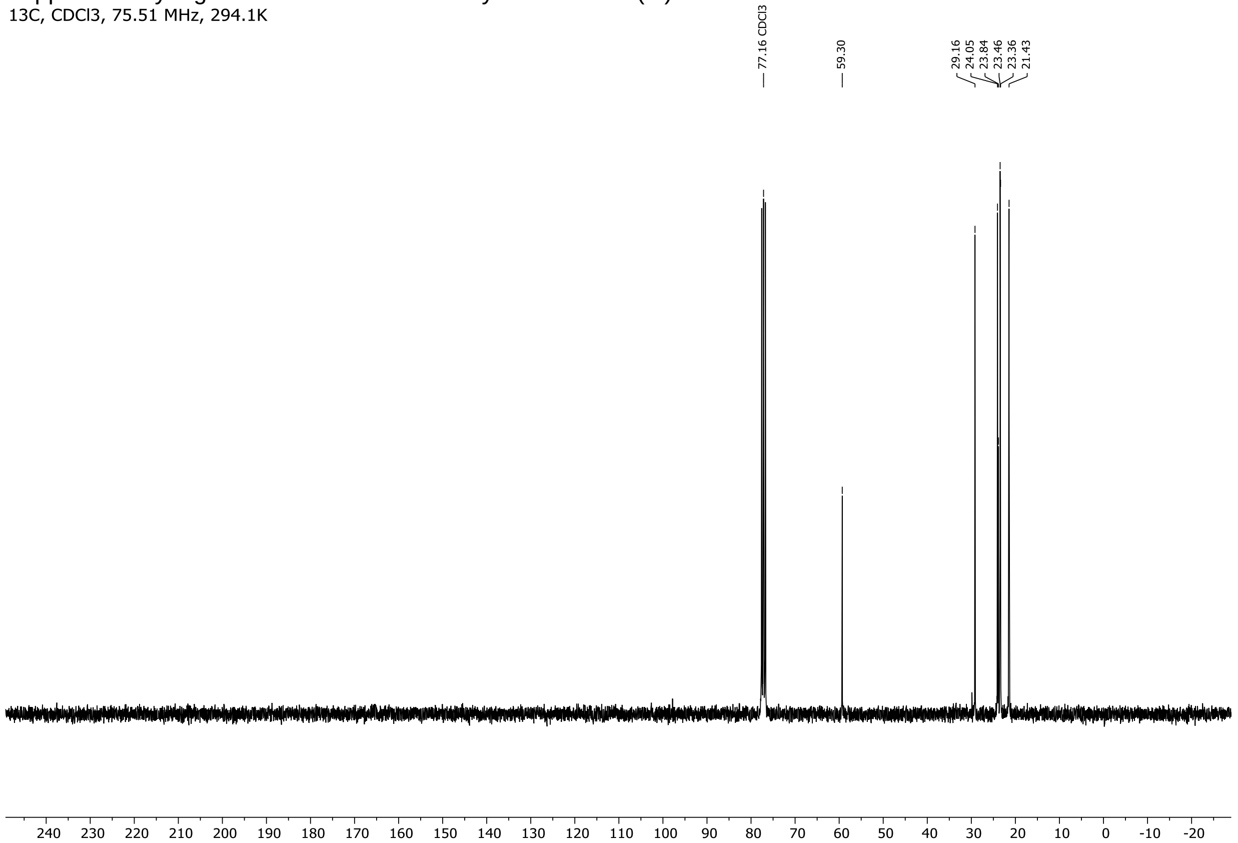

Supplementary Figure 96: ^{13}C NMR of Azidocyclododecane (**8i**)

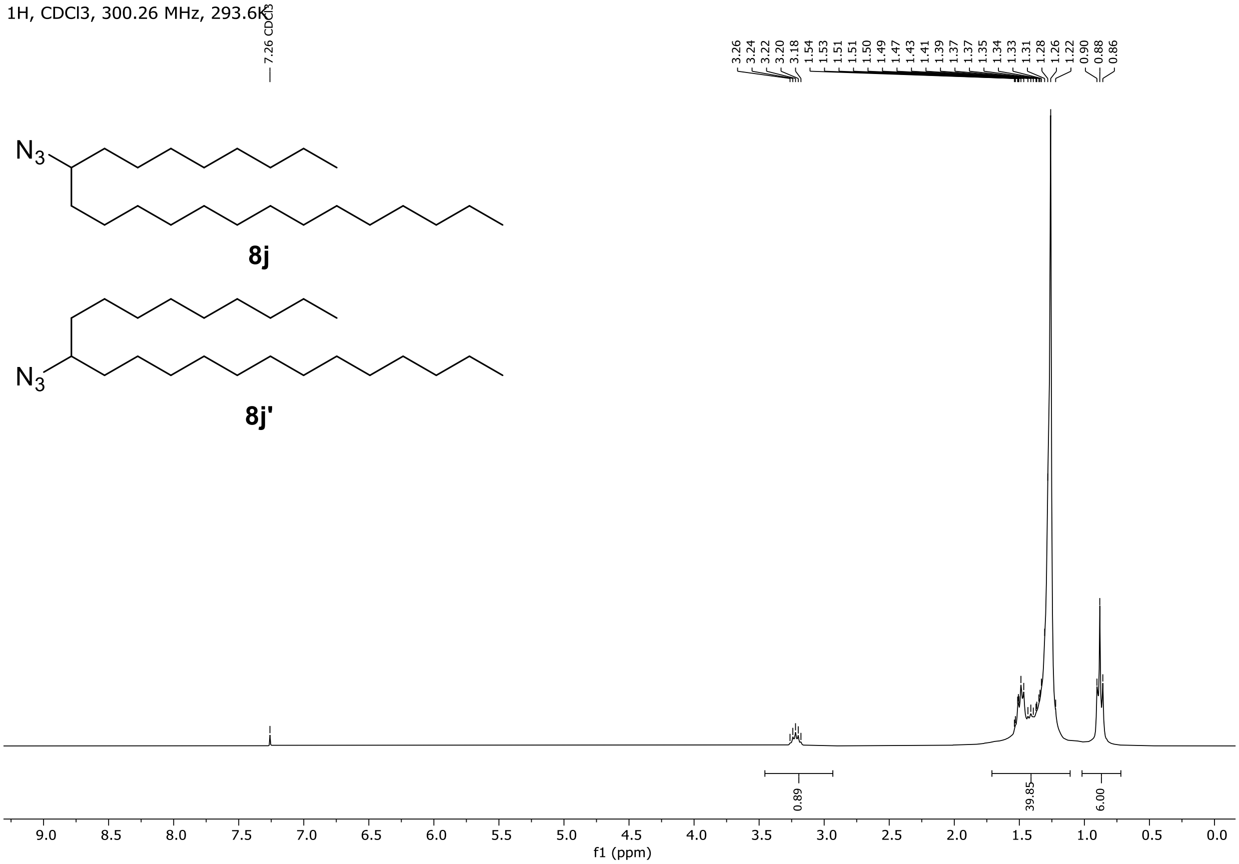

Supplementary Figure 97: ^{1}H NMR of 9-Azidotricosane (**8j**) / 10-Azidotricosane (**8j’**) 1:1

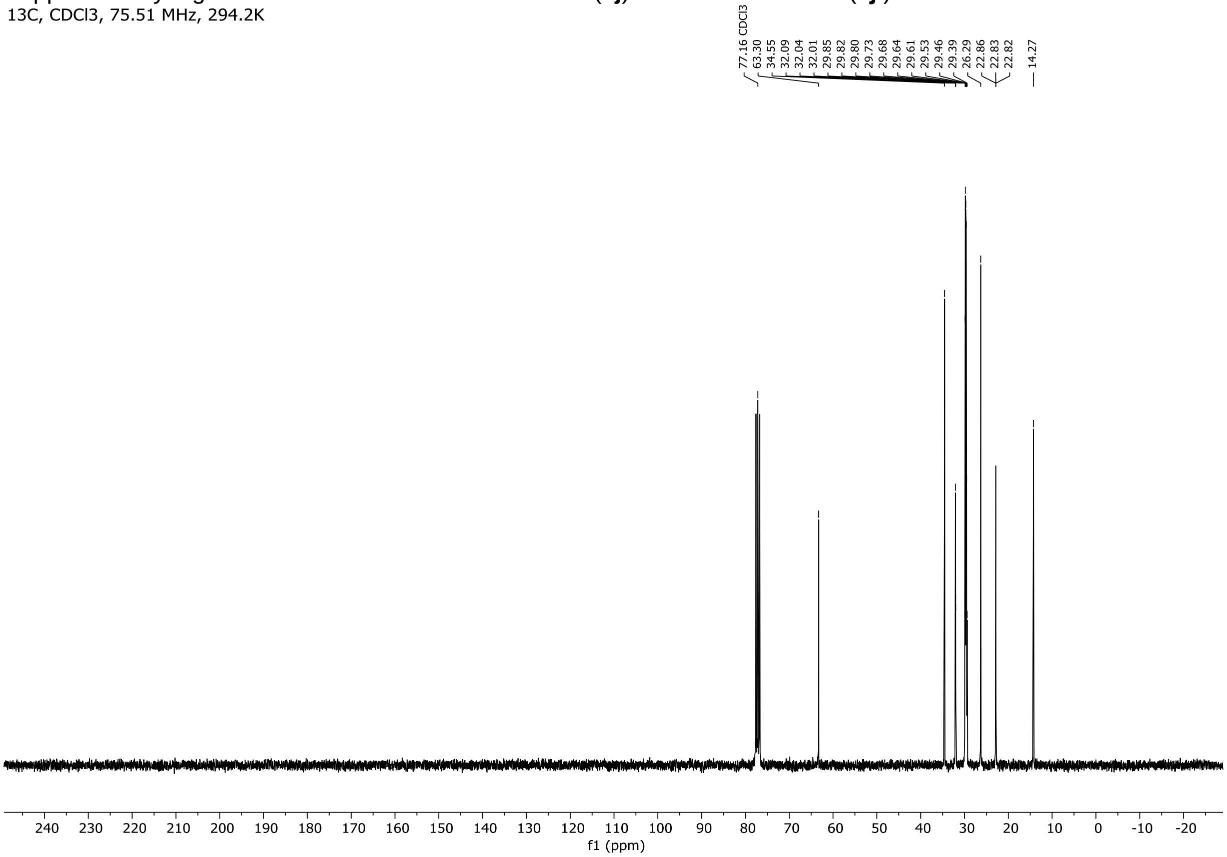

Supplementary Figure 98: ^{13}C NMR of 9-Azidotricosane (**8j**) / 10-Azidotricosane (**8j’**) 1:1

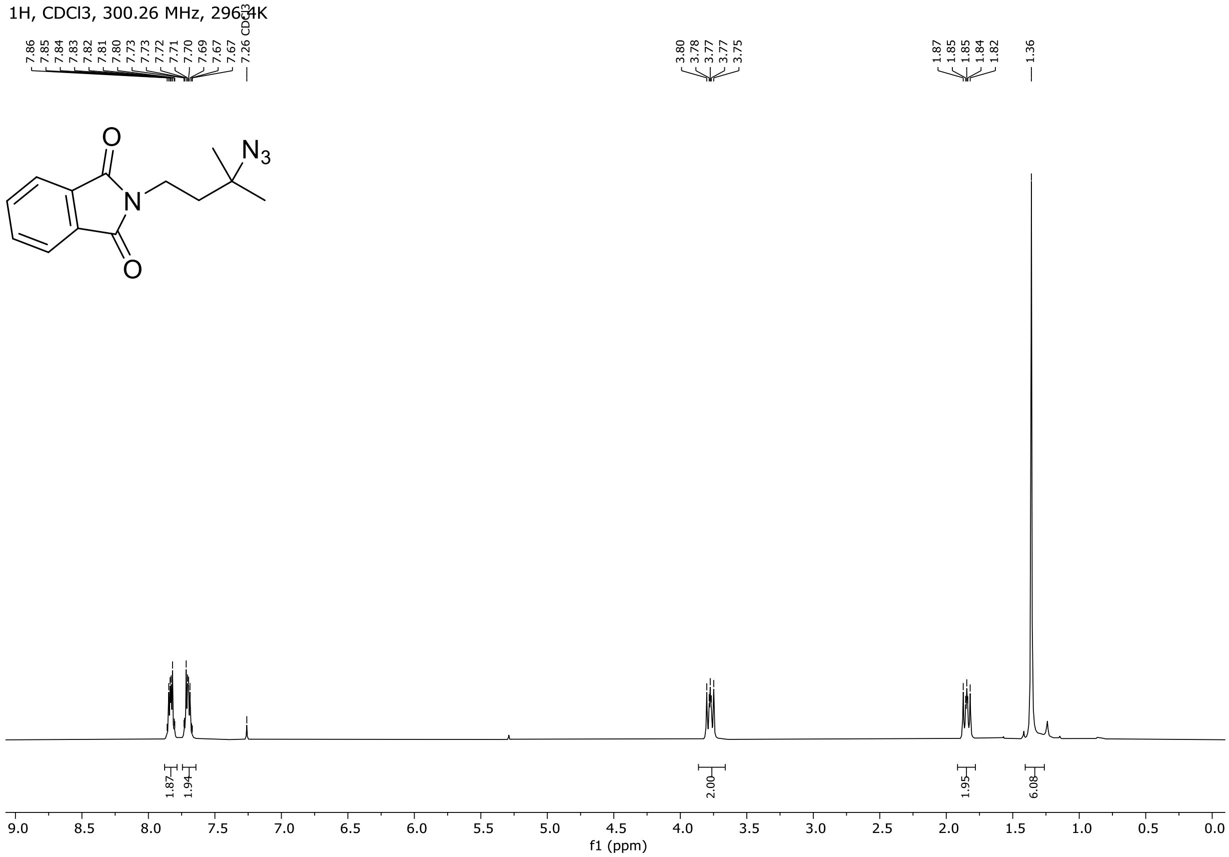

Supplementary Figure 99: ^{1}H NMR of 2-(3-Azido-3-methylbutyl)isoindoline-1,3-dione (**8k**)

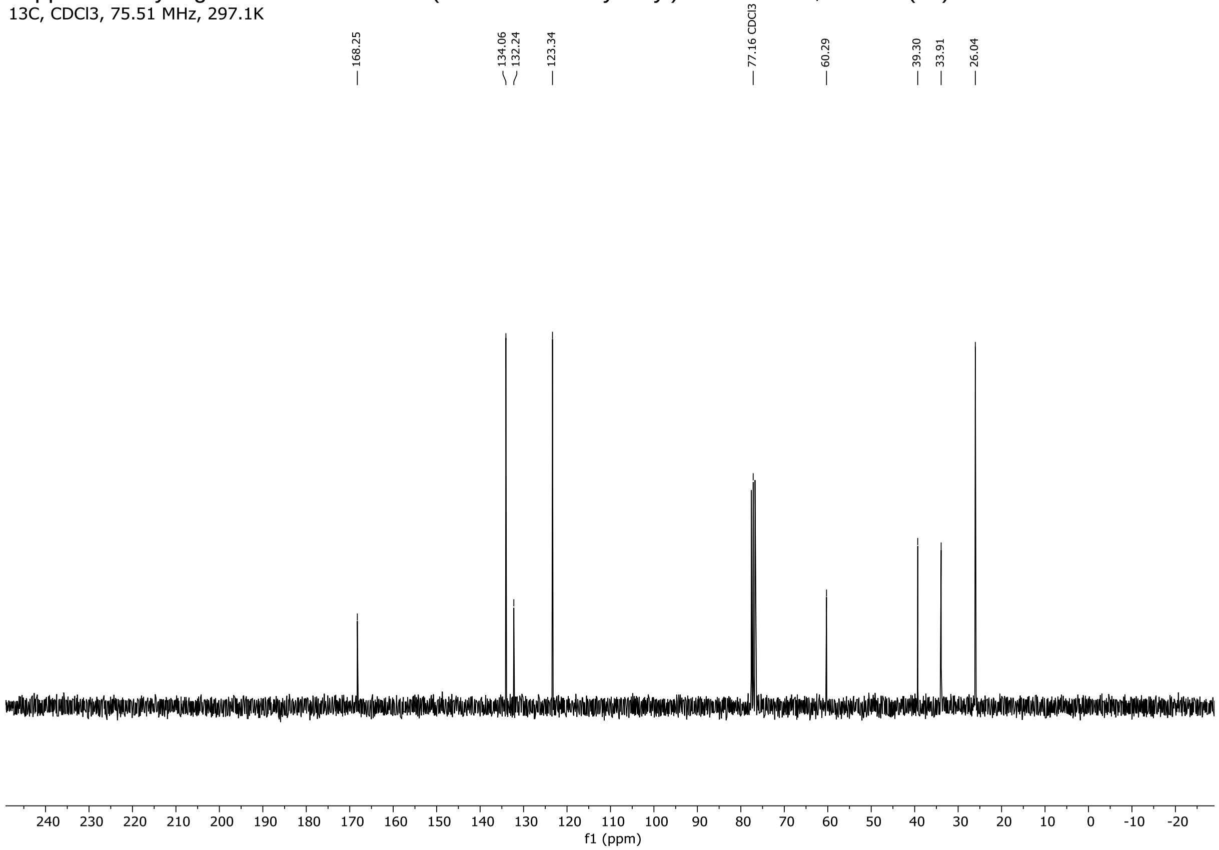

Supplementary Figure 100: ^{13}C NMR of 2-(3-Azido-3-methylbutyl)isoindoline-1,3-dione (**8k**)

^{1}H-NMR (400 MHz, $CDCl_3$, 298 K)

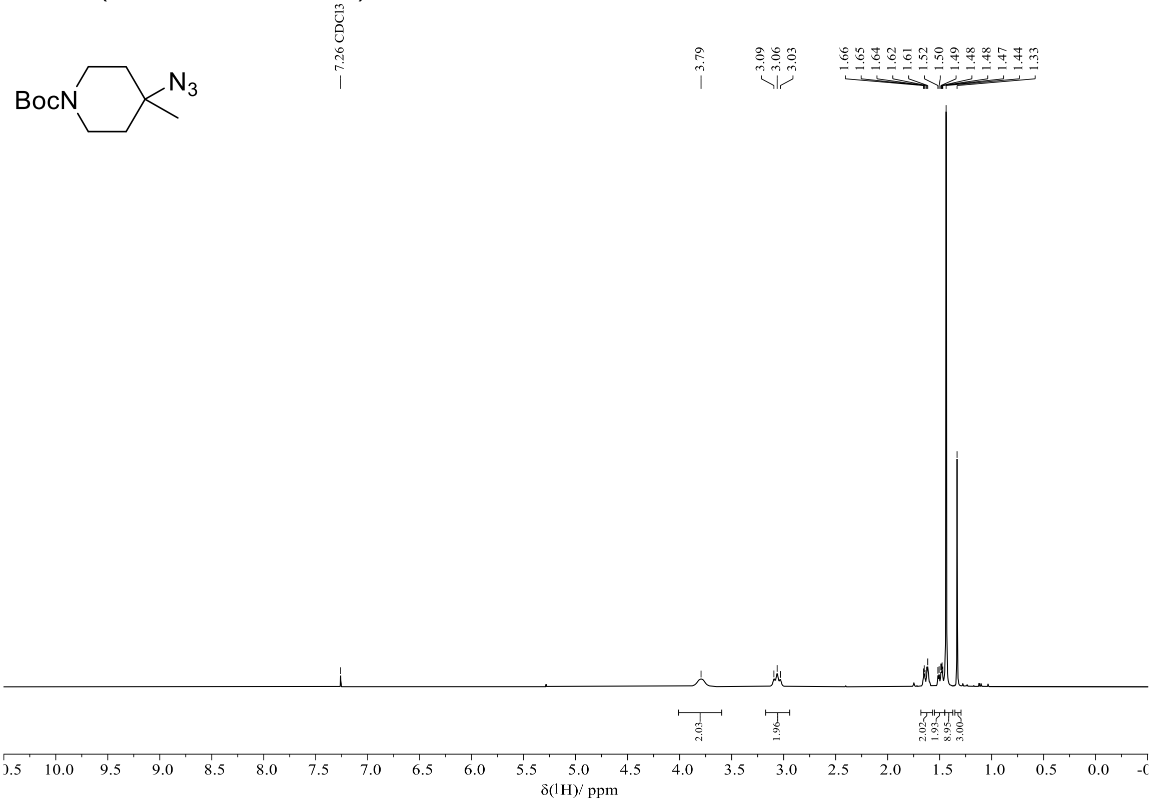

Supplementary Figure 101: ^{1}H NMR of *tert*-Butyl 4-azido-4-methylpiperidine-1-carboxylate (**8l**)

^{13}C-NMR (100 MHz, $CDCl_3$, 298 K)

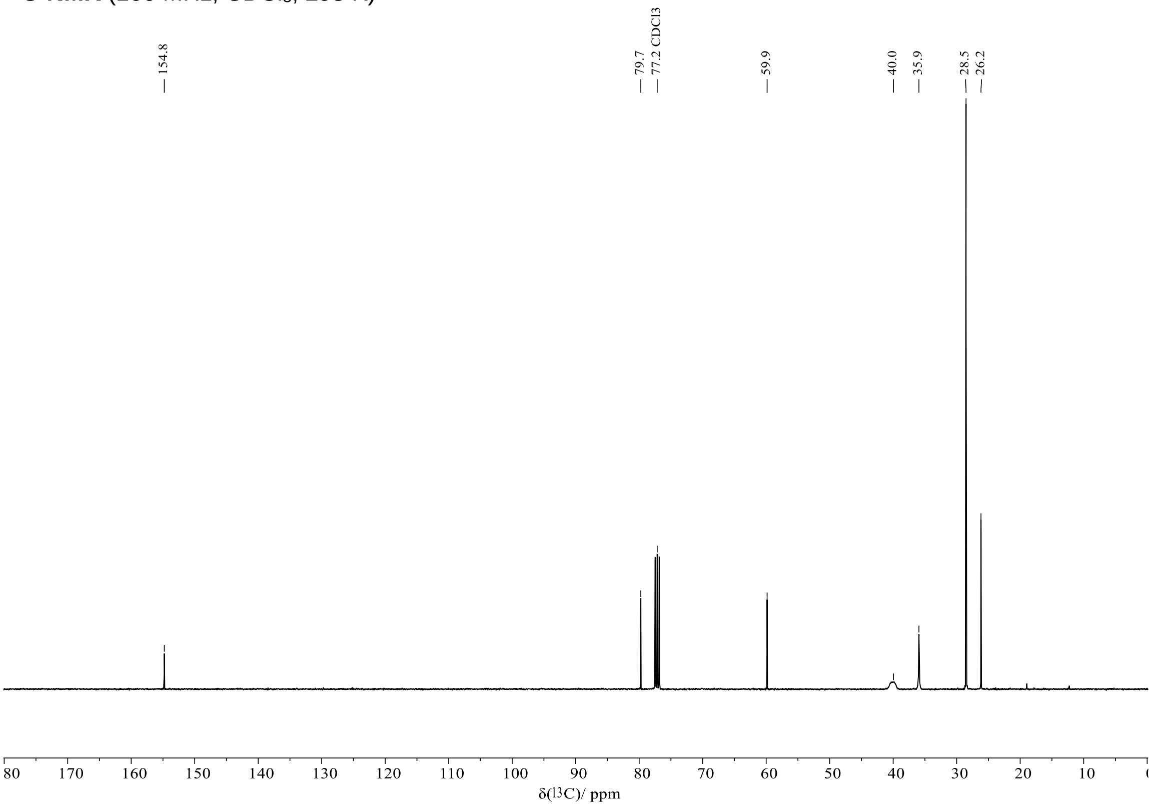

Supplementary Figure 102: ^{13}C NMR of *tert*-Butyl 4-azido-4-methylpiperidine-1-carboxylate (**8l**)

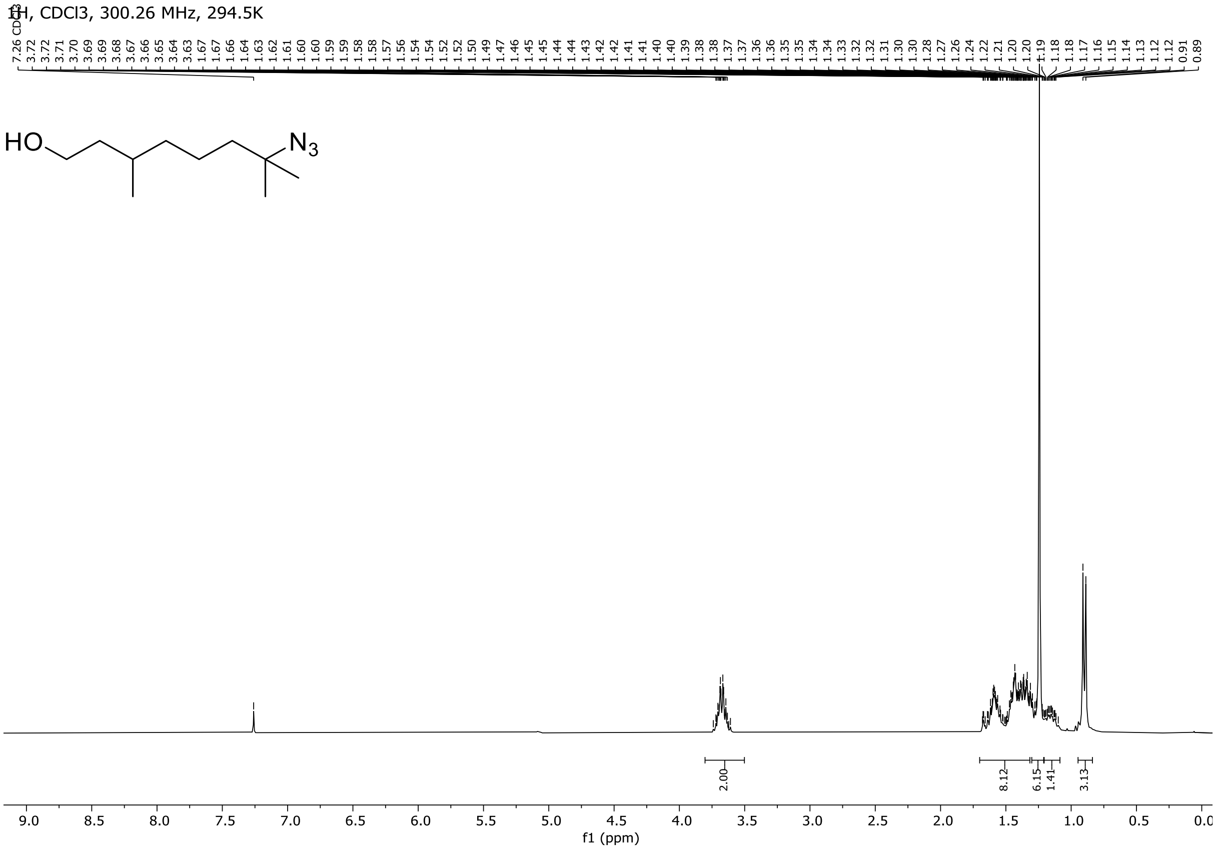

Supplementary Figure 103: ^{1}H NMR of 7-Azido-3,7-dimethyloctan-1-ol (**8m**)

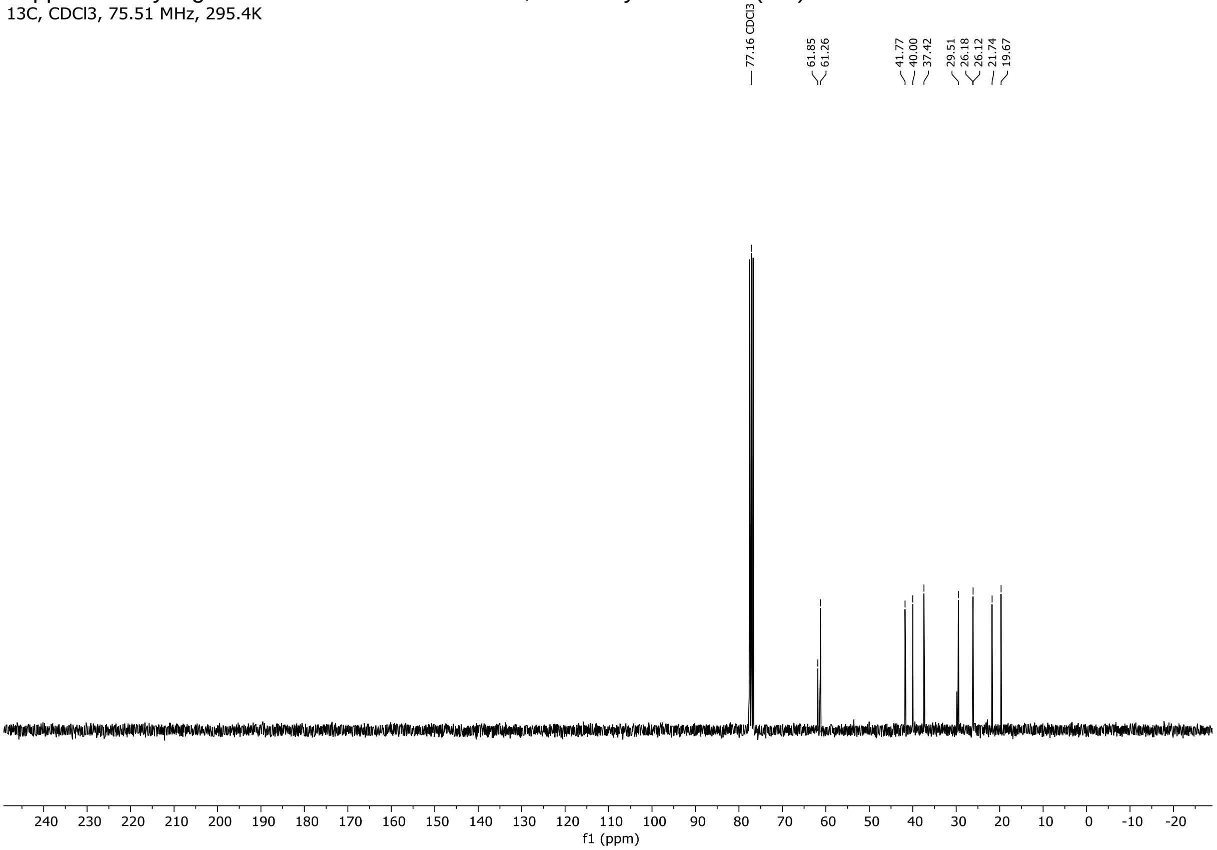

Supplementary Figure 104: ^{1}H NMR of 7-Azido-3,7-dimethyloctan-1-ol (**8m**)

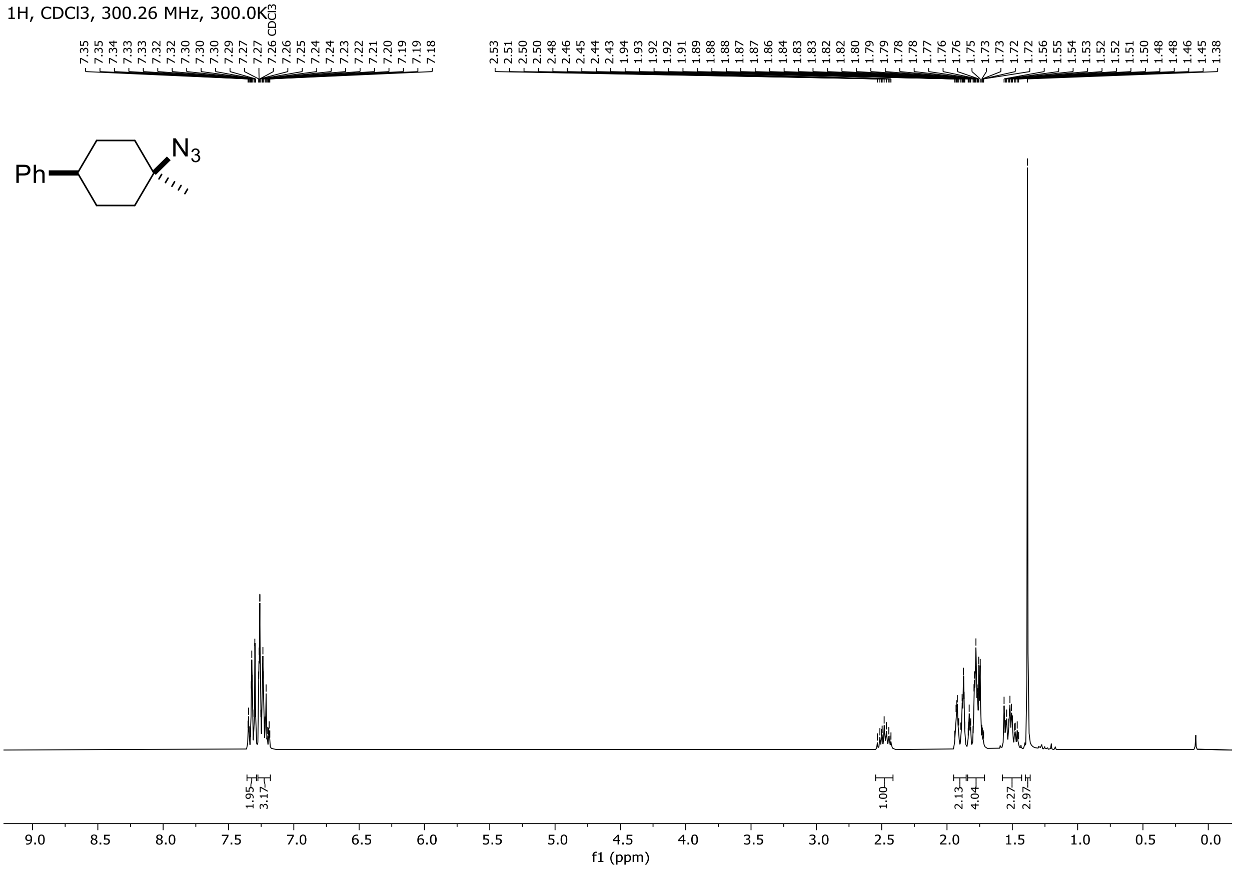

Supplementary Figure 105: ^{1}H NMR of (4-Azido-4-methylcyclohexyl)benzene (**8n**)

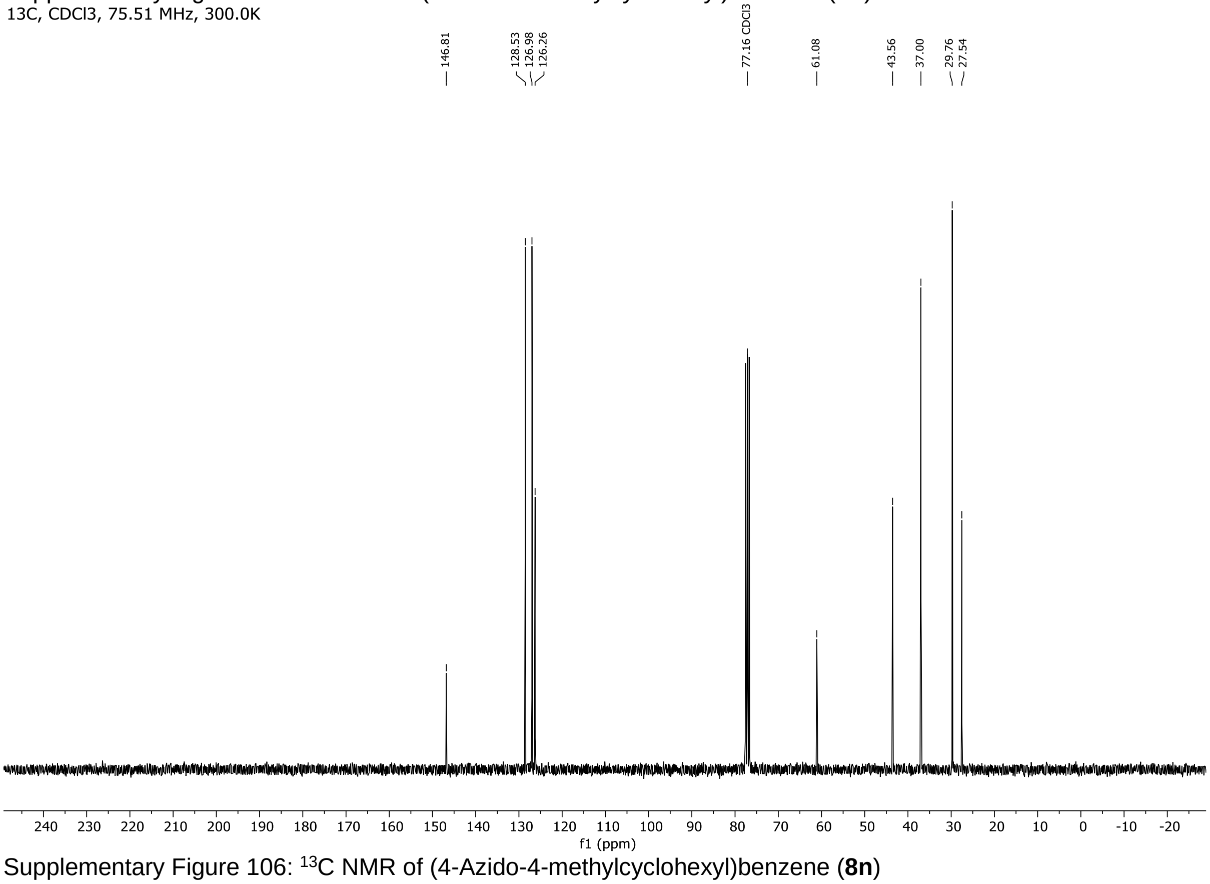

Supplementary Figure 106: ^{13}C NMR of (4-Azido-4-methylcyclohexyl)benzene (**8n**)

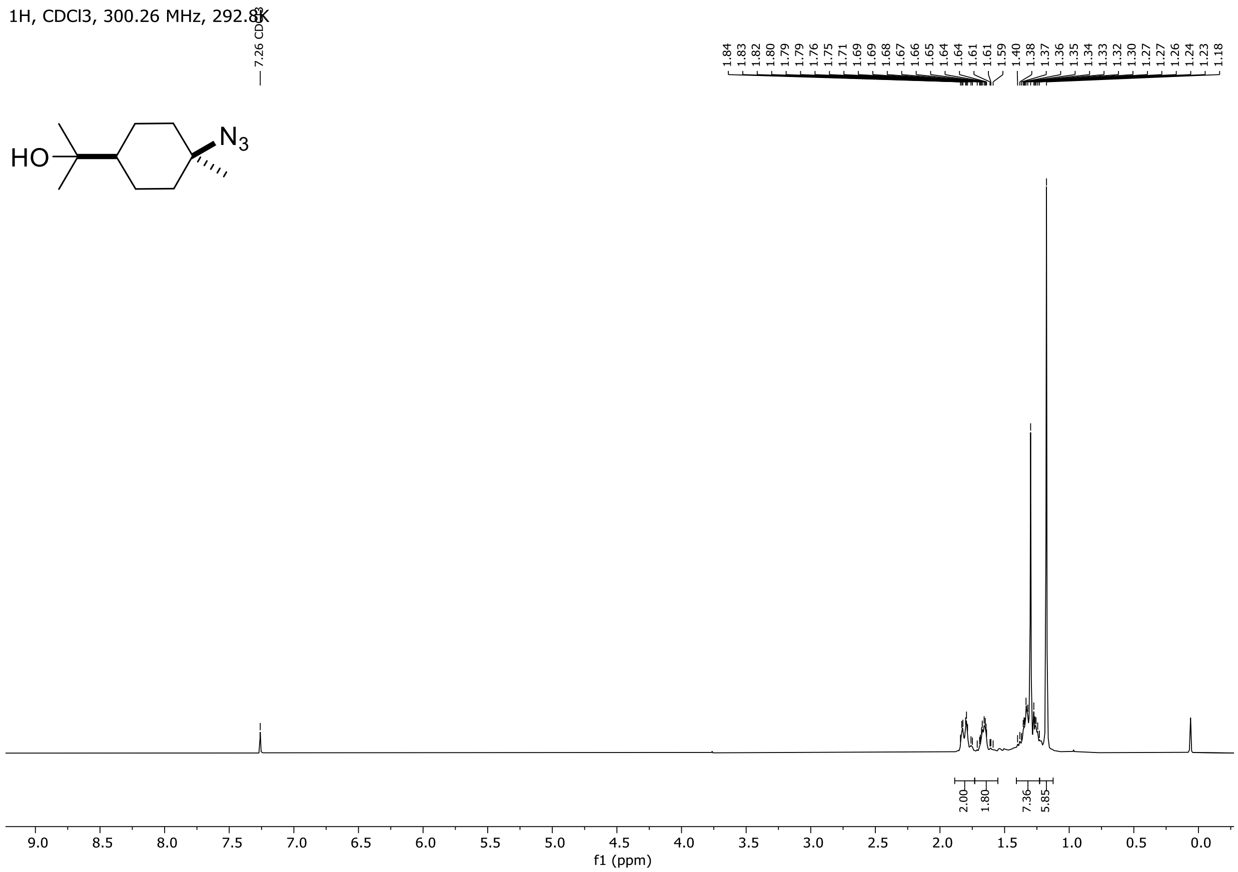

Supplementary Figure 107: ^{1}H NMR of 2-(4-Azido-4-methylcyclohexyl)propan-2-ol (**8o**)

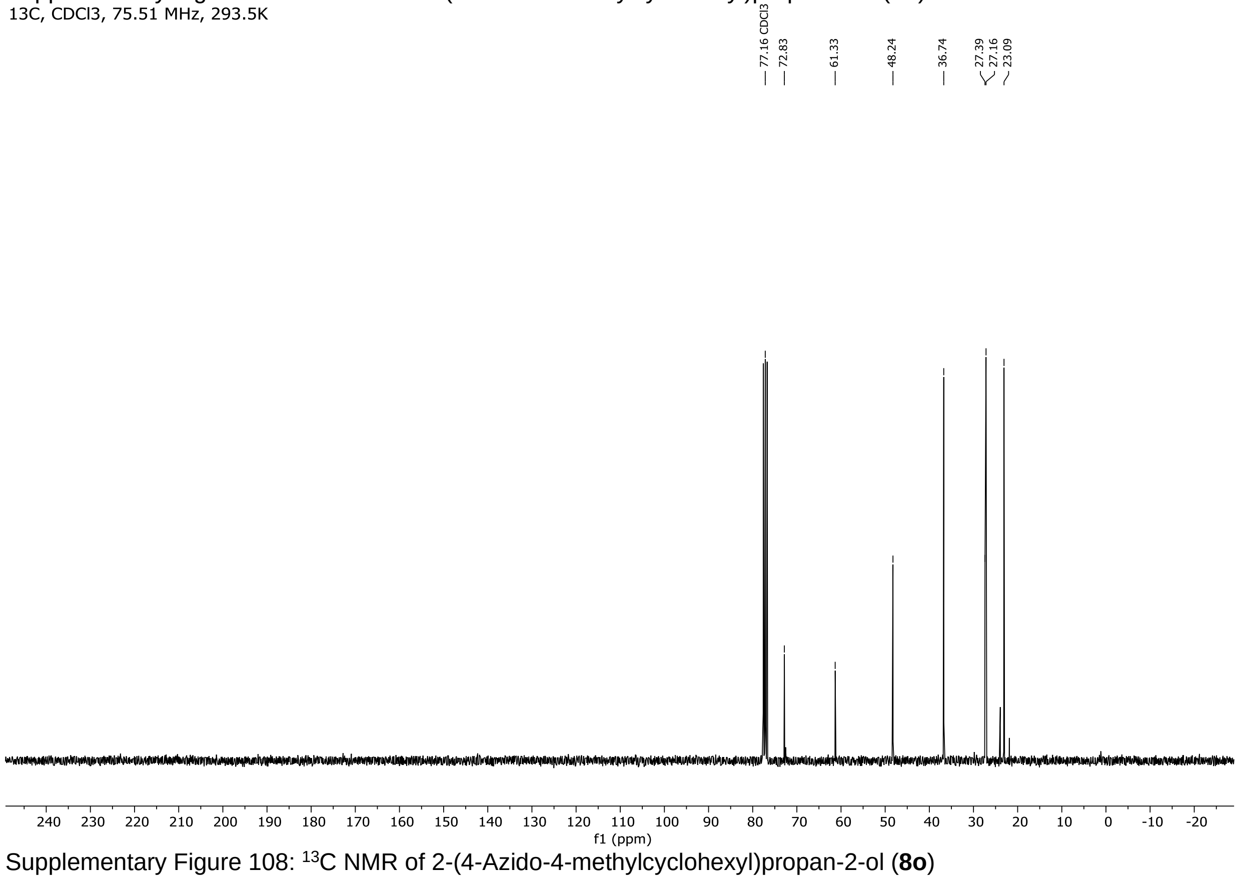

Supplementary Figure 108: ^{13}C NMR of 2-(4-Azido-4-methylcyclohexyl)propan-2-ol (**8o**)

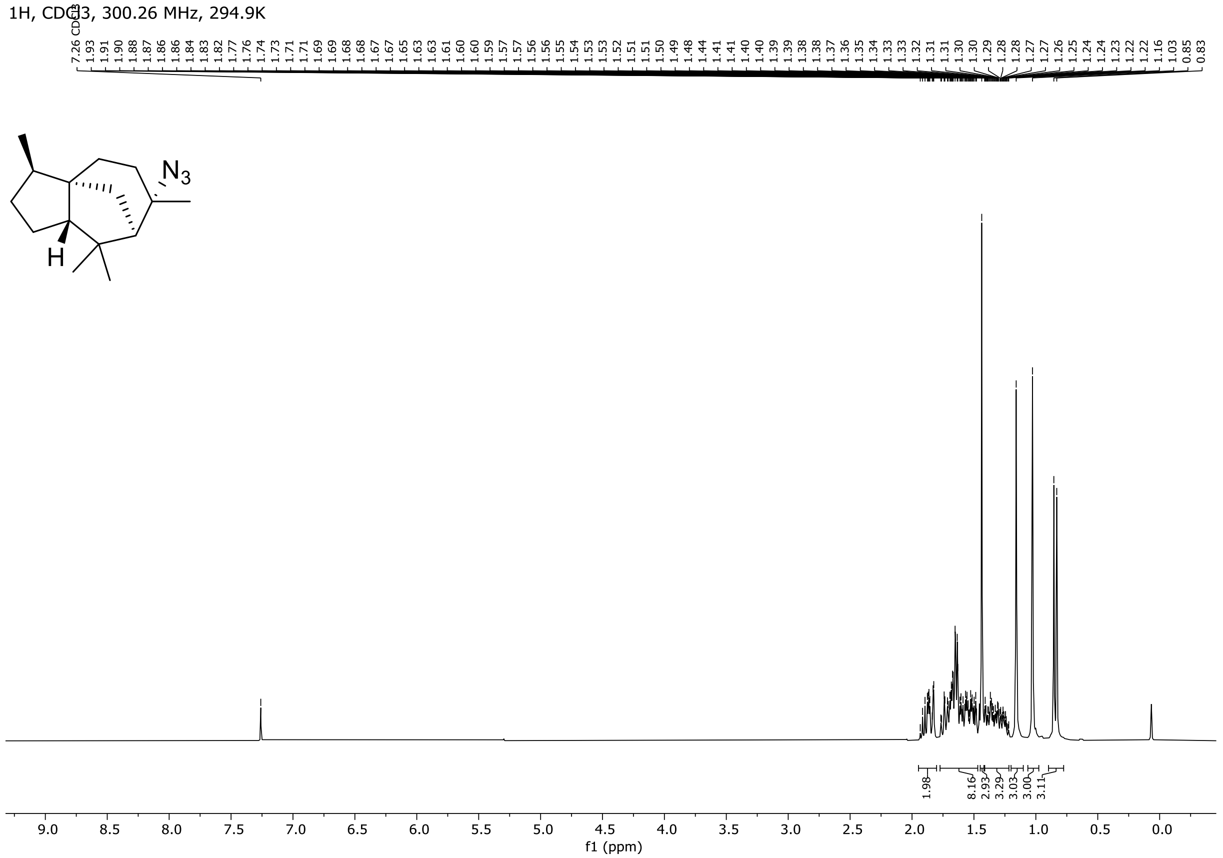

Supplementary Figure 109: ^{1}H NMR of (3*R*,3a*S*,6*S*,7*R*,8a*S*)-6-Azido-3,6,8,8-tetramethyloctahydro-1*H*-3a,7-methanoazulene (**8p**)

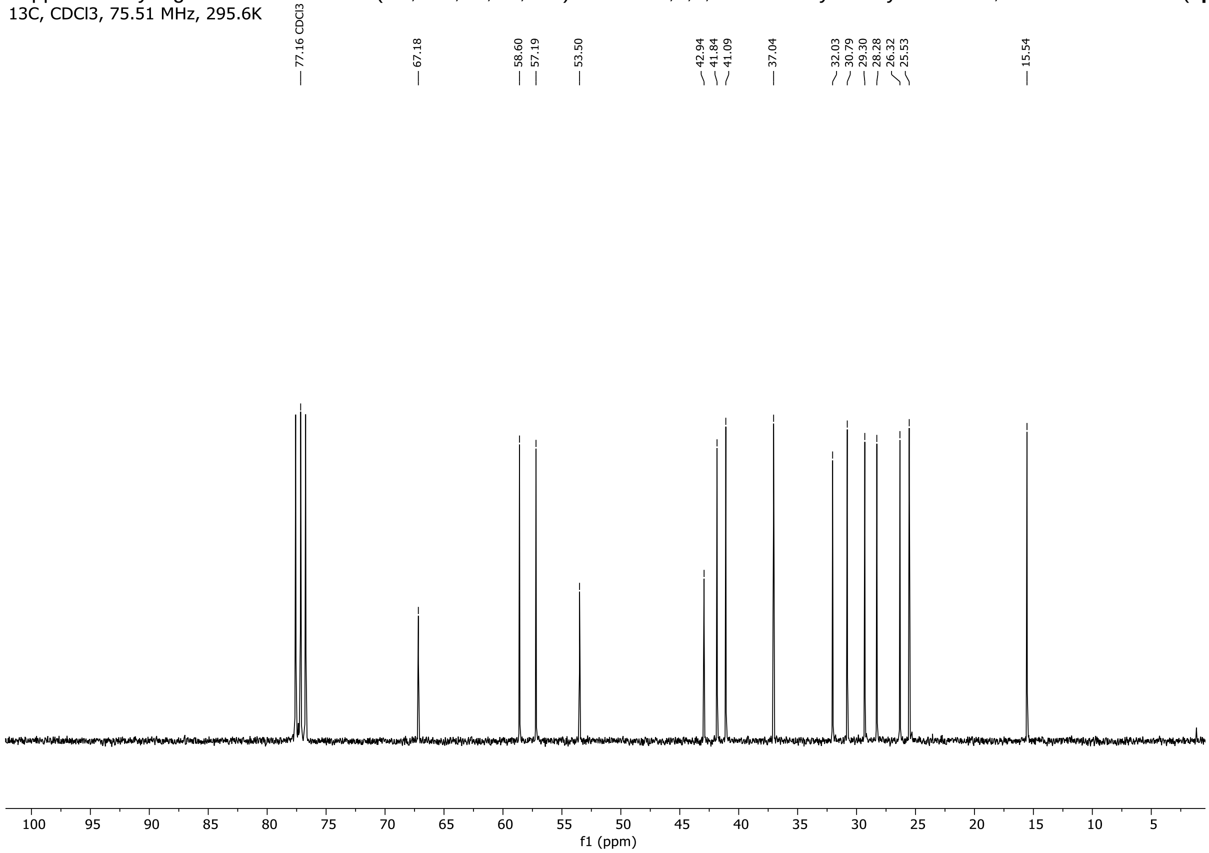

Supplementary Figure 110: ^{1}H NMR of (3*R*,3a*S*,6*S*,7*R*,8a*S*)-6-Azido-3,6,8,8-tetramethyloctahydro-1*H*-3a,7-methanoazulene (**8p**)

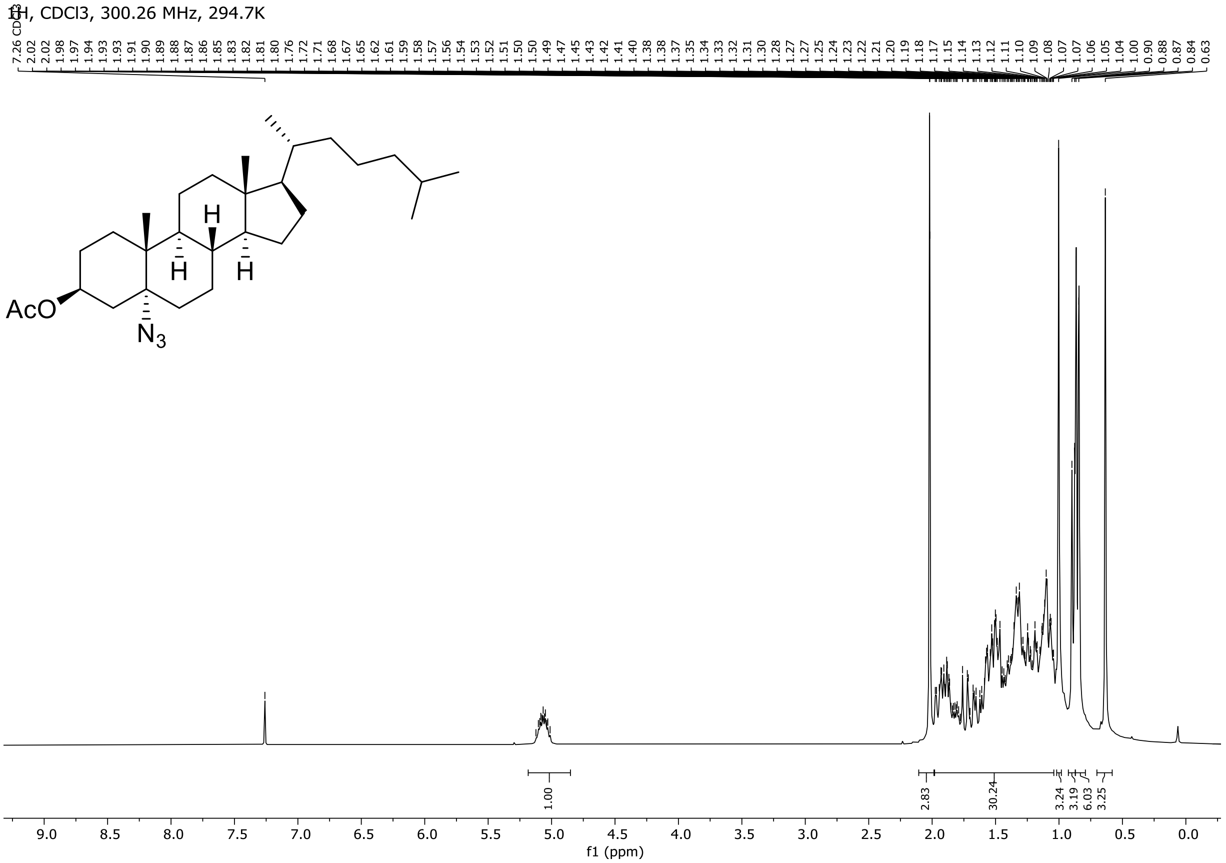

Supplementary Figure 111: ^{1}H NMR of (5*R*)-5-Azido-cholesteryl acetate (**8q**)

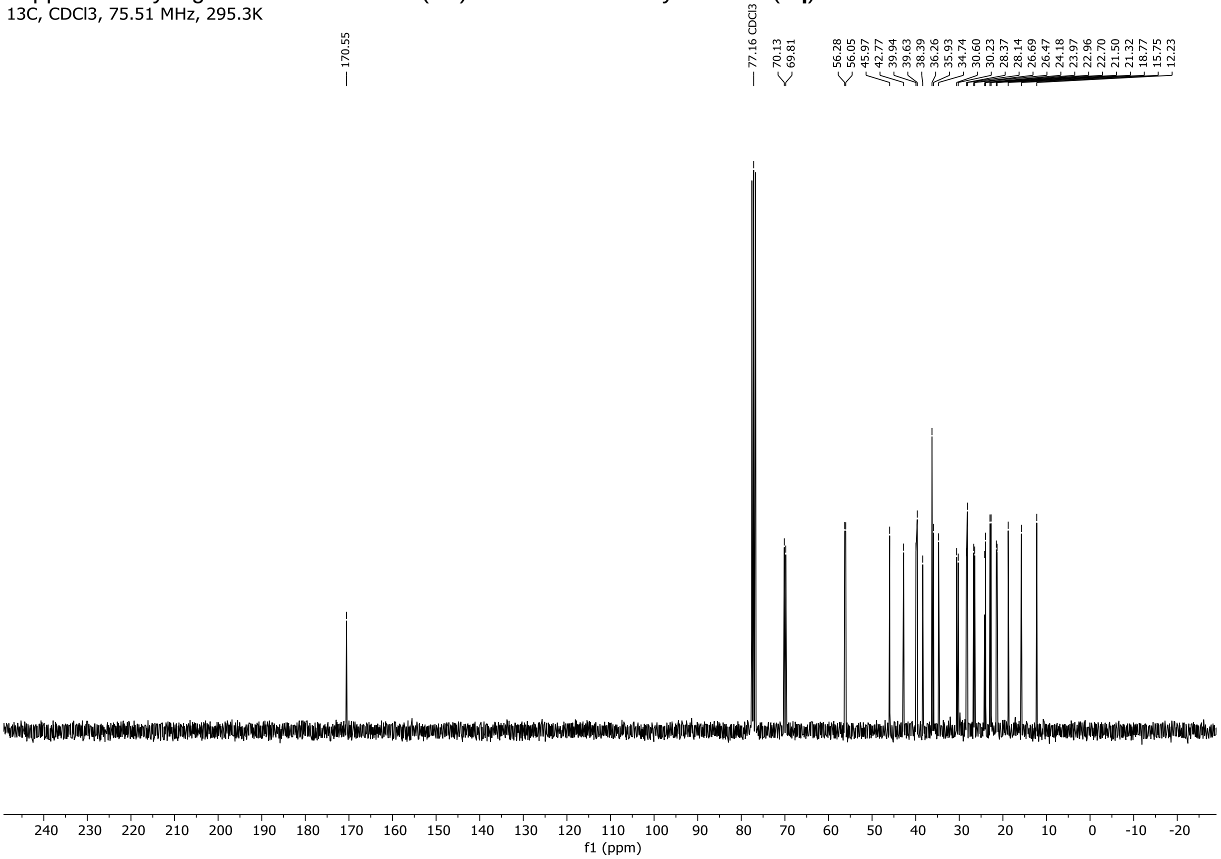

Supplementary Figure 112: ^{13}C NMR of (5*R*)-5-Azido-cholesteryl acetate (**8q**)

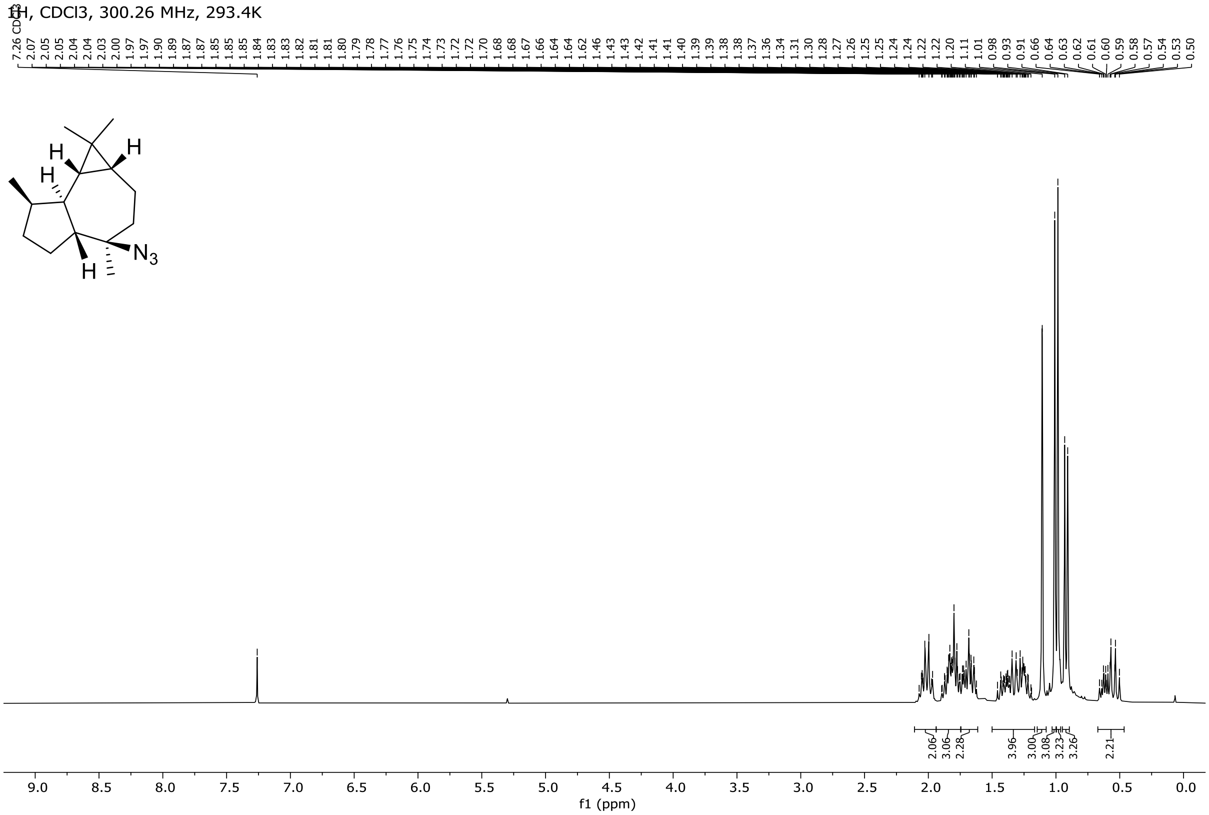

Supplementary Figure 113: ^{1}H NMR of (1a*R*,4*R*,4a*R*,7*R*,7a*S*,7b*S*)-4-Azido-1,1,4,7-tetramethyldecahydro-1*H*-cyclopropa[e]azulene (**8r-1**)

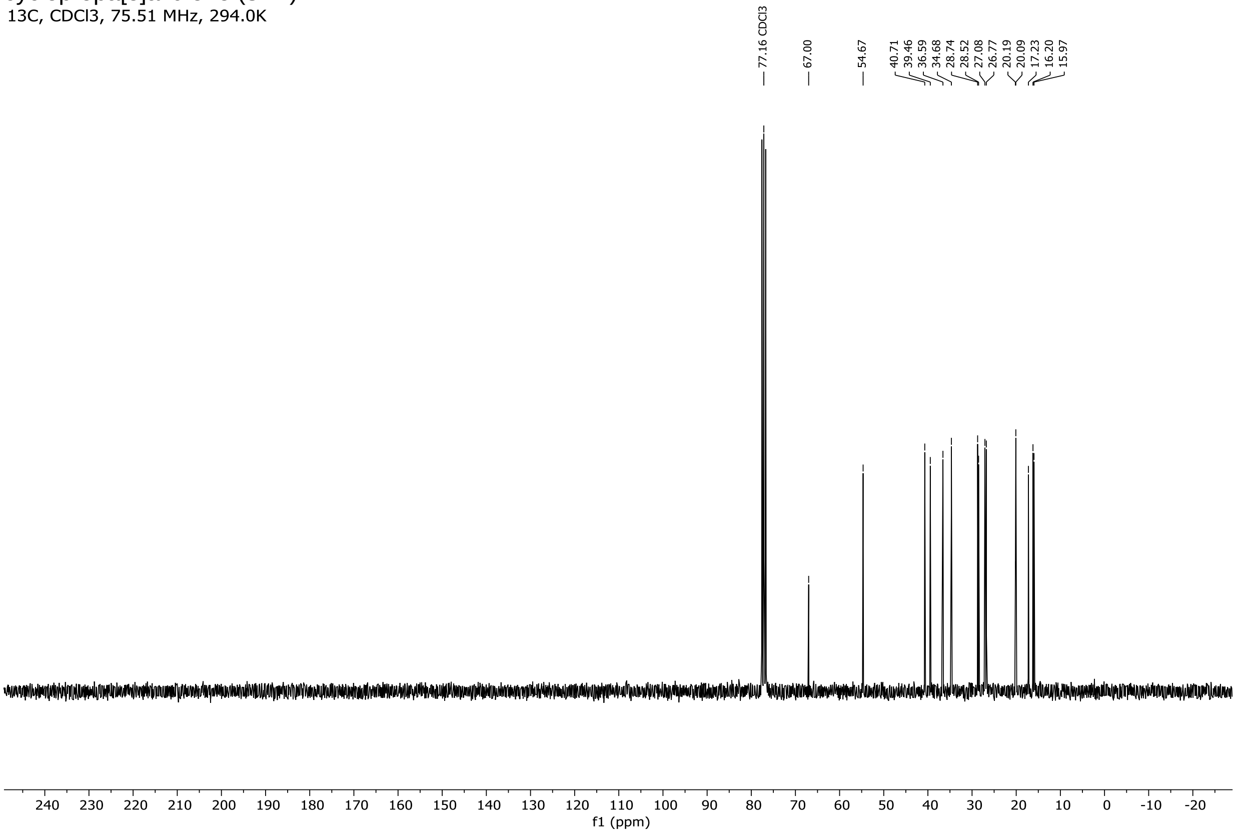

Supplementary Figure 114: ^{13}C NMR of (1a*R*,4*R*,4a*R*,7*R*,7a*S*,7b*S*)-4-Azido-1,1,4,7-tetramethyldecahydro-1*H*-cyclopropa[e]azulene (**8r-1**)

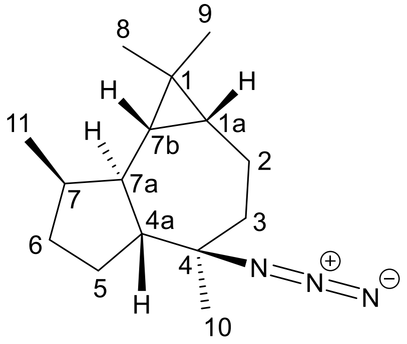

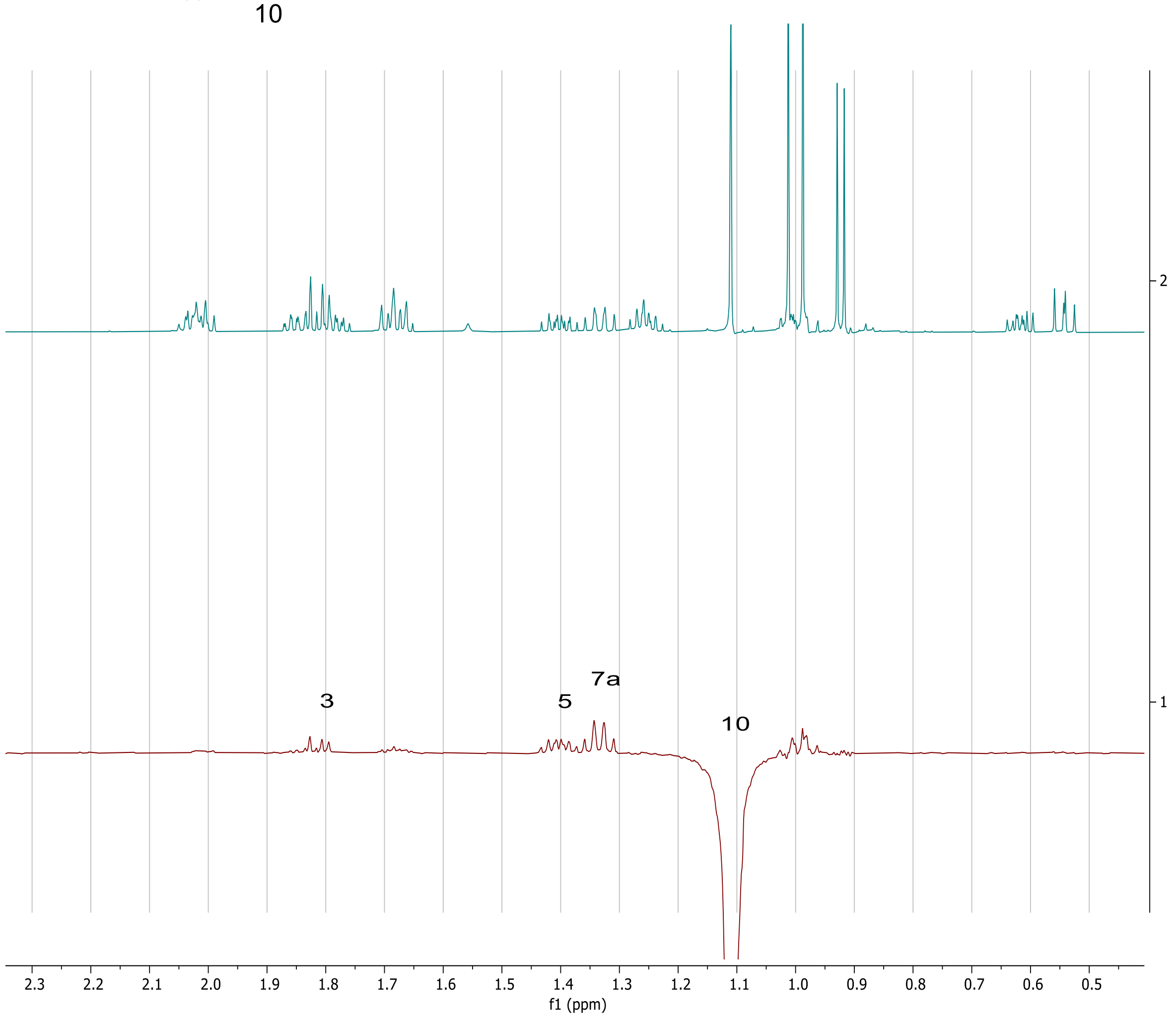

Supplementary Figure 115: 1D-NOESY NMR of (1a*R*,4*R*,4a*R*,7*R*,7a*S*,7b*S*)-4-Azido-1,1,4,7-tetramethyldecahydro-1*H*-cyclopropa[e]azulene (**8r-1**)

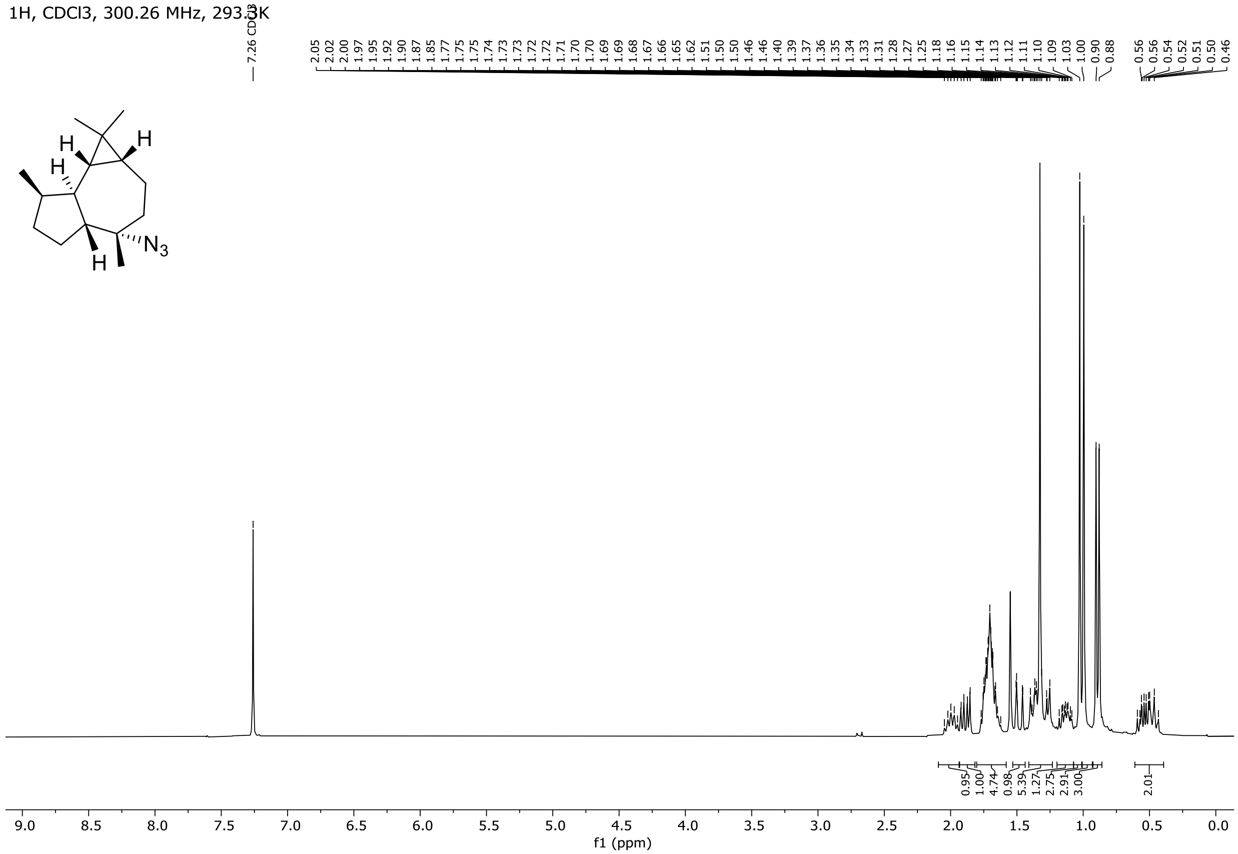

Supplementary Figure 116: ^{1}H NMR of (1a*R*,4*S*,4a*R*,7*R*,7a*S*,7b*S*)-4-azido-1,1,4,7-tetramethyldecahydro-1*H*-cyclopropa[e]azulene (**8r-2**)

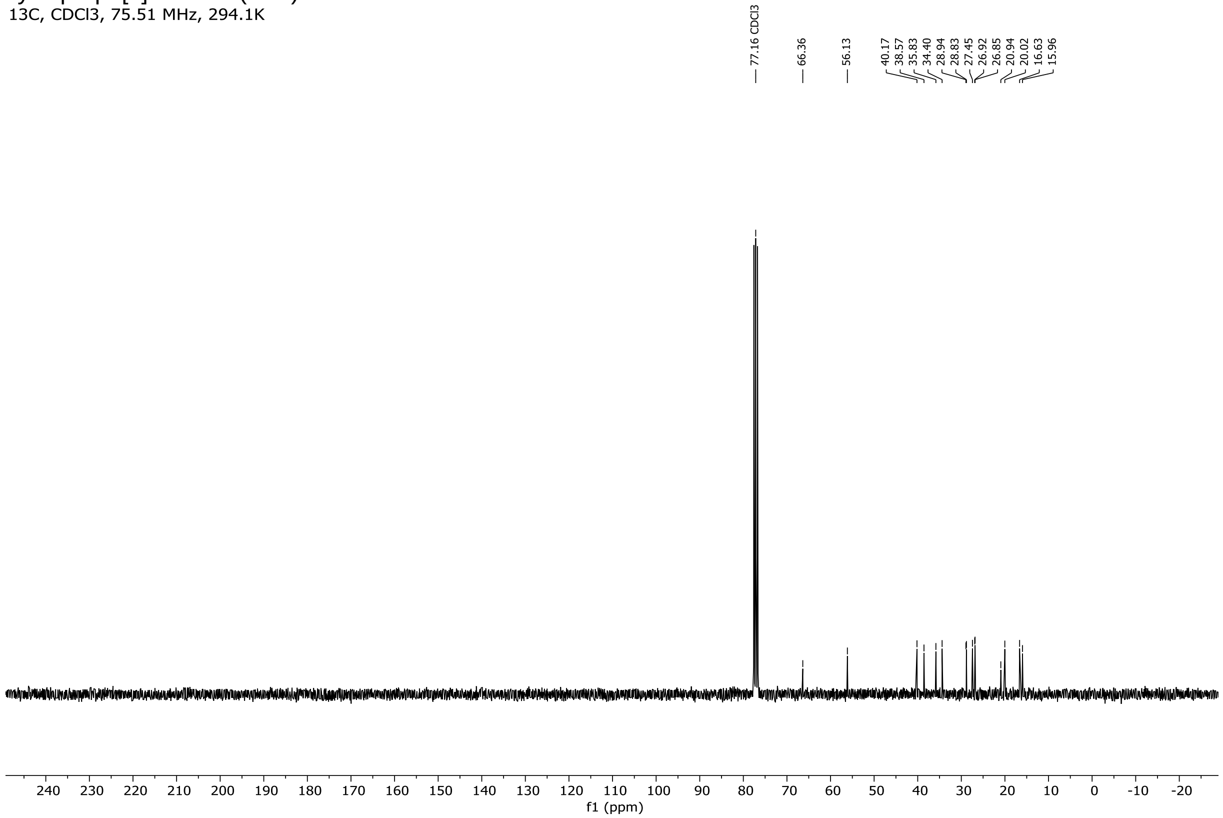

Supplementary Figure 117: ^{13}C NMR of (1a*R*,4*S*,4a*R*,7*R*,7a*S*,7b*S*)-4-azido-1,1,4,7-tetramethyldecahydro-1*H*-cyclopropa[e]azulene (**8r-2**)

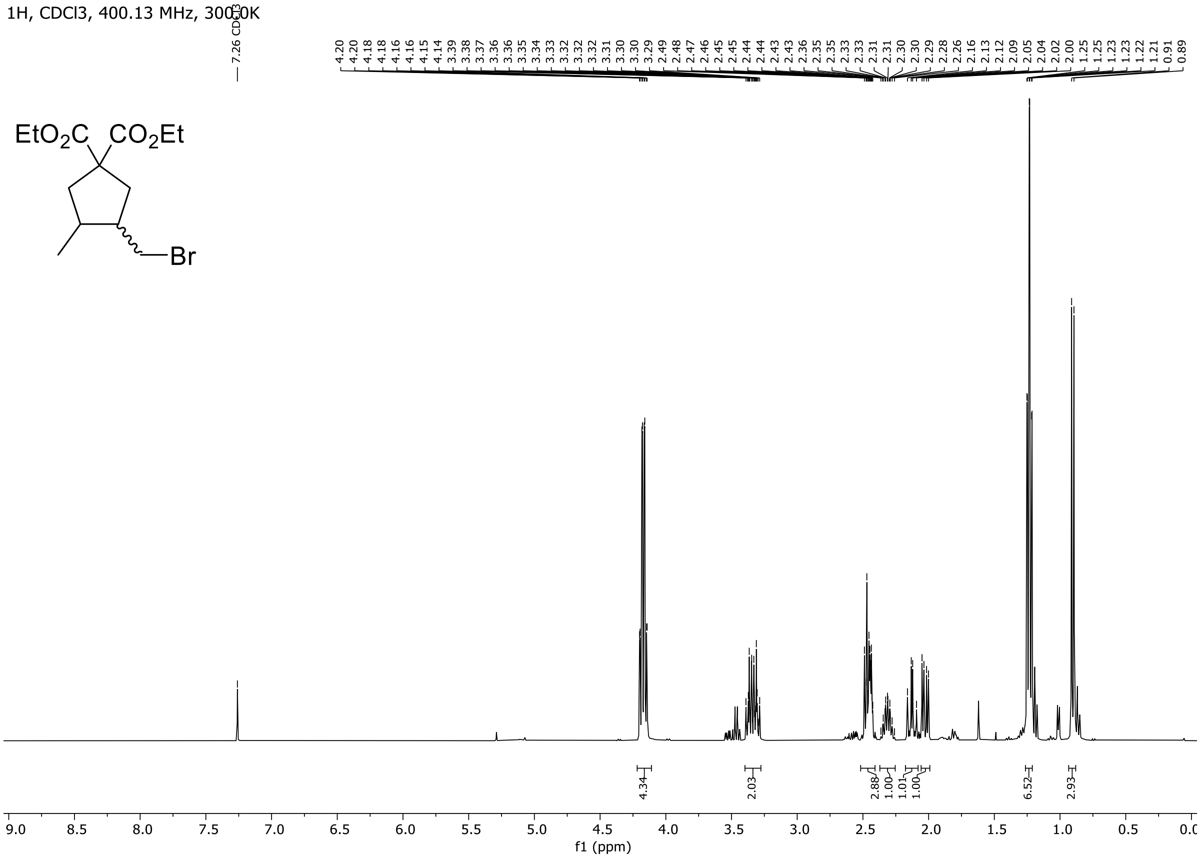

Supplementary Figure 118: ^{1}H NMR of Diethyl 3-(bromomethyl)-4-methylcyclopentane-1,1-dicarboxylate (**11**)

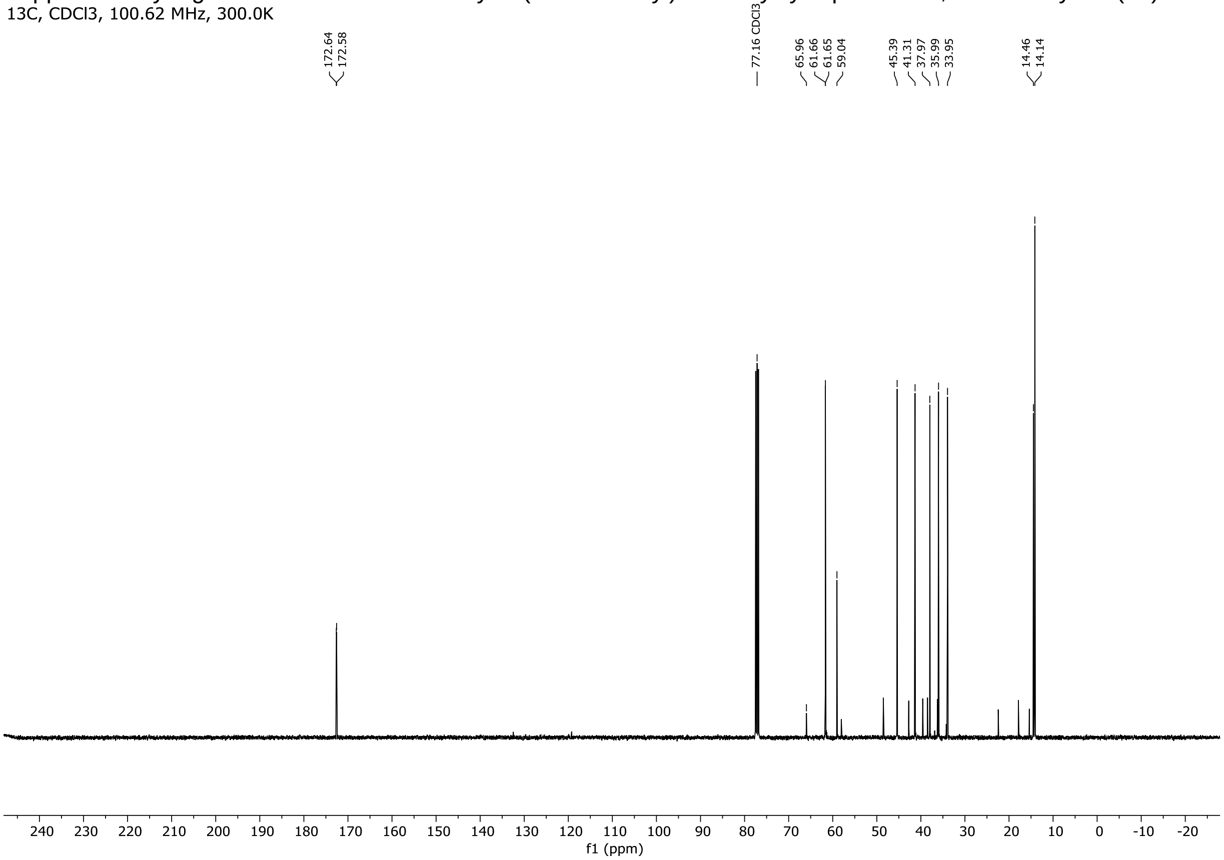

Supplementary Figure 119: ^{13}C NMR of Diethyl 3-(bromomethyl)-4-methylcyclopentane-1,1-dicarboxylate (**11**)

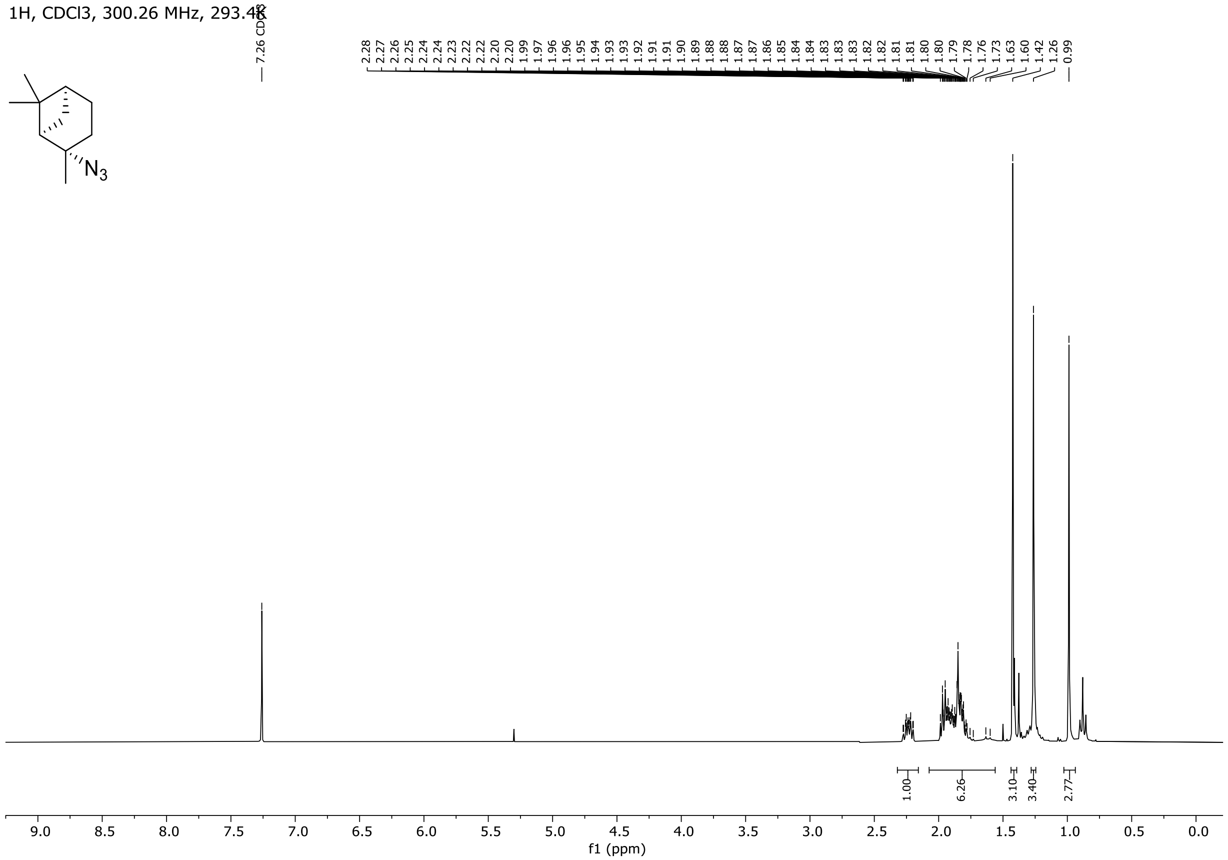

Supplementary Figure 120: ^{1}H NMR of (1*R*,5*S*)-2-Azido-2,6,6-trimethylbicyclo[3.1.1]heptane (**13a**)

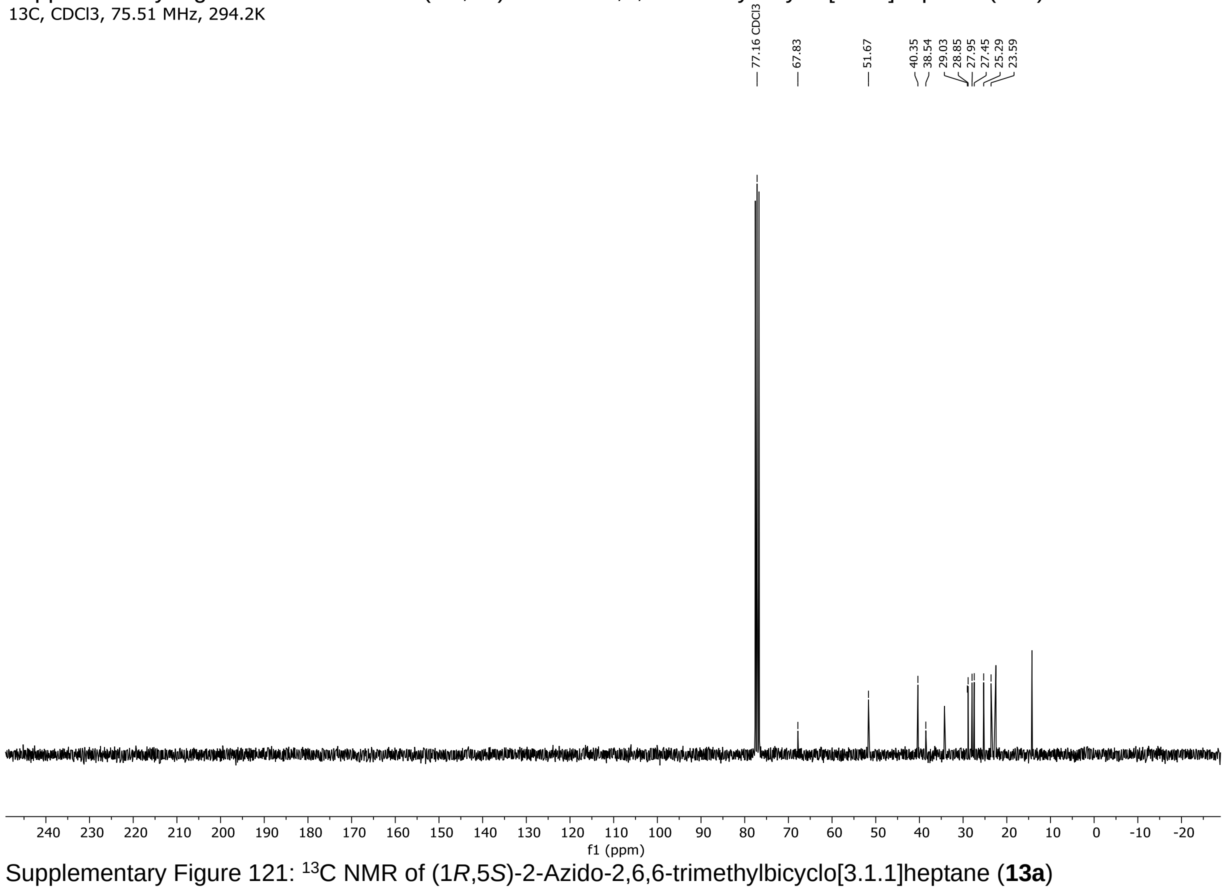

Supplementary Figure 121: ^{13}C NMR of (1*R*,5*S*)-2-Azido-2,6,6-trimethylbicyclo[3.1.1]heptane (**13a**)

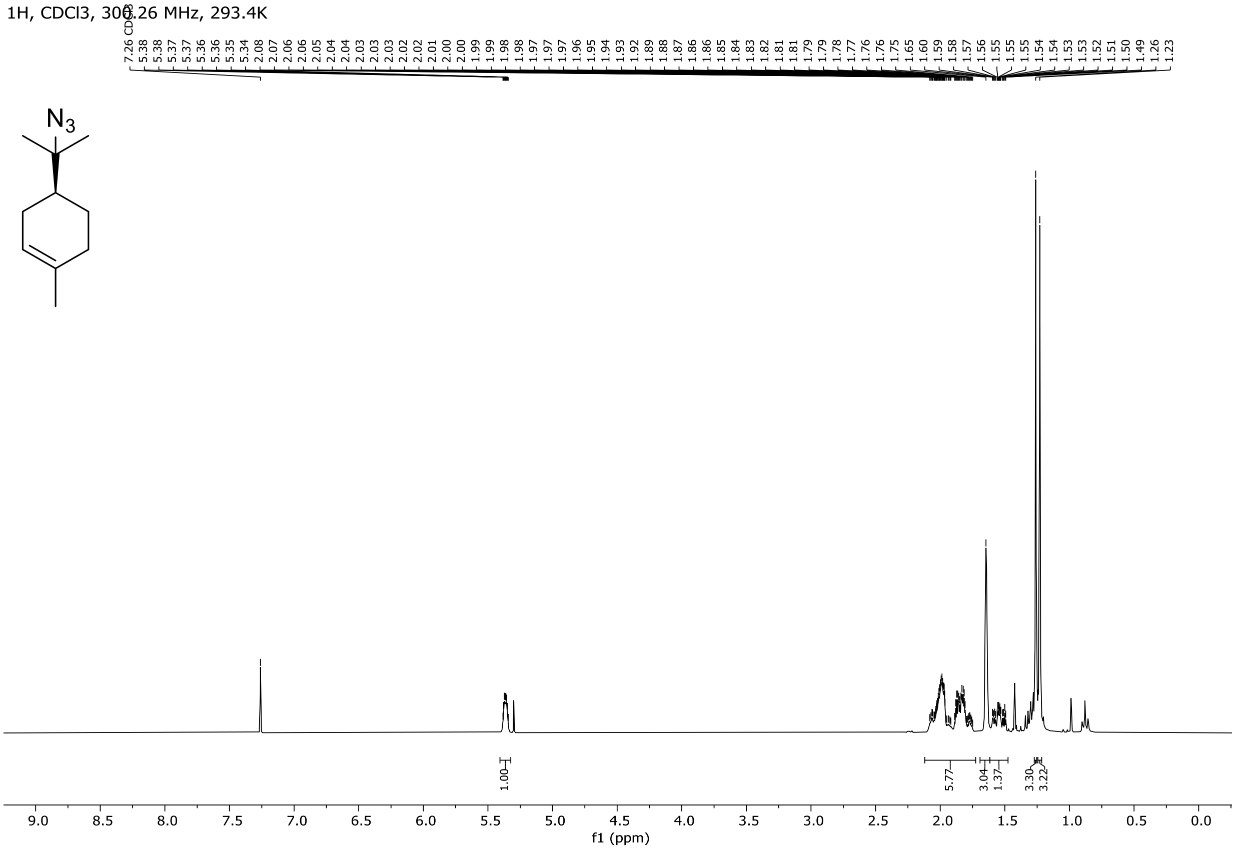

Supplementary Figure 121: ^{1}H NMR of (*S*)-4-(2-Azidopropan-2-yl)-1-methylcyclohex-1-ene (**13b**)

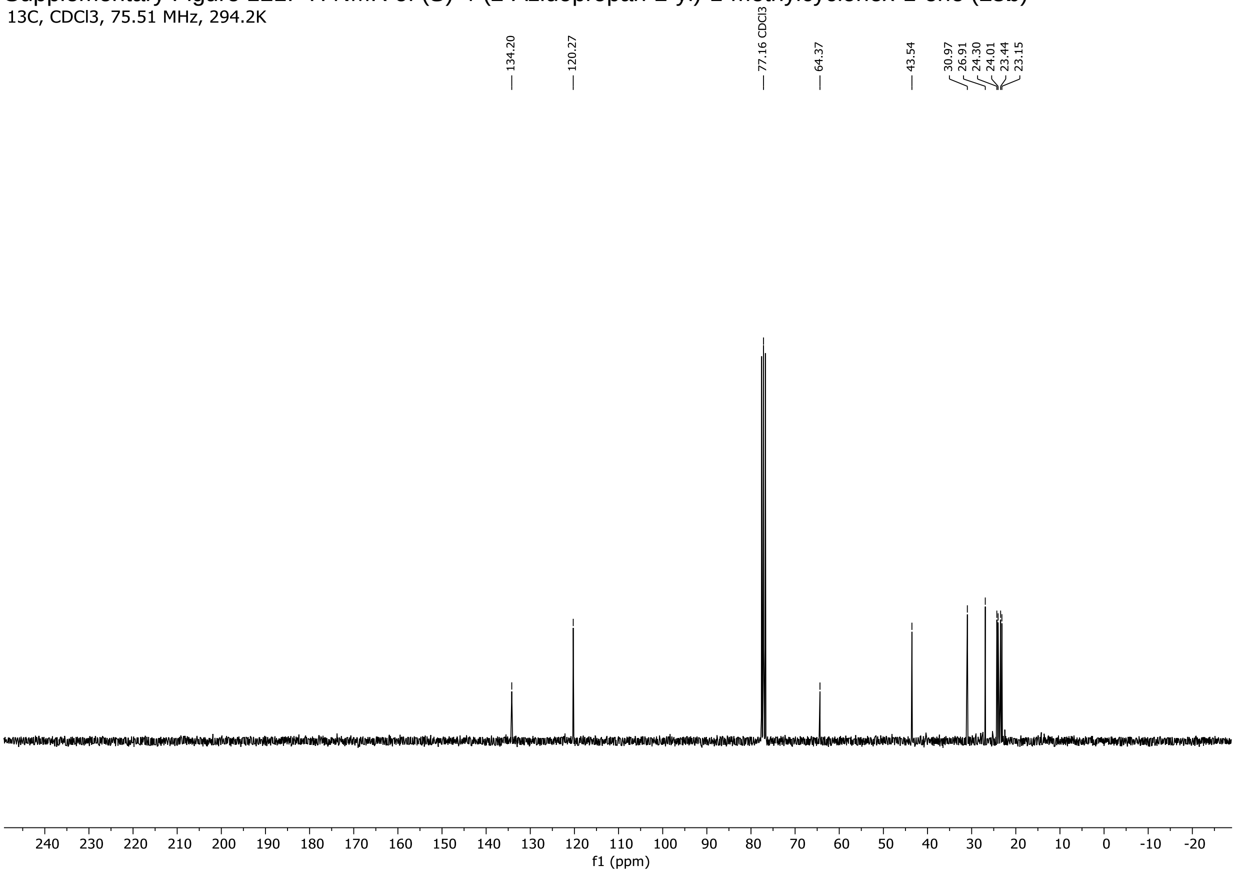

Supplementary Figure 122: ^{13}C NMR of (*S*)-4-(2-Azidopropan-2-yl)-1-methylcyclohex-1-ene (**13b**)

4. Supplementary References

1. Waser, J., Gaspar, B., Nambu, H. & Carreira, E. M. Hydrazines and Azides via the Metal-Catalyzed Hydrohydrazination and Hydroazidation of Olefins. *J. Am. Chem. Soc.* **128**, 11693–11712 (2006).
2. Dryzhakov, M., Hellal, M., Wolf, E., Falk, F. C. & Moran, J. Nitro-Assisted Brønsted Acid Catalysis: Application to a Challenging Catalytic Azidation. *J. Am. Chem. Soc.* **137**, 9555–9558 (2015).
3. Kim, D., Rahaman, S. M. W., Mercado, B. Q., Poli, R. & Holland, P. L. Roles of Iron Complexes in Catalytic Radical Alkene Cross-Coupling: A Computational and Mechanistic Study. *J. Am. Chem. Soc.* **141**, 7473–7485 (2019).
4. Zhao, B., Zhu, T., Ma, M. & Shi, Z. SOMOphilic Alkynylation of Unreactive Alkenes Enabled by Iron-Catalyzed Hydrogen Atom Transfer. *Molecules* **27**, 33 (2022).
5. Gaspar, B. & Carreira, E. M. Catalytic Hydrochlorination of Unactivated Olefins with para-Toluenesulfonyl Chloride. *Angew. Chem. Int. Ed.* **47**, 5758–5760 (2008).
6. Chen, X.-L., Dong, Y., Tang, L., Zhang, X.-M. & Wang, J.-Y. A Synthetic Strategy for 2-Alkylchromanones: Fe(III)-Catalyzed -Reductive Cross-Coupling of Unactivated Alkenes with Chromones. *Synlett* **29**, 1851–1856 (2018).
7. Zheng, J., Qi, J. & Cui, S. Fe-Catalyzed Olefin Hydroamination with Diazo Compounds for Hydrazone Synthesis. *Org. Lett.* **18**, 128–131 (2016).
8. Lang, M., Tardieu, D., Pousse, B., Compain, P. & Kern, N. Diastereoselective access to C , C -glycosyl amino acids via iron-catalyzed, auxiliary-enabled MHAT coupling. *Chem. Commun.* **60**, 3154–3157 (2024).
9. Mondal, B., Hazra, S., Chatterjee, A., Patel, M. & Saha, J. Fe-Catalyzed Hydroallylation of Unactivated Alkenes with Vinyl Cyclopropanes. *Org. Lett.* **25**, 5676–5681 (2023).
10. Dao, H. T., Li, C., Michaudel, Q., Maxwell, B. D. & Baran, P. S. Hydromethylation of Unactivated Olefins. *J. Am. Chem. Soc.* **137**, 8046–8049 (2015).
11. Zhang, Y. *et al.* Modular Synthesis of Alkylarylazo Compounds via Iron(III)-Catalyzed Olefin Hydroamination. *Org. Lett.* **21**, 2261–2264 (2019).
12. Bhunia, A., Bergander, K., Daniliuc, C. G. & Studer, A. Fe-Catalyzed Anaerobic Mukaiyama-Type Hydration of Alkenes using Nitroarenes. *Angew. Chem. Int. Ed.* **60**, 8313–8320 (2021).

13. Elfert, J., Bhunia, A., Daniliuc, C. G. & Studer, A. Intramolecular Radical Oxygen-Transfer Reactions Using Nitroarenes. *ACS Catal.* **13**, 6704–6709 (2023).

14. Gui, J. *et al.* Practical olefin hydroamination with nitroarenes. *Science* **348**, 886–891 (2015).

15. Shen, Y., Qi, J., Mao, Z. & Cui, S. Fe-Catalyzed Hydroalkylation of Olefins with para-Quinone Methides. *Org. Lett.* **18**, 2722–2725 (2016).

16. Saladrigas, M., Bosch, C., Saborit, G. V., Bonjoch, J. & Bradshaw, B. Radical Cyclization of Alkene-Tethered Ketones Initiated by Hydrogen-Atom Transfer. *Angew. Chem. Int. Ed.* **57**, 182–186 (2018).

17. Zhu, K., Shaver, M. P. & Thomas, S. P. Amine-bis(phenolate) Iron(III)-Catalyzed Formal Hydroamination of Olefins. *Chem. – Asian J.* **11**, 977–980 (2016).

18. Kong, L., Gan, X., van der Puyl Lovett, V. A. & Shenvi, R. A. Alkene Hydrobenzylation by a Single Catalyst That Mediates Iterative Outer-Sphere Steps. *J. Am. Chem. Soc.* **146**, 2351–2357 (2024).

19. Gan, X. *et al.* Iron-Catalyzed Hydrobenzylation: Stereoselective Synthesis of (−)-Eugenial C. *J. Am. Chem. Soc.* **145**, 15714–15720 (2023).

20. Saladrigas, M., Puig, J., Bonjoch, J. & Bradshaw, B. Iron-Catalyzed Radical Intermolecular Addition of Unbiased Alkenes to Aldehydes. *Org. Lett.* **22**, 8111–8115 (2020).

21. Xie, Y. *et al.* Ligand-Promoted Iron(III)-Catalyzed Hydrofluorination of Alkenes. *Angew. Chem. Int. Ed.* **58**, 7097–7101 (2019).

22. Combettes, L. E. *et al.* Synthesis of 3-Fluoropyrrolidines and 4-Fluoropyrrolidin-2-ones from Allylic Fluorides. *Chem. – Eur. J.* **18**, 13126–13132 (2012).

23. Hou, Z.-W., Zhang, M.-M., Yang, W.-C. & Wang, L. Catalyst- and Oxidizing Reagent-Free Electrochemical Benzylic C(sp3)–H Oxidation of Phenol Derivatives. *J. Org. Chem.* **87**, 7806–7817 (2022).

24. Obradors, C., Martinez, R. M. & Shenvi, R. A. Ph(i-PrO)SiH2: An Exceptional Reductant for Metal-Catalyzed Hydrogen Atom Transfers. *J. Am. Chem. Soc.* **138**, 4962–4971 (2016).

25. Li, Y. *et al.* Nickel-catalyzed migratory alkyl–alkyl cross-coupling reaction. *Chem. Sci.* **11**, 10461–10464 (2020).

26. Chen, H., Jia, X., Yu, Y., Qian, Q. & Gong, H. Nickel-Catalyzed Reductive Allylation of Tertiary Alkyl Halides with Allylic Carbonates. *Angew. Chem. Int. Ed.* **56**, 13103–13106 (2017).

27. Someya, H., Yorimitsu, H. & Oshima, K. Silver-catalyzed cross-coupling reactions of alkyl bromides with alkyl or aryl Grignard reagents. *Tetrahedron Lett.* **50**, 3270–3272 (2009).
28. Wu, J., Shu, C., Li, Z., Noble, A. & Aggarwal, V. K. Photoredox-Catalyzed Decarboxylative Bromination, Chlorination and Thiocyanation Using Inorganic Salts. *Angew. Chem. Int. Ed.* **62**, e202309684 (2023).
29. Wang, Y., Li, G.-X., Yang, G., He, G. & Chen, G. A visible-light-promoted radical reaction system for azidation and halogenation of tertiary aliphatic C–H bonds. *Chem. Sci.* **7**, 2679–2683 (2016).
30. Wang, G.-Z., Shang, R., Cheng, W.-M. & Fu, Y. Irradiation-Induced Heck Reaction of Unactivated Alkyl Halides at Room Temperature. *J. Am. Chem. Soc.* **139**, 18307–18312 (2017).
31. Dudnik, A. S. & Fu, G. C. Nickel-Catalyzed Coupling Reactions of Alkyl Electrophiles, Including Unactivated Tertiary Halides, To Generate Carbon–Boron Bonds. *J. Am. Chem. Soc.* **134**, 10693–10697 (2012).
32. Lv, X.-Y. & Martin, R. Cu-Catalyzed C(sp3) Amination of Unactivated Secondary Alkyl Iodides Promoted by Diaryliodonium Salts. *Org. Lett.* **25**, 3750–3754 (2023).
33. Liu, C., Zhang, Z., Zhao, L.-L., Bertrand, G. & Yan, X. Mesoionic Carbene-Catalyzed Formyl Alkylation of Aldehydes. *Angew. Chem. Int. Ed.* **62**, e202303478 (2023).
34. Han, L., Xia, J.-B., You, L. & Chen, C. Ketone-catalyzed photochemical C(sp3)–H chlorination. *Tetrahedron* **73**, 3696–3701 (2017).
35. Barker, T. J. & Boger, D. L. Fe(III)/NaBH4-Mediated Free Radical Hydrofluorination of Unactivated Alkenes. *J. Am. Chem. Soc.* **134**, 13588–13591 (2012).
36. Leggans, E. K., Barker, T. J., Duncan, K. K. & Boger, D. L. Iron(III)/NaBH4-Mediated Additions to Unactivated Alkenes: Synthesis of Novel 20′-Vinblastine Analogues. *Org. Lett.* **14**, 1428–1431 (2012).
37. Ardiansah, B., Tanimoto, H., Tomohiro, T., Morimoto, T. & Kakiuchi, K. Sulfonium ion-promoted traceless Schmidt reaction of alkyl azides. *Chem. Commun.* **57**, 8738–8741 (2021).
38. Bruker AXS. APEX4 Version 2021.4-0, SAINT Version 8.40B and SADABS Bruker AXS area detector scaling and absorption correction Version 2016/2. Bruker AXS Inc., Madison, Wisconsin, USA (2021).
39. Sheldrick, G. M. SHELXT – Integrated space-group and crystal-structure determination. *Acta Cryst* **A71**, 3–8 (2015).
40. Sheldrick, G. M. Crystal structure refinement with SHELXL. *Acta Cryst* **C71**, 3–8 (2015).

41. Bruker AXS. Interactive molecular graphics, Version 5.1. Bruker AXS Inc., Madison, Wisconsin, USA. (1998).

42. Zhang, M. *et al.* A hydrate salt-promoted reductive coupling reaction of nitrodienes with unactivated alkenes. *Org. Biomol. Chem.* **17**, 2258–2264 (2019).
